# Herpes simplex virus 1 harboring poly(T) DNA sequences as a key ligand for AIM2 inflammasome activation and host defense

**DOI:** 10.1038/s41467-026-71896-w

**Published:** 2026-04-13

**Authors:** SuHyeon Oh, Jueun Oh, Kyeongchan Im, Tae Hyoung Kim, Jihye Lee, Kihye Shin, Nabukenya Mariam, Cheong Seok, Jaewoo Park, GyeongJu Yu, Hayeon Kim, Suhyun Kim, Seyun Shin, Jinwoo Gil, Sehee Park, Yoon-Seok Chung, Daesik Kim, Young Ki Choi, Eui Tae Kim, Joo Sang Lee, SangJoon Lee

**Affiliations:** 1 Department of Biological Science, Ulsan National Institute of Science and Technology (UNIST), Ulsan, Republic of Korea; 2 Center for Study of Emerging and Re-emerging Viruses, Korea Virus Research Institute, Institute for Basic Science (IBS), Daejeon, Republic of Korea; 3 Department of Precision Medicine, School of Medicine, Sungkyunkwan University, Suwon, Republic of Korea; 4 Department of Biomedicine & Drug Development, Jeju National University Graduate School, Jeju, Republic of Korea; 5 Department of Microbiology and Immunology, Jeju National University College of Medicine, Jeju, Republic of Korea; 6 Jeju Research Center for Natural Medicine, Jeju National University Core Research Institute, Jeju, Republic of Korea; 7 Division of Acute Viral Disease, Center for Emerging Virus Research, National Institute of Infectious Diseases, National Institute of Health, Cheongju, Republic of Korea; 8 Division of High-Risk Pathogens, Department of Laboratory Diagnosis and Analysis, Korea Disease Control and Prevention Agency, KDCA, Cheongju, Republic of Korea; 9 Department of Digital Health, Samsung Advanced Institute of Health Science and Technology and Department of Artificial Intelligence, Sungkyunkwan University, Suwon, Republic of Korea; 10 Graduate School of Health Science and Technology, Ulsan National Institute of Science and Technology (UNIST), Ulsan, Republic of Korea

**Keywords:** Immune cell death, Infection, NOD-like receptors

## Abstract

Herpes simplex virus type 1 (HSV-1) infection remains a major global health challenge, yet the mechanisms underlying strain-specific innate immune responses are poorly understood. Here, we show that distinct HSV-1 strains differentially activate the absent in melanoma 2 (AIM2) inflammasome. The HF strain robustly induces AIM2-dependent inflammasome activation, whereas the F and KOS strains elicit minimal responses despite comparable infection efficiency. We demonstrate that this difference is driven by viral genomic features rather than replication capacity. Genomic analyses identify a poly(T) DNA sequence within the UL25-UL26 intergenic region that is enriched in the HF strain. Deletion of a 14-mer poly(T) sequence markedly impairs inflammasome activation, cytokine release, and host protection in vivo, whereas introduction of a poly(T) tract into the F strain is sufficient to confer AIM2 activation and enhanced host defense. Furthermore, poly(T)-mediated AIM2 activation is length-dependent, conserved in human macrophages, and requires a cGAS-STING-IRF1 licensing axis. Together, these findings identify viral poly(T) DNA as a key determinant of strain-specific AIM2 inflammasome activation and reveal how viral genomic variation shapes innate immune recognition.

## Abstract (web summary)

This study identifies viral poly(T) DNA as a key determinant of AIM2 inflammasome activation, revealing how viral genome variation shapes inflammatory responses and host survival during infection.

## Introduction

Herpes simplex virus type 1 (HSV-1) infection poses a significant global public health challenge, affecting approximately 67% of the global population. With a genome spanning 152 kbp of linear double-stranded DNA (dsDNA), HSV-1 encodes a diverse repertoire of at least 84 viral proteins. Although antiviral therapies can suppress viral replication and alleviate symptoms, HSV-1 establishes lifelong latency and periodically reactivates, underscoring the absence of curative strategies and the need for a deeper understanding of virus-host interactions that govern immune control and pathogenesis^1,2^. Upon infiltration into host cells, HSV-1 triggers the prompt activation of innate immunity and subsequent production of inflammatory cytokines and cell death, crucial for orchestrating a natural antiviral immune response essential for viral clearance and preventing prolonged infection.

Among innate immune defense mechanisms, inflammasome activation plays a pivotal role in detecting intracellular pathogens and initiating inflammatory cell death. Inflammasomes are multiprotein complexes that assemble in response to pathogen-associated molecular patterns (PAMPs) or damage-associated molecular patterns (DAMPs), leading to caspase-1 activation, maturation of interleukin-1β (IL-1β) and interleukin-18 (IL-18), and induction of pyroptosis^3–5^. These responses eliminate infected cells and amplify antiviral immunity. Several cytosolic inflammasome sensors have been characterized, including NOD-like receptor family pyrin domain-containing 3 (NLRP3), NOD-like receptor family CARD domain-containing protein 4 (NLRC4), and absent in melanoma 2 (AIM2), each responding to distinct molecular triggers. Notably, AIM2 directly binds cytosolic dsDNA through its hematopoietic interferon-inducible nuclear domain, forming an inflammasome complex that is essential for host defense against intracellular bacteria and DNA viruses^6–12^.

HSV-1 research has revealed a complex interplay of evolutionary nuances and genetic diversity, intricately influencing its interactions with the host immune system^1,2^. A notable aspect of this complexity lies in the differential activation of the AIM2 inflammasome by distinct HSV-1 strains. For example, while the F strain exhibits nuanced control over AIM2 inflammasome activation through the actions of viral protein 22 (VP22)^13,14^, the HF strain robustly stimulates AIM2 inflammasome activation and inflammatory cell death^7,11^. This distinction underscores the intricate interplay between viral genetics and host immune response, shedding light on the multifaceted dynamics governing HSV-1 pathogenesis. To date, no hypotheses or claims have been proposed to explain these strain-specific differences, suggesting a need for further research to deepen our understanding of the interactions between HSV-1 and the host immune system.

To elucidate the mechanisms of AIM2 inflammasome activation by distinct HSV-1 strains, we screened specific DNA ligands and identified 14-base pair (bp) poly(T) DNA sequences as key ligands of HSV-1 for AIM2 inflammasome activation and host defense. These findings advance our understanding of innate immunity, inflammasomes, and HSV-1 pathogenesis, paving the way for the development of novel therapeutics against HSV-1.

## Results

### HSV-1 HF strain selectively triggers AIM2 inflammasome activation and inflammatory cell death

**Fig. 1:**
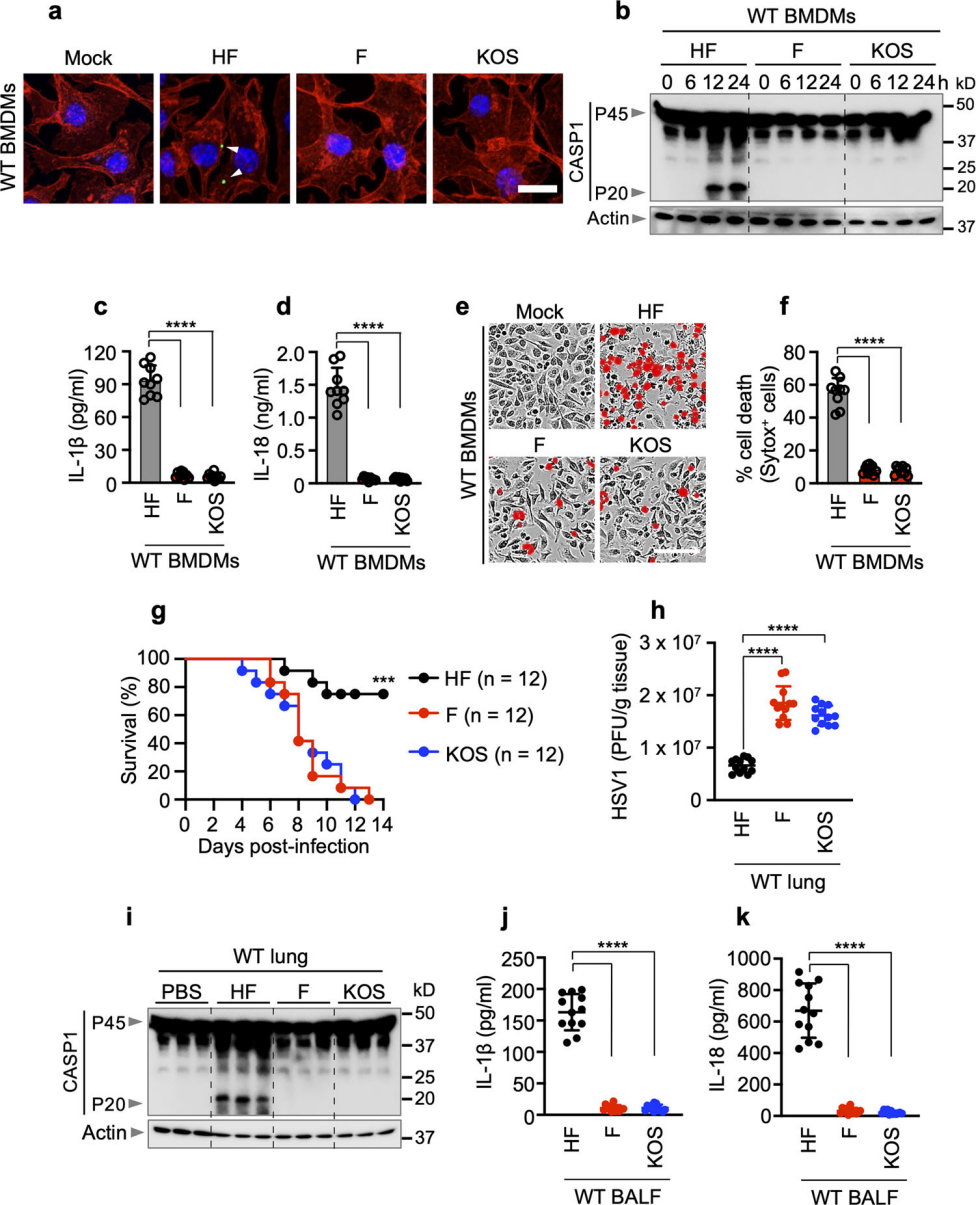
HSV-1, specifically the HF strain, triggers inflammasome activation and cell death. **a** Immunofluorescence (IF) images of wild-type (WT) bone marrow-derived macrophages (BMDMs) after infection with herpes simplex virus 1 (HSV-1) strains: HF, F, and KOS. Arrowheads indicate ASC specks. Scale bar: 10 μm. **b** Immunoblot analysis of pro-caspase-1 (CASP1; P45) and cleaved CASP1 (P20) in WT BMDMs over time after infection with different types of HSV-1 strains. **c**, **d** Interleukin-1 beta (IL-1β) (**c**) and IL-18 (**d**) release assessment in WT BMDMs following HSV-1 infection. **e** Cell death evaluation in BMDMs after HSV-1 infection. Dead cells are indicated in red. Scale bar: 100 μm. **f** Quantification of cell death in (**e**). **g** Survival of WT mice infected intranasally with 5 × 10^5^ plaque-forming units (PFU) of HSV-1 strains: HF, F, and KOS. ***P < 0.001 (HF strain versus F or KOS strain), log-rank test (Mantel-Cox). Survival data are pooled from two independent experiments. *n* = 12 mice per group. **h** Pulmonary viral titer at day 5 after infection with different strains of HSV-1. **i** Immunoblot analysis of pro- (P45) and activated (P20) caspase-1 (CASP1) in lung tissues from uninfected animals (PBS) or WT mice 5 days after HSV-1 infection. Each lane indicates independent biological replicates. IL-1β (**j**) and IL-18 (**k**) release assessment in bronchoalveolar lavage fluid (BALF) of the WT mice following HSV-1 infection. **a**, **e** Images representative of three biologically independent experiments. **c**, **d**, **f** Data as mean ± s.e.m. ****P < 0.0001 (one-way ANOVA with Dunnett’s multiple comparisons test; *n* = 9 biologically independent samples from three independent experiments). **b**, **i** Data from three independent experiments. Expected molecular weights: pro-caspase-1 (P45, ~45 kDa) and cleaved caspase-1 (P20, ~20 kDa). **h**, **j**, **k** Data as mean ± s.e.m. ****P < 0.0001 (one-way ANOVA with Dunnett’s multiple comparisons test; *n* = 12 mice per group; each symbol represents one biological replicate (one mouse)). Data are pooled from two independent experiments. Source data are provided as a Source data file.

To elucidate HSV-1 involvement in apoptosis-associated speck-like protein containing a CARD (ASC) inflammasome activation, we performed immunofluorescence staining using antibodies against ASC and tubulin. ASC speck-positive cells were observed following infection with three distinct HSV-1 strains: HF, F, and KOS. Notably, infection by the HF strain led to ASC speck formation, whereas the F and KOS strains did not induce this phenomenon (Fig. 1a). Furthermore, HSV-1 infection prompted caspase-1 cleavage (Fig. 1b), release of inflammasome-driven cytokines, IL-1β (Fig. 1c), and IL-18 (Fig. 1d), as well as cell death (Fig. 1e, f), in an HF strain-dependent manner.

Importantly, HSV-1 HF, F, and KOS strains exhibited comparable viral protein expression kinetics in both HaCaT cells and primary wild-type (WT) or *Aim2*^*−/−*^ bone marrow-derived macrophages (BMDMs), indicating similar infection efficiency across strains (Supplementary Fig. S1a, b). Subsequently, we extended our observations to in vivo models of HSV-1 infection with the different strains. While WT mice succumbed to F and KOS strain infections within 13 days, approximately 80% of WT mice infected with the HF strain survived (Fig. 1g). Furthermore, plaque assays of lung tissue homogenates from infected mice revealed significantly lower viral titers in the HF strain compared with those in the F and KOS strains (Fig. 1h). Lung lysates from infected WT mice exhibited caspase-1 cleavage (Fig. 1i), and IL-1β (Fig. 1j) and IL-18 (Fig. 1k) were detected in bronchoalveolar lavage fluid (BALF) in an HF strain-dependent manner. These results indicate that inflammasome activation and cell death are specifically induced by infection with the HF strain of HSV-1.

**Fig. 2:**
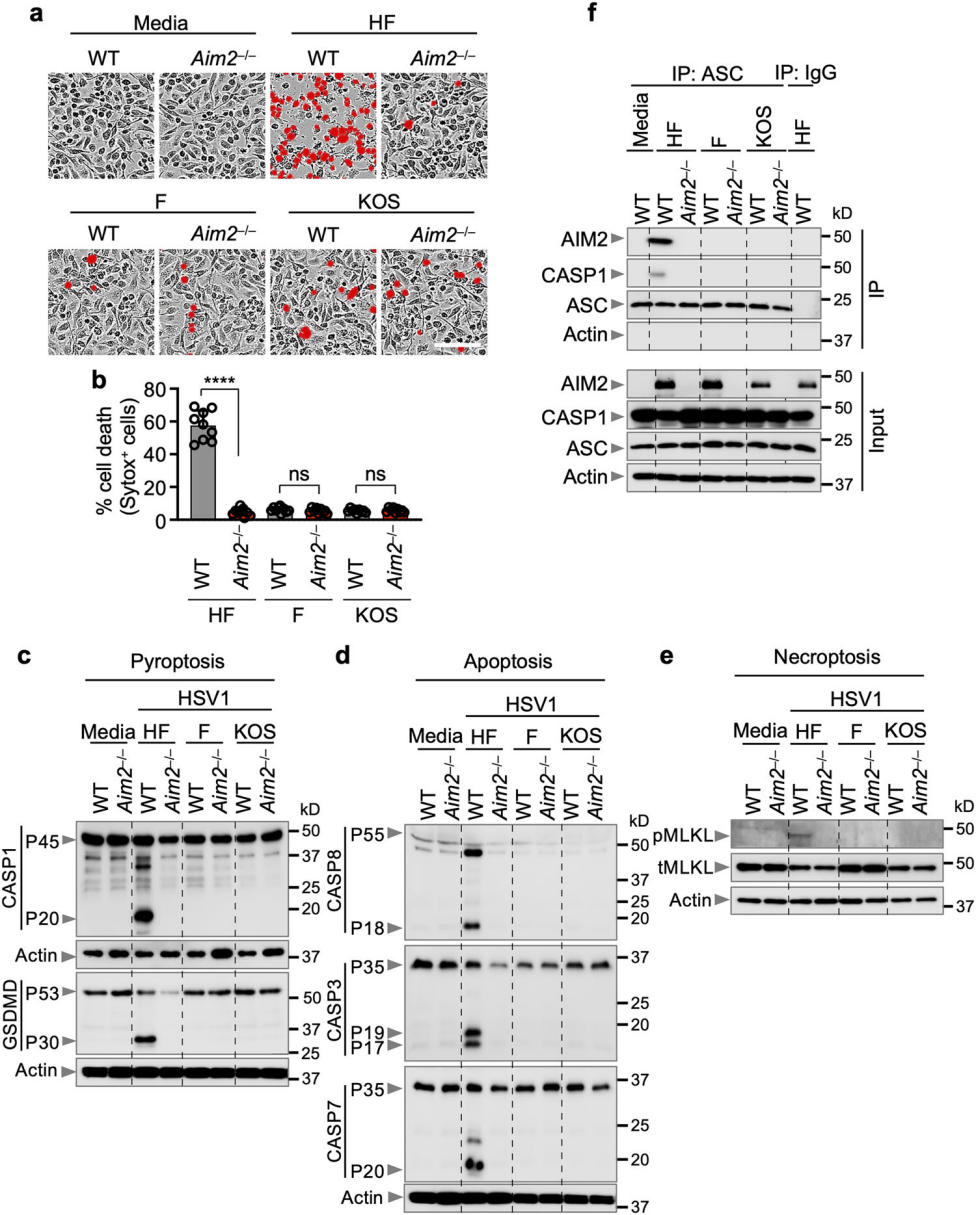
HF strain of HSV-1 triggers inflammatory cell death in an AIM2-dependent manner. **a** Cell death evaluation in wild-type (WT) or *Aim2*^−/−^ bone marrow-derived macrophages (BMDMs) after infection with herpes simplex virus 1 (HSV-1) strains: HF, F, and KOS. Dead cells are indicated in red. Scale bar: 100 μm. Images are representative of three biologically independent experiments. **b** Quantification of cell death in (**a**). Data are mean ± s.e.m. “ns” not significant; ****P < 0.0001 (one-way ANOVA with Dunnett’s multiple comparisons test; *n* = 9 biologically independent samples from three independent experiments). **c–e** Immunoblot analysis of pro- (P45) and activated (P20) caspase-1 (CASP1), pro- (P53) and activated (P30) GSDMD (**c**); pro- (P55) and cleaved (P18) caspase-8 (CASP8), pro- (P35) and cleaved (P17/P19) caspase-3 (CASP3), pro- (P35) and cleaved (P20) caspase-7 (CASP7) (**d**); phosphorylated MLKL (pMLKL), total MLKL (tMLKL) (**e**) in WT and *Aim2*^*−/−*^ BMDMs after infection with different strains of HSV-1. Expected molecular weights: CASP1 (P45 ~45 kDa; P20 ~20 kDa), GSDMD (P53 ~53 kDa; P30 ~30 kDa), CASP8 (P55 ~55 kDa; P18 ~18 kDa), CASP3 (P35 ~35 kDa; P17/P19 ~17–19 kDa), CASP7 (P35 ~35 kDa; P20 ~20 kDa), MLKL (~54 kDa). Data are representative of three independent experiments. **f** Immunoprecipitation (IP) in WT or *Aim2*^−/−^ BMDMs with IgG control antibodies or anti-ASC antibodies after infection with different strains of HSV-1. Data are representative of three independent experiments. Source data are provided as a Source data file.

To investigate whether cells infected with the HF strain of HSV-1 engage the inflammasome sensor, WT BMDMs and BMDMs singly deficient in several major inflammasome sensors were infected with HSV-1. Infection with the HF strain of HSV-1 induced AIM2-dependent cleavage of caspase-1 and cell death, independent of NLRP3 and NLRC4 (Supplementary Fig. S2a-i), suggesting that AIM2 serves as an inflammasome sensor for the HF strain of HSV-1. To determine AIM2 involvement in the F and KOS strains of HSV-1, we infected WT and AIM2-deficient BMDMs with HF, F, and KOS strains. Infection with the HF strain induced AIM2-dependent cell death, whereas the F and KOS strains did not induce cell death in either WT or AIM2-deficient BMDMs (Fig. 2a, b).

In addition to inflammasome activation, various pathogens and sterile triggers have been identified to induce programmed cell death pathways, including pyroptosis, apoptosis, and necroptosis^7,11,12,15–28^. Thus, our aim was to perform a biochemical characterization of HSV-1-induced cell death. In WT BMDMs, infection with the HF strain of HSV-1 induced activation of caspase-1 and GSDMD (pyroptotic), caspase-8, -3, and -7 (apoptotic), and MLKL (necroptotic) in an AIM2-dependent manner, whereas infection with the F and KOS strains did not activate these pathways (Fig. 2c–e). As the HF strain of HSV-1 induced activation of multiple cell death molecules, we investigated the relative contribution of each of them using a genetic approach. Despite reports of multiple caspases regulating cell death in different contexts^17,29–31^, immortalized BMDMs (iBMDMs) lacking Casp1, Casp3, Casp6, Casp7, or Casp9 showed similar dynamics of cell death compared with WT iBMDMs (Supplementary Fig. S3a, c, d). Loss of the pyroptotic executioner GSDMD and GSDME and necroptotic executioner MLKL did not protect cells against HF strain infection (Supplementary Fig. S3b, e, f). In contrast, Ripk3-deficient iBMDMs showed reduced cell death compared with WT iBMDMs, and Ripk3/Casp8 double-knockout iBMDMs completely abrogated cell death against HF strain infection (Supplementary Fig. S3b, e, f), suggesting that RIPK3 and caspase-8 are key executioner molecules in this process. Consistent with the engagement of multiple cell death pathways during HSV-1 HF strain infection, we next examined the role of caspase-1 in cytokine production and cell death. As expected, *Casp1*^*−/−*^ BMDMs displayed markedly reduced secretion of IL-1β and IL-18 following infection, whereas substantial cell death persisted, indicating the involvement of caspase-1-independent death pathways (Supplementary Fig. S4a, b). In contrast, loss of MyD88 or TRIF did not affect cell death, indicating that Toll-like receptor signaling is dispensable in this context (Supplementary Fig. S4c, d). Finally, deletion of cGAS, STING, or IRF1 significantly impaired virus-induced cell death, phenocopying AIM2 deficiency, and supporting a model in which the cGAS-STING-IRF1 axis licenses AIM2-dependent inflammasome activation and downstream inflammatory cell death (Supplementary Fig. S4e–g). We also observed interactions between ASC, AIM2, and caspase-1 following HF strain infection (Fig. 2f), indicating a distinct ability of this strain to form the AIM2 inflammasome. Overall, these findings suggest that AIM2 inflammasome activation and coordinated inflammatory cell death programs are crucial for host defense against HSV-1 HF strain infection.

### Strain-dependent contribution of VP22 to inflammasome activation during HSV-1 infection

**Fig. 3:**
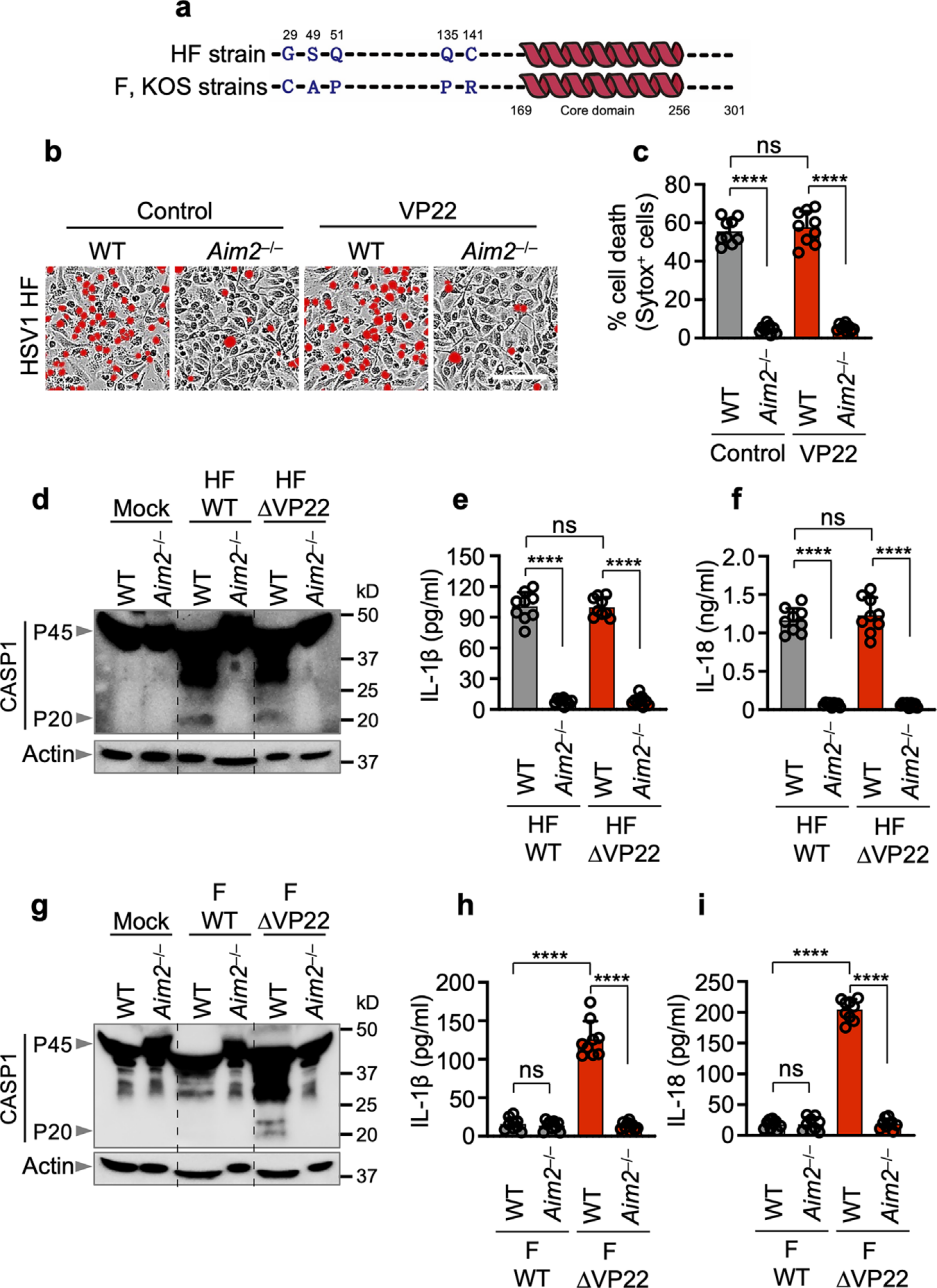
Evaluation of the role of HSV-1 viral protein VP22 in inflammasome activation during HF strain infection. **a** Herpes simplex virus 1 (HSV-1) HF strain exhibits five unique missense mutations within the VP22 protein (C29G, A49S, P51Q, P135Q, and R141C), differentiating it from other HSV-1 strains such as KOS and F. These mutations are designated as natural variants and are located outside the conserved core domain of VP22, which spans amino acids 169–256. **b** Cell death evaluation in control or VP22 plasmid-transfected WT or *Aim2*^−/−^ BMDMs after HSV-1 HF strain infection. Dead cells are indicated in red. Scale bar: 100 μm. Images are representative of three biologically independent experiments. **c** Quantification of cell death in (**b**). **d** Immunoblot analysis of pro-caspase-1 (CASP1; P45) and cleaved CASP1 (P20) in WT or *Aim2*^−/−^ BMDMs after infection with WT HF or mutant HSV-1 strain HF, lacking the VP22 DNA sequence (HF ΔVP22). IL-1β (**e**) and IL-18 (**f**) release assessment in WT or *Aim2*^−/−^ BMDMs following infection of WT HF strain or ΔVP22 HF strain of HSV-1. **g** Immunoblot analysis of pro-caspase-1 (CASP1; P45) and cleaved CASP1 (P20) in wild-type (WT) or *Aim2*^−/−^ BMDMs after infection with WT F strain and ΔVP22 F strain of HSV-1. IL-1β (**h**) and IL-18 (**i**) release assessment in WT or *Aim2*^−/−^ BMDMs following infection with WT F strain and ΔVP22 F strain of HSV-1. **c**, **e**, **f**, **h**, **i** Data as mean ± s.e.m. “ns” not significant; ****P < 0.0001 (one-way ANOVA with Dunnett’s multiple comparisons test; *n* = 9 biologically independent samples from three independent experiments). **d**, **g** Data from three independent experiments. Expected molecular weights: pro-caspase-1 (P45, ~45 kDa) and cleaved caspase-1 (P20, ~20 kDa). Source data are provided as a Source data file.

Previous studies have shown that the HSV-1 F strain viral protein VP22 modulates AIM2 inflammasome activation in BMDMs^13,14^. To assess potential sequence variation among HSV-1 strains, we compared VP22 from the HF strain with that from the reference F and KOS strains (Fig. 3a). This analysis identified five missense substitutions in HF VP22 (C29G, A49S, P51Q, P135Q, and R141C).

Structural annotations indicate that VP22 contains a conserved core domain spanning amino acids 169-254, defined based on sequence conservation and structural analyses across alpha herpesvirus species^32–35^. All identified HF-specific substitutions are located outside this conserved core. However, regions outside the structured core are less well-characterized and may include intrinsically disordered segments that mediate context-dependent interactions with host factors. Accordingly, while VP22 exhibits strain-specific sequence variation, the functional significance of these non-core substitutions, particularly in the context of inflammasome regulation, cannot be inferred from sequence or structural information alone.

To examine the inhibitory effect of AIM2 inflammasome activation by VP22, we transfected VP22 from the F strain into WT and *Aim2*^*−/−*^ BMDMs and subsequently infected them with the HF strain of HSV-1 (Supplementary Fig. S5a). Infection with the HF strain of HSV-1 resulted in the cleavage of caspase-1 (Supplementary Fig. S5b), release of inflammasome-driven cytokines IL-1β (Supplementary Fig. S5c) and IL-18 (Supplementary Fig. S5d), and cell death (Fig. 3b, c) in an AIM2-dependent manner, unaffected by the transfection of VP22 from the F strain. These findings suggest that, unlike the inflammasome inhibitory effect of VP22 in the F strain of HSV-1, VP22 in the HF strain does not play a role in AIM2 inflammasome activation.

To further evaluate the contribution of VP22 to AIM2 inflammasome regulation in the context of viral infection, we next generated VP22-null mutant viruses in both the HF and F strain backgrounds using CRISPR/Cas9-mediated genome editing (Supplementary Fig. S5e, f). Successful deletion of VP22 was confirmed by immunoblot analysis, demonstrating complete loss of VP22 protein expression in the mutant viruses (Supplementary Fig. S5g, h). Consistent with the known role of VP22 in supporting optimal viral fitness, VP22-null viruses exhibited a modest reduction in overall viral protein expression compared with their respective WT counterparts (Supplementary Fig. S5g, h).

We first assessed the impact of VP22 deletion in the HF strain background. Infection of BMDMs with either the WT HF or VP22-null HF virus resulted in comparable levels of caspase-1 cleavage as well as similar secretion of IL-1β and IL-18 (Fig. 3d–f). These results indicate that deletion of VP22 does not alter AIM2 inflammasome activation during HF strain infection, supporting the conclusion that VP22 is dispensable for inflammasome regulation in this context. Together with the results of the VP22 overexpression experiments, these findings suggest that the enhanced AIM2 inflammasome activation observed during HF strain infection is not driven by VP22 and instead points to other HF-specific genomic determinants. In contrast, a distinct outcome was observed in the F strain background. While the WT F virus failed to induce detectable caspase-1 activation or inflammasome-dependent cytokine release, infection with the VP22-null F mutant triggered robust caspase-1 cleavage and secretion of IL-1β and IL-18 (Fig. 3g–i). These findings demonstrate that AIM2 inflammasome activation in the F strain is revealed upon VP22 deletion, supporting its previously reported inhibitory role in inflammasome regulation in HSV-1 F and related strains^13,14^. Collectively, these results indicate a strain-dependent role of VP22 in regulating AIM2 inflammasome activation during HSV-1 infection. While VP22 functions as a negative regulator of inflammasome activation in the F strain, it is dispensable for inflammasome control in the HF strain. Given the highly conserved nature of the VP22 core domain reported in structural studies, these strain-specific differences are unlikely to reflect major intrinsic functional divergence of VP22 itself. Rather, our findings support a model in which viral genomic features, rather than viral proteins, play a dominant role in shaping AIM2-dependent innate immune responses during HF strain infection.

### HSV-1 strain HF containing poly(T) DNA sequences emerges as a crucial ligand for AIM2 inflammasome activation

**Fig. 4:**
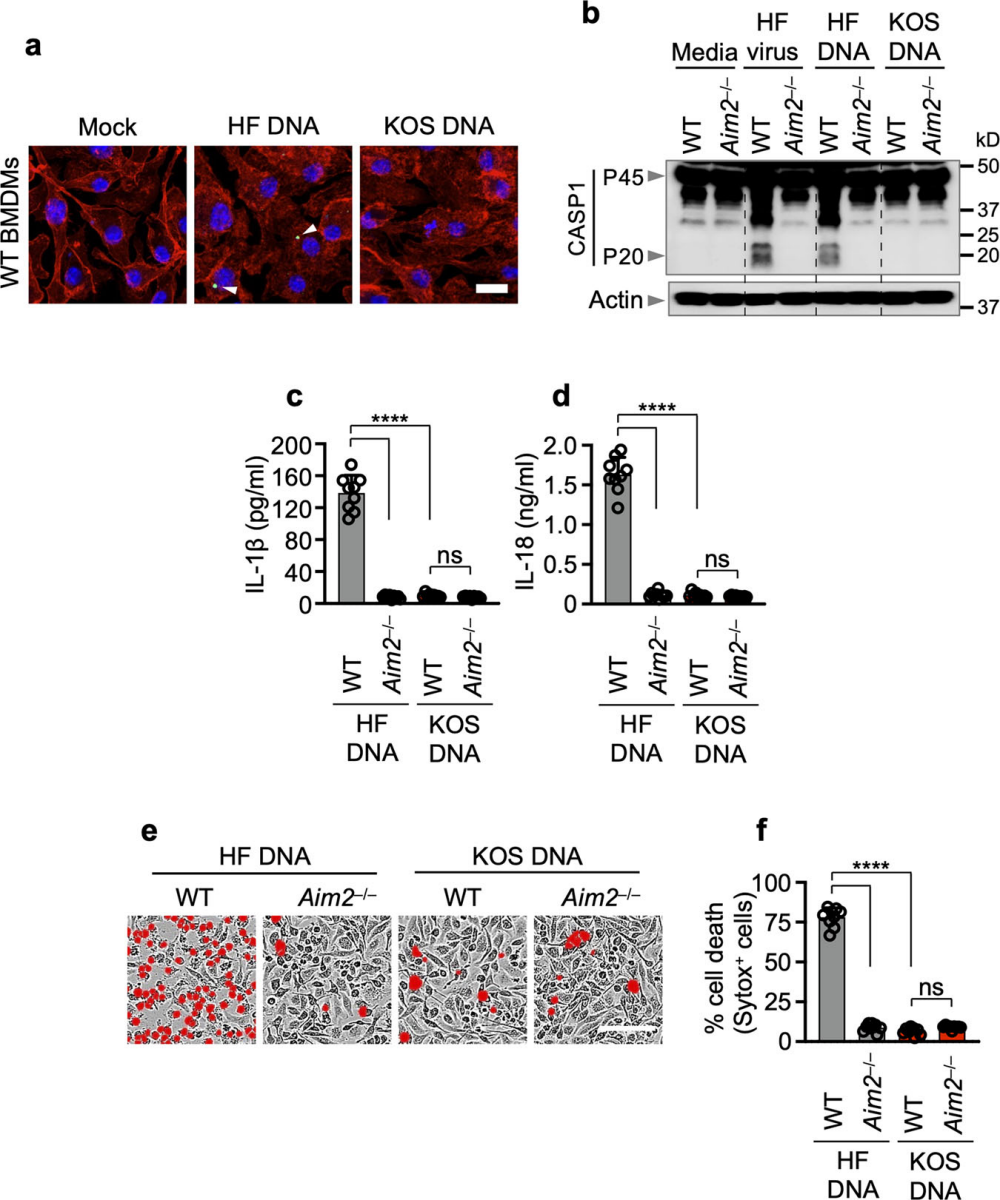
Genomic DNA from HSV-1 strain HF functions as an AIM2 ligand for inflammasome activation and cell death. **a** Immunofluorescence (IF) images of wild-type (WT) bone marrow-derived macrophages (BMDMs) after transfection of genomic DNA of HF and KOS strains of herpes simplex virus 1 (HSV-1). Arrowheads indicate ASC specks. Scale bar: 10 μm. **b** Immunoblot analysis of pro-caspase-1 (CASP1; P45) and cleaved CASP1 (P20) in WT or *Aim2*^−/−^ BMDMs after infection with HF strain of HSV-1 or transfection of genomic DNA of HF and KOS strains. Expected molecular weights: pro-caspase-1 (P45, ~45 kDa) and cleaved caspase-1 (P20, ~20 kDa). **c, d** IL-1β (**c**) and IL-18 (**d**) release assessment in WT or *Aim2*^−/−^ BMDMs following transfection of genomic DNA of HF and KOS strains. **e** Cell death evaluation in WT or *Aim2*^−/−^ BMDMs after transfection of genomic DNA of HF and KOS strains. Dead cells are indicated in red. Scale bar: 100 μm. **f** Quantification of cell death in (**e**). **a**, **b**, **e** Data from three independent experiments. **c**, **d**, **f** Data as mean ± s.e.m. “ns” not significant; ****P < 0.0001 (one-way ANOVA with Dunnett’s multiple comparisons test; *n* = 9 biologically independent samples from three independent experiments). Source data are provided as a Source data file.

Given AIM2’s known affinity for dsDNA and its role in forming an inflammasome against DNA viruses^6–12^, we investigated the impact of viral DNA as a ligand for AIM2-mediated inflammasome activation. Genomic DNA from HSV-1 strain HF induced ASC speck formation, caspase-1 cleavage, release of inflammasome-driven cytokines IL-1β and IL-18, and cell death in an AIM2-dependent manner, whereas genomic DNA from strain KOS did not (Fig. 4a–f). This suggests that genomic DNA from HSV-1 strain HF functions as a ligand for AIM2 inflammasome activation.

**Fig. 5:**
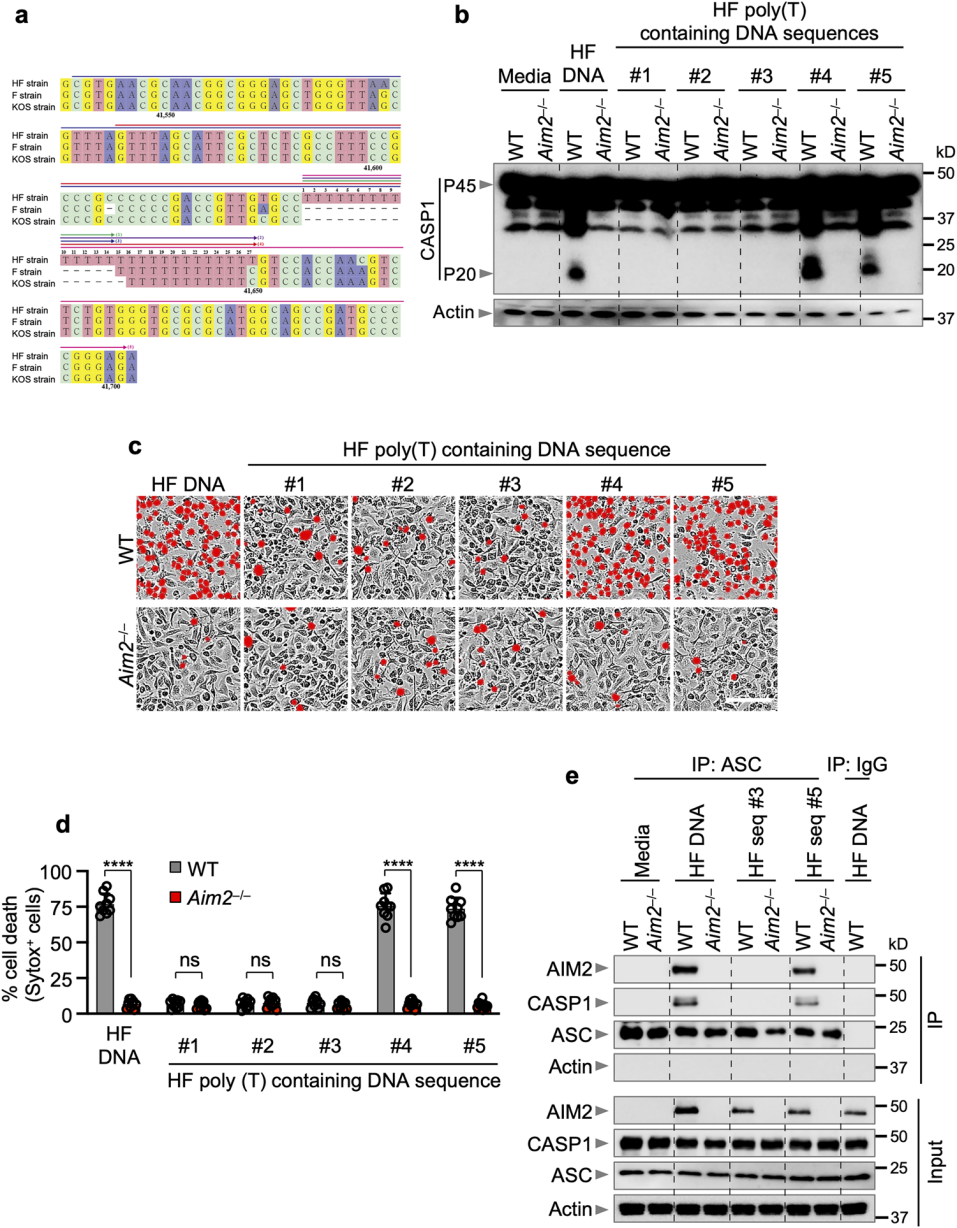
HF strain of HSV-1 containing poly(T) DNA sequences emerges as a crucial ligand for AIM2 inflammasome activation. **a** Mapping of primers on herpes simplex virus (HSV) genomic sequence of designing specific dsDNA to target unique regions HSV-1 HF (5′-TTTTTTTTTTTTTT-3′) from F and KOS DNA sequence. Indicated dsDNA (#1: 14 mer, #2: 27 mer, #3: 97 mer, #4: 89 mer, and #5: 80 mer) are designed. **b** Immunoblot analysis of pro-caspase-1 (CASP1; P45) and cleaved CASP1 (P20) in wild-type (WT) or *Aim2*^−/−^ bone marrow-derived macrophages (BMDMs) after transfection of indicated dsDNA. Expected molecular weights: pro-caspase-1 (P45, ~45 kDa) and cleaved caspase-1 (P20, ~20 kDa). Data are representative of three independent experiments. **c** Cell death evaluation in WT or *Aim2*^−/−^ BMDMs after transfection of indicated dsDNA. Dead cells are indicated in red. Scale bar: 100 μm. Images are representative of three biologically independent experiments. **d** Quantification of cell death in (**c**). Data are mean ± s.e.m. “ns” not significant; ****P < 0.0001 (two-way ANOVA with Šídák’s multiple comparisons test; *n* = 9 biologically independent samples from three independent experiments). **e** Immunoprecipitation (IP) in WT or *Aim2*^−/−^ BMDMs with IgG control antibodies or anti-ASC antibodies after transfection of indicated dsDNA. Data are representative of three independent experiments. Source data are provided as a Source data file.

To identify specific DNA sequences serving as ligands for AIM2 inflammasome activation, we designed primers targeting unique regions within the HSV-1 HF, F, and KOS sequences. Two unique DNA sequences in the non-coding region of HSV-1, including 5′-TTTTTTTTTTTTTTTTTTTTTTTTTTT-3′ (DQ889502.1) and 5′-CCCTGGGAGATGGGCCCACCGT-3′ (DQ889502.1) that were exclusive to the HF strain were identified (Fig. 5a, Supplementary Fig. S6a). Among these, only 27-mer poly(T) containing viral dsDNA sequences (#4 and #5) induced caspase-1 cleavage and cell death in an AIM2-dependent manner (Fig. 5b–d, Supplementary Fig. S6b–d).

To further define the DNA sequence features driving AIM2 activation, we next examined whether the observed HF-specific response could be attributed to general DNA composition rather than sequence length or context. Consistent with previous reports showing that AIM2 can be activated by various dsDNA ligands, including poly(dG:dC), in a GC-content-independent manner^10,36^, transfection of poly(dG:dC) duplexes into BMDMs induced caspase-1 cleavage and IL-1β secretion (Supplementary Fig. S6e, f). However, comparative analysis of HSV-1 genomes revealed that poly(G:C) tracts of comparable length (≤16 nucleotides) were present across HF, F, and KOS strains, with no selective enrichment in the HF genome. Moreover, longer poly(G:C) stretches were absent in all of the examined HSV-1 variants, making them unlikely candidates for mediating HF-specific AIM2 inflammasome activation. In contrast, genomic analysis identified a uniquely long poly(A:T) stretch, a 27-mer poly(T) sequence, present in the HF strain but absent or substantially shorter in the F and KOS strains. This observation prompted us to directly assess the length dependency of poly(T)-mediated AIM2 activation. Therefore, we synthesized double-stranded poly(T) DNA of defined lengths (10, 30, and 90 nucleotides) and transfected them into BMDMs. Robust caspase-1 cleavage and IL-1β release were observed exclusively in response to the 90-mer poly(T), while shorter poly(T) fragments failed to induce significant inflammasome activation (Supplementary Fig. S6g, h). These findings align with prior studies demonstrating that efficient AIM2 inflammasome assembly requires dsDNA exceeding a critical length threshold.

Consistent with the ability of long poly(T) DNA to promote AIM2 inflammasome activation^10^, we next examined whether poly(T)-containing DNA facilitates assembly of the AIM2 inflammasome complex. Immunoprecipitation analysis revealed an enhanced association between ASC, AIM2, and caspase-1 following stimulation with HF strain genomic DNA or the 27-mer poly(T)-containing viral dsDNA sequence (#5) (Fig. 5e). These interactions were not observed under conditions in which shorter poly(T) DNA failed to activate the inflammasome. Together, these data indicate that the presence of poly(T)-rich viral DNA in the HF strain promotes physical assembly of the AIM2 inflammasome, providing a mechanistic basis for strain-specific inflammasome activation.

### Poly(T) DNA length determines strain-specific AIM2 inflammasome activation and host defense during HSV-1 infection

**Fig. 6:**
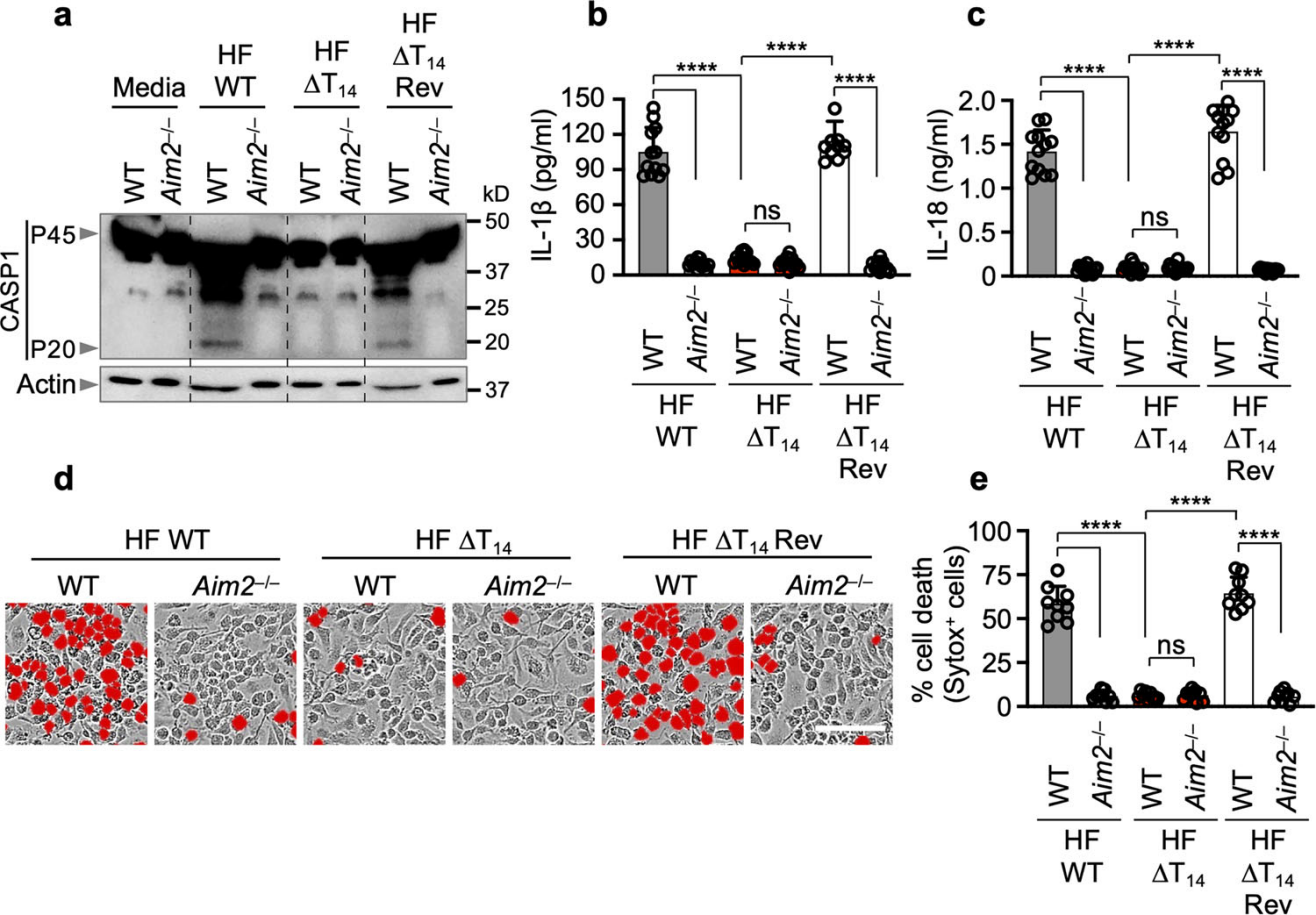
The 14-mer poly(T) DNA sequence is required for AIM2 inflammasome activation and inflammatory cell death in vitro. **a** Immunoblot analysis of pro-caspase-1 (CASP1; P45) and cleaved CASP1 (P20) in wild-type (WT) or *Aim2*^−/−^ bone marrow-derived macrophages (BMDMs) after infection with WT HF, mutant herpes simplex virus 1 (HSV-1) strain HF, lacking the 14-mer poly(T) DNA sequence (HF ΔT_14_), or the revertant virus (HF ΔT_14_-Rev), in which the deleted 14-bp poly(T) motif is reintroduced via homologous recombination. Expected molecular weights: pro-caspase-1 (P45, ~45 kDa) and cleaved caspase-1 (P20, ~20 kDa). **b, c** IL-1β (**b**) and IL-18 (**c**) release assessment in WT or *Aim2*^−/−^ BMDMs following WT, HF ΔT_14_, or HF ΔT_14_-Rev infection. **d** Cell death evaluation in WT or *Aim2*^−/−^ BMDMs after infection with WT, HF ΔT_14_, or HF ΔT_14_-Rev. Dead cells are indicated in red. Scale bar: 100 μm. **e** Quantification of cell death in (**d**). **a**, **d** Data from three independent experiments. **b**, **c**, **e** Data as mean ± s.e.m. “ns” not significant; ****P < 0.0001 (one-way ANOVA with Dunnett’s multiple comparisons test; *n* = 12 biologically independent samples for (**b**, **c**), and *n* = 9 biologically independent samples for (**e**), each derived from three independent experiments). Source data are provided as a Source data file.

To directly assess the role of HF-specific poly(T) DNA in inflammasome activation, we generated a panel of recombinant HSV-1 HF viruses, including the WT HF strain (WT HF), a mutant lacking the 14-mer poly(T) sequence in the UL25-UL26 intergenic region (HF ΔT_14_), and a revertant virus restoring the deleted poly(T) sequence (HF ΔT_14_-Rev) (Supplementary Fig. S7a, b). All three viruses exhibited comparable expression of representative viral proteins and showed no differences in UL25 or UL26 transcript levels, indicating preserved viral gene expression and intact neighboring loci (Supplementary Fig. S1c; Supplementary Fig. S7c, e). In primary BMDMs, infection with WT HF or HF ΔT_14_-Rev robustly induced caspase-1 cleavage, secretion of IL-1β and IL-18, and inflammatory cell death, whereas HF ΔT_14_ infection failed to elicit these responses (Fig. 6a-e). Notably, all inflammasome-associated responses were completely abolished in *Aim2*^*−/−*^ BMDMs (Fig. 6a–e), confirming that poly(T)-mediated inflammasome activation by the HF strain is strictly AIM2-dependent.

**Fig. 7:**
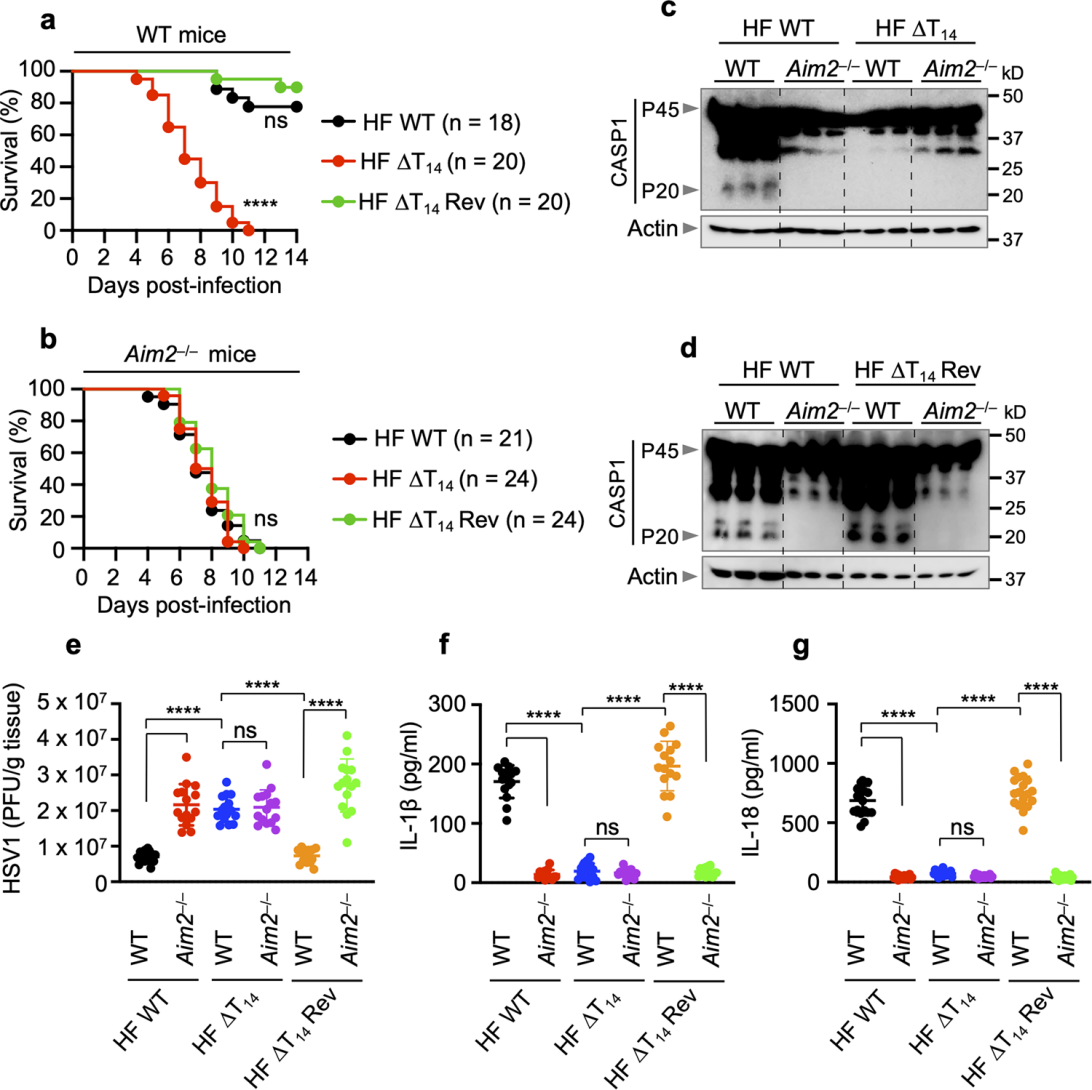
Viral poly(T) DNA is essential for AIM2-dependent host protection during HSV-1 infection in vivo. **a, b** Survival of WT (**a**) or *Aim2*^−/−^ mice (**b**) infected intranasally with 5 × 10^5^ plaque-forming units (PFU) with WT HF, mutant herpes simplex virus 1 (HSV-1) strain HF, lacking the 14-mer poly(T) DNA sequence (HF ΔT_14_), or the revertant virus (HF ΔT_14_-Rev), in which the deleted 14-bp poly(T) motif is reintroduced via homologous recombination. “ns” not significant (WT HF versus HF ΔT_14_-Rev in WT mice, WT HF versus HF ΔT_14_ or HF ΔT_14_-Rev in *Aim2*^−/−^ mice); ****P < 0.0001 (WT HF versus HF ΔT_14_ in WT mice), log-rank test (Mantel–Cox). Survival data are pooled from three independent experiments. **c, d** Immunoblot analysis of pro- (P45) and activated (P20) caspase-1 (CASP1) in lung tissue from WT or *Aim2*^−/−^ mice 5 days after infection with WT HF and HF ΔT_14_ HSV-1 (**c**), or WT HF and HF ΔT_14_-Rev HSV-1 (**d**). Expected molecular weights: pro-caspase-1 (P45, ~45 kDa) and cleaved caspase-1 (P20, ~20 kDa). Each lane indicates independent biological replicates. Data are representative of three independent experiments. **e** Pulmonary viral titer at day 5 after infection with WT HF, HF ΔT_14_, or HF ΔT_14_-Rev HSV-1. **f, g** IL-1β (**f**) and IL-18 (**g**) release assessment in bronchoalveolar lavage fluid (BALF) of the WT or *Aim2*^−/−^ mice following WT HF, HF ΔT_14_, or HF ΔT_14_-Rev HSV-1 infection. **e**–**g** Data as mean ± s.e.m. ****P < 0.0001 (one-way ANOVA with Dunnett’s multiple comparisons test; each symbol represents one mouse). Data are pooled from three independent experiments. Source data are provided as a Source data file.

We next examined the impact of poly(T) deletion in vivo. WT mice infected with HF ΔT_14_ rapidly succumbed to infection, whereas most WT HF-infected mice survived (Fig. 7a). Consistent with this, viral titers were elevated, and caspase-1 activation and IL-1β and IL-18 production in the lungs were abolished in HF ΔT_14_-infected WT mice, phenocopying infection in *Aim2*^*−/−*^ animals (Fig. 7b–g). Restoration of the poly(T) sequence in HF ΔT_14_-Rev rescued survival, viral control, and inflammasome activation to levels indistinguishable from WT HF infection (Fig. 7a–g). In *Aim2*^*−/−*^ mice, all three viruses caused uniformly lethal infection with no detectable inflammasome activation, demonstrating that the protective effect of the poly(T) motif is entirely AIM2-dependent in vivo.

To determine whether poly(T) DNA is sufficient to trigger AIM2 inflammasome activation, we generated a recombinant HSV-1 F strain harboring a 27-mer poly(T) insertion in the UL25-UL26 intergenic region (F T_27_), along with a corresponding revertant virus (F T_27_-Rev) (Supplementary Fig. S7b). In primary BMDMs, infection with F T_27_, but not WT F or F T_27_-Rev, induced robust caspase-1 cleavage in an AIM2-dependent manner (Supplementary Fig. S8a). In vivo, WT mice infected with F T_27_ exhibited significantly improved survival and enhanced caspase-1 activation compared with those infected with WT F or F T_27_-Rev, whereas these responses were completely abolished in *Aim2*^*−/−*^ mice (Supplementary Fig. S8b–e). Viral protein expression and neighboring gene transcription were comparable across all F strain variants, ruling out differences in viral replication or fitness as confounding factors (Supplementary Fig. S1d; Supplementary Fig. S7d, f).

Given the human specificity of HSV-1, we extended these findings to a human macrophage model. In PMA-differentiated THP-1 cells, siRNA-mediated knockdown of AIM2 abolished inflammatory cell death induced by WT HF, HF ΔT_14_-Rev, and F T_27_, while leaving responses to HF ΔT_14_, WT F, and F T_27_-Rev unchanged (Supplementary Fig. S9a–f). Across all viral backgrounds and genetic manipulations, the inflammasome phenotypes observed in murine BMDMs were faithfully recapitulated in human macrophages, demonstrating conservation of poly(T)-mediated AIM2 activation across species.

Finally, to determine whether long poly(T) motifs represent viral genomic features present in both clinical and laboratory isolates rather than solely culture-derived artifacts, we comprehensively analyzed publicly available viral genome datasets. Among 492 complete HSV-1 genomes, both 14-mer and 23-mer poly(T) sequences were detected in multiple clinical isolates and laboratory strains, with the 14-mer present in approximately 15% and the 23-mer in 3% of clinical samples (Supplementary Fig. S10a, b). Moreover, long poly(T) tracts were identified across a broad range of DNA and RNA viruses, including hepacivirus, monkeypox virus, SARS-CoV, adenovirus, and herpes viruses (Supplementary Data 1 and 2), indicating that these motifs are consistent with naturally occurring variation and appear to be evolutionarily conserved and widespread.

Collectively, these loss-of-function, gain-of-function, rescue, and cross-species analyses provide strong genetic and functional evidence that poly(T) DNA length is a key determinant of strain-specific AIM2 inflammasome activation. Our findings establish viral poly(T) DNA as genome-encoded and functionally relevant innate immune ligand that drives inflammatory cell death and antiviral host defense during HSV-1 infection.

## Discussion

Strain-dependent differences in AIM2 inflammasome activation during HSV-1 infection have long been recognized; however, the underlying viral determinants responsible for this heterogeneity have remained unclear. In this study, we demonstrated that these differences are primarily driven by distinct viral genomic elements rather than by intrinsic differences in viral protein function. Systematic comparisons of HSV-1 HF, F, and KOS strains demonstrated that robust AIM2 inflammasome activation is a unique feature of the HF strain, while F and KOS strains elicit minimal activation despite comparable infection efficiency and viral protein expression. These findings establish viral genomic composition as a critical factor shaping AIM2-dependent innate immune responses.

Although previous studies have identified the viral tegument protein VP22 as an inhibitor of AIM2 inflammasome activation in certain HSV-1 strains^13,14^, our expanded genetic and functional analyses reveal a more nuanced, strain-dependent role. In the F strain background, deletion of VP22 restores AIM2 inflammasome activation, consistent with earlier reports^13,14^. In contrast, VP22 deletion or overexpression in the HF background does not alter inflammasome activation, indicating that VP22 is dispensable in the presence of a dominant genomic ligand. These results support a model in which VP22-mediated immune evasion operates in some HSV-1 strains but is overridden by potent viral DNA-based inflammasome ligands in others.

By focusing on viral DNA as a potential AIM2 ligand, we identified unique poly(T)-containing sequences within the UL25-UL26 non-coding intergenic region that are enriched in the HF strain. Functional dissection, including viral DNA transfection, targeted deletion (HF ΔT_14_), genetic reversion (HF ΔT_14_-Rev), and gain-of-function insertion into the F strain (F-T_27_), demonstrates that these poly(T) sequences are both necessary and sufficient to drive AIM2-dependent inflammasome activation in vitro and in vivo. Importantly, deletion of the poly(T) motif abolishes inflammasome activation and host protection without affecting viral replication, neighboring gene expression, or global viral protein production, ruling out indirect effects from genome editing.

A key conceptual advance of this study is reconciling our findings with prior biochemical studies showing that AIM2 hematopoietic interferon-inducible nuclear-domain binding is largely sequence- and GC-content-independent. While synthetic ligands such as poly(dG:dC) can robustly activate AIM2 when delivered directly into the cytosol, our comparative genomic analyses reveal that such GC-rich tracts are short (≤16 bp) and evenly distributed across HSV-1 strains, meaning that they are unlikely to account for strain-specific inflammasome activation. In contrast, we identified long poly(A:T), particularly poly(T), stretches present in HSV-1 genomes and selectively enriched in the HF strain. Length-dependence experiments further demonstrated that only long dsDNA containing poly(T) tracts of sufficient length (≥80 bp total dsDNA) can efficiently trigger AIM2 activation, consistent with structural models requiring higher-order DNA scaffolds for AIM2 oligomerization and inflammasome assembly^10^. Although the poly(T) tract itself is relatively short (27-mer), it is embedded within the much longer HSV-1 genomic DNA, which provides an extended dsDNA platform capable of supporting AIM2 filament formation and inflammasome assembly. Thus, the poly(T) motif likely functions as a sequence determinant within a longer genomic DNA context rather than as an isolated ligand, enabling efficient AIM2 activation despite the local motif length. Thus, while AIM2 is capable of sensing diverse DNA ligands in principle, our data highlight how genome-encoded sequence length polymorphisms confer physiological specificity during viral infection.

Beyond inflammasome activation, our findings clarify the understanding of downstream cell death pathways engaged during HSV-1 infection. Although AIM2-dependent caspase-1 activity is essential for IL-1β and IL-18 maturation, inflammatory cell death still occurs in the absence of caspase-1 or gasdermins, implicating alternative execution mechanisms. Consistent with our previous work on PANoptosis^11^, we showed that RIPK3 and caspase-8 are required for full execution of AIM2-driven inflammatory cell death, supporting the formation of a multiprotein PANoptosome complex during HSV-1 infection. Importantly, genetic ablation of MYD88 or TRIF does not attenuate these responses, ruling out any major contribution from TLR signaling pathways.

Mechanistically, we further demonstrated that efficient AIM2-dependent responses require upstream licensing via the cGAS-STING-IRF1 axis. Genetic ablation of cGAS, STING, or IRF1 markedly impaired AIM2-dependent inflammasome activation and inflammatory cell death during HSV-1 infection, indicating that this signaling axis is required to enable effective AIM2-mediated responses. This layered sensing architecture suggests that AIM2 inflammasome activation is contingent on prior cytosolic DNA sensing pathways, thereby providing an additional level of regulation that restricts inflammasome activation to contexts of bona fide viral infection and limits inappropriate inflammatory responses under homeostatic conditions.

Importantly, the relevance of poly(T)-mediated AIM2 activation extends beyond murine systems. Using human THP-1 macrophages, we confirmed that poly(T)-dependent inflammasome activation and inflammatory cell death are conserved in human innate immune cells and strictly AIM2-dependent. Furthermore, our large-scale analysis of public viral genome datasets revealed that long poly(T) tracts are not restricted to laboratory-adapted HSV-1 strains but are present in multiple clinical HSV-1 isolates and across diverse DNA and RNA viruses, including poxviruses, herpesviruses, and coronaviruses. These observations suggest that poly(T) motifs represent a broader class of innate immune, relevant genomic features with potential implications across viral pathogenesis.

From a translational perspective, the identification of viral poly(T) DNA as a dominant AIM2 ligand raises the possibility that selective modulation of DNA-inflammasome interactions could represent a novel therapeutic avenue to be explored. For example, targeting poly(T)-rich genomic regions or downstream AIM2-dependent signaling pathways may represent a strategy to dampen excessive inflammasome activation and immunopathology during severe HSV-1 infection, while preserving antiviral host defense. Conversely, enhancing AIM2 responsiveness to such DNA motifs may offer a strategy to strengthen protective immunity in immunocompromised settings.

Future studies are required to determine how poly(T) length polymorphisms influence disease severity in patients and whether similar DNA motifs contribute to inflammasome activation in other viral or inflammatory disorders. Systematic profiling of poly(T)-rich genomic regions across viral genomes, coupled with clinical outcome data, may further elucidate how viral genomic variation shapes innate immune responses in vivo.

Collectively, our study establishes viral poly(T) DNA as a physiologically relevant ligand that governs strain-specific AIM2 inflammasome activation during HSV-1 infection. By revealing how genome-encoded sequence variation shapes innate immune sensing, these findings advance our fundamental understanding of host-virus interactions and highlight poly(T) DNA as a potential target for therapeutic modulation of inflammasome responses. More broadly, this work provides a conceptual framework for exploring how subtle viral genome polymorphisms can decisively influence immune outcomes, extending beyond HSV-1 to other viral and inflammatory diseases.

## Methods

### Mice

C57BL/6J mice (WT) were obtained from Hyochang Science, while *Aim2*^−/−^ (Jackson Laboratory), *Nlrp3*^−/−^ (Cyagen), *Nlrc4*^−/−^ (Cyagen), *Casp1*^−/−^ (Jackson Laboratory) mice, all on a C57BL/6J genetic background, were purchased from the indicated sources^7,12^. All mice used in this study were of the species Mus musculus. The mice were group-housed, with up to five mice per cage, and were bred under standard pathogen-free conditions in the animal facility at the Ulsan National Institute of Science and Technology (UNIST). The mice were maintained on a 12-h light/dark cycle (lights on from 7 AM to 7 PM) at a controlled ambient temperature (22 ± 2 °C) and relative humidity (50 ± 10%), and were provided with standard chow. Both male and female mice were included in this study. In vivo investigations utilized age- and sex-matched mice aged 6–8 weeks, while in vitro studies involved mice aged 6–12 weeks. Co-housed animals were chosen for in vivo analyses. All experimental procedures were executed following protocols approved by the Institutional Animal Care and Utilization Committee of UNIST [UNISTIACUC-23-22].

### Cell culture

Primary bone marrow-derived macrophages (BMDMs) were cultured for 6 days in Iscove’s Modified Dulbecco’s Medium (IMDM, Thermo Fisher Scientific, Cat. No. 12440061), supplemented with 10% fetal bovine serum (FBS, Thermo Fisher Scientific, Cat. No. 16000044), 30% L929-conditioned medium, 1% non-essential amino acids (Thermo Fisher Scientific, Cat. No. 11140-050), and 1% penicillin-streptomycin solution (Thermo Fisher Scientific, Cat. No. 15070-063). These BMDMs were seeded in 12-well plates at a density of 1 million cells/well and incubated overnight before further use. L929 cells (ATCC, CCL-1) were procured and cultured in IMDM supplemented with 10% FBS (Thermo Fisher Scientific, Cat. No. 16000044), 1% non-essential amino acids, and 1% penicillin-streptomycin. Immortalized bone marrow-derived macrophages (iBMDMs) were cultivated in Dulbecco’s Modified Eagle Medium (DMEM, Thermo Fisher Scientific, Cat. No. 11995081) containing 10% FBS and 1% penicillin-streptomycin. THP-1 cells were kindly provided by Atsushi Kawaguchi (University of Tsukuba), and cells were grown in RPMI 1640 (Thermo Fisher Scientific, Cat. No. 11875119) with 10% FBS and differentiated into macrophages in RPMI 1640 medium containing 20% FBS and 100 ng/ml PMA (SIGMA, Cat. No. P1585-5MG) for 2 days. Vero cells (provided by Atsushi Kawaguchi (University of Tsukuba)), 293FT cells (Thermo Fisher Scientific, Cat. No. R70007), and HaCaT cells (Cytion, Cat. No. 300493) were cultured in Dulbecco’s Modified Eagle Medium (DMEM, Thermo Fisher Scientific, Cat. No. 11995081) supplemented with 10% fetal bovine serum (FBS, Thermo Fisher Scientific, Cat. No. 16000044) and 1% penicillin-streptomycin (Thermo Fisher Scientific, Cat. No. 15070-063). Cells were maintained at 37 °C in a humidified incubator with 5% CO₂ and routinely passaged to maintain exponential growth. All cell lines were tested and confirmed to be free of mycoplasma contamination.

### Generation of knockout clonal cells

WT and *Gsdmd*^*−/−*^ iBMDMs were kindly provided by Professor Tae-Hyuk Kwon (UNIST). In addition, *Casp1*^−/−^, *Casp3*^−/−^, *Casp6*^−/−^, *Casp7*^−/−^, *Casp9*^−/−^, *Gsdme*^−/−^, *Mlkl*^−/−^, *Ripk3*^−/−^, *Ripk3*^−/−^*Casp8*^−/−^, *Aim2*^−/−^, and *Irf1*^−/−^ iBMDMs had been generated previously, as described earlier (Supplementary Data 3)^7,12^. For the present study, *Sting*^*−/−*^, *cGas*^*−/−*^, *Myd88*^*−/−*^, and *Trif*^*−/−*^ iBMDMs were newly generated using a CRISPR/Cas9-mediated genome-editing approach (Supplementary Data 3). The WT iBMDM cells underwent electroporation with 1 µg of Cas9-encoding plasmid (Addgene, #43945) and 1 µg of gRNA-expressing plasmid (Addgene, #104174), following the Amaxa 4D-Nucleofector program DS-136. For electroporation, a total of 4 × 10^5^ cells were employed. To create single-cell-derived knockout clones in iBMDMs, the transfected cells were seeded at a low density (0.5 cells/well) in a 96-well plate. After a period of 10–14 days, each well was scrutinized for the presence of viable single-cell-derived clones. These clones were detached using trypsin, and genomic DNA extraction was performed using the DNeasy Blood & Tissue Kit (Qiagen). For confirmation of the knockout of the target gene in the single-cell-derived clones, genomic regions encompassing the Cas9 target site were subjected to amplification using KAPA HiFi HotStart PCR polymerase (KK2502). The ensuing amplicons were further amplified using TruSeq HT dual-index-containing primers to produce libraries for deep sequencing. The sequencing of these libraries was accomplished using an Illumina iSeq equipped with a paired-end sequencing system. The calculation of mutation frequencies was performed using the MAUND program, accessible at https://github.com/ibs-cge/maund.

### Gene silencing mediated by siRNA

siRNA against the AIM2 gene was purchased from Life Technologies. The nucleotide sequences of the siRNAs used were as follows: 5′-GACAUCUGGAGUUCAUAGCACCAUA-3′ and 5′-UAUGGUGCUAUGAACUCCAGAUGUC-3′. For transfection, 15 pmol of siRNA was incubated with 500 μl of Opti-MEM and 7.5 μl of Lipofectamine RNAi Max (Life Technologies) for 15 min at room temperature. The mixture was transferred into 12-well plates at a density of 10^5^ cells/well for THP-1 macrophages. As a negative control, non-specific scrambled siRNA was used.

### Generation of recombinant HSV-1 viruses

Recombinant HSV-1 viruses were generated using a two-step CRISPR/Cas9-mediated homologous recombination strategy^37^. To construct the HF ΔT_14_ mutant, in which 14 thymidines were deleted from the 27T stretch located between the UL25 and UL26 genes of the HF strain, 293FT cells were co-transfected with a CRISPR/Cas9 plasmid expressing a guide RNA (gRNA#1) targeting the UL25-UL26 intergenic region and a donor plasmid carrying a CMV promoter-driven eGFP cassette flanked by homology arm sequences (HAS). To maintain expression of the essential UL26 gene, an internal ribosome entry site (IRES) sequence was inserted between the eGFP cassette and the UL26 start codon. Transfection was performed using jetOPTIMUS reagent (Polyplus, 101000006), followed by treatment with 1 μM SCR7 (Sigma, SML1546) to inhibit non-homologous end joining and 1 μg/mL puromycin (InvivoGen, ANT-PR-1) for selection. After 24 h, cells were infected with WT HSV-1 HF at a multiplicity of infection (MOI) of 0.5. Viral progenies were released by three freeze-thaw cycles, serially diluted, and used to infect Vero cells for plaque purification. eGFP-positive plaques were identified using fluorescence microscopy, isolated, and propagated to generate an intermediate recombinant virus containing an eGFP insertion between UL25 and UL26. The final HF ΔT_14_ mutant was obtained by replacing the eGFP cassette with a donor template carrying the ΔT_14_ sequence through a second round of CRISPR/Cas9 recombination using gRNA#2 targeting the CMV promoter. Recombinant viruses were plaque-purified and verified by PCR and Sanger sequencing. CRISPR/Cas9 plasmids were constructed by inserting synthesized oligonucleotides into pSpCas9(BB)-2A-Puro V2.0 (Addgene #62988), and donor templates containing either the eGFP cassette or ΔT_14_ sequence flanked by homology arm sequences were assembled using the AccuRapid Cloning Kit (Bioneer, K−7130). Additional recombinant viruses, including HF ΔT_14_-Rev (revertant), F T_27_, and F T_27_-Rev, were generated using the same procedure with strain-specific gRNAs and donor constructs specific to each strain. HSV-1 VP22 knockout viruses (ΔVP22) were produced by the same strategy. Briefly, 293FT cells were co-transfected with a CRISPR/Cas9 plasmid expressing a gRNA targeting the VP22 coding region and a donor plasmid containing a CMV promoter-driven eGFP cassette flanked by homology arms corresponding to sequences upstream and downstream of VP22. eGFP-positive plaques were isolated using fluorescence microscopy and propagated in Vero cells. Deletion of VP22 and insertion of the eGFP cassette were confirmed using PCR and immunoblotting with anti-VP22 antibodies, and viral genome integrity was verified by Sanger sequencing.

### Virus culture, infection, and cell stimulations

Human HSV-1 HF strain (ATCC; VR-260), HSV-1 F strain (ATCC; VR-733), and HSV-1 KOS strain (ATCC; VR-1493) were purchased and propagated in Vero cells (gift from Dr. Atsushi Kawaguchi, University of Tsukuba); the virus titer was measured using the plaque assay in Vero cells. The HF ΔT_14_, HF ΔT_14_-Rev, F T_27_, F T_27_-Rev, and ΔVP22 mutant viruses were also propagated in Vero cells. For HSV-1 (16 h; MOI 10) infection, primary BMDMs and iBMDMs were cultured in DMEM high glucose (Gibco, 11995-065). For NLRP3 inflammasome activation, cells were primed for 4 h with 500 ng/mL ultrapure LPS from *Escherichia coli* (0111:B4) (InvivoGen, tlrl-3pelps) and then stimulated for 6 h with 1 μM nigericin (InvivoGen, tlrl-nig). For ligand-mediated AIM2 inflammasome activation, 1 ng of poly(dA:dT) (InvivoGen, tlrl-patn), poly(dG:dC) (InvivoGen, tlrl-pgcn), or poly(T) DNA of 10-, 30-, or 90-mer length was transfected into cells using DOTAP (Roche, 11202375001) according to the manufacturer’s instructions, followed by incubation for 6 h. For NLRC4 inflammasome activation, 10 ng of flagellin (Invivogen, tlrl-epstfla-5) was transfected into cells using DOTAP (Roche, 11202375001) according to the manufacturer’s instructions, followed by incubation for 6 h. In the context of VP22, F strain VP22 vector (pLV[Exp]-EGFP:T2A:Puro-EF1A > VP22) was purchased (Vector Builder, VB220920-1383rfw), and BMDMs were transfected with the VP22 expression plasmid 12 h prior to viral infection using DOTAP (Roche, 11202375001). Cells were then infected with HSV-1 and harvested 16 h post-incubation. Genomic DNA from HSV-1 strain HF (ATCC, VR-260D) and genomic DNA from HSV-1 strain KOS (ATCC, VR-1493DQ) were purchased, and we transfected 1 ng of genomic DNA using DOTAP (Roche, 11202375001), following the manufacturer’s instructions. Cells were harvested at 16 h post-infection for immunoblotting, cytokine analysis, ASC speck, and cell death quantification. For HSV-1 ligand screening, we transfected 1 ng of indicated dsDNA using DOTAP (Roche, 11202375001), using the following dsDNA sets: 5′-TTTTTTTTTTTTTT-3′, 5′-TTTTTTTTTTTTTTTTTTTTTTTTTTT-3′, 5′-CGTGAACGCAACGGCGGGAGCTGGGTTAACGTTTAGTTTAGCATTCGCTCTCGCCTTTCCGCCCGCCCCCCGACCGTTGTGCCTTTTTTTTTTTTTT-3′, 5′-GTTTAGTTTAGCATTCGCTCTCGCCTTTCCGCCCGCCCCCCGACCGTTGTGCCTTTTTTTTTTTTTTTTTTTTTTTTTTTGTCCACCAA-3′, 5′- GCCTTTTTTTTTTTTTTTTTTTTTTTTTTTGTCCACCAACGTCTCTGTGGGTGCGCGCATGGCAGCCGATGCCCCGGGAG-3′, 5′-CCCTGGGAGATGGGCCCACCGT-3′, 5′-CGTAACCATACCCAAACCGACTCTGGGGGTTGTTTGTGGGGTCGGGACTATAGGATAAACAAACAGACCACCCCCGTACCTCCCCCTGGGAGATGGGCCCACCGTCCCAC-3′, and 5′-CCCCGTACCTCCCCCTGGGAGATGGGCCCACCGTCCCACCCAAGGGTGCGGGTGGCTCATCGGCATCTGTGCGGTATTGGTTGTTACCCGCCCACTCGCGTTCGGACGT-3′.

### Cell death imaging and analysis

We employed an IncuCyte S3 imaging system (Sartorius). Cell death analysis was executed following established methodologies^7,11,12,23,28,38–40^, utilizing the same IncuCyte S3 imaging system. The primary BMDMs, iBMDMs, or THP-1 were seeded into 12-well plates at a density of 10^6^ cells/well for BMDMs, iBMDMs, or 10^5^ cells/well for THP-1 and subjected to stimulation. After the stipulated incubation period, SYTOX Green (Thermo Fisher Scientific, S7020) was administered following the manufacturer’s protocol. The ensuing images were subjected to analysis using the software packaged with the IncuCyte imager.

### Cytokine analysis

Cytokine levels were measured using IL-1β ELISA (Invitrogen, BMS6002TEN) and IL-18 ELISA (R&D Systems, DY7625-05). For in vitro experiments, cell culture supernatants from infected BMDMs were collected and centrifuged to remove cellular debris prior to cytokine quantification. For in vivo experiments, bronchoalveolar lavage fluid (BALF) was harvested from infected mice. All samples were analyzed according to the manufacturers’ instructions.

### Immunoblotting analysis

Immunoblotting was conducted following established protocols^7,11,12,23,28,38,40–44^. To assess caspase activity, BMDMs were lysed along with their supernatant using 50 μL of caspase lysis buffer (comprising 1× protease inhibitors, 1× phosphatase inhibitors, 10% NP-40, and 25 mM DTT), followed by the addition of 100 μL of 4× SDS loading buffer. For signaling analysis, BMDM supernatants were collected at specified time points, and after one wash with PBS, cells were lysed with RIPA buffer. For all in vitro immunoblot analyses, BMDMs and iBMDMs were seeded at 1 × 10⁶ cells per well, and lysates were prepared from the entire well. Equal numbers of cells were processed under each condition, so additional protein concentration-based normalization was not performed. Instead, equal volumes of lysates were loaded for SDS-PAGE, and protein loading consistency was verified using internal control proteins, including β-actin or GAPDH. For in vivo immunoblot analyses, approximately 100 mg of lung tissue from infected mice was homogenized in cold RIPA buffer. Tissue lysates were clarified using centrifugation, and protein concentrations were determined using a BCA protein assay. Lysates were then adjusted to a final concentration of 2 mg/mL with the addition of RIPA buffer. Proteins were resolved through electrophoresis on 8-15% polyacrylamide gels. After electrophoretic transfer onto PVDF membranes (Millipore, IPVH00010), non-specific binding was blocked with 5% skimmed milk. Subsequently, the membranes were incubated with the following primary antibodies: anti-caspase-1 (AdipoGen, AG-20B-0042, 1:2000), anti-caspase-3 (CST, #9662, 1:2000), anti-cleaved caspase-3 (CST, #9661, 1:2000), anti-caspase-7 (CST, #9492, 1:2000), anti-cleaved caspase-7 (CST, #9491, 1:2000), anti-caspase-8 (CST, #4927, 1:2000), anti-cleaved caspase-8 (CST, #8592, 1:2000), anti-pMLKL (CST, #37333, 1:2000), anti-MLKL (Abgent, AP14272b, 1:2000), anti-GSDMD (Abcam, ab209845, 1:2000), anti-β-actin (Proteintech, 66009-1-IG, 1:5000), anti-ICP0 (Santa Cruz Biotechnology, sc‑53070, 1:1000), anti-ICP8 (Santa Cruz Biotechnology, sc-53329, 1:1000), anti-gD (Santa Cruz Biotechnology, sc-21719, 1:1000), anti-gC (Abcam, ab6509, 1:2000), anti-GAPDH (Santa Cruz Biotechnology, sc-365062, 1:3000), anti-VP22 (kindly provided by Yasushi Kawaguchi, 1:2000), anti-CASP6 (CST, #9762, 1:2000), anti-CASP9 (CST, #9504, 1:2000), anti-tRIPK3 (Prosci, #2283, 1:2000), anti-cGAS (CST, #31659, 1:2000), anti-STING (CST, #13647, 1:2000), anti-IRF1 (CST, #8478, 1:2000), anti-ASC (Millipore, #04-147), and anti-AIM2 (Abcam, ab119791, 1:2000). After washing, the membranes were exposed to suitable horseradish peroxidase (HRP)-conjugated secondary antibodies (diluted 1:5000; Jackson ImmunoResearch Laboratories, anti-rabbit [111-035-047], anti-mouse [315-035-047]) for 1 h. Protein bands were visualized using Luminata Forte Western HRP Substrate (Millipore, WBLUF0500), and the membranes were examined using Amersham ImageQuant 800 UV. The resulting images were analyzed utilizing ImageJ software (v1.53a). Uncropped immunoblot images are provided in the Source data file.

### Immunofluorescence staining

Immunofluorescence staining was conducted as previously described^7,11,12,27,43,44^. In brief, the cells were fixed with 4% paraformaldehyde (PFA) for 10 min, permeabilized with PBS containing 0.5% Triton X-100 for 3 min, and subsequently incubated in PBS containing 1% skim milk for 1 h. The coverslips were then incubated with anti-ASC (Millipore, 04-147) and anti-tubulin (CST, 2144) antibodies. The secondary antibodies used were Alexa Fluor 488- conjugated anti-mouse IgG (Life Technologies, A21202; 1:200) and Alexa Fluor 568-conjugated anti-rabbit IgG (Life Technologies, A10042; 1:200). For visualization, DAPI mounting medium (P36931; Invitrogen, Carlsbad, CA, USA) was used for counterstaining. The images were captured via a confocal laser scanning microscope (LSM900; Carl Zeiss) equipped with a 63× apochromatic objective.

### Immunoprecipitation

Immunoprecipitation was performed following previously established protocols^7,11,28,44^. Briefly, 1 × 10⁷ cells per condition were seeded and subjected to HSV-1 infection or stimulation as indicated. Cells were then lysed in ice-cold lysis buffer containing 20 mM Tris-HCl (pH 7.4), 100 mM NaCl, 30 mM KCl, and 0.1% NP-40. Lysates were clarified by centrifugation at 16,000 × *g* for 10 min at 4 °C to remove insoluble debris. Protein concentrations were quantified using a BCA protein assay, and lysates were normalized to a final concentration of 1 mg/mL total protein. Equal amounts of protein were used for each immunoprecipitation reaction. The normalized lysates were incubated overnight at 4 °C with either an IgG control antibody (CST, 3900S) or an anti-ASC antibody (AdipoGen, AG-25B-006-C100), together with protein A Sepharose beads (GE Healthcare, GE17-5280-01). Following extensive washing with the lysis buffer, immunoprecipitated proteins were eluted using 0.1 mM glycine (pH 3.0).

### RT-qPCR

HaCaT cells were infected with HSV-1 HF (WT, ΔT_14_, and ΔT_14_-Rev) or F (WT, T_27_, and T_27_-Rev) strains at an MOI of 3. At 6 hpi, total RNA was extracted using the AccuPrep Universal RNA Extraction Kit (Bioneer, K-3140). Complementary DNA (cDNA) was synthesized from the isolated RNA using the High-Capacity RNA-to-cDNA Kit (Applied Biosystems, 4387406). Quantitative PCR was performed with the TOPreal SYBR Green qPCR High-ROX Premix (Enzynomics, RT501M) to measure UL25 and UL26 transcript levels. Gene expression values were normalized to ICP27 mRNA, which served as an internal control.

### Analysis for HSV-1 genomic sequence and protein structure

Sequence alignment was performed using the BioPython package to compare the VP22 protein sequences of HSV-1 strains HF (GenBank Accession No. DQ889502), F (GenBank Accession No. GU734771), and KOS (GenBank Accession No. JQ673480). The Multiple Sequence Alignment module facilitated the identification of sequence variations, with a specific focus on missense mutations exclusive to the HF strain. In addition to the above procedures, a comprehensive search was conducted for the presence of specific sequences within the whole genomic sequence of HSV-1. The three-dimensional structure of the VP22 protein was predicted using the SWISS-MODEL server, which provided insights into the structural implications of the HF strain-specific variants^33^. Subsequent modeling was performed to assess the effects of these variants on the protein’s overall structure.

### Identification and quantification of poly(T) tracts in HSV-1 genomes

A total of 492 complete HSV-1 genome sequences, including reference strains, laboratory strains, and clinical isolates, were obtained from the NCBI Virus database (Supplementary Data 1 and 2). Each genome assembly was computationally screened for homopolymeric thymidine [poly(T)] tracts using custom Python scripts. poly(T) tracts of predefined lengths, poly(T)14 (≥14 consecutive thymidines) and poly(T)23 (≥23 consecutive thymidines), were identified across all genomes. Each genome was annotated with binary indicators reflecting the presence or absence of poly(T)14 and poly(T)23 motifs. Genomes were grouped according to strain designation, including laboratory strains (H166, KOS63, RH2), clinical samples, and other annotated HSV-1 strains. The proportion of genomes containing poly(T)14 or poly(T)23 motifs was calculated for each group as the percentage of positive samples relative to the total number of genomes within the group. Percentage distributions were summarized and visualized using Python-based data analysis workflows (pandas, matplotlib, and seaborn). Visualization parameters were standardized to generate publication-quality figures. This analysis enabled comparative assessment of poly(T) tract enrichment across HSV-1 strains and revealed that poly(T)23 motifs were exclusively detected in H166, KOS63, RH2, and clinical isolates, whereas poly(T)14 motifs were broadly distributed across the HSV-1 population.

### In vivo infection

Age- and sex-matched cohorts of 6- to 8-week-old WT and *Aim2*^*−/−*^ mice, housed together, were utilized for the infection experiments. Mice were anesthetized with 250 mg/kg^−1^ avertin and then infected intranasally with HSV-1 in 100 μL PBS containing approximately 5 × 10^5^ PFU. Infected mice were monitored over a period of 14 days for survival. Lung tissue collected at 5 days post-infection was homogenized in 1 mL PBS for viral titers to be enumerated by plaque assays.

### Statistical analysis

We performed data analysis using GraphPad Prism 10.0 software. The data are expressed as the mean ± SEM or ± SE. We determined statistical significance using a two-tailed unpaired t-test, a one-way ANOVA (Dunnett’s multiple comparisons test), and two-way ANOVA (Šídák’s multiple comparisons test) with multiple comparisons for analyses involving multiple groups. Survival curves were analyzed using the log-rank (Mantel-Cox) test. Statistical significance was acknowledged for *P* values below 0.05 and denoted as *P < 0.05, **P < 0.01, ***P < 0.001, and ****P < 0.0001.

### Reporting summary

Further information on research design is available in the Nature Portfolio Reporting Summary linked to this article.

## Supplementary information

Supplementary Information

Description of additional supplementary files

Supplementary Data 1

Supplementary Data 2

Supplementary Data 3

Reporting summary

Transparent Peer Review file

## Source data

Source data

## Data Availability

All data supporting the findings of this study are available within the article, its Supplementary Information files, and the Source data file. Publicly available HSV-1 genome sequences used in this study were obtained from the NCBI database under the following accession numbers: GU734771.1 (HSV-1 strain F), DQ889502.1 (HSV-1 strain HF), and JQ673480 (HSV-1 strain KOS). Source data are provided with this paper.
