# Supplementary material for: Herpes simplex virus 1 harboring poly(T) DNA sequences as a key ligand for AIM2 inflammasome activation and host defense
Source: Nat Commun. 2026 Apr 13;17:5161. doi: 10.1038/s41467-026-71896-w (PMC13250049; doi:10.1038/s41467-026-71896-w)
Supplement: Supplementary file 1 — Supplementary Information [file 41467_2026_71896_MOESM1_ESM.pdf]

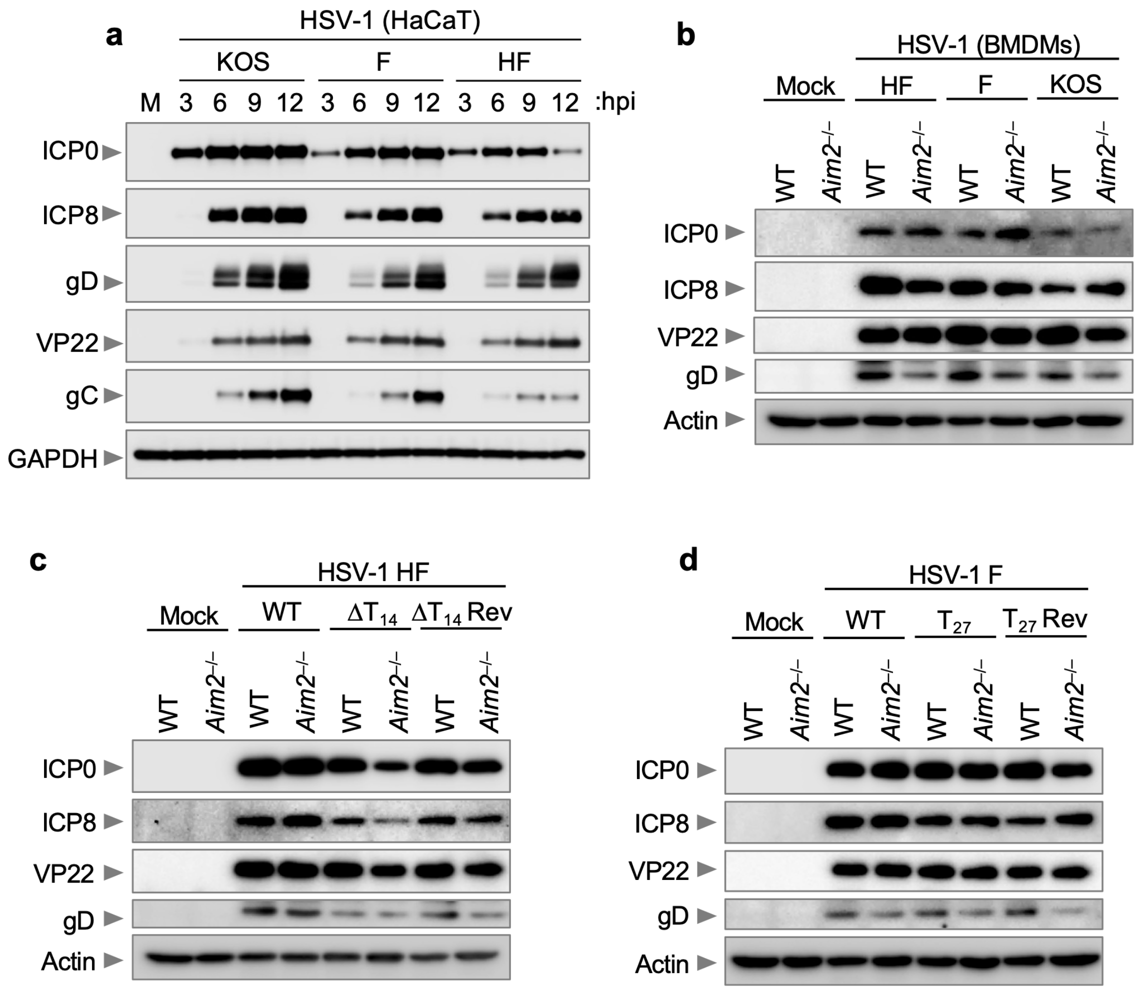
a
HSV-1 (HaCaT)
KOS
F
HF
M 3 6 9 12 3 6 9 12 3 6 9 12 :hpi
ICP0
ICP8
gD
VP22
gC
GAPDH
b
HSV-1 (BMDMs)
Mock
HF
F
KOS
WT
Aim2−/−
ICP0
ICP8
VP22
gD
Actin
c
HSV-1 HF
Mock
WT
ΔT14
ΔT14 Rev
WT
Aim2−/−
ICP0
ICP8
VP22
gD
Actin
d
HSV-1 F
Mock
WT
T27
T27 Rev
WT
Aim2−/−
ICP0
ICP8
VP22
gD
Actin

Supplementary Figure S1. Comparable infection efficiency and viral protein expression among HSV-1 strains irrespective of AIM2 status.

a, Immunoblot analysis of representative HSV-1 viral proteins, including ICP0, ICP8, gD, VP22, and gC in HaCaT cells infected with HSV-1 strains HF, F, or KOS at the indicated time points post-infection. Expected molecular weights: ICP0 (~110 kDa), ICP8 (~128 kDa), gD (~55 kDa), VP22 (~38 kDa), and gC (~120 kDa). **b–d,** Immunoblot analysis of ICP0, ICP8, VP22, and gD in wild-type (WT) or *Aim2*$^{-/-}$ bone marrow–derived macrophages (BMDMs) infected with HSV-1 strains HF, F, or KOS (**b**); WT HF, the mutant HSV-1 strain lacking the 14-mer poly(T) DNA sequence (HF ΔT_{14}), or the corresponding revertant virus (HF ΔT_{14}-Rev) (**c**); or WT F, the HSV-1 F strain harboring a 27-mer poly(T) sequence inserted into the UL25–UL26 intergenic region (F T_{27}), as well as the corresponding revertant virus in which the poly(T) sequence was precisely removed by homologous recombination (F T_{27}-Rev) (**d**). Expected molecular weights: ICP0 (~110 kDa), ICP8 (~128 kDa), VP22 (~38 kDa), and gD (~55 kDa). Panels **a–d** represent data from three independent experiments.

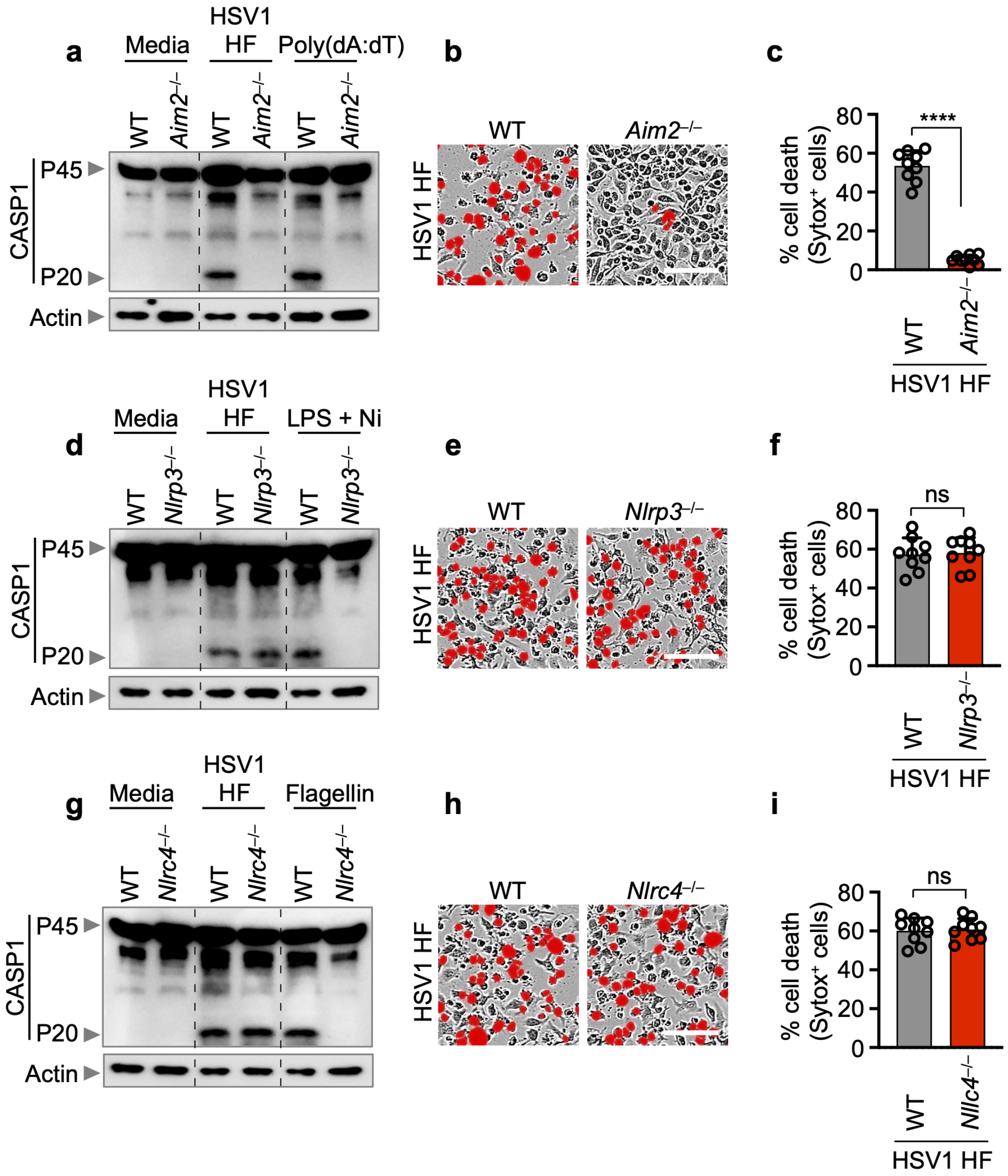
a
Media
HSV1 HF
Poly(dA:dT)
WT
Aim2–/–
CASP1
P45
P20
Actin
b
WT
Aim2–/–
HSV1 HF
c
% cell death (Sytox+ cells)
****
HSV1 HF
d
Media
HSV1 HF
LPS + Ni
Nlrp3–/–
e
Nlrp3–/–
f
ns
g
Flagellin
Nlrc4–/–
h
i

Supplementary Figure S2. AIM2 serves as an inflammasome sensor for the HF strain of HSV-1.

a, Immunoblot analysis of pro-caspase-1 (CASP1; P45) and cleaved CASP1 (P20) in wild-type (WT) or *Aim2*$^{-/-}$ bone marrow-derived macrophages (BMDMs) after infection with HSV-1 strain HF. **b,** Cell death evaluation in BMDMs after HSV-1 infection. **c,** Quantification of cell death in (**b**). **d–f,** Immunoblot analysis of pro-caspase-1 and cleaved CASP1 (**d**), cell death evaluation (**e**), and quantification of cell death (**f**) in WT or *Nlrp3*$^{-/-}$ BMDMs after infection with HSV-1 strain HF. **g–i,** Immunoblot analysis of pro-caspase-1 and cleaved CASP1 (**g**), cell death evaluation (**h**), and quantification of cell death (**i**) in WT or *Nlrc4*$^{-/-}$ BMDMs after infection with HSV-1 strain HF. Panels **a, d, g** represent data from three independent experiments. Expected molecular weights: pro-caspase-1 (P45, ~45 kDa) and cleaved caspase-1 (P20, ~20 kDa). Panels **b, e, h** are representative of three biologically independent experiments. Dead cells are indicated in red. Scale bar: 100 µm. Panels **c, f, i** depict data as mean ± s.e.m. 'ns', not significant; ****$P < 0.0001$ (two-tailed t-test; $n = 9$ biologically independent samples from three independent experiments). Source data are provided as a Source Data file.

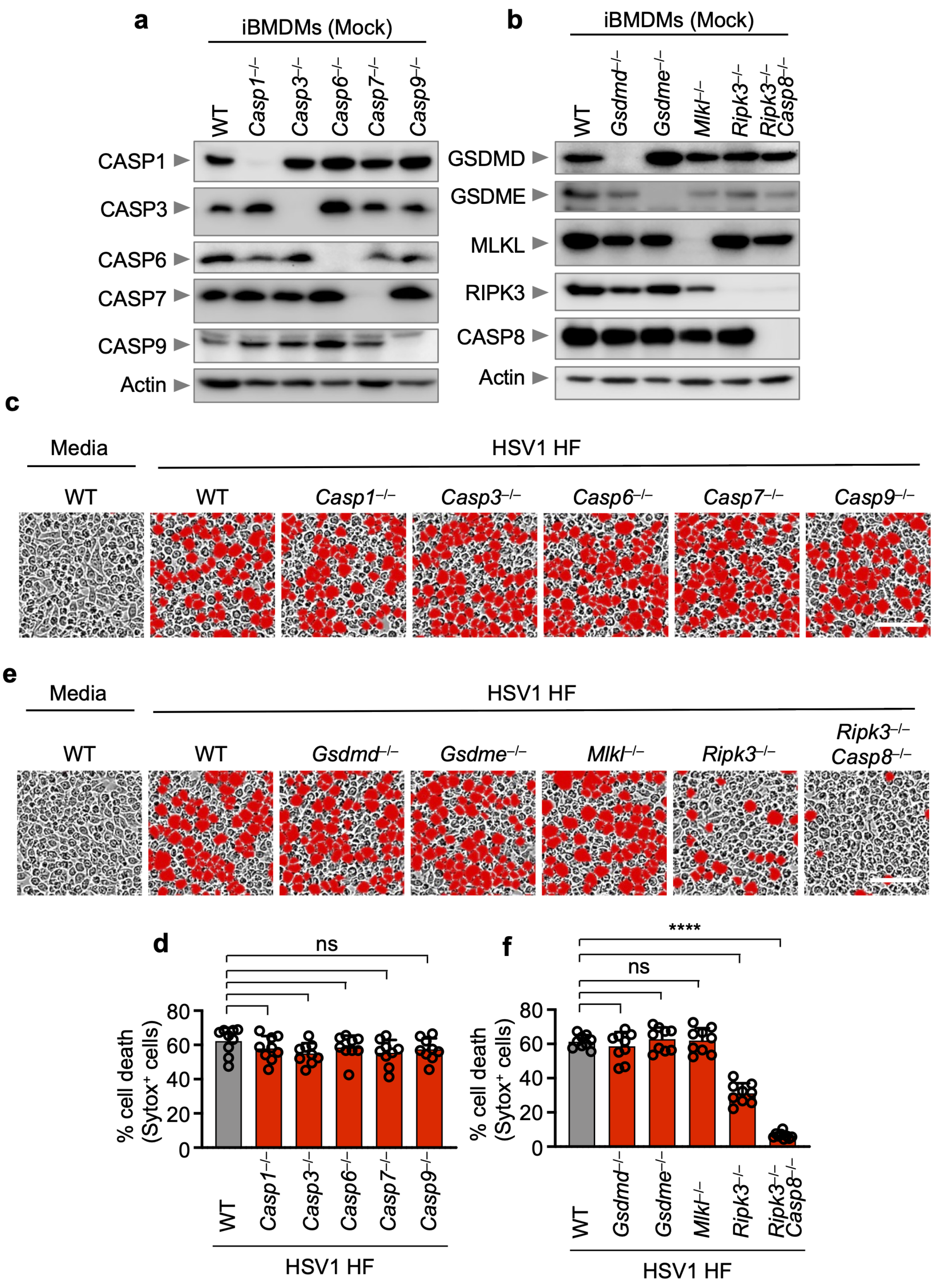

a
iBMDMs (Mock)
WT
Casp1–/–
Casp3–/–
Casp6–/–
Casp7–/–
Casp9–/–
CASP1
CASP3
CASP6
CASP7
CASP9
Actin
b
iBMDMs (Mock)
WT
Gsdmd–/–
Gsdme–/–
Mlkl–/–
Ripk3–/–
Ripk3–/– Casp8–/–
GSDMD
GSDME
MLKL
RIPK3
CASP8
Actin
c
Media
HSV1 HF
WT
WT
Casp1–/–
Casp3–/–
Casp6–/–
Casp7–/–
Casp9–/–
e
Media
HSV1 HF
WT
WT
Gsdmd–/–
Gsdme–/–
Mlkl–/–
Ripk3–/–
Ripk3–/– Casp8–/–
d
ns
% cell death (Sytox+ cells)
0
20
40
60
80
WT
Casp1–/–
Casp3–/–
Casp6–/–
Casp7–/–
Casp9–/–
HSV1 HF
f
****
ns
% cell death (Sytox+ cells)
0
20
40
60
80
WT
Gsdmd–/–
Gsdme–/–
Mlkl–/–
Ripk3–/–
Ripk3–/– Casp8–/–
HSV1 HF

Supplementary Figure S3. RIPK3 and caspase-8 are important executioner molecules of cell death against the HF strain of HSV-1.

a, b, Immunoblot analysis validating knockout of caspase-1 (CASP1), caspase-3 (CASP3), caspase-6 (CASP6), caspase-7 (CASP7), and caspase-9 (CASP9) in wild-type (WT), *Casp1*$^{-/-}$, *Casp3*$^{-/-}$, *Casp6*$^{-/-}$, *Casp7*$^{-/-}$, or *Casp9*$^{-/-}$ immortalized bone marrow–derived macrophages (iBMDMs) (**a**), or of GSDMD, GSDME, MLKL, RIPK3, and CASP8 in WT, *Gsdmd*$^{-/-}$, *Gsdme*$^{-/-}$, *Mlkl*$^{-/-}$, *Ripk3*$^{-/-}$, or *Ripk3*$^{-/-}$*Casp8*$^{-/-}$ iBMDMs (**b**). Expected molecular weights: CASP1 (~45 kDa), CASP3 (~35 kDa), CASP6 (~34 kDa), CASP7 (~35 kDa), CASP9 (~47 kDa), GSDMD (~53 kDa), GSDME (~55 kDa), MLKL (~54 kDa), RIPK3 (~57 kDa), and CASP8 (~55 kDa). Data are representative of three independent experiments. **c,** Cell death evaluation in WT, *Casp1*$^{-/-}$, *Casp3*$^{-/-}$, *Casp6*$^{-/-}$, *Casp7*$^{-/-}$, or *Casp9*$^{-/-}$ iBMDMs after HSV-1 strain HF infection. **d,** Quantification of cell death in (**c**). **e,** Cell death evaluation in WT, *Gsdmd*$^{-/-}$, *Gsdme*$^{-/-}$, *Mlkl*$^{-/-}$, *Ripk3*$^{-/-}$, or *Ripk3*$^{-/-}$*Casp8*$^{-/-}$ iBMDMs after HSV-1 strain HF infection. **f,** Quantification of cell death in (**e**). Panels **c, e** are representative of three biologically independent experiments. Dead cells are indicated in red. Scale bar: 100 μm. Panels **d, f** depict data as mean ± s.e.m. 'ns', not significant; ****P < 0.0001 (one-way ANOVA with Dunnett's multiple comparisons test; n = 9 biologically independent samples from three independent experiments). Source data are provided as a Source Data file.

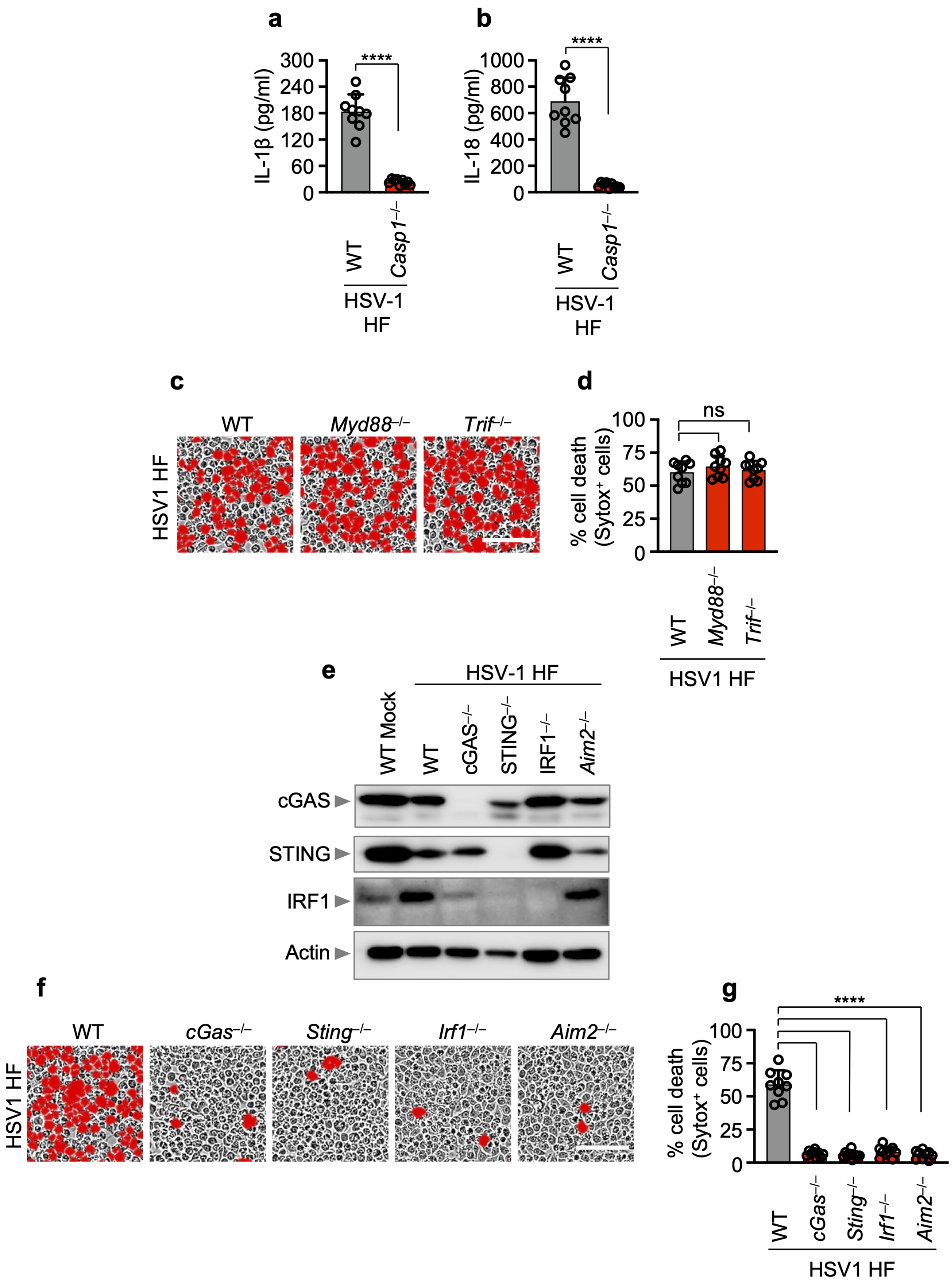
a
****
IL-1β (pg/ml)
300
240
180
120
60
0
WT
Casp1–/–
HSV-1
HF
b
****
IL-18 (pg/ml)
1000
800
600
400
200
0
WT
Casp1–/–
HSV-1
HF
c
WT
Myd88–/–
Trif–/–
HSV1 HF
d
ns
% cell death
(Sytox+ cells)
100
75
50
25
0
WT
Myd88–/–
Trif–/–
HSV1 HF
e
HSV-1 HF
WT Mock
WT
cGAS–/–
STING–/–
IRF1–/–
Aim2–/–
cGAS
STING
IRF1
Actin
f
WT
cGas–/–
Sting–/–
Irf1–/–
Aim2–/–
HSV1 HF
g
****
% cell death
(Sytox+ cells)
100
75
50
25
0
WT
cGas–/–
Sting–/–
Irf1–/–
Aim2–/–
HSV1 HF

Supplementary Figure S4. cGAS–STING–IRF1 signaling is required for HSV-1–induced inflammatory cell death.

a, b, IL-1β (**a**) and IL-18 (**b**) release assessment in wild-type (WT) or *Casp1*$^{-/-}$ bone marrow–derived macrophages (BMDMs) following HSV-1 strain HF infection. **c,** Cell death evaluation in WT, *Myd88*$^{-/-}$, or *Trif*$^{-/-}$ immortalized bone marrow-derived macrophages (iBMDMs) after HSV-1 strain HF infection. **d,** Quantification of cell death in (**c**). **e,** Immunoblot analysis validating knockout of cGAS, STING, and IRF1 in WT, *cGas*$^{-/-}$, *Sting*$^{-/-}$, *Irf1*$^{-/-}$, or *Aim2*$^{-/-}$ iBMDMs after HSV-1 strain HF infection. Expected molecular weights: cGAS (~60 kDa), STING (~35 kDa), and IRF1 (~48 kDa). Data are representative of three independent experiments. **f,** Cell death evaluation in WT, *cGas*$^{-/-}$, *Sting*$^{-/-}$, *Irf1*$^{-/-}$, or *Aim2*$^{-/-}$ iBMDMs after HSV-1 strain HF infection. **g,** Quantification of cell death in (**f**). **a, b** depict data as mean ± s.e.m. ****$P < 0.0001$ (two-tailed t-test; $n = 9$ biologically independent samples from three independent experiments). Panels **c, f** are representative of three biologically independent experiments. Dead cells are indicated in red. Scale bar: 100 μm. **d, g** depict data as mean ± s.e.m. 'ns', not significant; ****$P < 0.0001$ (one-way ANOVA with Dunnett's multiple comparisons test; $n = 9$ biologically independent samples from three independent experiments). Source data are provided as a Source Data file.

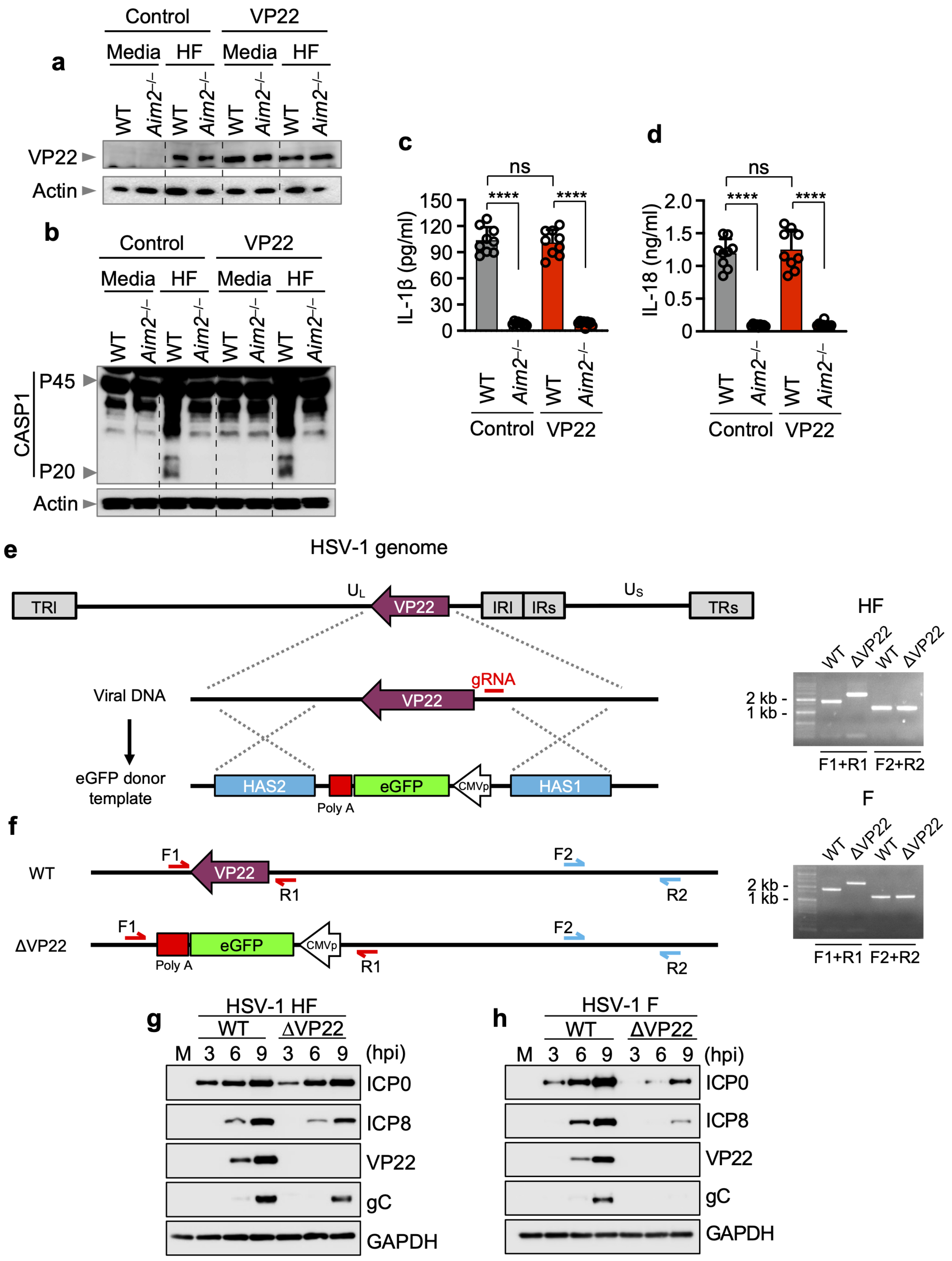
a
Control
VP22
Media
HF
WT
Aim2−/−
VP22
Actin
b
CASP1
P45
P20
c
IL-1β (pg/ml)
ns
****
d
IL-18 (ng/ml)
e
HSV-1 genome
TRl
UL
IRl
IRs
US
TRs
Viral DNA
gRNA
eGFP donor template
HAS2
eGFP
CMVp
HAS1
Poly A
HF
ΔVP22
2 kb -
1 kb -
F1+R1
F2+R2
F
f
F1
R1
F2
R2
g
HSV-1 HF
M
(hpi)
ICP0
ICP8
gC
GAPDH
h
HSV-1 F

Supplementary Figure S5. Validation of VP22 overexpression and generation of VP22-deficient HSV-1 viruses.

a, Immunoblot analysis of VP22 in control or VP22 plasmid-transfected wild-type (WT) or *Aim2*$^{-/-}$ bone marrow-derived macrophages (BMDMs) after infection with HSV-1 HF strain. Expected molecular weight: VP22 (~38 kDa). **b,** Immunoblot analysis of pro-caspase-1 (CASP1; P45) and cleaved CASP1 (P20) in control or VP22 plasmid-transfected WT or *Aim2*$^{-/-}$ BMDMs after infection with HSV-1 HF strain. Expected molecular weights: pro-caspase-1 (P45, ~45 kDa) and cleaved caspase-1 (P20, ~20 kDa). **c, d,** IL-1β (**c**) and IL-18 (**d**) release assessment in control or VP22 plasmid-transfected WT or *Aim2*$^{-/-}$ BMDMs following HSV-1 HF strain infection. **e,** Schematic representation of CRISPR/Cas9-mediated deletion of the VP22 gene in HSV-1. A guide RNA (gRNA) targeting VP22 is used to introduce a double-strand break, and a donor template containing an eGFP cassette flanked by homology arm sequences (HAS) is provided to promote homology-directed repair (HDR). **f,** PCR analysis confirming VP22 deletion in HF and F backgrounds. Primer pair F1/R1 is used to detect size differences at the insertion site, while F2/R2 serves as a positive control. **g, h** Immunoblot analysis of representative HSV-1 viral proteins, including ICP0, ICP8, VP22, and gC, in HaCaT cells infected with HSV-1 HF (WT or ΔVP22) (**g**) or HSV-1 F (WT or ΔVP22) (**h**). Expected molecular weights: ICP0 (~110 kDa), ICP8 (~128 kDa), VP22 (~38 kDa), and gC (~120 kDa). Cell lysates are collected at 3, 6, and 9 h post-infection (hpi). Panels **a, b, g, h** represent data from three independent experiments. Panels **c, d** depict data as mean ± s.e.m. 'ns', not significant; ****$P < 0.0001$ (one-way ANOVA with Dunnett's multiple comparisons test; $n = 9$ biologically independent samples from three independent experiments). Source data are provided as a Source Data file.

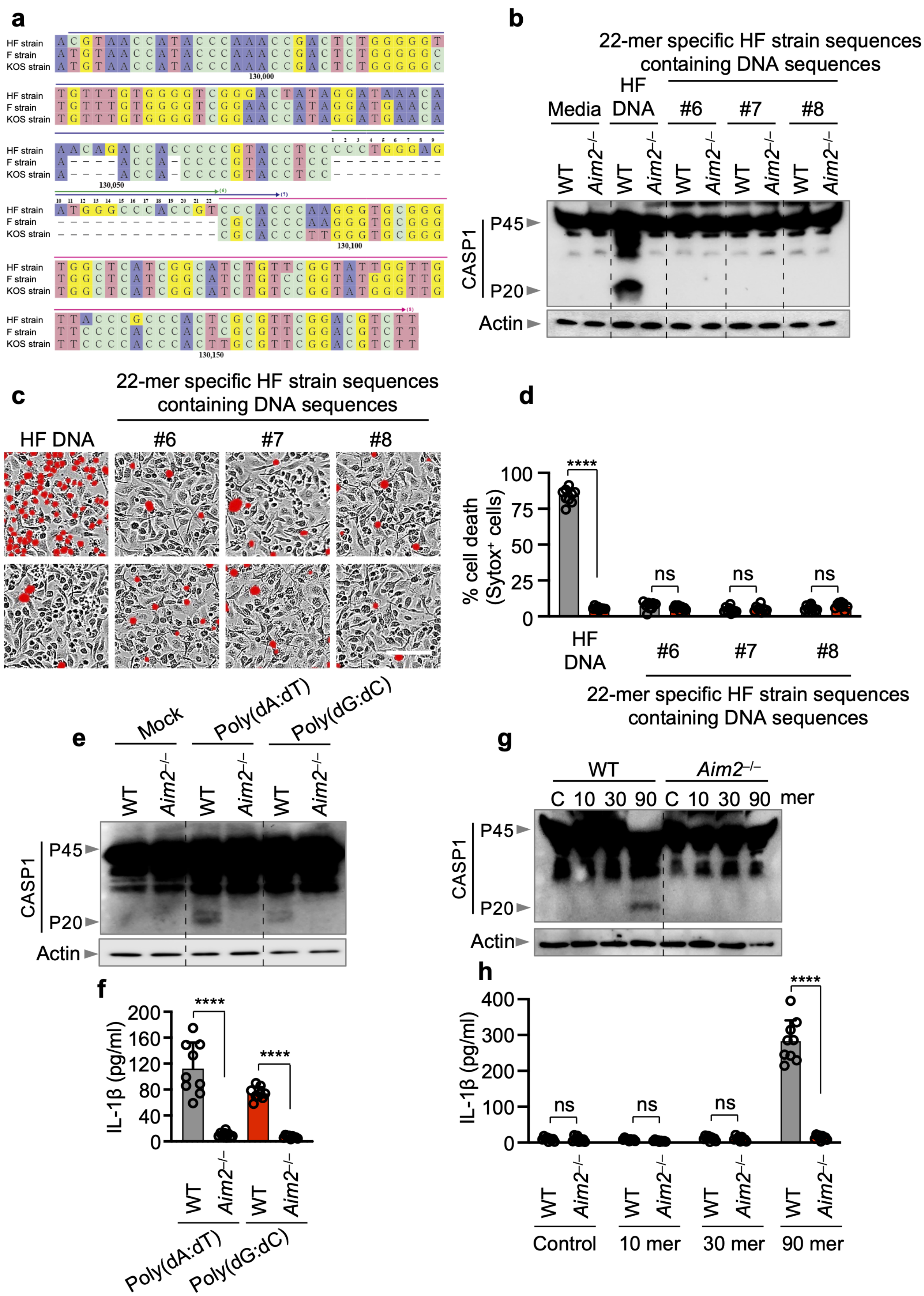
a
HF strain
F strain
KOS strain
130,000
130,050
130,100
130,150
b
22-mer specific HF strain sequences containing DNA sequences
Media
HF DNA
#6
#7
#8
WT
Aim2−/−
CASP1
P45
P20
Actin
c
22-mer specific HF strain sequences containing DNA sequences
HF DNA
#6
#7
#8
WT
Aim2−/−
d
% cell death (Sytox+ cells)
100
75
50
25
0
****
ns
HF DNA
#6
#7
#8
22-mer specific HF strain sequences containing DNA sequences
e
Mock
Poly(dA:dT)
Poly(dG:dC)
WT
Aim2−/−
CASP1
P45
P20
Actin
f
IL-1β (pg/ml)
200
160
120
80
40
0
****
WT
Aim2−/−
Poly(dA:dT)
Poly(dG:dC)
g
WT
Aim2−/−
C 10 30 90 C 10 30 90 mer
CASP1
P45
P20
Actin
h
IL-1β (pg/ml)
400
300
200
100
0
ns
****
WT
Aim2−/−
Control
10 mer
30 mer
90 mer

Supplementary Figure S6. HF strain of HSV-1 containing 5'-CCCTGGGAGATGGGCCCACCGT-3' DNA sequences does not function as a ligand for AIM2 inflammasome activation.

a, Schematic diagram of designing specific dsDNA to target unique regions HSV-1 HF (5'-CCCTGGGAGATGGGCCCACCGT-3') from F and KOS DNA sequence. Indicated dsDNA (#6: 22 mer, #7: 110 mer, and #8: 109 mer) were designed. **b,** Immunoblot analysis of pro-caspase-1 (CASP1; P45) and cleaved CASP1 (P20) in wild-type (WT) or *Aim2*$^{-/-}$ bone marrow-derived macrophages (BMDMs) after transfection of indicated dsDNA. **c,** Cell death evaluation in WT or *Aim2*$^{-/-}$ BMDMs after transfection of indicated dsDNA. Dead cells are indicated in red. Scale bar: 100 μm. Images are representative of three biologically independent experiments. **d,** Quantification of cell death in (**c**). **e,** Immunoblot analysis of pro-caspase-1 (CASP1; P45) and cleaved CASP1 (P20) in WT or *Aim2*$^{-/-}$ BMDMs following transfection with poly(dA:dT) or poly(dG:dC). **f,** IL-1β release assessment in WT or *Aim2*$^{-/-}$ BMDMs following transfection with poly(dA:dT) or poly(dG:dC). **g,** Immunoblot analysis of pro-caspase-1 (CASP1; P45) and cleaved CASP1 (P20) in WT or *Aim2*$^{-/-}$ BMDMs following transfection with double-stranded poly(T) DNA of 10-, 30-, or 90-mer length. **h,** IL-1β release assessment in WT or *Aim2*$^{-/-}$ BMDMs following transfection with double-stranded poly(T) DNA of 10-, 30-, or 90-mer length. Panels **b, e, g** represent data from three independent experiments. Expected molecular weights: pro-caspase-1 (P45, ~45 kDa) and cleaved caspase-1 (P20, ~20 kDa). **f,** depict data as mean ± s.e.m. ****P < 0.0001 (one-way ANOVA with Dunnett's multiple comparisons test; n = 9 biologically independent samples from three independent experiments). **d, h** depict data as mean ± s.e.m. 'ns', not significant; ****P < 0.0001 (two-way ANOVA with Šídák's multiple comparisons test; n = 9 biologically independent samples from three independent experiments). Source data are provided as a Source Data file.

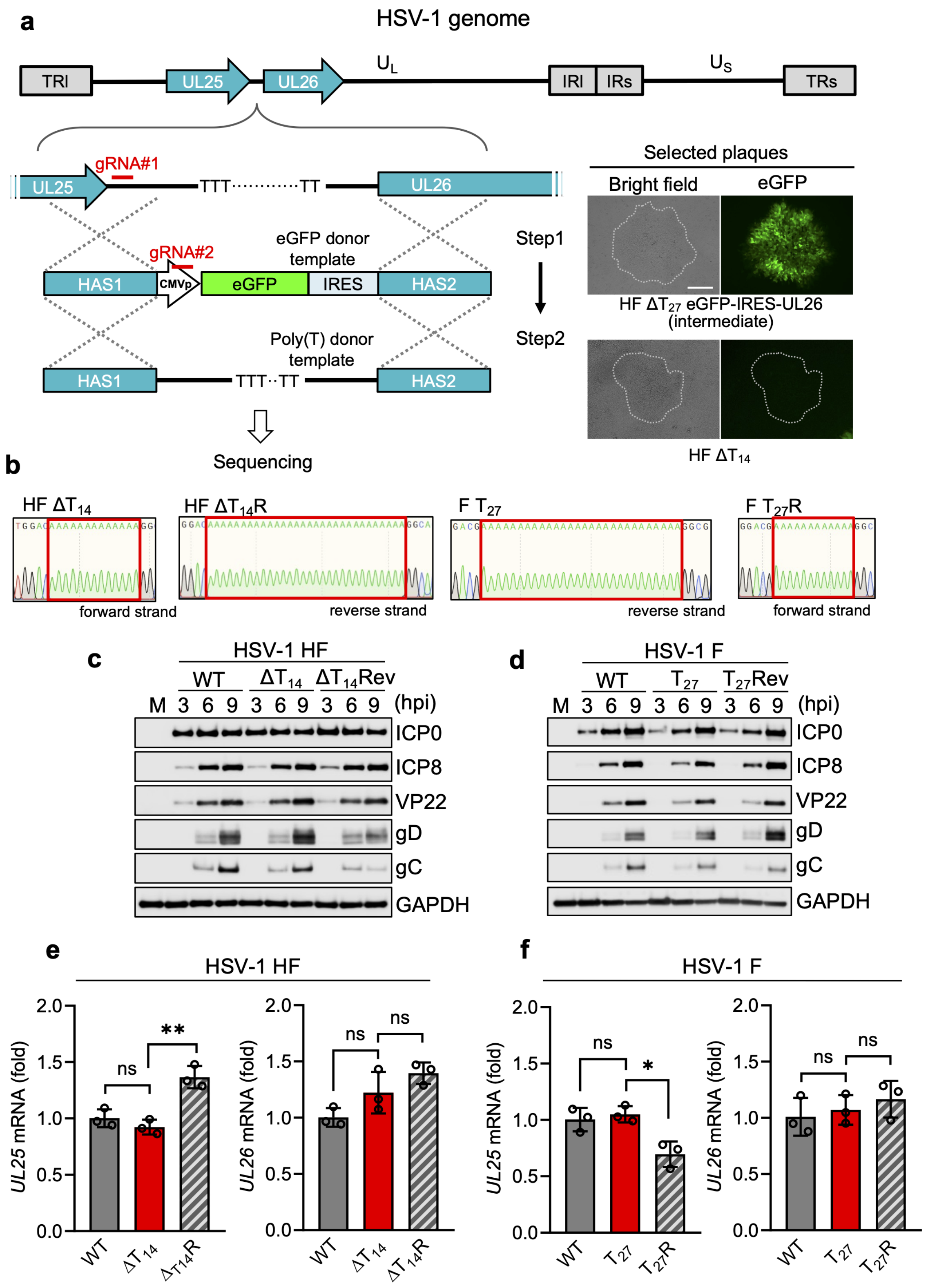
a
HSV-1 genome
TRl
UL25
UL26
UL
IRl
IRs
US
TRs
gRNA#1
TTT···········TT
gRNA#2
eGFP donor template
HAS1
CMVp
eGFP
IRES
HAS2
Poly(T) donor template
TTT··TT
Sequencing
Step1
Step2
Selected plaques
Bright field
eGFP
HF ΔT27 eGFP-IRES-UL26 (intermediate)
HF ΔT14
b
HF ΔT14
HF ΔT14R
F T27
F T27R
forward strand
reverse strand
reverse strand
forward strand
c
HSV-1 HF
WT
ΔT14
ΔT14Rev
M 3 6 9 3 6 9 3 6 9 (hpi)
ICP0
ICP8
VP22
gD
gC
GAPDH
d
HSV-1 F
WT
T27
T27Rev
M 3 6 9 3 6 9 3 6 9 (hpi)
ICP0
ICP8
VP22
gD
gC
GAPDH
e
HSV-1 HF
UL25 mRNA (fold)
UL26 mRNA (fold)
ns
**
ns
ns
WT
ΔT14
ΔT14R
f
HSV-1 F
UL25 mRNA (fold)
UL26 mRNA (fold)
ns
*
ns
ns
WT
T27
T27R
0.0
0.5
1.0
1.5
2.0

Supplementary Figure S7. CRISPR/Cas9-mediated generation, sequence validation, and viral gene expression profiling of recombinant HSV-1 HF and F strains.

a, Schematic of CRISPR/Cas9-mediated generation of the HSV-1 HF ΔT_{14} mutant lacking 14 thymidines between UL25 and UL26. Recombinants are obtained through two-step recombination and confirmed by Sanger sequencing. HAS, homology arm sequences; scale bar, 200 μm. **b,** Representative Sanger sequencing chromatograms of the poly(T) region from the recombinant viruses. Reverse strand sequencing reads confirm the expected poly(T) stretch in each recombinant. **c, d,** Immunoblot analysis of cells infected with HF strain (WT, ΔT_{14}, ΔT_{14}-Rev) (**c**) or F strain (WT, T_{27}, T_{27}-Rev) (**d**) and harvested at the indicated times post infection (hpi). The expression of viral proteins representing different kinetic classes, ICP0 (immediate early), ICP8 (early), VP22 (leaky late), gD (leaky late), and gC (true late), are examined. Expected molecular weights: ICP0 (~110 kDa), ICP8 (~128 kDa), VP22 (~38 kDa), gD (~55 kDa), and gC (~120 kDa). Data are representative of three independent experiments. **e, f,** RT-qPCR analysis of UL25 and UL26 mRNA expression in cells infected with the HF strain (WT, ΔT_{14}, ΔT_{14}-Rev) (**e**) and F strain (WT, T_{27}, T_{27}-Rev) (**f**). Data are normalized to ICP27 mRNA levels. Values represent the mean ± SD from three independent experiments (*n* = 3 biologically independent experiments). Statistical significance is determined using a two-tailed unpaired Student's t-test ('ns', not significant; *$P < 0.05$; **$P < 0.01$). Source data are provided as a Source Data file.

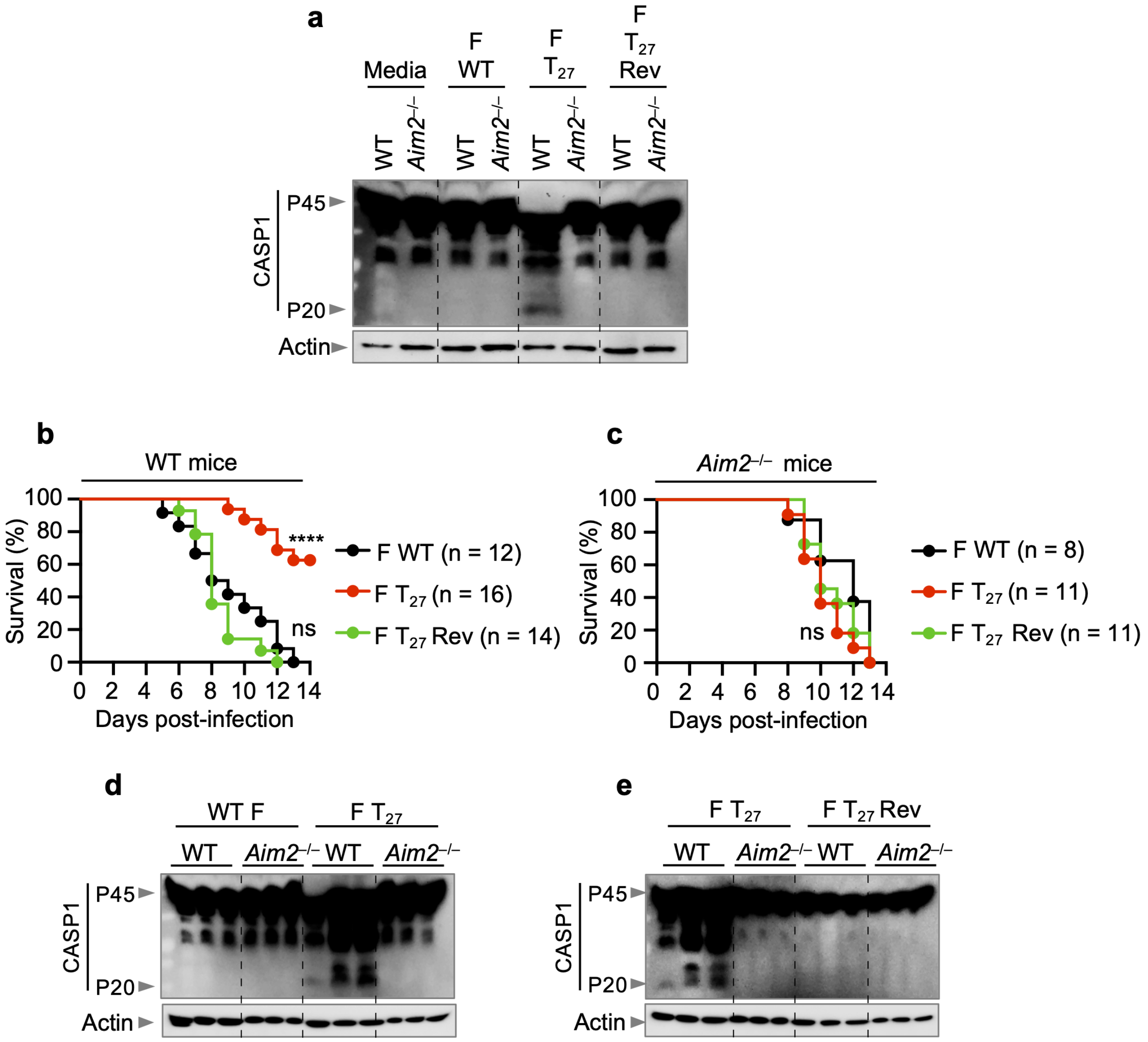
a
Media
F WT
F T27
F T27 Rev
WT
Aim2–/–
CASP1
P45
P20
Actin
b
WT mice
Survival (%)
Days post-infection
****
ns
F WT (n = 12)
F T27 (n = 16)
F T27 Rev (n = 14)
c
Aim2–/– mice
Survival (%)
Days post-infection
ns
F WT (n = 8)
F T27 (n = 11)
F T27 Rev (n = 11)
d
WT F
F T27
WT
Aim2–/–
CASP1
P45
P20
Actin
e
F T27
F T27 Rev
WT
Aim2–/–
CASP1
P45
P20
Actin

Supplementary Figure S8. ***In vitro*** **and** ***in vivo*** **analysis of caspase-1 activation and survival following infection with recombinant HSV-1 F strains.**

a, Immunoblot analysis of pro-caspase-1 (CASP1; P45) and cleaved CASP1 (P20) in wild-type (WT) or *Aim2*$^{-/-}$ bone marrow-derived macrophages (BMDMs) after infection with F strain (WT, T_{27}, T_{27}-Rev). Expected molecular weights: pro-caspase-1 (P45, ~45 kDa) and cleaved caspase-1 (P20, ~20 kDa). Data are representative of three independent experiments. **b, c,** Survival of WT (**b**) or *Aim2*$^{-/-}$ mice (**c**) infected intranasally with 5×10^5 plaque-forming units (PFU) with HSV-1 of F strain (WT, T_{27}, T_{27}-Rev). 'ns', not significant (WT F versus F T_{27}-Rev in WT mice, WT F versus F T_{27} or F T_{27}-Rev in *Aim2*$^{-/-}$ mice); ****$P < 0.0001$ (WT F versus F T_{27} in WT mice), log-rank test (Mantel–Cox). Survival data are pooled from two independent experiments. **d, e,** Immunoblot analysis of pro- (P45) and activated (P20) caspase-1 (CASP1) in lung tissue from WT or *Aim2*$^{-/-}$ mice 5 days after infection with WT F and F T_{27} HSV-1 (**d**), or F T_{27} and F T_{27}-Rev (**e**). Expected molecular weights: pro-caspase-1 (P45, ~45 kDa) and cleaved caspase-1 (P20, ~20 kDa). Each lane indicates independent biological replicates. Data are representative of three independent experiments. Source data are provided as a Source Data file.

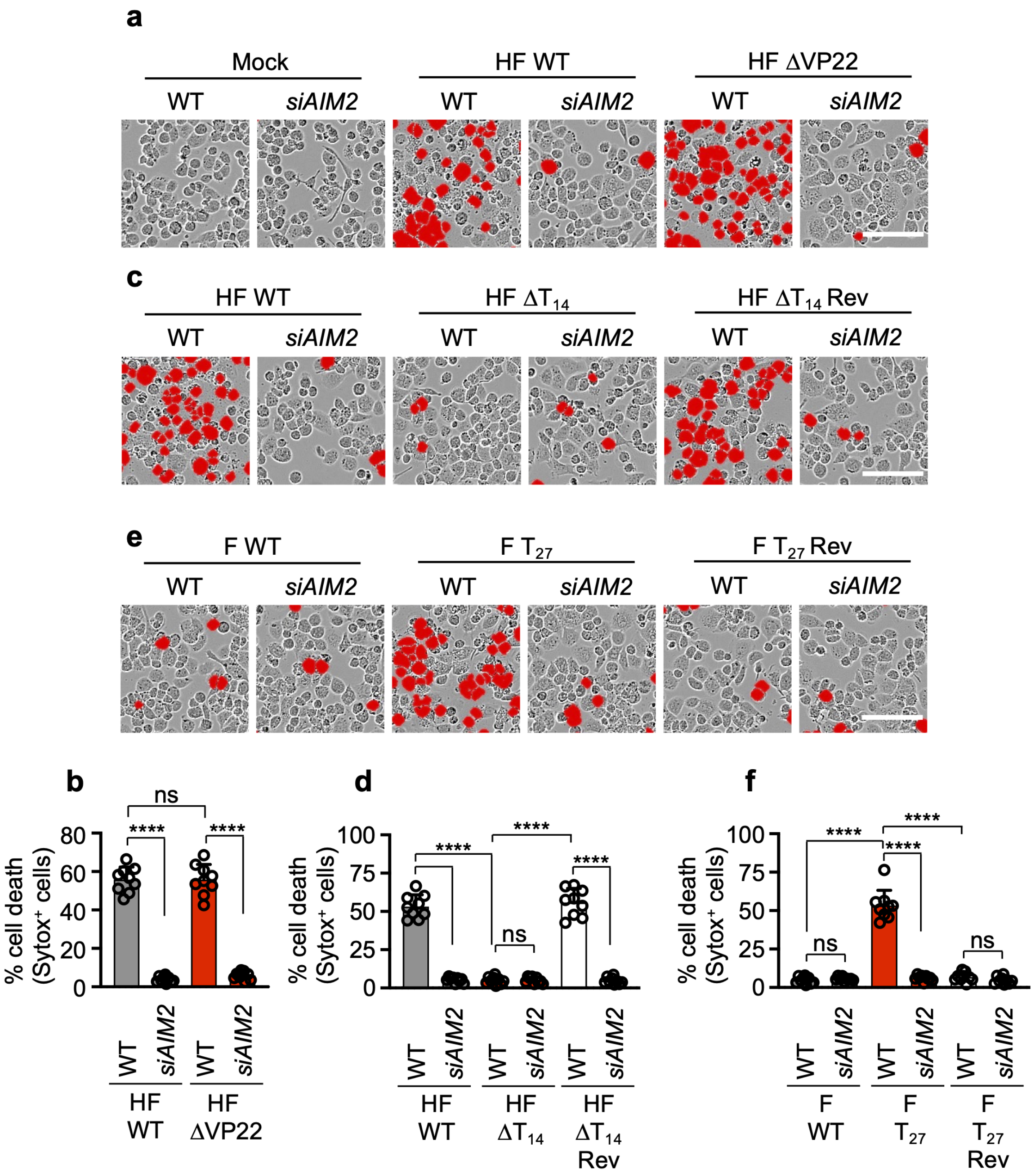
a
Mock
HF WT
HF ΔVP22
WT
siAIM2
c
HF WT
HF ΔT14
HF ΔT14 Rev
e
F WT
F T27
F T27 Rev
b
d
f
% cell death (Sytox+ cells)
ns
****
HF WT
HF ΔVP22
HF ΔT14 Rev
F T27 Rev

Supplementary Figure S9. AIM2-dependent regulation of cell death in human THP-1 macrophages.

a, Cell death in THP-1 macrophages treated with control siRNA (Control) or siRNA targeted to AIM2 (siAIM2) after infection with WT HF or HF ΔVP22 HSV-1. **b,** Quantification of cell death in (**a**). **c,** Cell death in THP-1 macrophages treated with control siRNA (Control) or siRNA targeted to AIM2 (siAIM2) after infection with WT, HF ΔT_{14}, or HF ΔT_{14}-Rev. **d,** Quantification of cell death in (**c**). **e,** Cell death in THP-1 macrophages treated with control siRNA (Control) or siRNA targeted to AIM2 (siAIM2) after infection with F strain (WT, T_{27}, T_{27}-Rev). **f,** Quantification of cell death in (**e**). Panels **a, c, e** show images representative of three biologically independent experiments. Dead cells are indicated in red. Scale bar: 100 μm. **b, d, f** depict data as mean ± s.e.m. 'ns', not significant; ****P < 0.0001 (one-way ANOVA with Dunnett's multiple comparisons test; n = 9 biologically independent samples from three independent experiments). Source data are provided as a Source Data file.

a

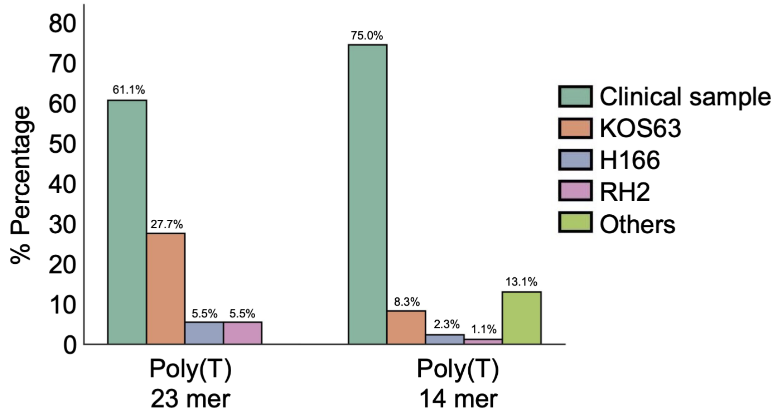

% Percentage
80
70
60
50
40
30
20
10
0
61.1%
27.7%
5.5%
5.5%
75.0%
8.3%
2.3%
1.1%
13.1%
Poly(T) 23 mer
Poly(T) 14 mer
Clinical sample
KOS63
H166
RH2
Others

b

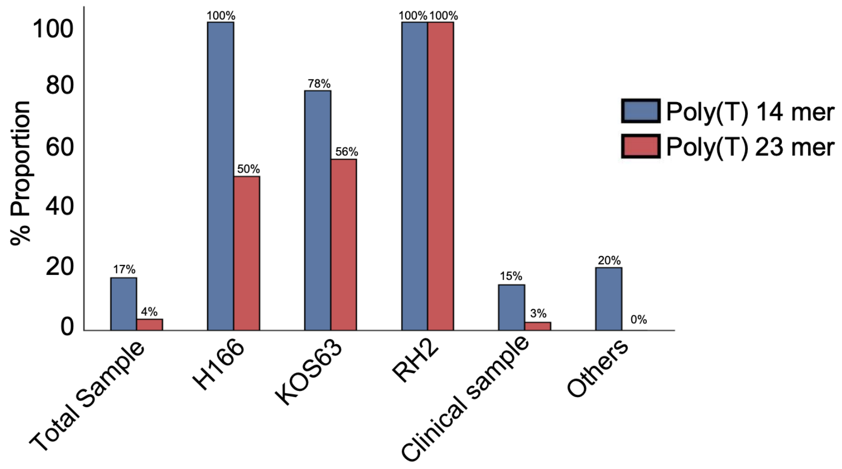

% Proportion
100
80
60
40
20
0
17%
4%
100%
50%
78%
56%
100%
100%
15%
3%
20%
0%
Total Sample
H166
KOS63
RH2
Clinical sample
Others
Poly(T) 14 mer
Poly(T) 23 mer

Supplementary Figure S10. Distribution of poly(T) DNA sequences across laboratory and clinical HSV-1 genomes.

Analysis of the 492 complete HSV-1 genome sequences obtained from the NCBI database. **a,** Percentage of H166, KOS63, ZW63, other strains and clinical samples in 23-mer poly(T) positive (left; total *n* = 18) and 14-mer poly(T) positive (right; total *n* = 84) HSV-1 genomes. The 23-mer poly(T) tract is identified in 18 HSV-1 genomes, including well-characterized laboratory strains (e.g., H166, KOS63, ZW63) as well as multiple clinical isolates reported by independent sequencing studies. These data indicate that the 23-mer poly(T) sequence is present in both laboratory strains and clinical isolates and is consistent with naturally occurring variation in HSV-1 populations. **b,** Relative proportion of 14-mer positive (blue) and 23-mer positive (red) poly(T) HSV-1 genomes in total samples (*n* = 492), H166, KOS63, RH2, clinical samples, and other strains. Among 426 clinical HSV-1 genomes analyzed, a 14-mer poly(T) sequence was detected in approximately 15% of samples (63/426), whereas a 23-mer poly(T) sequence was present in 3% of samples (11/426). This distribution supports poly(T) length variability as a genomic polymorphism observed across HSV-1 strains and is consistent with variation present in circulating viral populations. Source data are provided as a Source Data file.

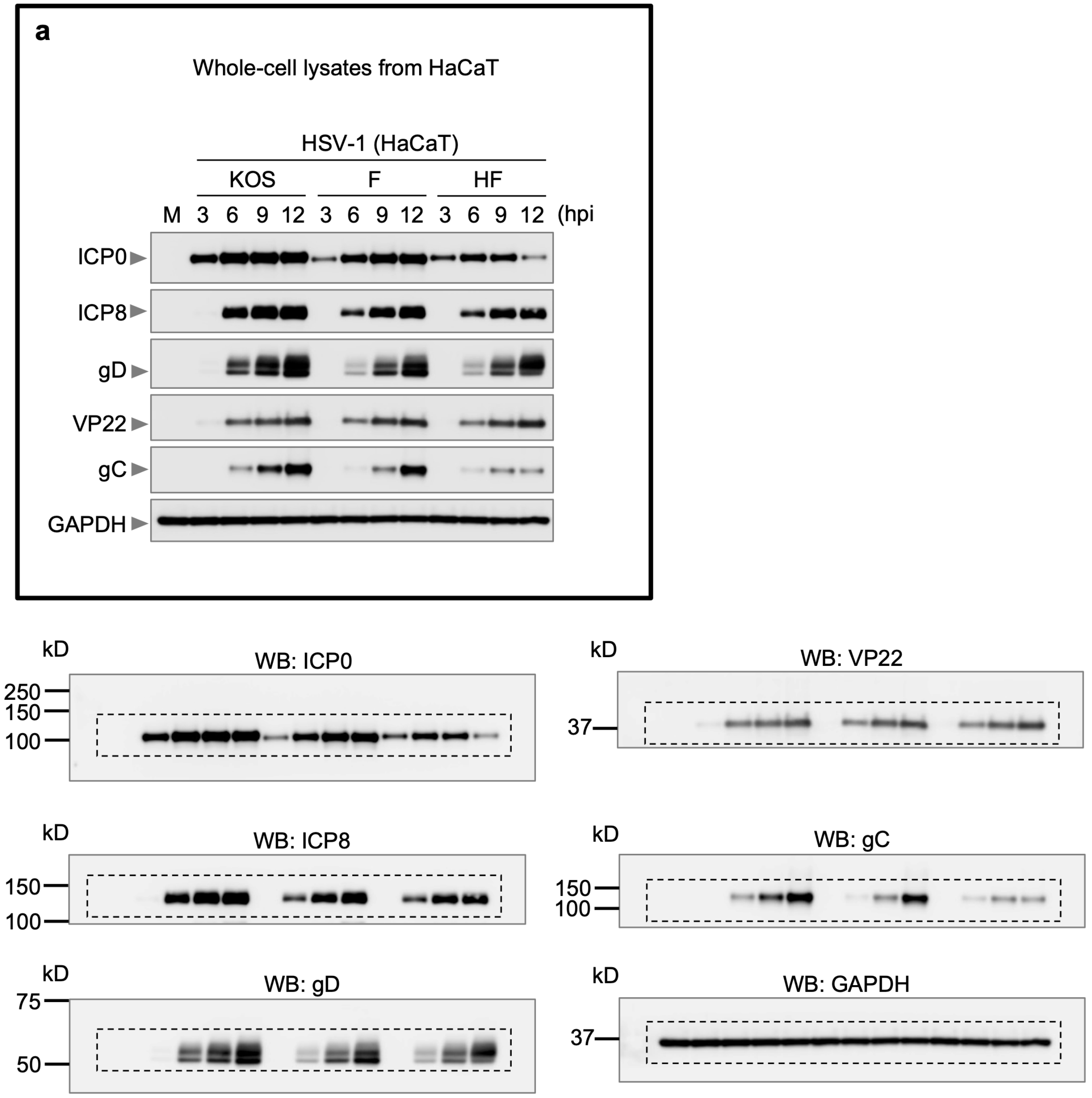

Supplementary Figure S1

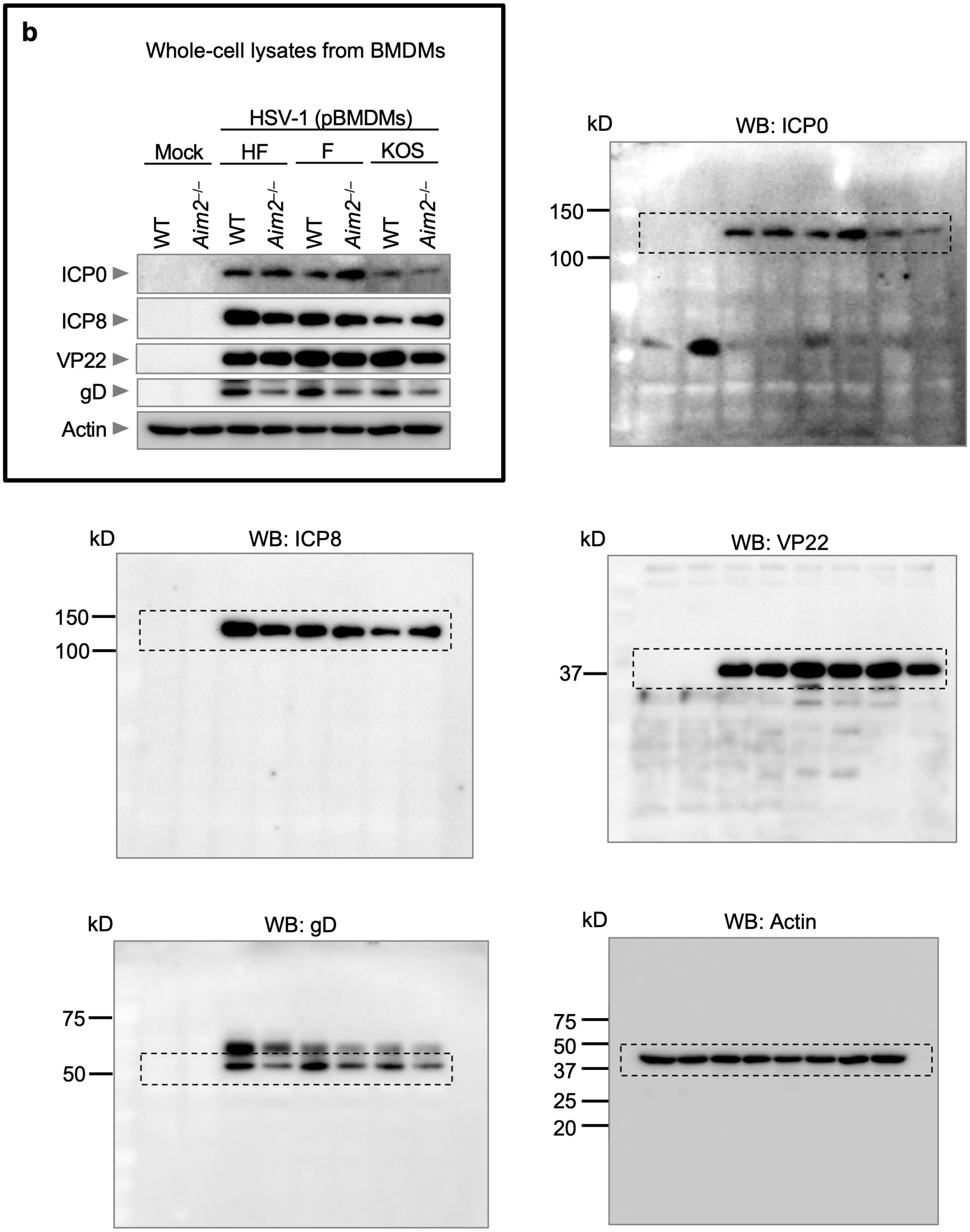

Supplementary Figure S1

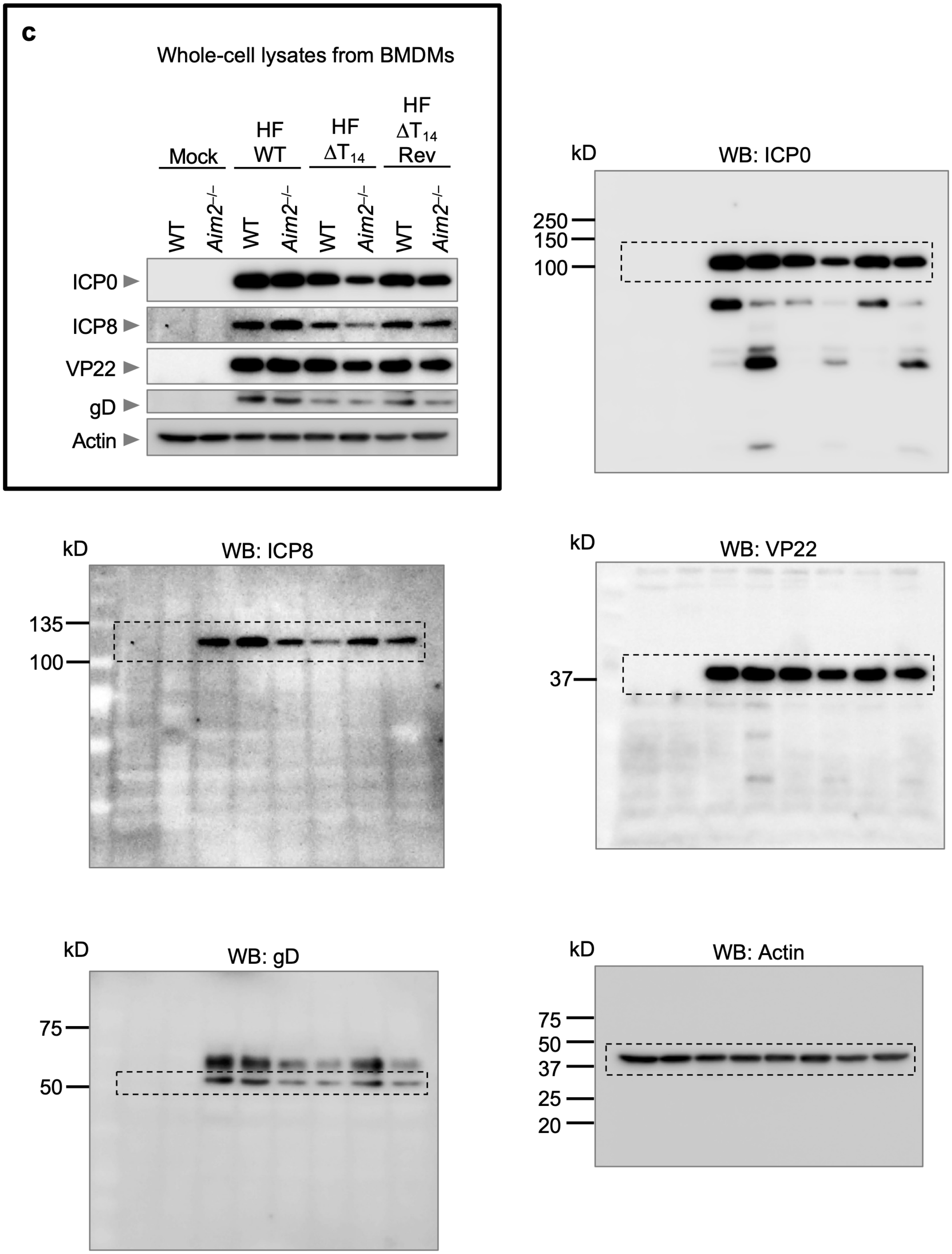

Supplementary Figure S1

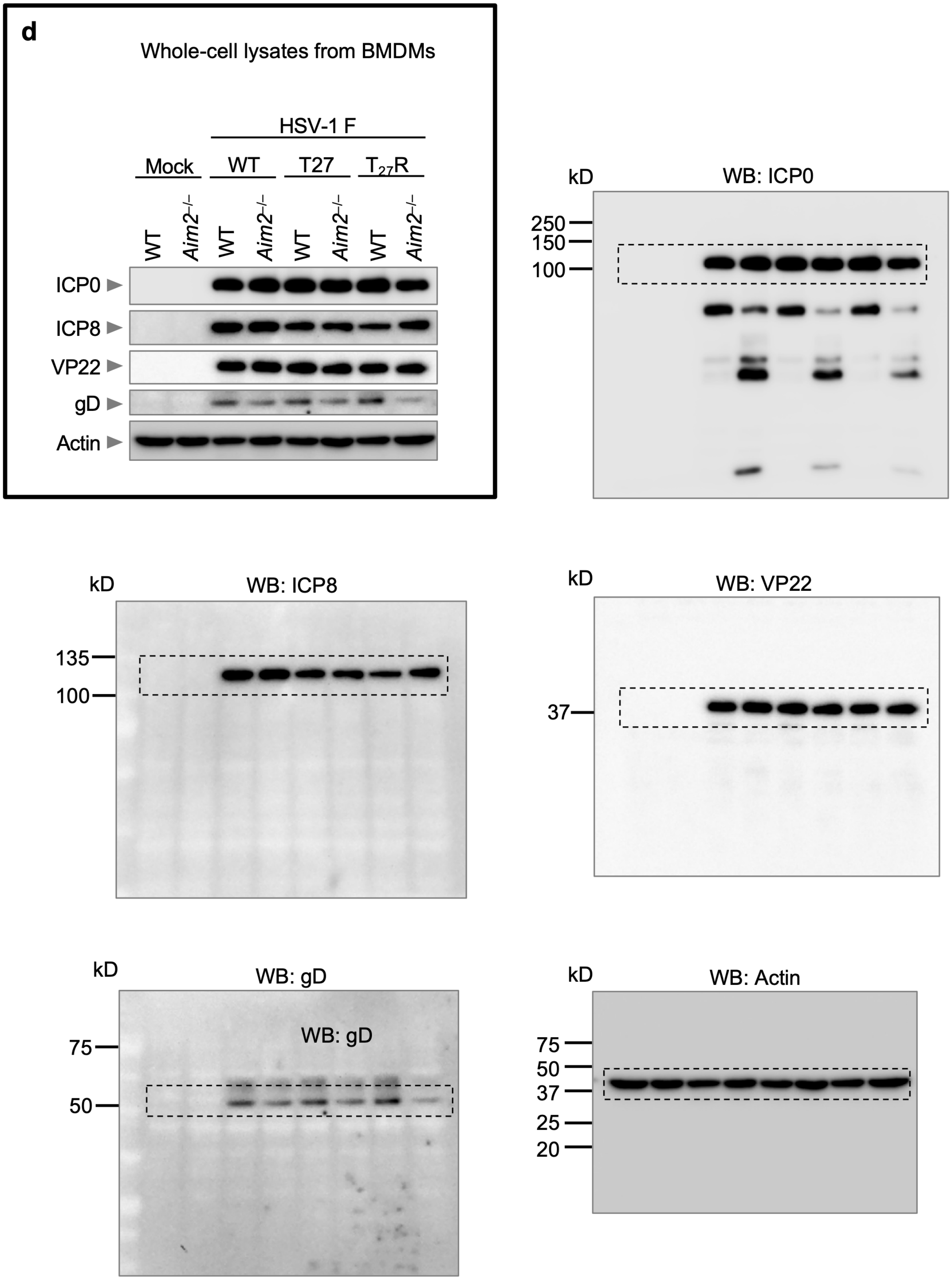

Supplementary Figure S1

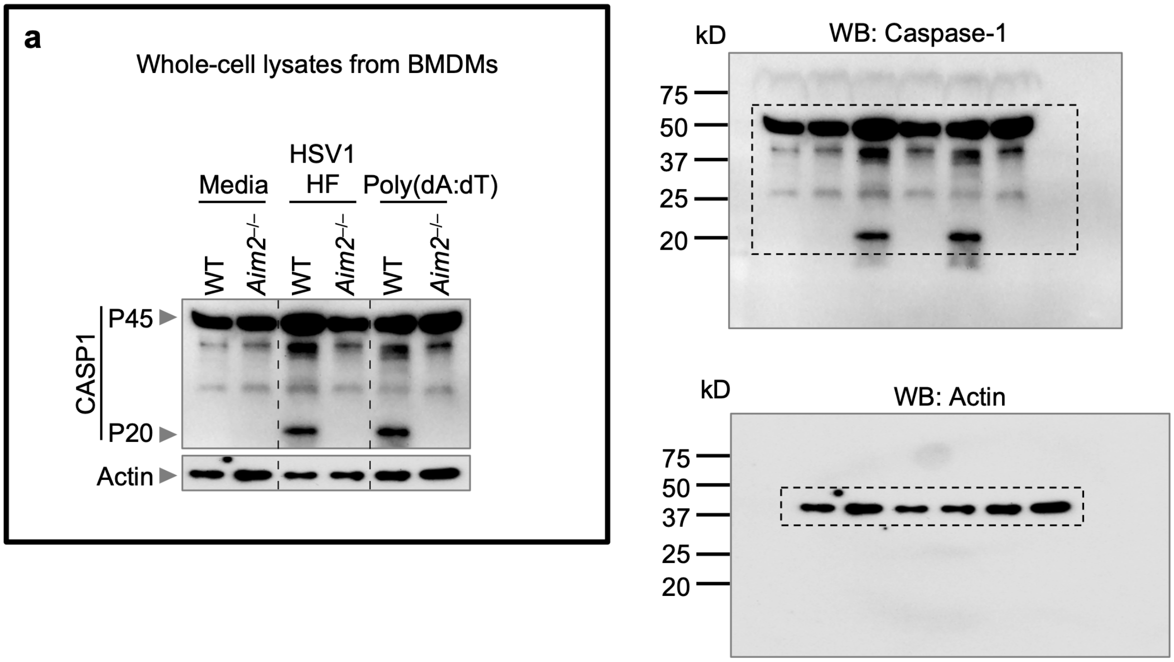

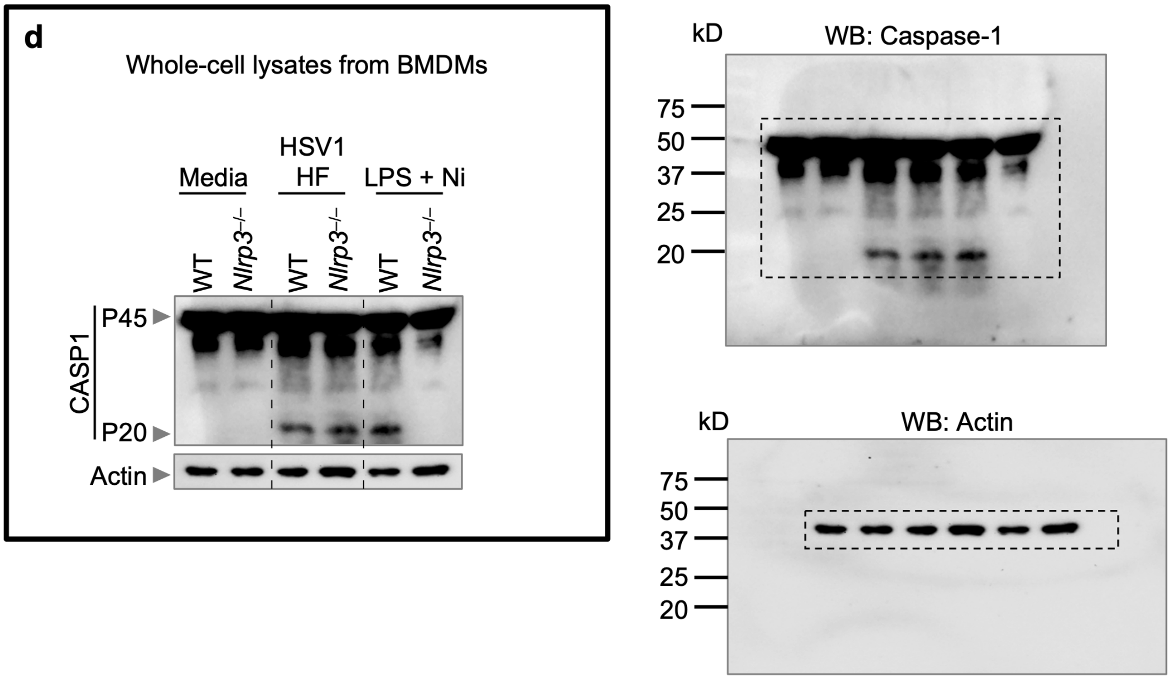

Supplementary Figure S2

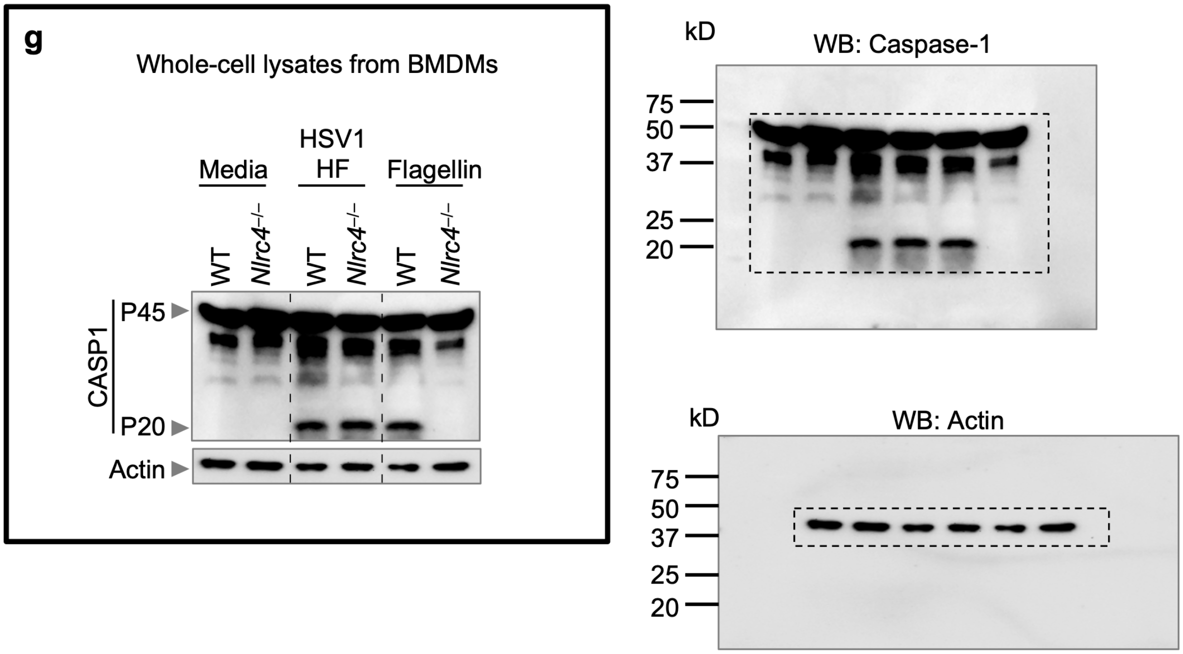

Supplementary Figure S2

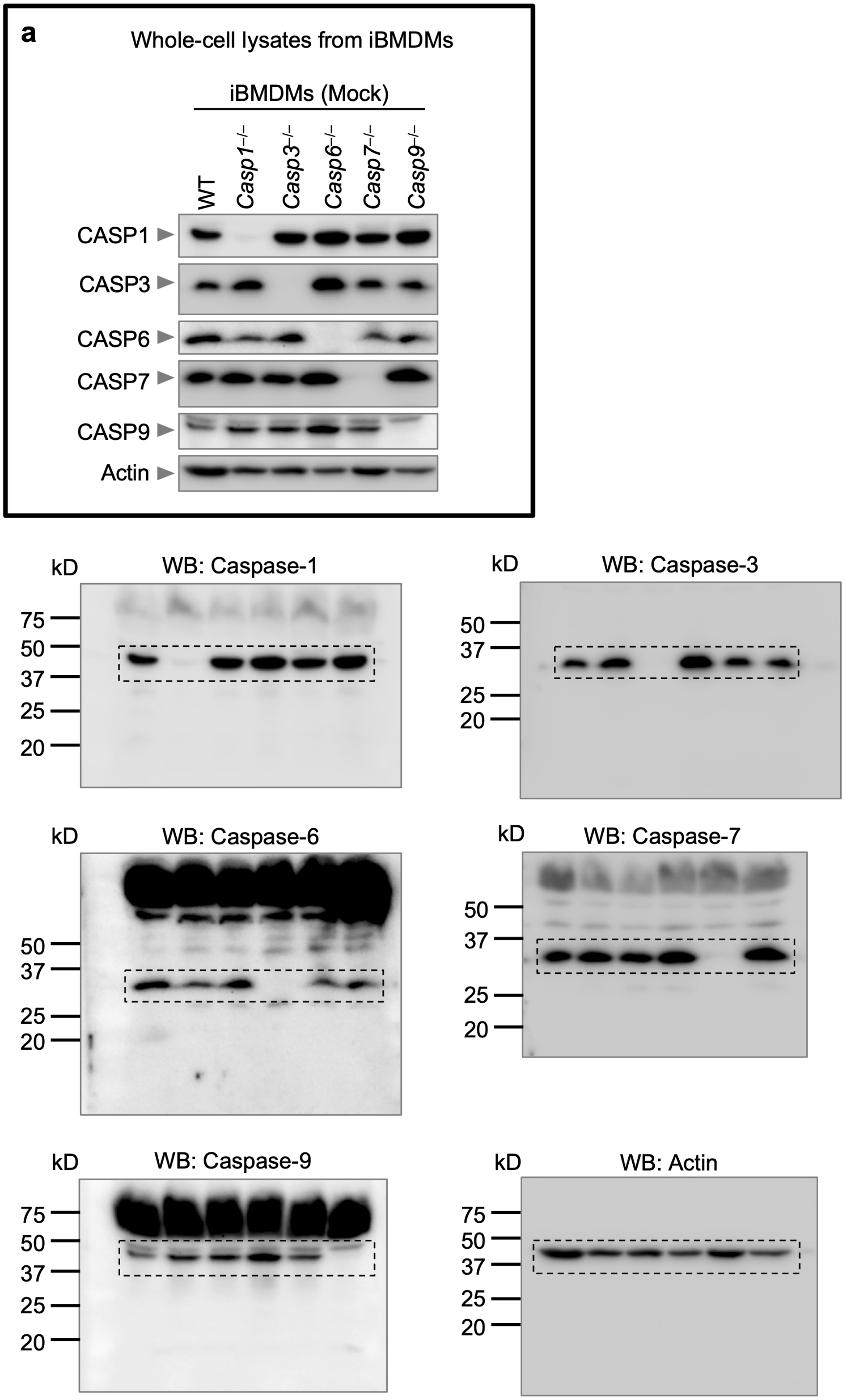

Supplementary Figure S3

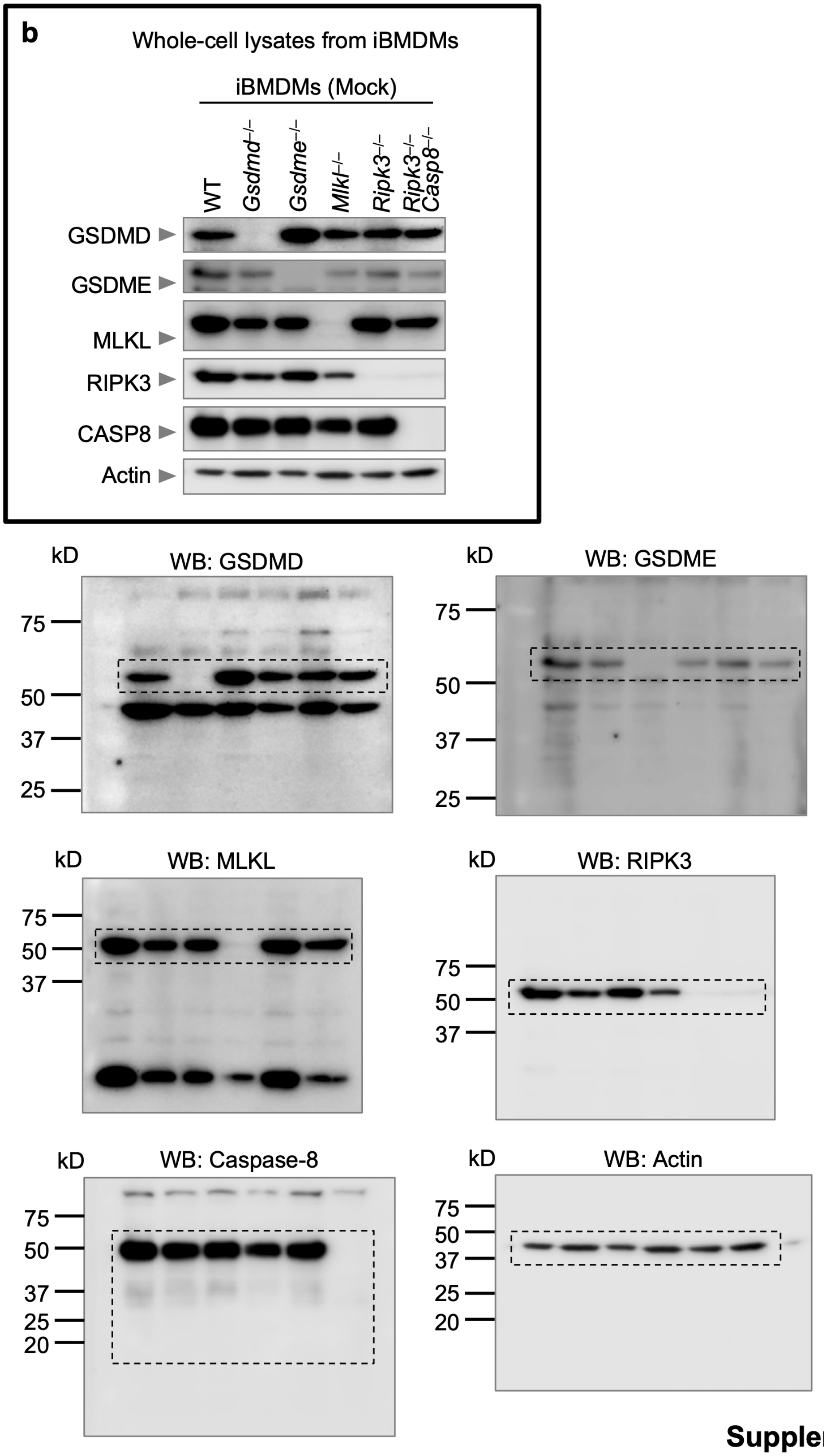

Supplementary Figure S3

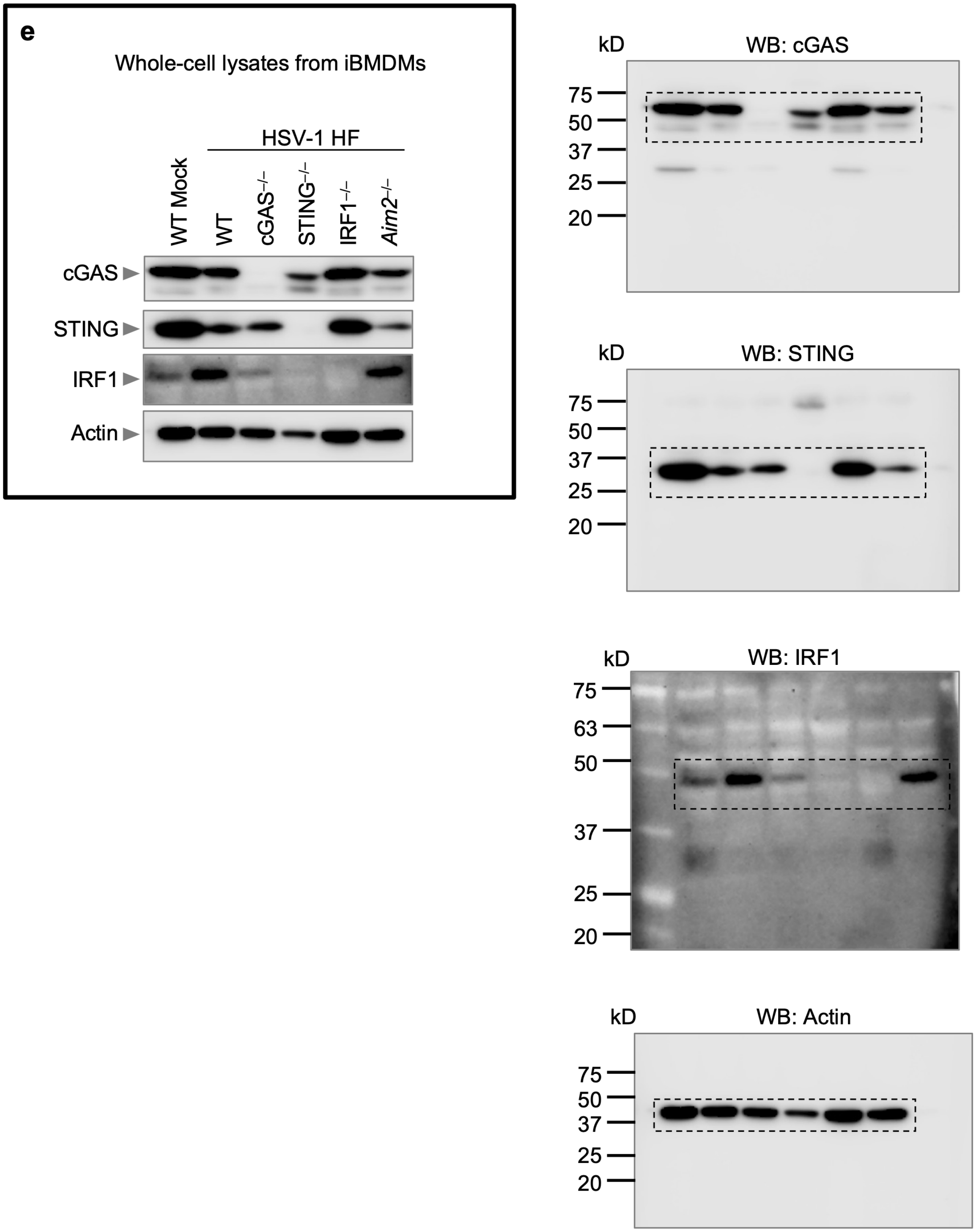

Supplementary Figure S4

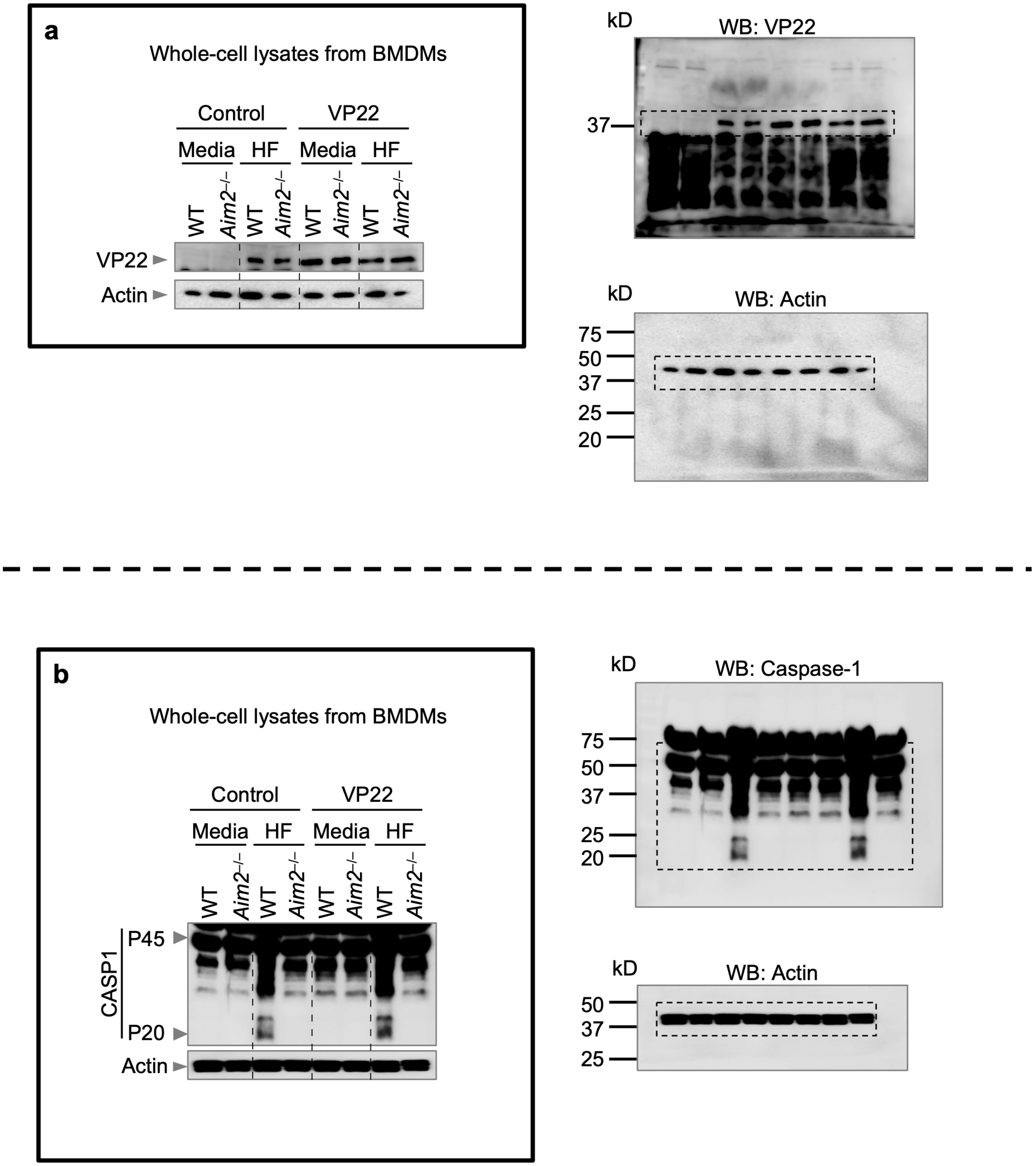

Supplementary Figure S5

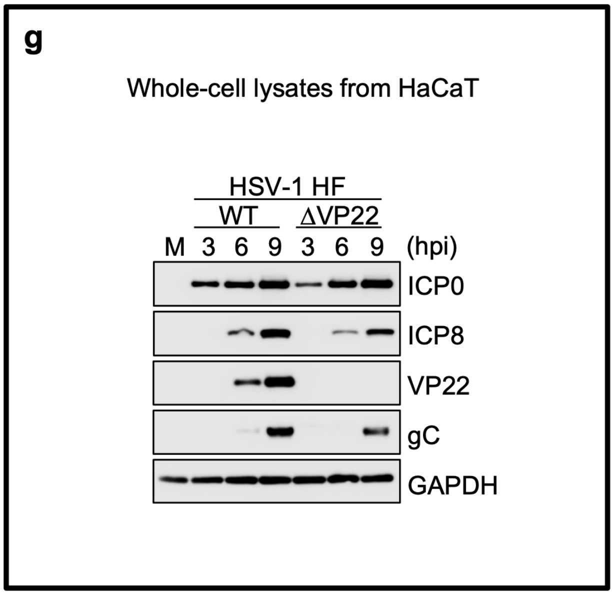

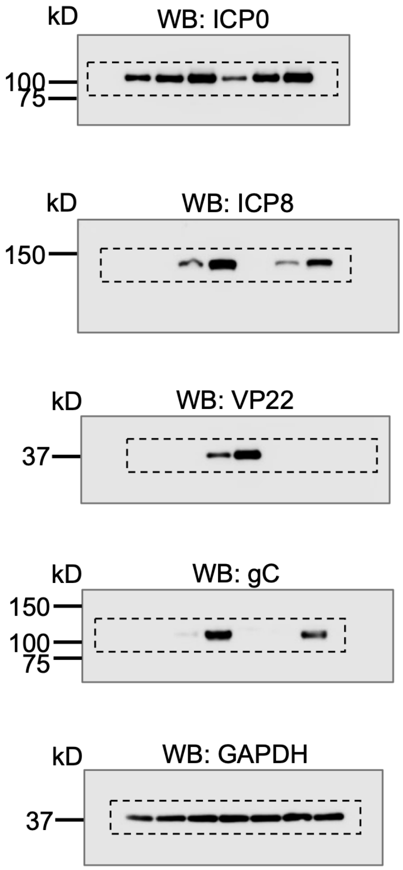

Supplementary Figure S5

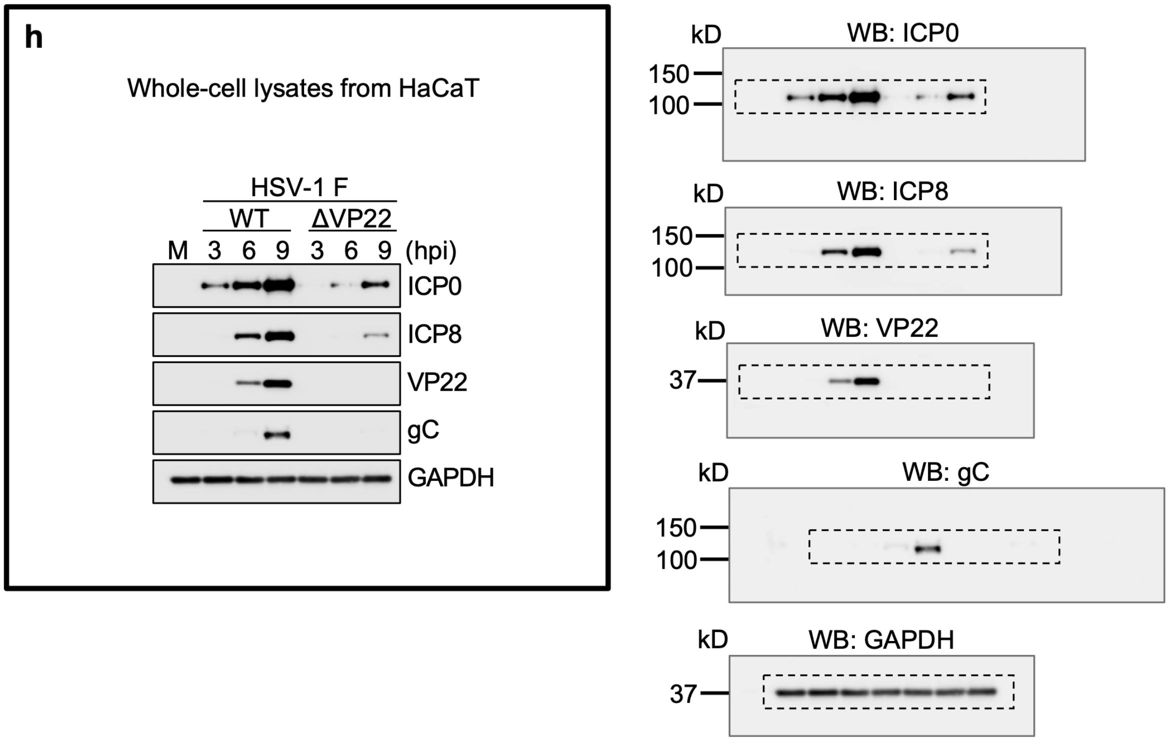

Supplementary Figure S5

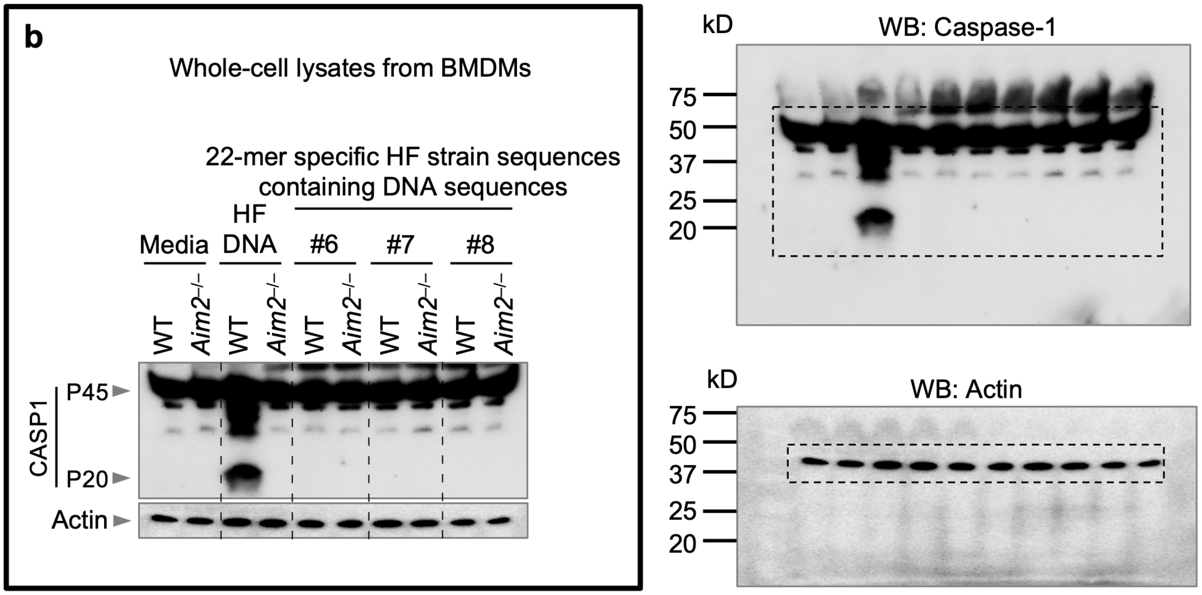

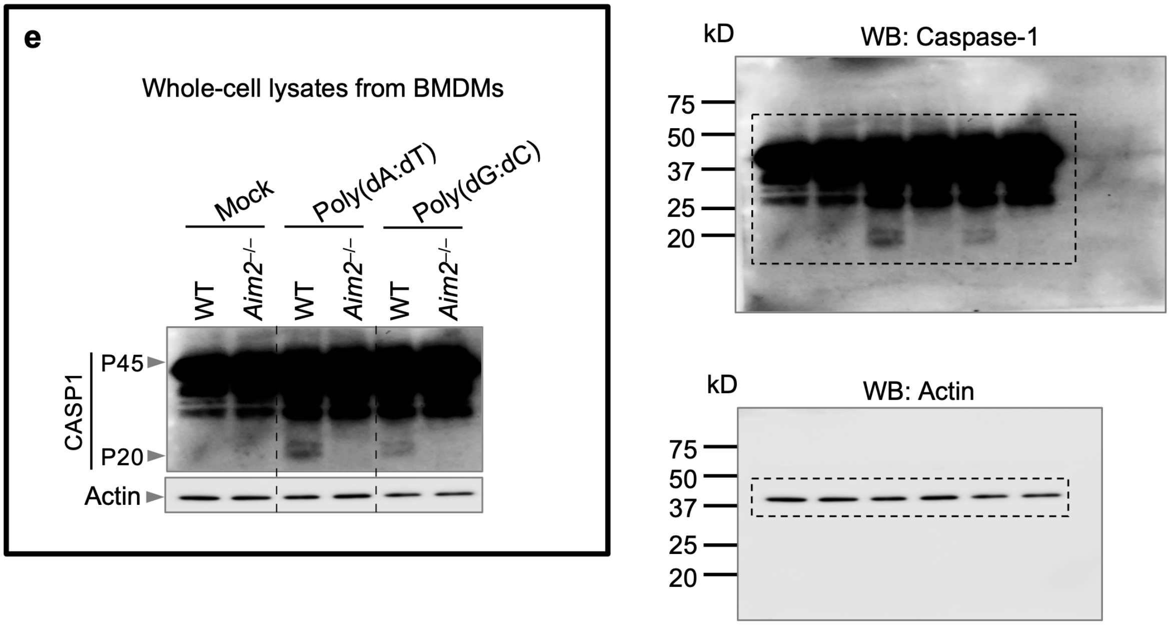

Supplementary Figure S6

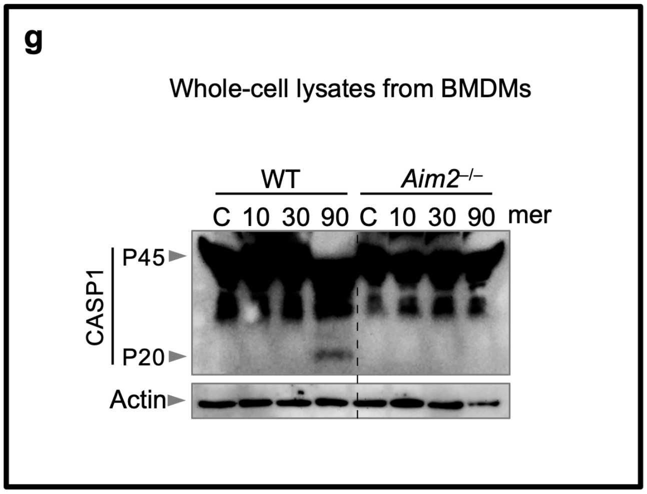

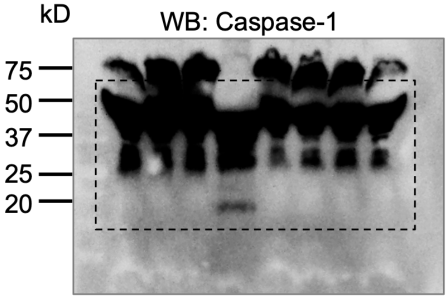

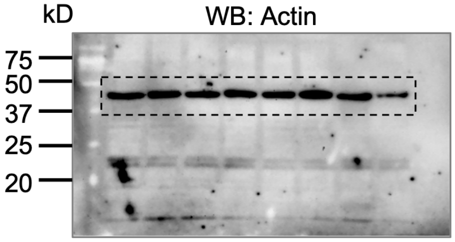

Supplementary Figure S6

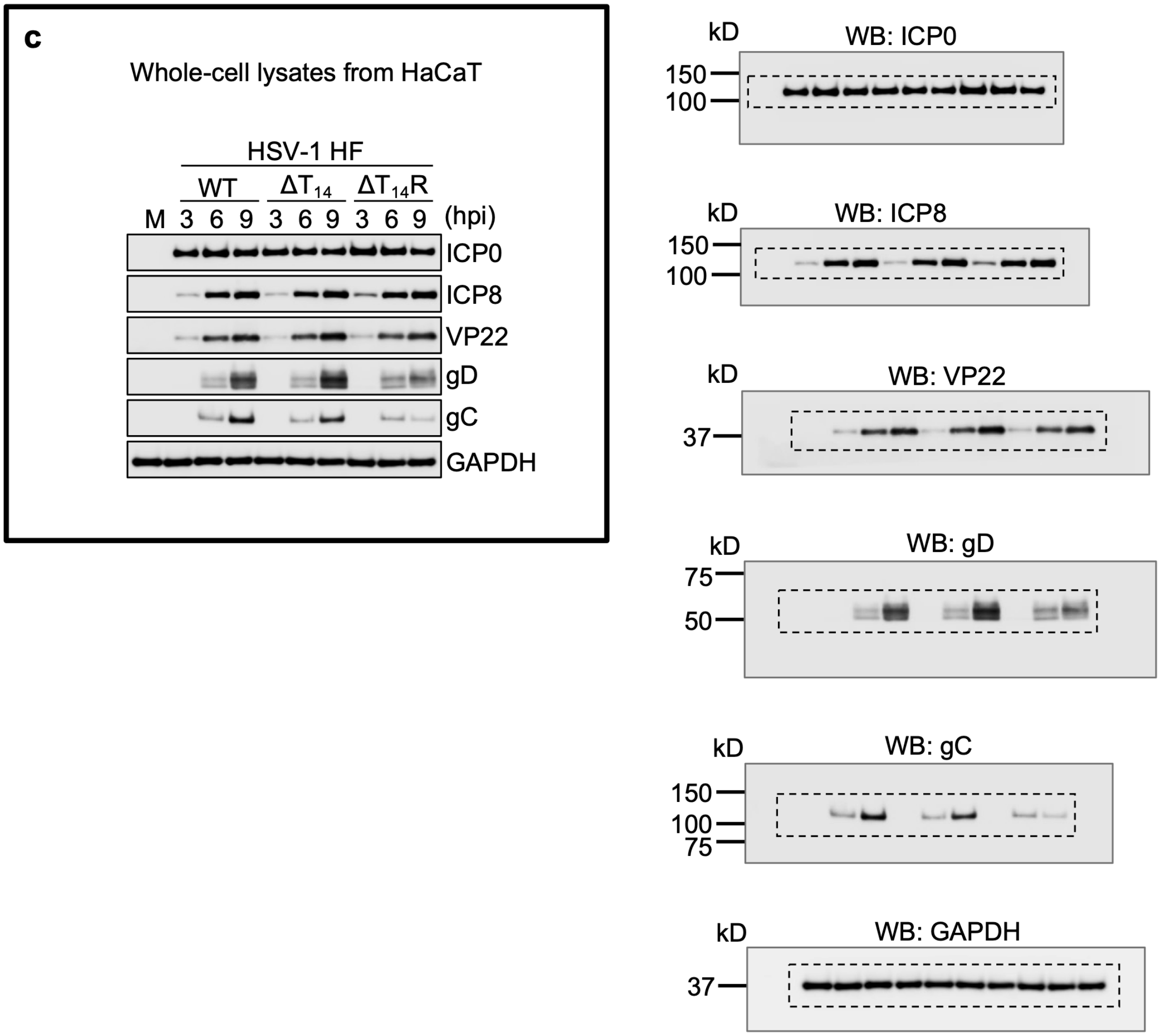

Supplementary Figure S7

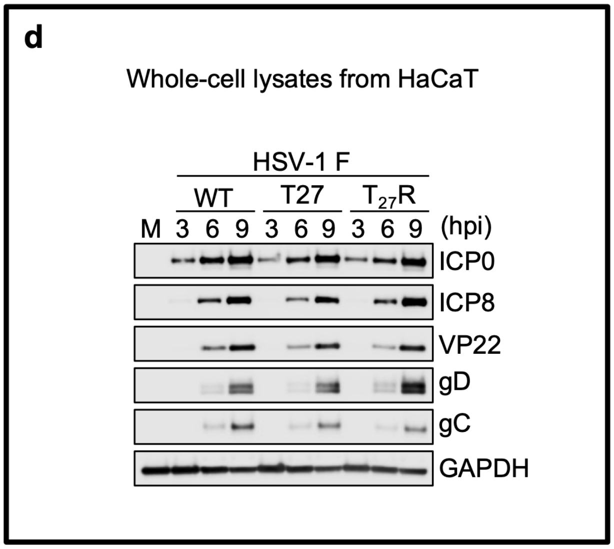

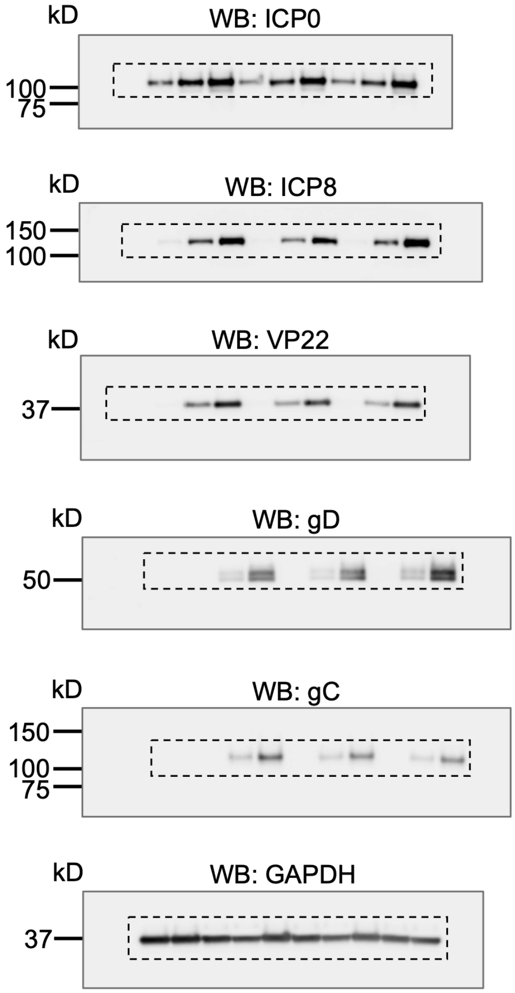

Supplementary Figure S7

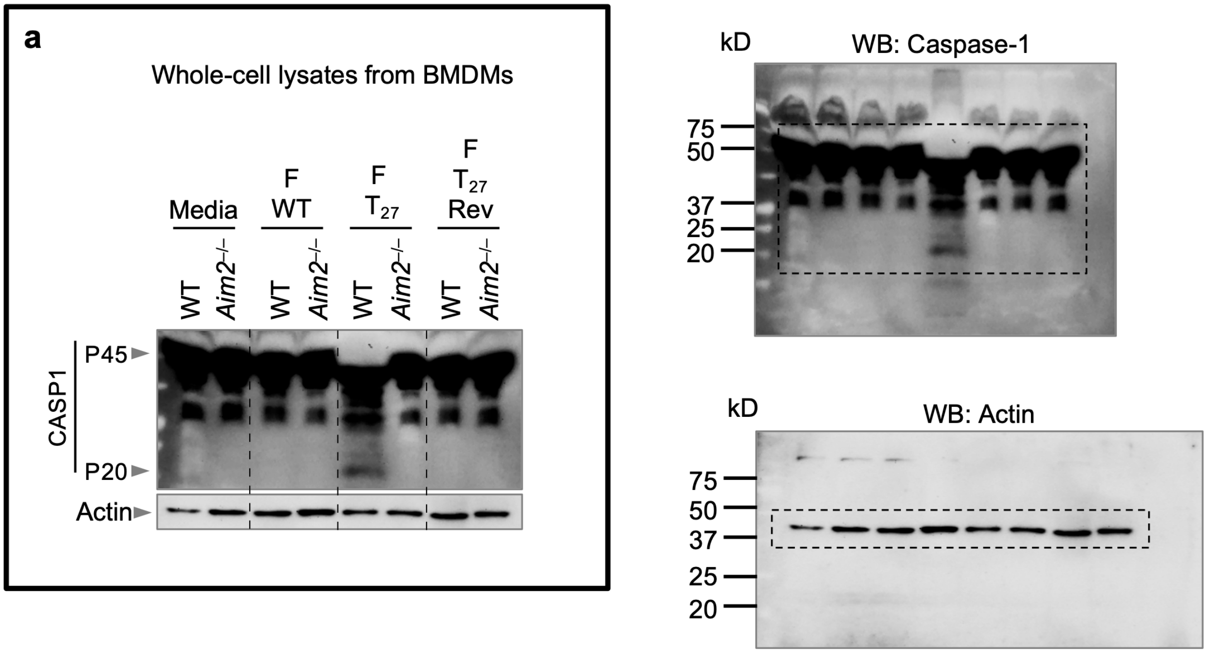

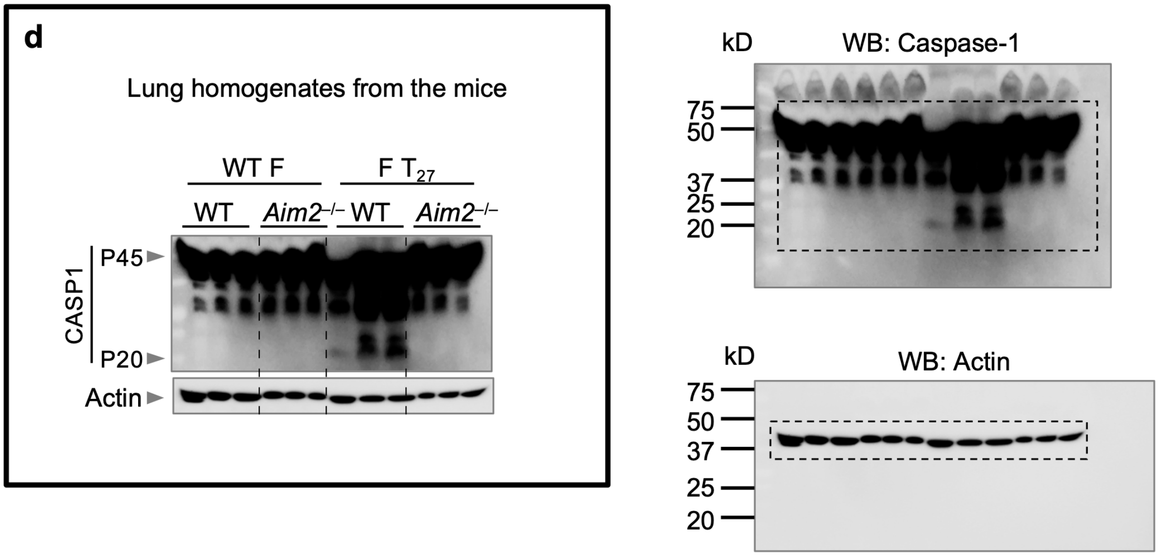

Supplementary Figure S8

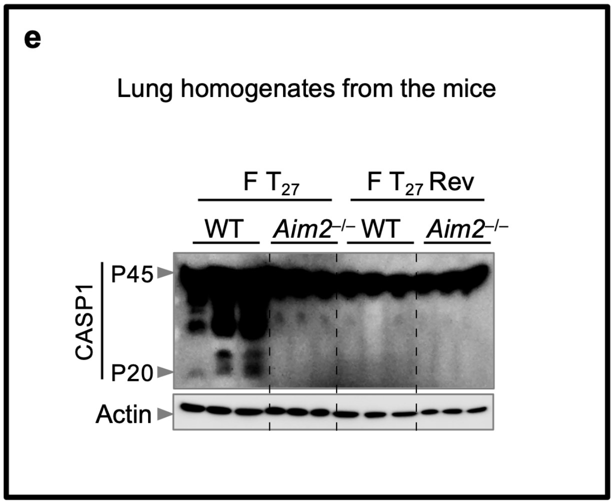

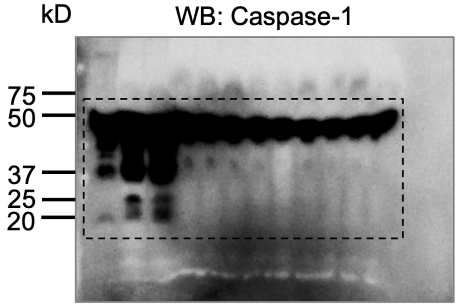

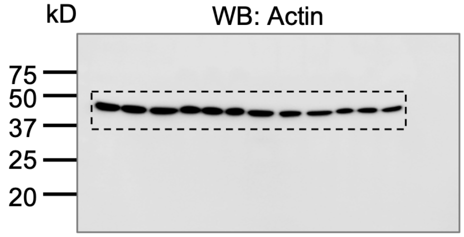

Supplementary Figure S8
