# Supplementary material for: Herpes simplex virus 1 harboring poly(T) DNA sequences as a key ligand for AIM2 inflammasome activation and host defense
Source: Nat Commun. 2026 Apr 13;17:5161. doi: 10.1038/s41467-026-71896-w (PMC13250049; doi:10.1038/s41467-026-71896-w)
Supplement: Supplementary file 2 — Description of additional supplementary files [file 41467_2026_71896_MOESM2_ESM.pdf]

Description of additional supplementary files

Supplementary Data 1

Title: Identification of RNA viruses harboring poly(T) sequences of defined lengths ($T_{(23)}$ and $T_{(14)}$) from the NCBI public virus database

Description: This file contains the results of large-scale screening of RNA virus genomes from the NCBI public virus database to identify poly(T) sequences of 23-mer ($T_{(23)}$) and 14-mer ($T_{(14)}$) lengths. A total of 12,738,910 entries were initially collected, of which 2,069,973 duplicate entries were removed, resulting in 10,668,937 unique viral genome sequences included in the final analysis.

Supplementary Data 2

Title: Identification of DNA viruses harboring poly(T) sequences of defined lengths ($T_{(23)}$ and $T_{(14)}$) from the NCBI public virus database

Description: This file provides the results of genome-wide analysis of DNA viruses from the NCBI public virus database to identify poly(T) sequences of 23-mer ($T_{(23)}$) and 14-mer ($T_{(14)}$) lengths. A total of 285,390 entries were initially retrieved, of which 33,578 duplicate entries were excluded, yielding 251,812 unique viral genome sequences for downstream analysis.

Supplementary Data 3

Title: Validation of gene knockout cell lines by sequence analysis

Description: This file provides sequence validation data for CRISPR/Cas9-generated knockout cell lines, including indel mutations across target genes such as Caspases, Gsdme, Ripk3, AIM2, cGAS, IRF1, STING, MYD88, and TRIF.
