# Supplementary material for: Herpes simplex virus 1 harboring poly(T) DNA sequences as a key ligand for AIM2 inflammasome activation and host defense
Source: Nat Commun. 2026 Apr 13;17:5161. doi: 10.1038/s41467-026-71896-w (PMC13250049; doi:10.1038/s41467-026-71896-w)
Supplement: Supplementary file 5 — Supplementary Data 3 [file 41467_2026_71896_MOESM5_ESM.pdf]

Casp1$^{-/-}$

GAGGAAGCAATTTATCAACTCAGTGAGTATAGGGACAATAAATGGATTGTTGGATGAACTTTTAGAGAAGAGAGTGCTGAATCAGGAA Reference
GAGGAAGCAATTT---TAGAGAAGAGAGTGCTGAATCAGGAA -49 bp

Casp3$^{-/-}$

GTTGACTAGCTTCTTCAGAGGCGACTACTGCCGGAGTCTGACTGGAAAGCCGAAACTCTTCATCATTCAGGTAGGGCATTGCCCAGCA Reference
GTTGA---CCAGCA -77 bp

Casp6$^{-/-}$

TGCGAGTCAGGTTGTCTCTGTCGCGTTGGTGCCCCGCCTCTCTGGGAGGGTCAGGTGCCAAAAGAACCTCTCGTGATTGAAGATGAGG Reference
TGCGAGTCAGGTTGTCTCTGTCGCGTTGGTGCCCCGC----CTGGGAGGGTCAGGTGCCAAAAGAACCTCTCGTGATTGAAGATGAGG -4 bp

Casp7$^{-/-}$

CGGTACAGATAAGTGGCACTCGGTCCCGGCCGGTCCTGACGGGGCCCGCAGAGGCATTTCTCTTCTTCTTCCTTGGGTTACAGAAAG Reference
CGGTACAGATAAGTGGCACTCGGTCCCGGCCGGTCCT-ACGGGGCCCGCAGAGGCATTTCTCTTCTTCTTCCTTGGGTTACAGAAAG -1 bp

Casp9$^{-/-}$

CACATGAGGACTCACCATATCTGCATGTCCCCTGATCTTCCCTGGAACACCTGAGAAAACAGGAGAAAGAAGAAATGTGGGTGTTGAG Reference
CACATGAGGACTCACCATATCTGCATGTCCCCTGATCT-CCCTGGAACACCTGAGAAAACAGGAGAAAGAAGAAATGTGGGTGTTGAG -1 bp

Gsdme$^{-/-}$

AAGTGTGAGAACCATAAGAGCGGGGCTATTGGGACAGTC-GTGGGGAAGGTCAAGCTGAACGTTGGTGGCAAAGGCGTGGTGGAGAGT Reference
AAGTGTGAGAACCATAAGAGCGGGGCTATTGGGAC------------AGGTCAAGCTGAACGTTGGTGGCAAAGGCGTGGTGGAGAGT -11 bp
AAGTGTGAGAACCATAAGAGCGGGGCTATTGGGACAGTCCGTGGGGAAGGTCAAGCTGAACGTTGGTGGCAAAGGCGTGGTGGAGAGT +1 bp

Tmlkl$^{-/-}$

TCTGCTGGTTAGCCTCCTTCAGGACTTCATCAAAACGGCCCAGGGCAGCAGTAATGTCATCGGGCAGGTTCTTCTTTCCTTGGGCCTG Reference
TCTATCTGCTGGTTAGCCTCCT--------------------------------------TCATCGGGCAGGTTCTTCTTTCCTTGGG -38 bp
TCTGCTGGTTAGCCTCCTTCAGGACTTCATCAAAACGG-CCAGGGCAGCAGTAATGTCATCGGGCAGGTTCTTCTTTCCTTGGGCCTG -1 bp

Ripk3$^{-/-}$

GAAGAACCTTTCTCAGGCCTACTGGTGCGTCAGCGGTTCCTCTGGTGAGCCGTGAAGAACTGAAGAAGCTGGAGTTTGTGGGTAAAGG Reference
GAAGAACCTTTCTCAGGCCTACTGGTGCGTCAGCGGTT-CTCTGGTGAGCCGTGAAGAACTGAAGAAGCTGGAGTTTGTGGGTAAAGG -1 bp

Ripk3$^{-/-}$Caps8$^{-/-}$

RIPK3

GAGCAAGAGAGAATGAAGAATGGGTACCTGTCATTGGATTCGGTGGGTCCAGGGATACCAAGGAGTGCCGTGTCTTCCATCTCCCTGC Reference
GAGCAAGAGAGAATGAAGAATGGG------------------------------ATACCAAGGAGTGCCGTGTCTTCCATCTCCCTGC -30 bp

Casp8

CGGAATATAGTTCTTGTGAGATGATGAATCCACTTCTAAAGTGTGGTTCTGTTGCTCGAAGCCTGCCTCATCAGGCACTCCTTTCTGG Reference
CGGAATATAGTTCTTGTGAGATGATGAATCCACTTCTAA-GTGTGGTTCTGTTGCTCGAAGCCTGCCTCATCAGGCACTCCTTTCTGG -1 bp
CGGAATATAGTTCTTGTGAGATGATGAATCCACTT-----GTGTGGTTCTGTTGCTCGAAGCCTGCCTCATCAGGCACTCCTTTCTGG -5 bp

AIM2$^{-/-}$

CTTTTCAGGCTGATCCTGGGACTGTGAGATGGAGAGTGAGTACCGGGAAATGCTGTTGTTGACCGGCCTGGACCACATCACGGAGGAAGA Reference
CTTTTCAGGCTGATCCTGGGACTGTGAGATGGAGAGTG----CCGGGAAATGCTGTTGTTGACCGGCCTGGACCACATCACGGAGGAAGA -4 bp
CTTTTCAGGCTGATCCTGGGACTGTGAGATGGAGAGTGAG-ACCGGGAAATGCTGTTGTTGACCGGCCTGGACCACATCACGGAGGAAGA -1 bp

cGAS$^{-/-}$

GCCATCTTTCACATGTGGACCCAGGACCCGCAGGACAGTCAGTGGGACCCCAGGAACCTCAGCTCCTGCTTCGATAAGTTGTTAGCATTC Reference
GCCATCTTTCACATGTGGACCCAGGACCCGCAGGACAA--AGTGGGACCCCAGGAACCTCAGCTCCTGCTTCGATAAGTTGTTAGCATTC -2bp
GCCATCTTTCACATGTGGACCCAGGACCCGCAGGACAG--AGTGGGACCCCAGGAACCTCAGCTCCTGCTTCGATAAGTTGTTAGCATTC -2bp

IRF1$^{-/-}$

TAACACTCACTTTATTGATCCAGATCAGCCCTGGGATTTGGTTGGAATTAATCTGCATCTCTAGCCAGGGTCTCATCCGCATTCGAGTGA Reference
TAACACTCACTTTATTGATCCAGATCAGCCCTGGGATT-GGTTGGAATTAATCTGCATCTCTAGCCAGGGTCTCATCCGCATTCGAGTGA -1bp
TAACACTCACTTTATTGATCCAGATCAGCCCTGG-----GATTGGAATTAATCTGCATCTCTAGCCAGGGTCTCATCCGCATTCGAGTGA -5bp

STING$^{-/-}$

CACTCTGAAGTACCTAGCACTTCACCTAGCCTCGCACGA-ACTTGGACTACTGTTGAAAAACCTCTGCTGTCTGGCTGAAGAGCTGTGCC Reference
CACTCTGAAGTACCTAGCACTTCACCTAGCCTCGCACGAGACTTGGACTACTGTTGAAAAACCTCTGCTGTCTGGCTGAAGAGCTGTGCC +1bp

MYD88$^{-/-}$

TGGCAATGGACCAGACACAGGTGCCCGGCAGGACGTCACGGTCGGACACACACAACTTAAGCCGATAGTCTGTCTGTTCTAGTTGCCGGA Reference
TGGCAATGGACCAGACACAGGTGCCCGG--------------------------------GCCGATAGTCTGTCTGTTCTAGTTGCCGGA -32
TGGCAATGGACCAGACACAGGTGCCCGGCAGGACGTCA-GGTCGGACACACACAACTTAAGCCGATAGTCTGTCTGTTCTAGTTGCCGGA -1

TRIF$^{-/-}$

GGGAGGAAGCAGAGGACAGAGGGGAAGGAGGCAGCTTAGGGGAGGCTTGGAGGGATGGTGGCTGGGCAGGAGGGCTGGATGACGTGGTGT Reference
GGGAGGAAGCAGAGGACAGAGGGGAAGGAGGCAGC-----GGAGGCTTGGAGGGATGGTGGCTGGGCAGGAGGGCTGGATGACGTGGTGT -5
