# Supplementary material for: Herpes simplex virus 1 harboring poly(T) DNA sequences as a key ligand for AIM2 inflammasome activation and host defense
Source: Nat Commun. 2026 Apr 13;17:5161. doi: 10.1038/s41467-026-71896-w (PMC13250049; doi:10.1038/s41467-026-71896-w)
Supplement: Supplementary file 6 — Reporting summary [file 41467_2026_71896_MOESM6_ESM.pdf]

Corresponding author(s): SangJoon Lee

Last updated by author(s): Mar 26, 2026

Reporting Summary

Nature Portfolio wishes to improve the reproducibility of the work that we publish. This form provides structure for consistency and transparency in reporting. For further information on Nature Portfolio policies, see our Editorial Policies and the Editorial Policy Checklist.

Statistics

For all statistical analyses, confirm that the following items are present in the figure legend, table legend, main text, or Methods section.

n/a	Confirmed	
☐	☒	The exact sample size (*n*) for each experimental group/condition, given as a discrete number and unit of measurement
☐	☒	A statement on whether measurements were taken from distinct samples or whether the same sample was measured repeatedly
☐	☒	The statistical test(s) used AND whether they are one- or two-sided<br>*Only common tests should be described solely by name; describe more complex techniques in the Methods section.*
☒	☐	A description of all covariates tested
☒	☐	A description of any assumptions or corrections, such as tests of normality and adjustment for multiple comparisons
☐	☒	A full description of the statistical parameters including central tendency (e.g. means) or other basic estimates (e.g. regression coefficient) AND variation (e.g. standard deviation) or associated estimates of uncertainty (e.g. confidence intervals)
☐	☒	For null hypothesis testing, the test statistic (e.g. *F*, *t*, *r*) with confidence intervals, effect sizes, degrees of freedom and *P* value noted<br>*Give P values as exact values whenever suitable.*
☒	☐	For Bayesian analysis, information on the choice of priors and Markov chain Monte Carlo settings
☒	☐	For hierarchical and complex designs, identification of the appropriate level for tests and full reporting of outcomes
☒	☐	Estimates of effect sizes (e.g. Cohen's *d*, Pearson's *r*), indicating how they were calculated

Our web collection on statistics for biologists contains articles on many of the points above.

Software and code

Policy information about availability of computer code

Data collection	Microscopy images were collected using manufacturer supplied software (IncuCyte S3). Confocal microscopy samples were visualized and imaged using a Zeiss LSM 900 confocal microscope. For immunoblotting, membranes were developed with an Amersham imager.
Data analysis	For immunoblotting, images were analyzed with ImageJ. Graphpad Prism version 10 and IncuCyte S3 (v2022) were used for data analysis. Sequence alignment was performed using the Bio python package to compare the VP22 protein sequences of HSV-1 strains HF (GenBank Accession No. DQ889502), F (GenBank Accession No. GU734771), and KOS (GenBank Accession No. JQ673480). The three-dimensional structure of the VP22 protein was predicted using the SWISS-MODEL server.

For manuscripts utilizing custom algorithms or software that are central to the research but not yet described in published literature, software must be made available to editors and reviewers. We strongly encourage code deposition in a community repository (e.g. GitHub). See the Nature Portfolio guidelines for submitting code & software for further information.

Data

Policy information about availability of data

All manuscripts must include a data availability statement. This statement should provide the following information, where applicable:

- Accession codes, unique identifiers, or web links for publicly available datasets
- A description of any restrictions on data availability
- For clinical datasets or third party data, please ensure that the statement adheres to our policy

The datasets generated and analyzed during this study are available within the article, its Supplementary Information, and Supplementary Data files. Publicly available HSV-1 VP22 protein sequences were obtained from the NCBI database (GU734771.1 (HSV-1 strain F), DQ889502.1 (HSV-1 strain HF), and JQ673480 (HSV-1 strain KOS).

Research involving human participants, their data, or biological material

Policy information about studies with human participants or human data. See also policy information about sex, gender (identity/presentation), and sexual orientation and race, ethnicity and racism.

Reporting on sex and gender	n/a
Reporting on race, ethnicity, or other socially relevant groupings	n/a
Population characteristics	n/a
Recruitment	n/a
Ethics oversight	n/a

Note that full information on the approval of the study protocol must also be provided in the manuscript.

Field-specific reporting

Please select the one below that is the best fit for your research. If you are not sure, read the appropriate sections before making your selection.

☒ Life sciences ☐ Behavioural & social sciences ☐ Ecological, evolutionary & environmental sciences

For a reference copy of the document with all sections, see nature.com/documents/nr-reporting-summary-flat.pdf

Life sciences study design

All studies must disclose on these points even when the disclosure is negative.

Sample size	Prior sample size determination was not done. Key experiments were repeated by 3 independent researchers. If all 3 replicates gave similar results, experiments were considered as reproducible and completed.
Data exclusions	ROUT test with Q-value of 0.1 as a cut-off was used to exclude outliers. The data exclusion criterion was pre-established. All data were retained if removing outliers could have resulted in a sample size of less than 3. No outliers were removed from the datasets reported.
Replication	Each experiment was performed with at least 3 biological replicates. All the reported results are from experiments in which every repeat gave similar results.
Randomization	For in vitro experiments, cells from the same pool of BMDMs were randomly split into separate wells and subjected to the infection. For in vivo experiments, animals from the same cage were randomly selected for mock treatment or infection.
Blinding	Investigators were not blinded. None of the reported experiments require subjective decision making. Key experiments were repeated by 3 independent researchers. Therefore, there was no need for blinding.

Reporting for specific materials, systems and methods

We require information from authors about some types of materials, experimental systems and methods used in many studies. Here, indicate whether each material, system or method listed is relevant to your study. If you are not sure if a list item applies to your research, read the appropriate section before selecting a response.

Materials & experimental systems

n/a	Involved in the study	
	X	Antibodies
	X	Eukaryotic cell lines
X		Palaeontology and archaeology
	X	Animals and other organisms
X		Clinical data
X		Dual use research of concern
X		Plants

Methods

n/a	Involved in the study	
X		ChIP-seq
X		Flow cytometry
X		MRI-based neuroimaging

Antibodies

Antibodies used

Immunoblotting: anti-caspase-1 (AdipoGen, AG-20B-0042, 1:1000), anti-caspase-3 (CST, #9662, 1:1000), anti-cleaved caspase-3 (CST, #9661, 1:1000), anti-caspase-7 (CST, #9492, 1:1000), anti-cleaved caspase-7 (CST, #9491, 1:1000), anti-caspase-8 (CST, #4927, 1:1000), anti-cleaved caspase-8 (CST, #8592, 1:1000), anti-pRIPK3 (CST, #91702S, 1:1000), anti-RIPK3 (ProSci, #2283, 1:1000), anti-pMLKL (CST, #37333, 1:1000), anti-MLKL (Abgent, AP14272b, 1:1000), anti-GSDMD (Abcam, ab209845, 1:1000), anti-β-actin (Proteintech, 66009-1-IG, 1:5000), anti-ICP0 (Santa Cruz Biotechnology, sc-53070, 1:1000), anti-ICP8 (Santa Cruz Biotechnology, sc-53329, 1:1000), anti-gD (Santa Cruz Biotechnology, sc-21719, 1:1000), anti-gC (Abcam, ab6509, 1:2000), anti-GAPDH (Santa Cruz Biotechnology, sc-365062, 1:3000), anti-VP22 (kindly provided by Yasushi Kawaguchi, 1:2000), anti-CASP6 (CST, #9762, 1:2000), anti-CASP9 (CST, #9504, 1:2000), anti-tRIPK3 (Prosci, #2283, 1:2000), anti-cGAS (CST, #31659, 1:2000), anti-STING (CST, #13647, 1:2000), and anti-IRF1 (CST, #8478, 1:2000); secondary anti-rabbit (111-035-047) or anti-mouse (315-035-047) HRP antibodies from Jackson ImmunoResearch Laboratories.

Microscopy: The primary antibodies used were anti-ASC (Millipore, 04-147; 1:100), anti-tubulin (CST, 2144). The secondary antibodies used were Alexa Fluor 488- conjugated anti-mouse IgG (Life Technologies, A21202; 1:200) and Alexa Fluor 568-conjugated anti-rabbit IgG (Life Technologies, A10042; 1:200).

Validation

All antibodies were validated by their source company.
anti-caspase-1 (AdipoGen, AG-20B-0042): Measuring the inflammasome: O. Gross; Methods Mol. Biol. (2012). https://adipogen.com/ag-20b-0042-anti-caspase-1-p20-mouse-mab-casper-1.html
anti-caspase-3 (CST, #9662): Type I interferon signaling mediates Mycobacterium tuberculosis-induced macrophage death: L. Zhang; J. Exp. Med. (2021). https://www.cellsignal.com/products/primary-antibodies/caspase-3-antibody/9662
anti-cleaved caspase-3 (CST, #9661): RIPK1 Distinctly Regulates Yersinia-Induced Inflammatory Cell Death, PANoptosis: R K Subbarao Malireddi; Immunohorizons (2020). https://www.cellsignal.com/products/primary-antibodies/cleaved-caspase-3-asp175-antibody/9661
anti-caspase-7 (CST, #9492): RIPK1 Distinctly Regulates Yersinia-Induced Inflammatory Cell Death, PANoptosis: R K Subbarao Malireddi; Immunohorizons (2020). https://www.cellsignal.com/products/primary-antibodies/caspase-7-antibody/9492
anti-cleaved caspase-7 (CST, #9491): RIPK1 Distinctly Regulates Yersinia-Induced Inflammatory Cell Death, PANoptosis: R K Subbarao Malireddi; Immunohorizons (2020). https://www.cellsignal.com/products/primary-antibodies/cleaved-caspase-7-asp198-antibody/9491
anti-caspase-8 (CST, #4927): Caspase-6 Is a Key Regulator of Innate Immunity, Inflammasome Activation, and Host Defense: Min Zheng; Cell (2020). https://www.cellsignal.com/products/primary-antibodies/caspase-8-antibody-mouse-specific/4927
anti-cleaved caspase-8 (CST, #8592): Caspase-6 Is a Key Regulator of Innate Immunity, Inflammasome Activation, and Host Defense: Min Zheng; Cell (2020). https://www.cellsignal.com/products/primary-antibodies/caspase-8-antibody-mouse-specific/8592
anti-pRIPK3 (CST, #91702S): Diverse sequence determinants control human and mouse receptor interacting protein 3 (RIP3) and mixed lineage kinase domain-like (MLKL) interaction in necroptotic signaling: Wanze Chen; J. Biol. Chem. (2013). https://www.cellsignal.com/products/primary-antibodies/phospho-rip3-thr231-ser232-e7s1r-rabbit-mab/91702?site-search-type=Products&N=4294956287&Ntt=91702s&fromPage=plp&_requestid=4790034
anti-RIPK3 (ProSci, #2283): Receptor interacting protein kinase-3 determines cellular necrotic response to TNF-alpha: Sudan He; Cell (2009). https://www.prosci-inc.com/rip3-antibody-2283.html
anti-pMLKL (CST, #37333): Caspase-6 Is a Key Regulator of Innate Immunity, Inflammasome Activation, and Host Defense. Min Zheng; Cell (2020). https://www.cellsignal.com/products/primary-antibodies/phospho-mlkl-ser345-d6e3g-rabbit-mab/37333
anti-MLKL (Abgent, AP14272b): Caspase-6 Is a Key Regulator of Innate Immunity, Inflammasome Activation, and Host Defense. Min Zheng; Cell (2020). https://www.citeab.com/antibodies/240195-ap14272b-m-mlkl-antibody-c-term
anti-GSDMD (Abcam, ab209845): Caspase-6 Is a Key Regulator of Innate Immunity, Inflammasome Activation, and Host Defense: Min Zheng; Cell (2020). https://www.abcam.com/gsdmd-antibody-epr19828-ab209845.html
anti-β-actin (Proteintech, 66009-1-IG): Caspase-6 Is a Key Regulator of Innate Immunity, Inflammasome Activation, and Host Defense: Min Zheng; Cell (2020). https://www.ptglab.com/products/Pan-Actin-Antibody-66009-1-Ig.htm
anti-ICP0 (Santa Cruz Biotechnology, sc-53070): N-Terminal Phosphorylation Sites of Herpes Simplex Virus 1 ICP0 Differentially Regulate Its Activities and Enhance Viral Replication: Heba H. Mostafa; Journal of Virology (2013). https://www.scbt.com/p/hsv-1-icp0-antibody-11060?srsltid=AfmBOopfMFQEzX32yql0cB6yPdX1_yss7yAn7NU7ATc94MsC4NTAnt27
anti-ICP8 (Santa Cruz Biotechnology, sc-53329): Herpes Simplex Virus Type 1 Enhances Expression of the Synaptic Protein Arc for Its Own Benefit: Francisca Acuña-Hinrichsen; Frontiers in Cellular Neuroscience (2019). https://www.scbt.com/ko/p/hsv-1-icp8-antibody-10a3
anti-gD (Santa Cruz Biotechnology, sc-21719): TRPC1 participates in the HSV-1 infection process by facilitating viral entry: DONGXU HE; Science Advances (2020). https://www.scbt.com/ko/p/hsv-1-gd-antibody-dl6
anti-gC (Abcam, ab6509): Inhibition of herpes simplex virus-1 infection by MBZM-N-IBT: in silico and in vitro studies: Abhishek Kumar; Virology Journal (2021). https://www.abcam.com/en-us/products/primary-antibodies/hsv1-gc-envelope-protein-antibody-3g9-ab6509
anti-GAPDH (Santa Cruz Biotechnology, sc-365062): Biomaterial Surface-Mediated Macrophages Exert Immunomodulatory Roles by

Exosomal CCL2-Induced Membrane Integrin β1 Trafficking in Recipient Cells: Yuyu Zhao; Adavanced Science (2025). https://www.scbt.com/p/gapdh-antibody-g-9?srsltid=AfmBOooqFg1-yxqTnji-blfo1J2R3dLFyYksyJi4jiwQyOxGthloWJ36
anti-VP22 (kindly provided by Yasushi Kawaguchi): Herpes Simplex Virus 1 VP22 Inhibits AIM2-Dependent Inflammasome Activation to Enable Efficient Viral Replication: Yuhei Maruzuru: Cell Host & Microbe (2018).
anti-CASP6 (CST, #9762): Caspase-6 Is a Key Regulator of Innate Immunity, Inflammasome Activation, and Host Defense. Min Zheng; Cell (2020). https://www.cellsignal.com/products/primary-antibodies/caspase-6-antibody/9762?srsltid=AfmBOopxkj2HfESn2BR4encz-LjpSTtuxY818LZ_fwuuXi4GZJxgXgG1
anti-CASP9 (CST, #9504): ICE-LAP6, a novel member of the ICE/Ced-3 gene family, is activated by the cytotoxic T cell protease granzyme B: H Duan; Journal of Biological Chemistry (1996). https://www.cellsignal.com/products/primary-antibodies/caspase-9-antibody/9504?srsltid=AfmBOoqPEW0HnSM9cqkvPqVM6KFaI5NztTAfaP5LtWCiH4ufWag0-cnv
anti-cGAS (CST, #31659): Cyclic GMP-AMP synthase is a cytosolic DNA sensor that activates the type I interferon pathway: Lijun Sun; Science (2013). https://www.cellsignal.com/products/primary-antibodies/cgas-d3o8o-rabbit-monoclonal-antibody/31659?srsltid=AfmBOoqw0FJ2QeKmPV4IVnr53DbI8hsI_2xlmIxiO61MEPCDxcvjUwEC
anti-STING (CST, #13647): STING is an endoplasmic reticulum adaptor that facilitates innate immune signalling: Hiroki Ishikawa; Nature (2008). https://www.cellsignal.com/products/primary-antibodies/sting-d2p2f-rabbit-monoclonal-antibody/13647?srsltid=AfmBOoogUeuOGUG83kC5YCwMpTSErCjplXKH-AsbCnAVQVkWIX4uwSkH
anti-IRF1 (CST, #8478): Elevated level of SUMOylated IRF-1 in tumor cells interferes with IRF-1-mediated apoptosis: Junsoo Park; PNAS (2007). https://www.cellsignal.com/products/primary-antibodies/irf-1-d5e4-rabbit-monoclonal-antibody/8478?srsltid=AfmBOoq57fT9L9HjgYWVLsCK6WAda7SbxMFMGHZOv-oM_cxs0poDw8fo
anti-ASC (Millipore, #04-147): HMGB1 promotes the activation of NLRP3 and caspase-8 inflammasomes via NF-kB pathway in acute glaucoma: W. Chi; J Neuroinflammation (2015).. https://www.emdmillipore.com/US/en/product/Anti-ASC-Antibody-clone-2EI-7,MM_NF-04-147?ReferrerURL=https%3A%2F%2Fwww.google.com%2F&bd=1
anti-tubulin (CST, 2144): Asynchronous mouse embryo polarization leads to heterogeneity in cell fate specification: Adiyant Lamba; Elife (2025). https://www.cellsignal.com/products/primary-antibodies/alpha-tubulin-antibody/2144?srsltid=AfmBOorcipilBxxAw89c5OIDhaPtMHbDo-0DEf9c36aip8_4ZIATs4ou
anti-AIM2 (Abcam, ab119791): A prodrug of epigallocatechin-3-gallate alleviates high glucose-induced pro-angiogenic factor production by inhibiting the ROS/TXNIP/NLRP3 inflammasome axis in retinal Müller cells: JingxiaDu; Experimental Eye Research (2020). https://www.abcam.com/aim2-antibody-ab119791.html

Eukaryotic cell lines

Policy information about cell lines and Sex and Gender in Research

Cell line source(s)	L929 cells (ATCC, CCL-1); THP-1 cells and Vero cells were kindly provided by Atsushi Kawaguchi (University of Tsukuba); 293FT cells (Thermo Fisher Scientific, Cat. No. R70007); HaCaT cells (Cytion, Cat. No. 300493); WT iBMDMs were kindly provided by Tae-Hyuk Kwon (UNIST).
Authentication	Cell line authentication was not performed for the cell lines used in this study. All cell lines were obtained from reputable sources or provided by established laboratories.
Mycoplasma contamination	All cell lines were tested and confirmed to be free of mycoplasma contamination.
Commonly misidentified lines (See ICLAC register)	None of the cell lines used in this study are listed in the ICLAC register of commonly misidentified cell lines.

Animals and other research organisms

Policy information about studies involving animals; ARRIVE guidelines recommended for reporting animal research, and Sex and Gender in Research

Laboratory animals	C57BL/6J mice (WT) were obtained from Hyochang Science, while Aim2−/− (Jackson Laboratory), Nlrp3−/− (Cyagen), Nlrc4−/− (Cyagen), Casp1−/− (Jackson Laboratory) mice, all on a C57BL/6J genetic background, were purchased from the indicated sources. All mice used in this study were of the species Mus musculus. The mice were group-housed, with up to five mice per cage, and were bred under standard pathogen-free conditions in the animal facility at the Ulsan National Institute of Science and Technology (UNIST). The mice were maintained on a 12-h light/dark cycle (lights on from 7 AM to 7 PM) at a controlled ambient temperature (22 ± 2°C) and relative humidity (50 ± 10%), and were provided with standard chow. Both male and female mice were included in this study. In vivo investigations utilized age- and sex-matched mice aged 6 to 8 weeks, while in vitro studies involved mice aged 6 to 12 weeks. Co-housed animals were chosen for in vivo analyses. All experimental procedures were executed following protocols approved by the Institutional Animal Care and Utilization Committee of UNIST [UNISTIACUC-23-22].
Wild animals	The study did not involve wild animals.
Reporting on sex	In vivo investigations utilized age- and sex-matched mice aged 6 to 8 weeks, while in vitro studies involved mice aged 6 to 12 weeks.
Field-collected samples	The study did not involve field collected samples.
Ethics oversight	Age- and sex-matched cohorts of 6- to 8-week-old mice, housed together, were utilized for the infection experiments. The study was approved by the Institutional Animal Care and Utilization Committee of UNIST (IACUC) under protocol number [UNISTIACUC-23-22].

Note that full information on the approval of the study protocol must also be provided in the manuscript.

Plants

Seed stocks	n/a
Novel plant genotypes	n/a
Authentication	n/a
