# Supplementary material for: Herpes simplex virus 1 harboring poly(T) DNA sequences as a key ligand for AIM2 inflammasome activation and host defense
Source: Nat Commun. 2026 Apr 13;17:5161. doi: 10.1038/s41467-026-71896-w (PMC13250049; doi:10.1038/s41467-026-71896-w)
Supplement: Supplementary file 8 — Source data [file 41467_2026_71896_MOESM8_ESM.zip › Source Data 2. 2nd revision-1 uncropped WB SL final.pdf]

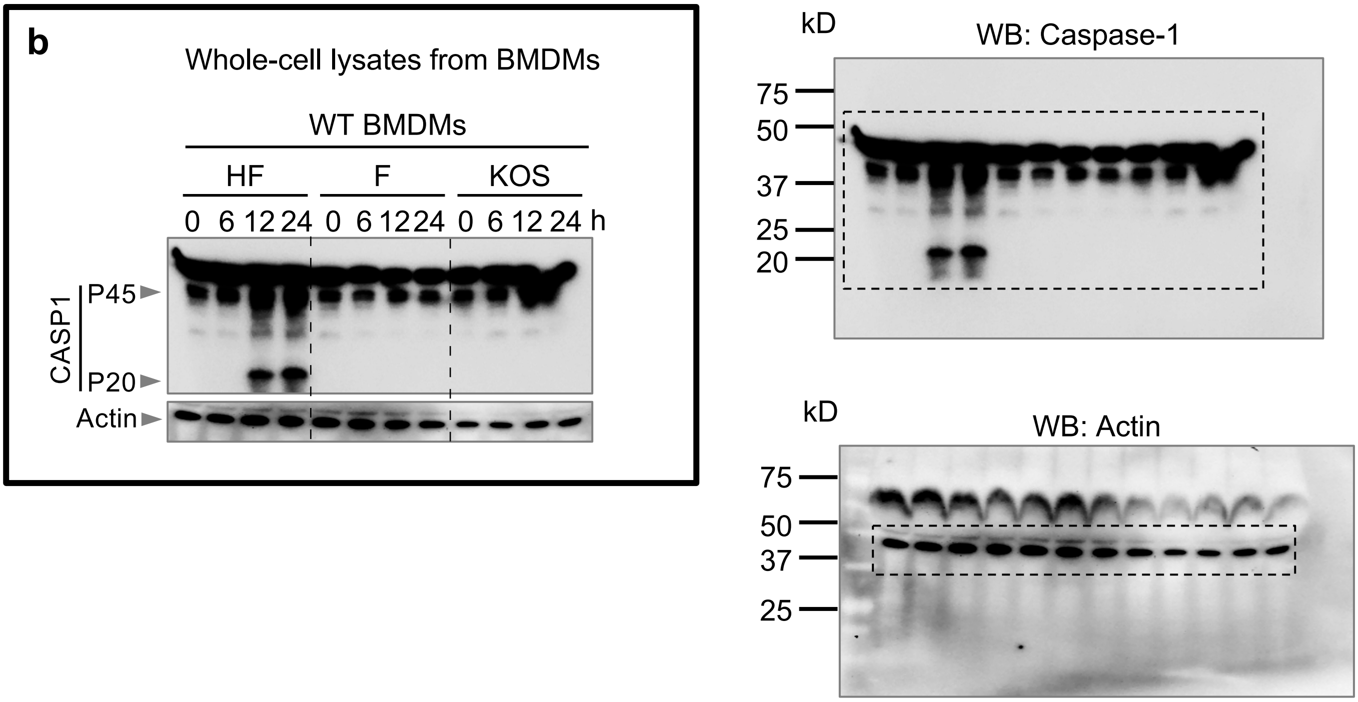
b
Whole-cell lysates from BMDMs
WT BMDMs
HF
F
KOS
0 6 12 24 0 6 12 24 0 6 12 24 h
CASP1
P45
P20
Actin
kD
WB: Caspase-1
75
50
37
25
20
kD
WB: Actin
75
50
37
25

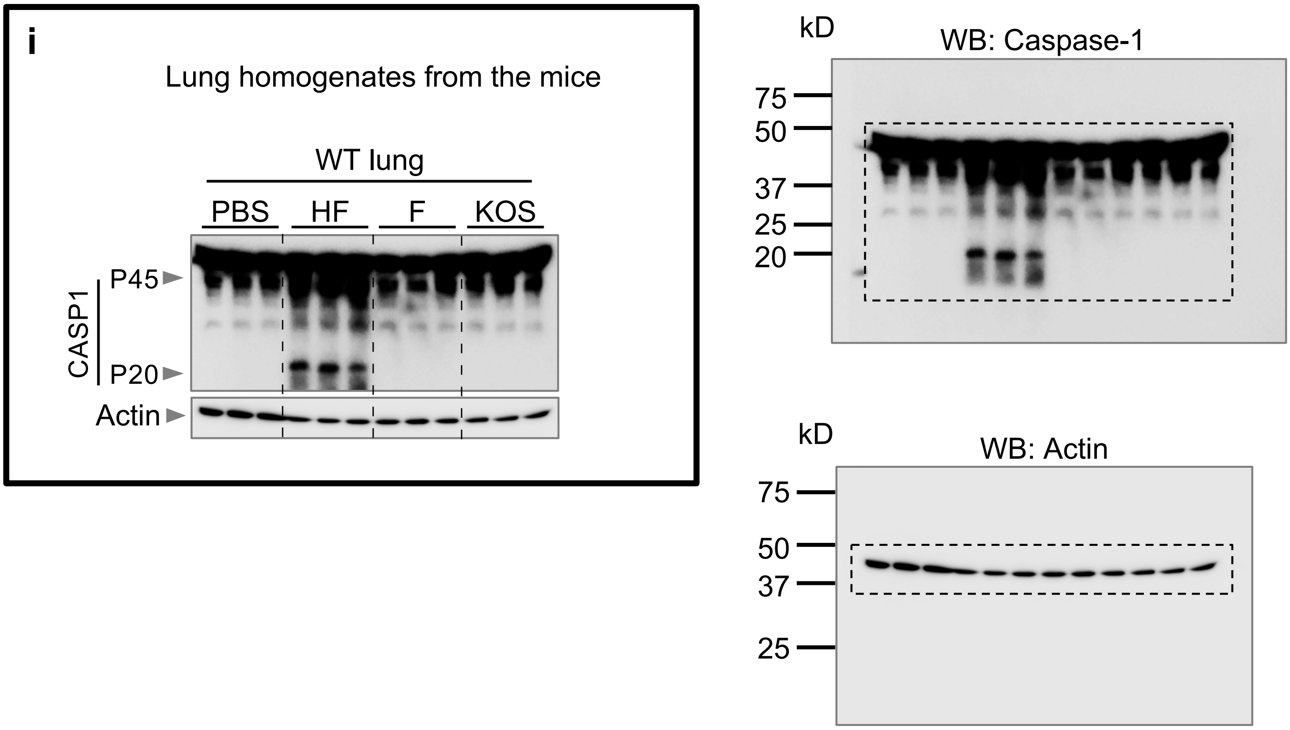
i
Lung homogenates from the mice
WT lung
PBS
HF
F
KOS
CASP1
P45
P20
Actin
kD
WB: Caspase-1
75
50
37
25
20
kD
WB: Actin
75
50
37
25

Figure 1

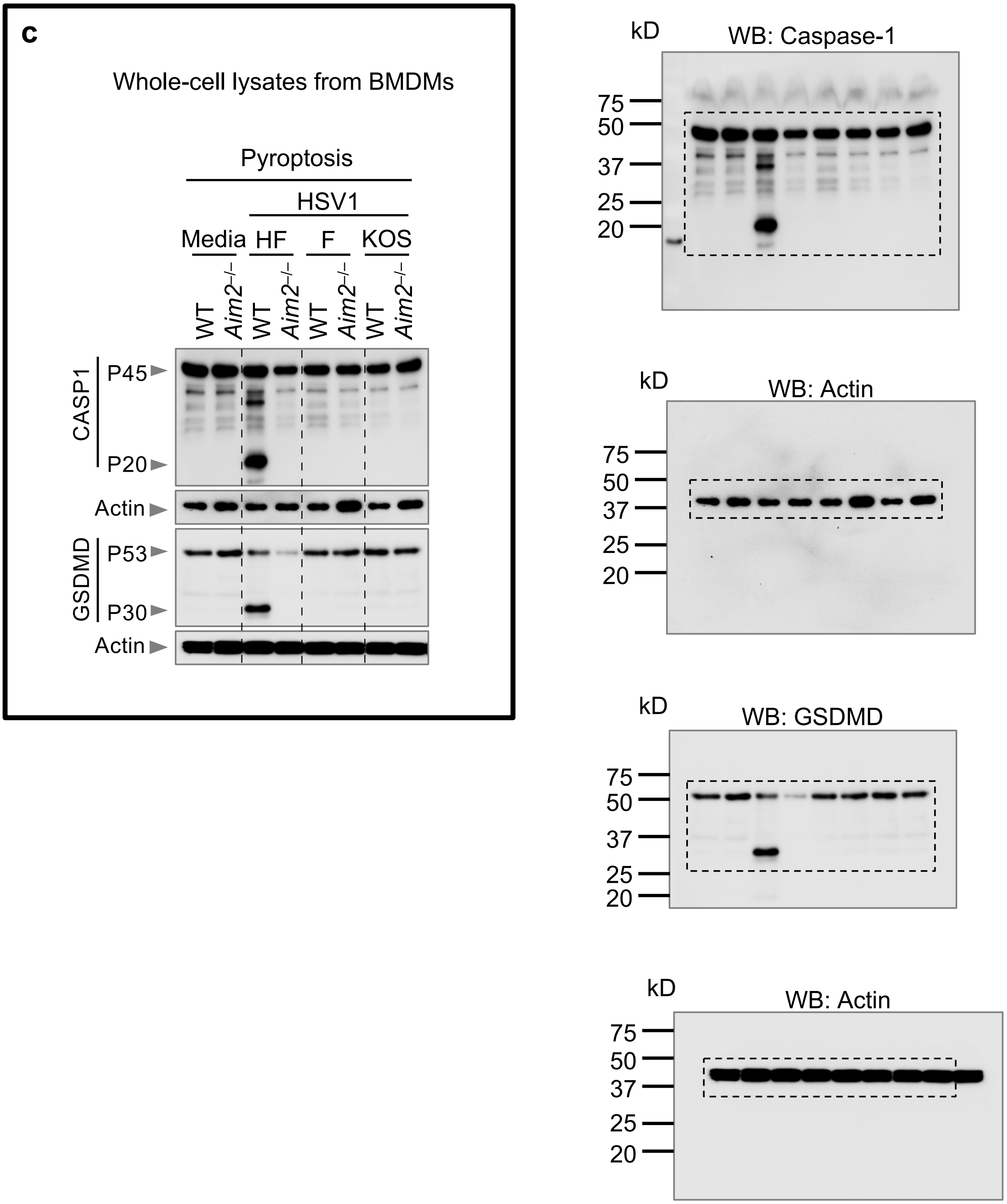
c
Whole-cell lysates from BMDMs
Pyroptosis
HSV1
Media
HF
F
KOS
WT
Aim2−/−
CASP1
P45
P20
Actin
GSDMD
P53
P30
kD
WB: Caspase-1
75
50
37
25
20
WB: Actin
WB: GSDMD

Figure 2

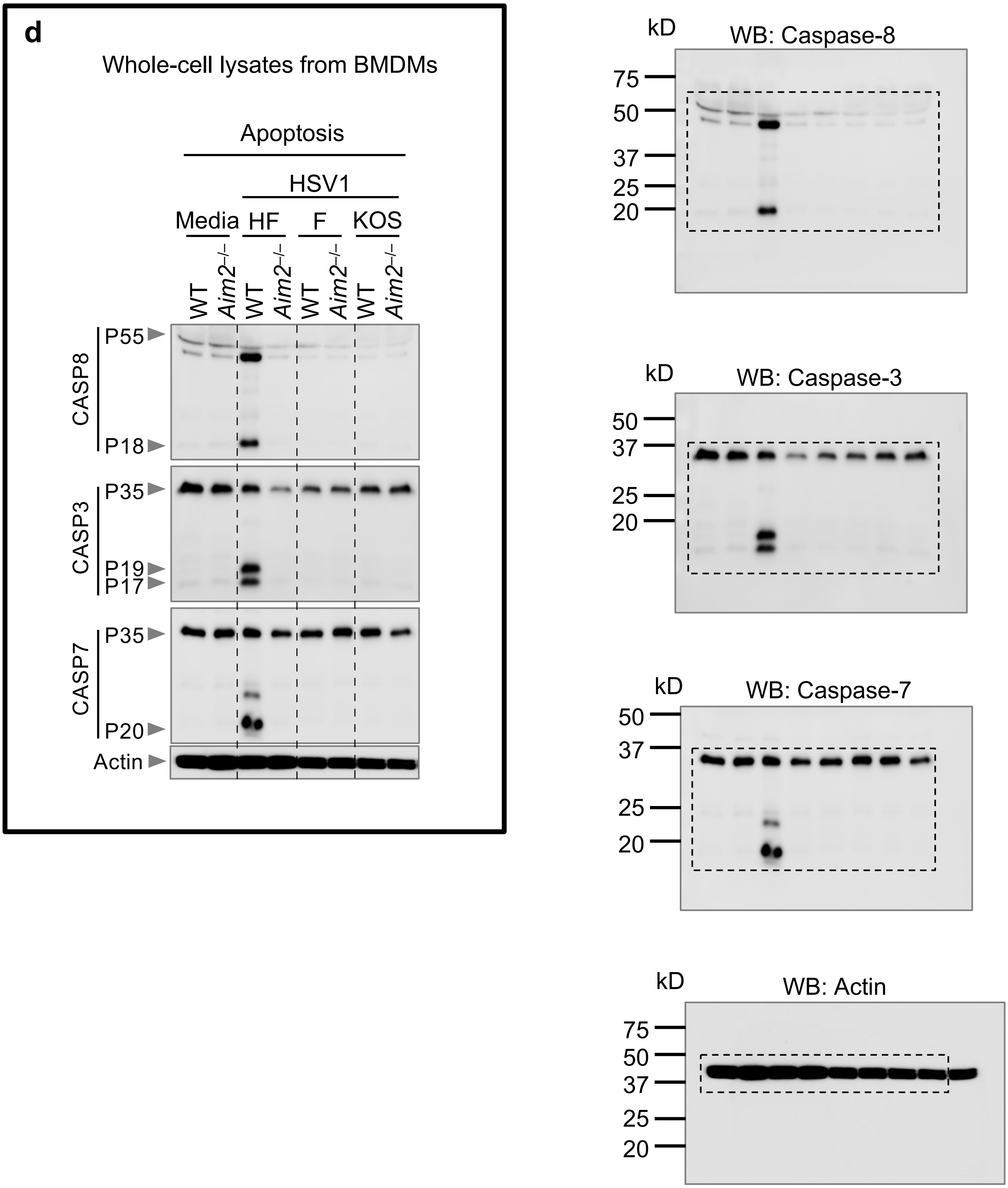
d
Whole-cell lysates from BMDMs
Apoptosis
HSV1
Media
HF
F
KOS
WT
Aim2−/−
WT
Aim2−/−
WT
Aim2−/−
WT
Aim2−/−
CASP8
P55
P18
CASP3
P35
P19
P17
CASP7
P35
P20
Actin
kD
WB: Caspase-8
75
50
37
25
20
kD
WB: Caspase-3
50
37
25
20
kD
WB: Caspase-7
50
37
25
20
kD
WB: Actin
75
50
37
25
20

Figure 2

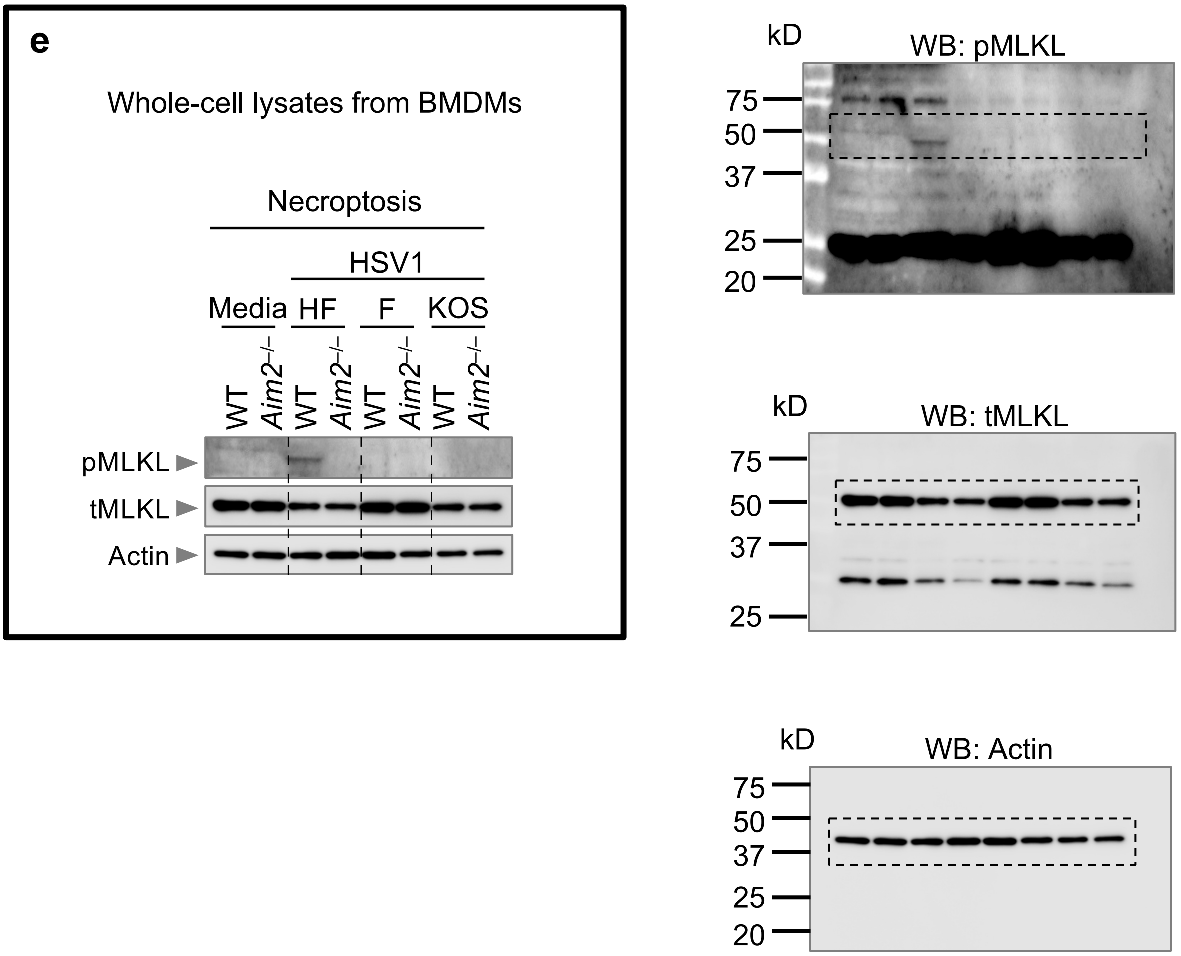
e
Whole-cell lysates from BMDMs
Necroptosis
HSV1
Media
HF
F
KOS
WT
Aim2−/−
WT
Aim2−/−
WT
Aim2−/−
WT
Aim2−/−
pMLKL
tMLKL
Actin
kD
WB: pMLKL
75
50
37
25
20
kD
WB: tMLKL
75
50
37
25
kD
WB: Actin
75
50
37
25
20

Figure 2

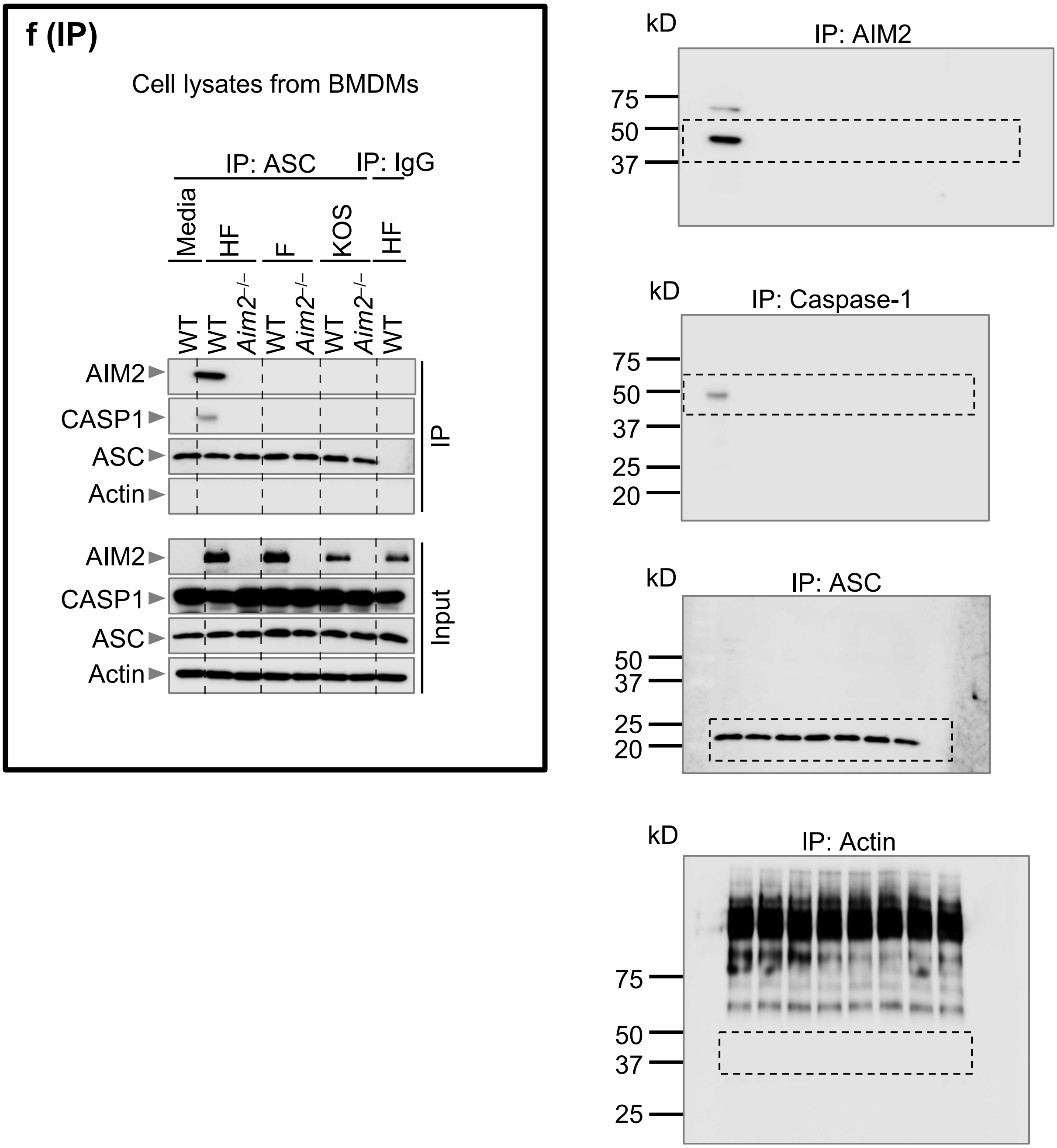
f (IP)
Cell lysates from BMDMs
IP: ASC
IP: IgG
Media
HF
F
KOS
HF
WT
WT
Aim2−/−
WT
Aim2−/−
WT
Aim2−/−
WT
AIM2
CASP1
ASC
Actin
IP
AIM2
CASP1
ASC
Actin
Input
kD
IP: AIM2
75
50
37
kD
IP: Caspase-1
75
50
37
25
20
kD
IP: ASC
50
37
25
20
kD
IP: Actin
75
50
37
25

Figure 2

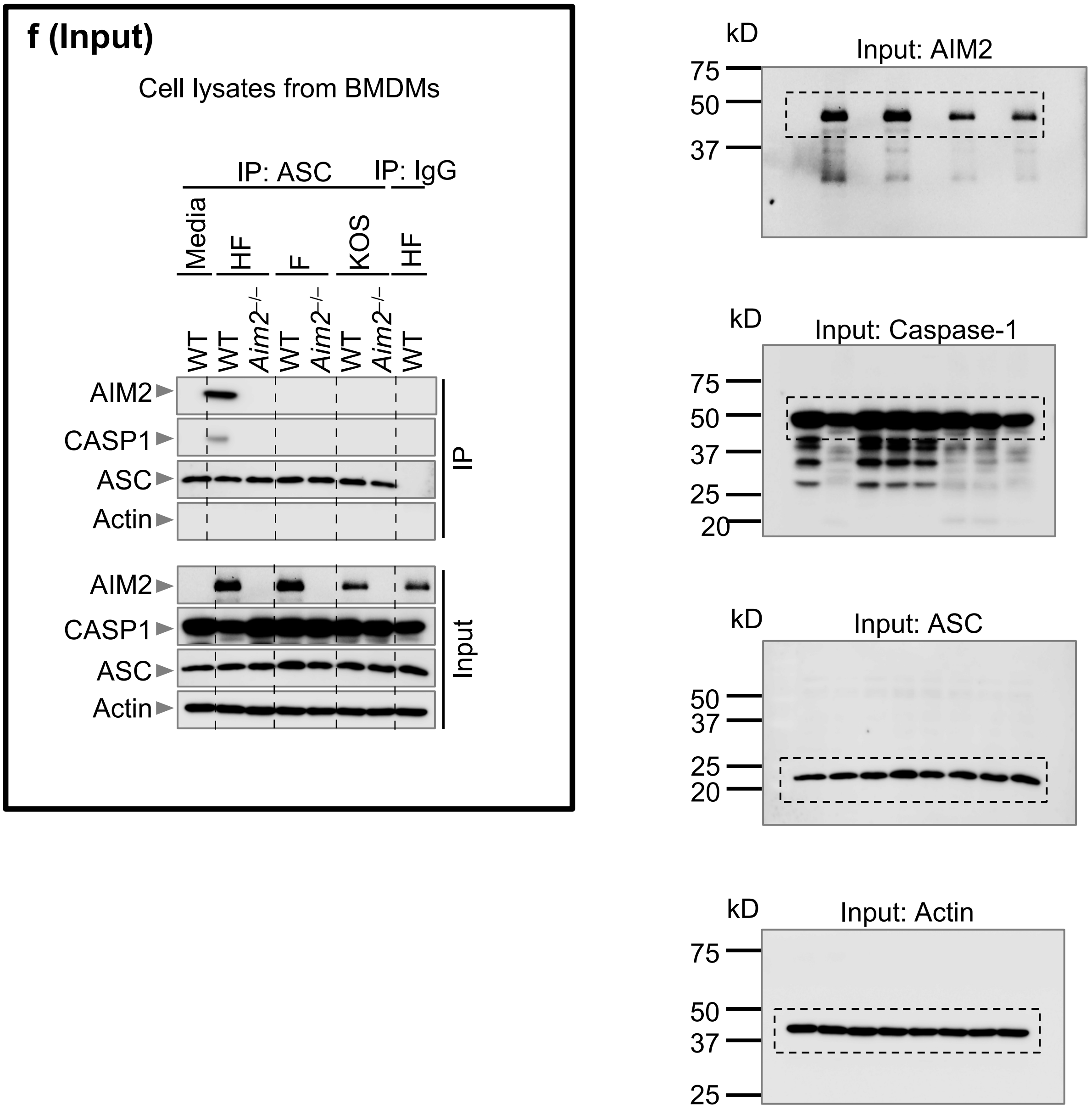

f (Input)
Cell lysates from BMDMs
IP: ASC
IP: IgG
Media
HF
F
KOS
HF
WT
WT
Aim2−/−
WT
Aim2−/−
WT
Aim2−/−
WT
AIM2
CASP1
ASC
Actin
IP
AIM2
CASP1
ASC
Actin
Input
kD
Input: AIM2
75
50
37
kD
Input: Caspase-1
75
50
37
25
20
kD
Input: ASC
50
37
25
20
kD
Input: Actin
75
50
37
25

Figure 2

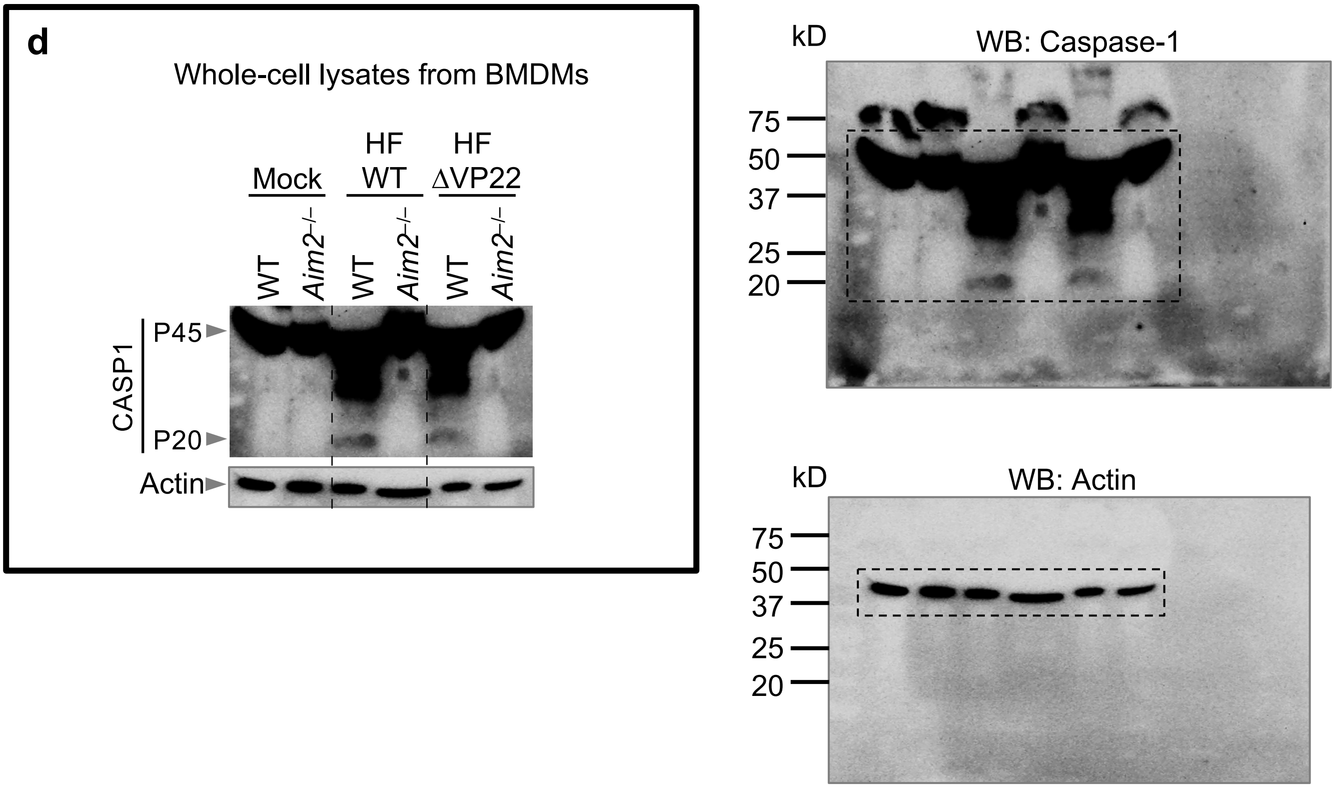
d
Whole-cell lysates from BMDMs
Mock
HF WT
HF ΔVP22
WT
Aim2–/–
CASP1
P45
P20
Actin
kD
WB: Caspase-1
75
50
37
25
20
WB: Actin

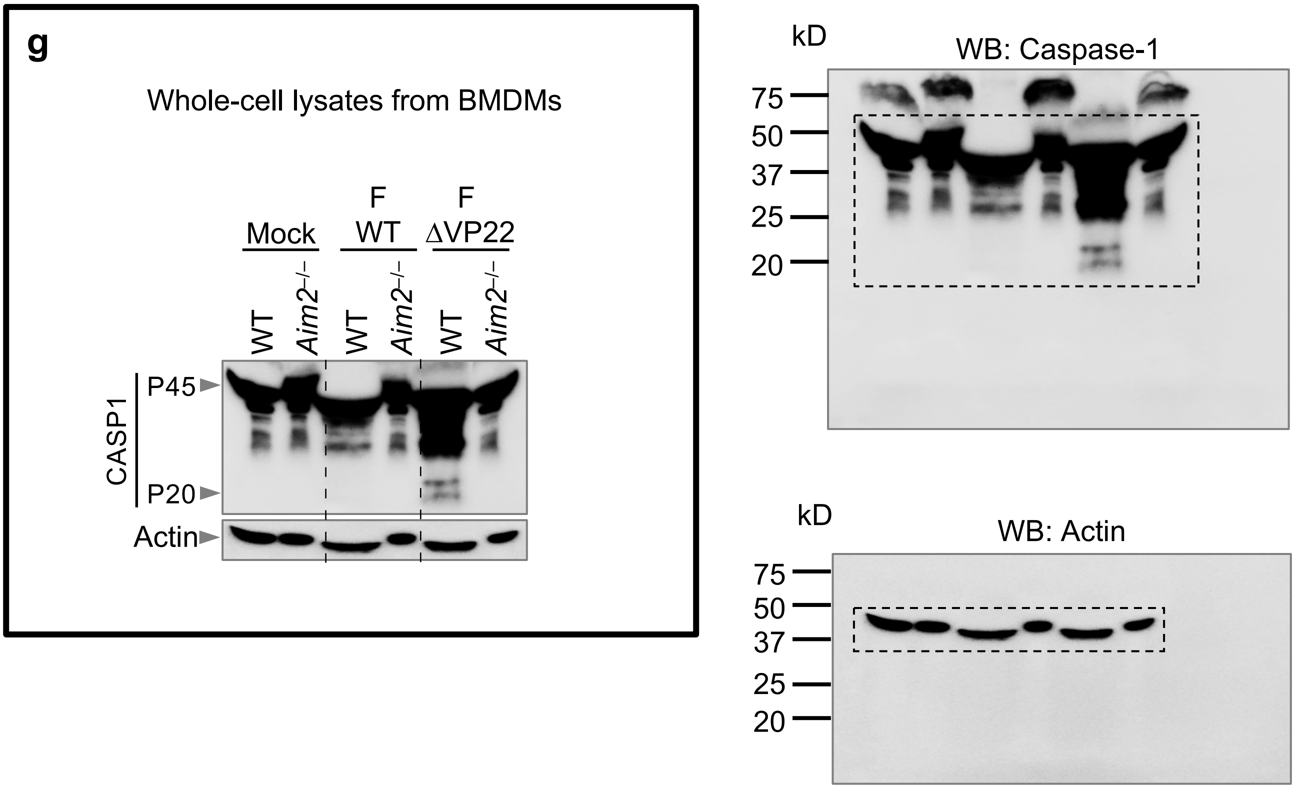
g
Whole-cell lysates from BMDMs
Mock
F WT
F ΔVP22
WT
Aim2–/–
CASP1
P45
P20
Actin
kD
WB: Caspase-1
75
50
37
25
20
WB: Actin

Figure 3

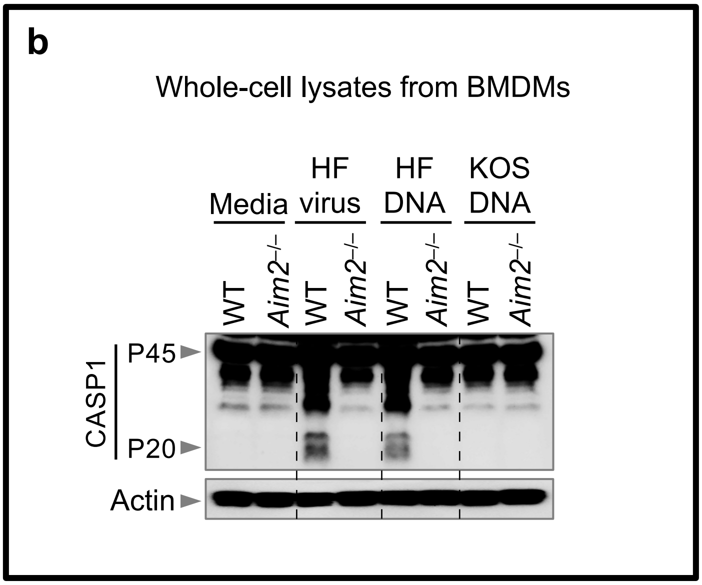
b
Whole-cell lysates from BMDMs
Media
HF virus
HF DNA
KOS DNA
WT
Aim2−/−
CASP1
P45
P20
Actin

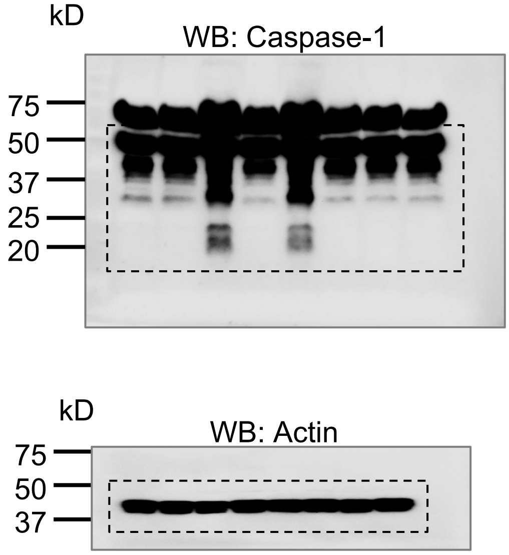
kD
WB: Caspase-1
75
50
37
25
20
kD
WB: Actin
75
50
37

Figure 4

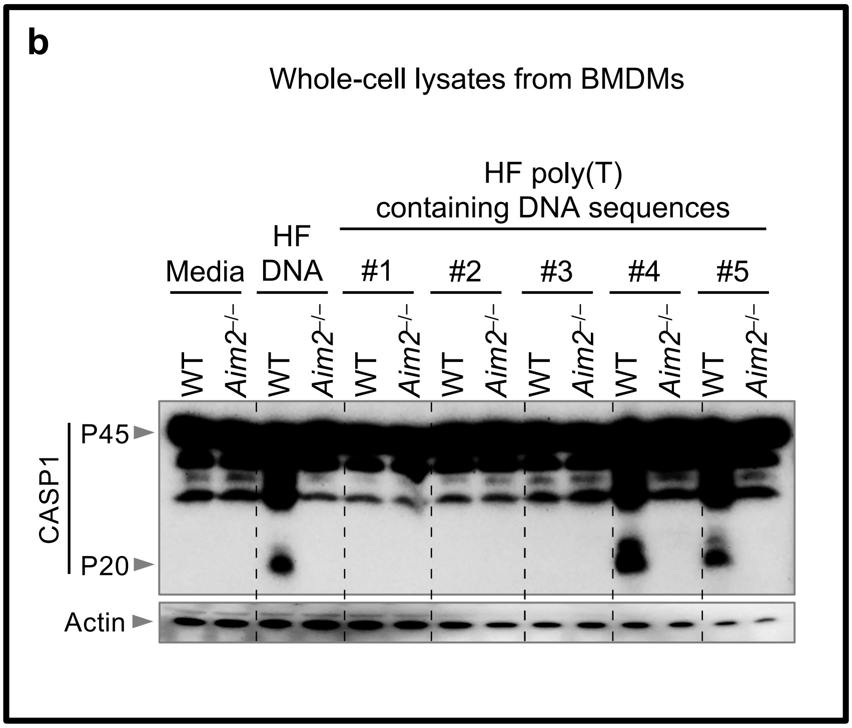
b
Whole-cell lysates from BMDMs
HF poly(T)
containing DNA sequences
Media
HF
DNA
#1
#2
#3
#4
#5
WT
Aim2−/−
CASP1
P45
P20
Actin

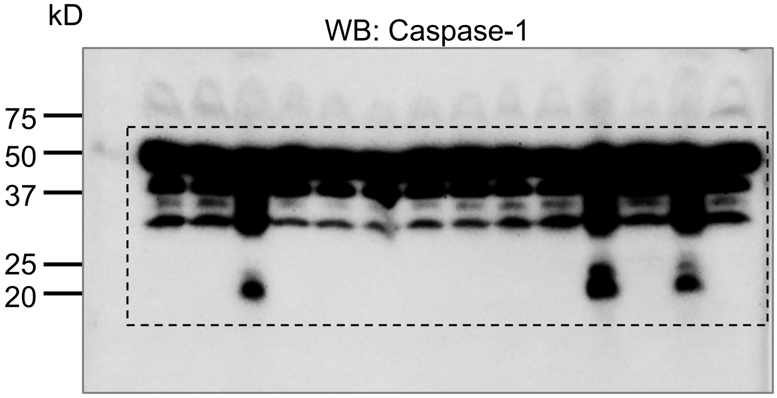
kD
WB: Caspase-1
75
50
37
25
20

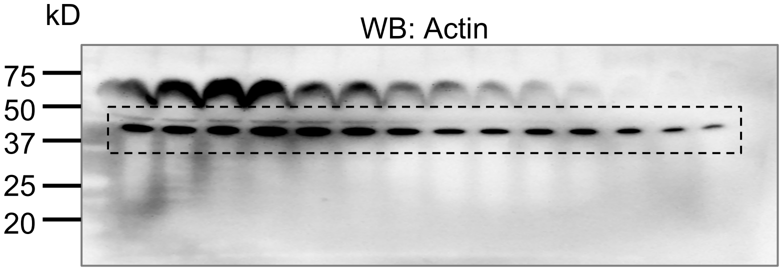
kD
WB: Actin
75
50
37
25
20

Figure 5

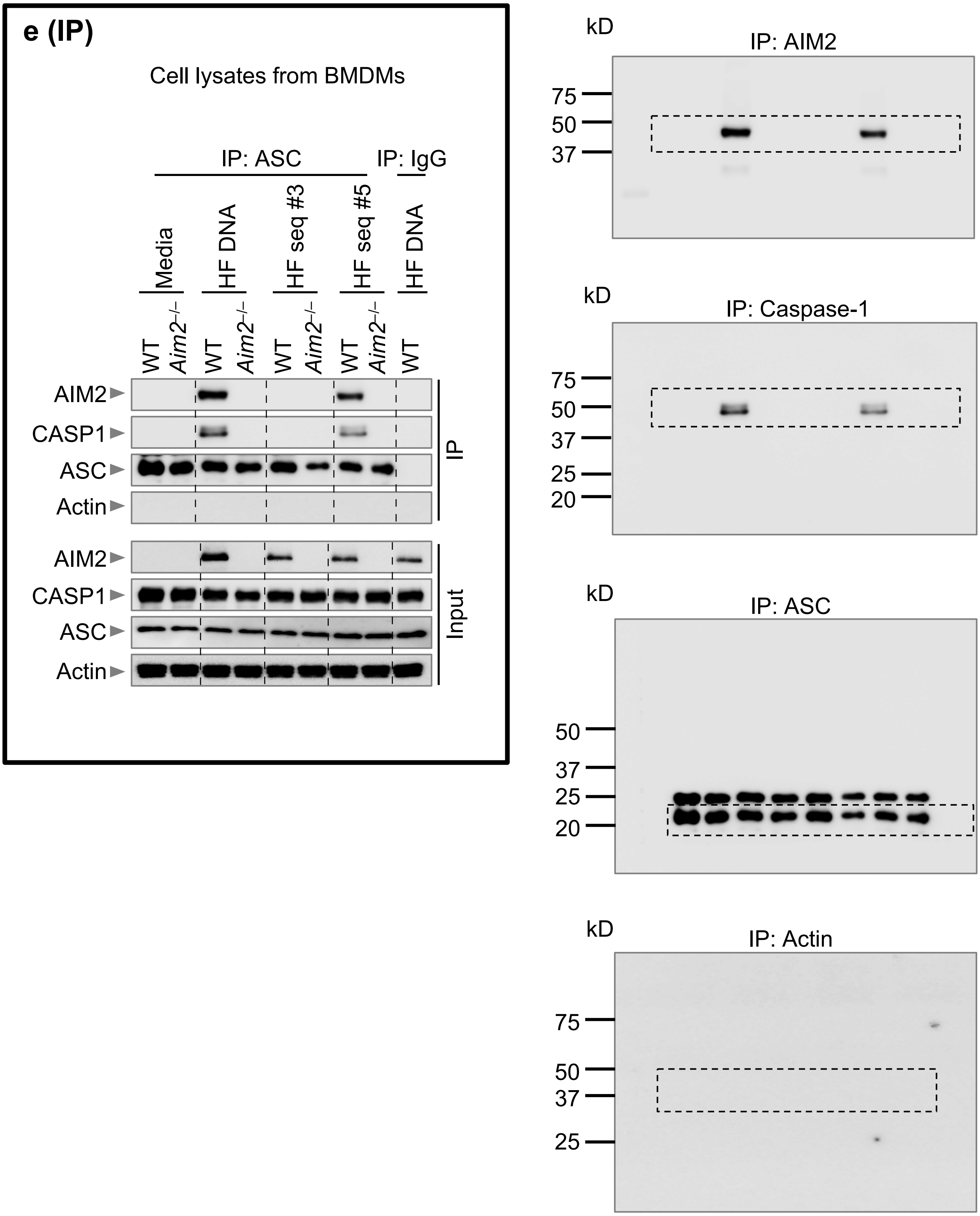
e (IP)
Cell lysates from BMDMs
IP: ASC
IP: IgG
Media
HF DNA
HF seq #3
HF seq #5
HF DNA
WT
Aim2−/−
WT
Aim2−/−
WT
Aim2−/−
WT
Aim2−/−
WT
AIM2
CASP1
ASC
Actin
IP
AIM2
CASP1
ASC
Actin
Input
kD
IP: AIM2
75
50
37
kD
IP: Caspase-1
75
50
37
25
20
kD
IP: ASC
50
37
25
20
kD
IP: Actin
75
50
37
25

Figure 5

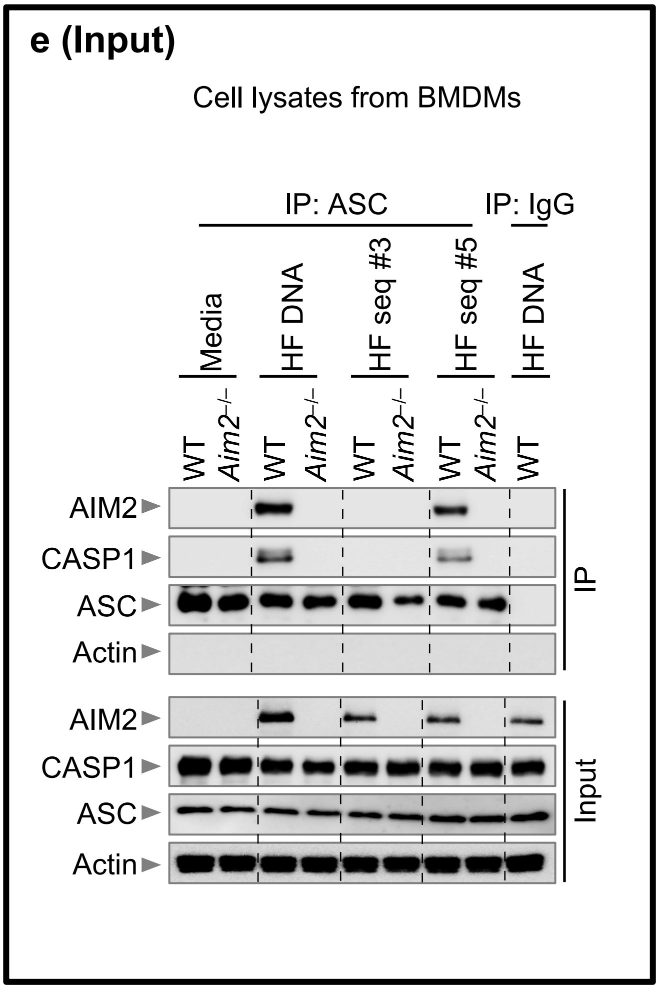
e (Input)
Cell lysates from BMDMs
IP: ASC
IP: IgG
Media
HF DNA
HF seq #3
HF seq #5
HF DNA
WT
Aim2−/−
AIM2
CASP1
ASC
Actin
IP
Input

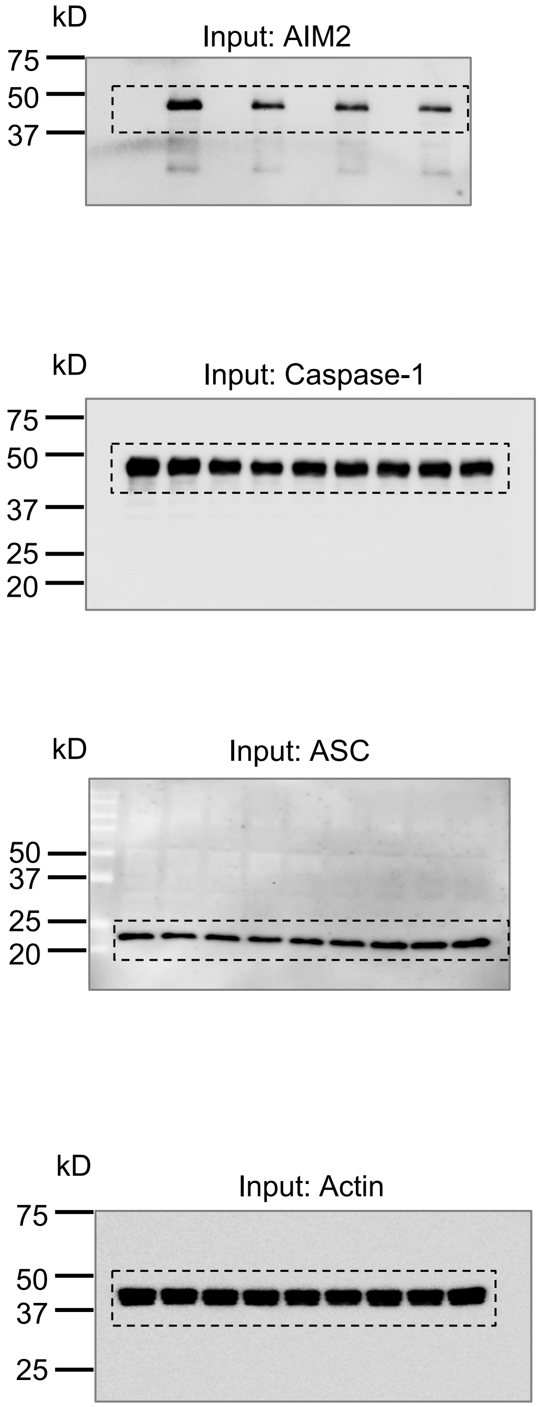
kD
Input: AIM2
75
50
37
kD
Input: Caspase-1
75
50
37
25
20
kD
Input: ASC
50
37
25
20
kD
Input: Actin
75
50
37
25

Figure 5

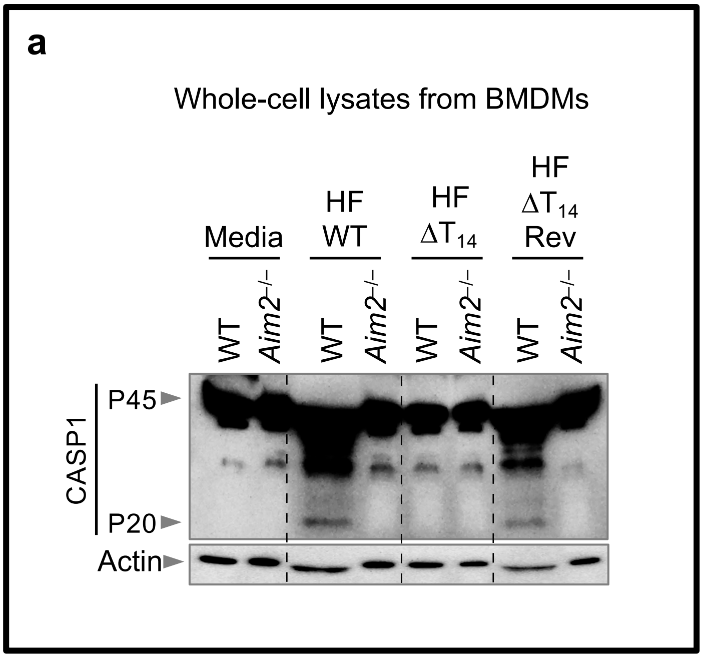
a
Whole-cell lysates from BMDMs
Media
HF WT
HF ΔT14
HF ΔT14 Rev
WT
Aim2−/−
CASP1
P45
P20
Actin

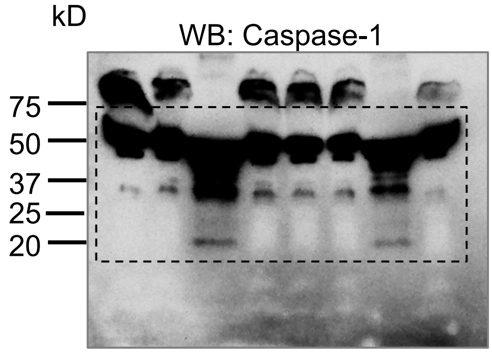
kD
WB: Caspase-1
75
50
37
25
20

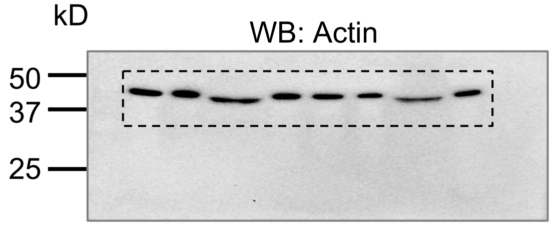
kD
WB: Actin
50
37
25

Figure 6

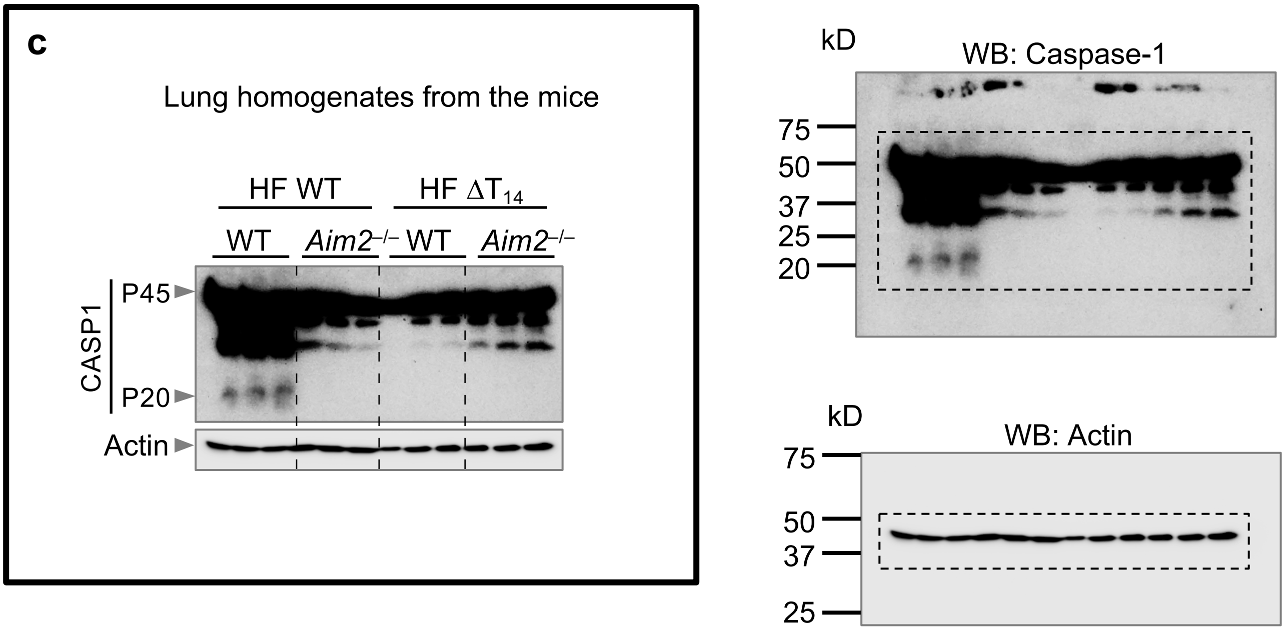
c
Lung homogenates from the mice
HF WT
HF ΔT14
WT
Aim2–/–
WT
Aim2–/–
CASP1
P45
P20
Actin
kD
WB: Caspase-1
75
50
37
25
20
kD
WB: Actin
75
50
37
25

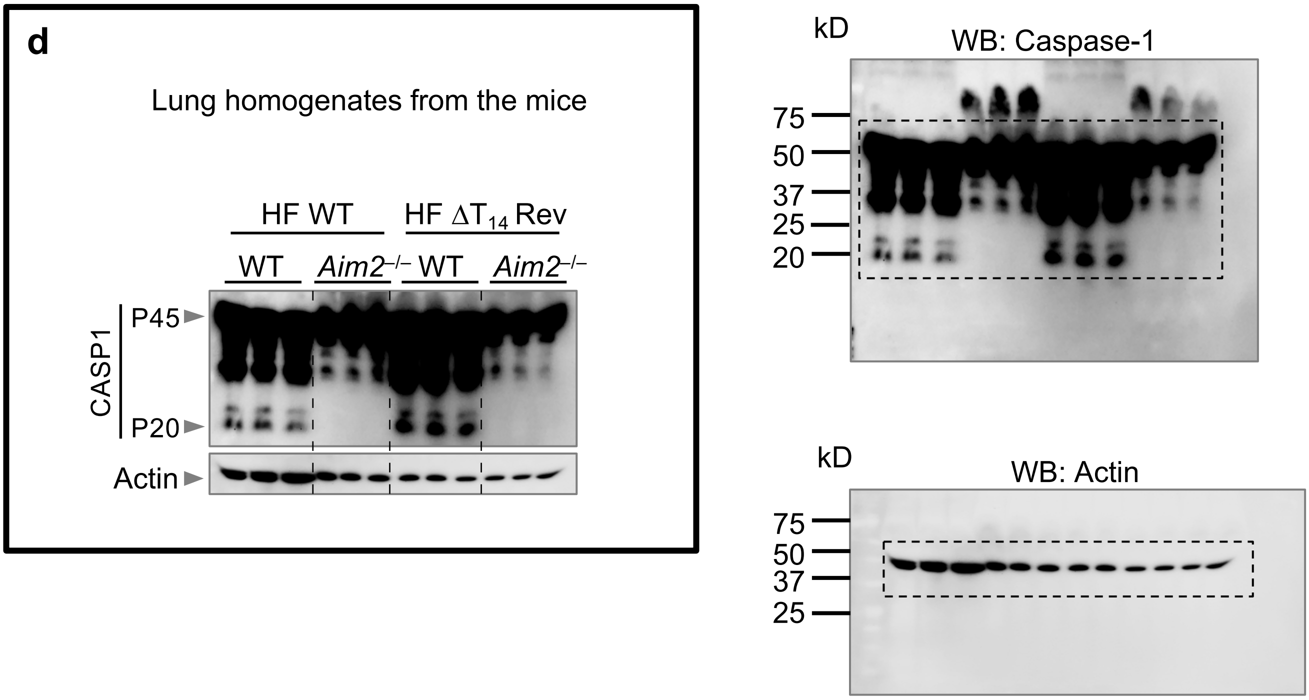
d
Lung homogenates from the mice
HF WT
HF ΔT14 Rev
WT
Aim2–/–
WT
Aim2–/–
CASP1
P45
P20
Actin
kD
WB: Caspase-1
75
50
37
25
20
kD
WB: Actin
75
50
37
25

Figure 7
