# Supplementary material for: Herpes simplex virus 1 harboring poly(T) DNA sequences as a key ligand for AIM2 inflammasome activation and host defense
Source: Nat Commun. 2026 Apr 13;17:5161. doi: 10.1038/s41467-026-71896-w (PMC13250049; doi:10.1038/s41467-026-71896-w)
Supplement: Supplementary file 7 — Transparent Peer Review file [file 41467_2026_71896_MOESM7_ESM.pdf]

nature portfolio

Peer Review File

Herpes Simplex Virus 1 Harboring Poly(T) DNA Sequences as a Key Ligand for AIM2 Inflammasome Activation and Host Defense

Corresponding Author: Professor SangJoon Lee

This file contains all reviewer reports in order by version, followed by all author rebuttals in order by version.

Version 0:

Reviewer comments:

Reviewer #1

(Remarks to the Author)
This study focuses on the differences in AIM2 inflammasome activation among HSV-1 strains, revealing that the HF strain strongly stimulates the inflammasome, whereas the F and KOS strains show minimal activation. In this work, genomic analysis identified unique 14 bp poly(T) DNA sequences in the HF strain, located between the UL25 and UL26 genes, as critical for AIM2 inflammasome activation. Removing these sequences from the HF strain confirmed their importance, showing reduced inflammasome activation and compromised host defense. Transfection experiments with various lengths of poly(T) DNA further suggested that longer sequences are essential for AIM2 activation. These interesting findings not only clarify the role of poly(T) sequences in AIM2 inflammasome activation during HSV-1 infection but also suggest broader pathological implications of these sequences in other diseases. Understanding these interactions could lead to the development of novel therapeutic strategies against HSV-1 infections and other poly(T)-associated pathologies.
This work contributes to the current literature and brings new information to the field. Indeed, the work presented is original and provides important insights into a particular aspect of AIM2 inflammasome activation induced by different strains of HSV-1 both in vitro and in vivo. The authors have appropriately identified the work's hypothesis that the unique poly(T) DNA sequence of the HF strain interferes in AIM 2 inflammasome activation. The data are presented in a scientific manner, and all results are easy to interpret, making the scientific content easy to understand.
Instead, the information regarding materials and methods needs to be increased to make the procedures used clearer.

Specific comments to the authors are as follow:

1) Acronym should be explained as it’s the first time it appears in the text (AIM2/ASC/IL-1b/IL18/NLRP3/NLRC4/VP22 etc.).

2) The manuscript would benefit from a thorough review of the terminology used, specifically the correct usage of HSV-1 and caspase-1 to ensure accuracy and consistency throughout.

3) In the first paragraph of the results, especially the in vivo part (Fig. 1h, 1i and 1j) I would suggest remarking the site of infection in mice. Regarding the plaque assay I would suggest specifying the type of sample used (lung tissue) to facilitate the reader's understanding.
Also in the first paragraph, I would suggest specifying which major inflammasome sensor you tested.

4) I suggest clarifying any ambiguities in the methodology section, particularly regarding the techniques used for cytokine analysis (what samples did you use? What procedure did you perform to prepare the samples?), protein quantification in Western blots and immunoprecipitation.

5) Provide additional discussion on the potential clinical implications of your findings and avenues for future research in this field.

6) I observed that for some western blots housekeeping is shown (Figures 1j, 6d) in others it is not (Figures 1c;3f and 4c). Why?

7) Also, why is actin housekeeping represented in Figure 2c for both caspase 1 and GSDMD?
Furthermore, I suggest specifying the samples used for western blots in the supplementary materials images as well.

8) Finally, in most of the western blots shown in Supplementary Materials, the marker is not present. Can you show it?

Reviewer #2

(Remarks to the Author)
I co-reviewed this manuscript with one of the reviewers who provided the listed reports. This is part of the Nature Communications initiative to facilitate training in peer review and to provide appropriate recognition for Early Career Researchers who co-review manuscripts.

Reviewer #3

(Remarks to the Author)
AIM2 is a sensor of cytosolic double-stranded (ds)DNA that stimulates ASC- and caspase-1-dependent inflammasome activation and subsequent IL-1β and IL-18 secretion and pyroptosis to combat various pathogens, including herpes simplex virus 1 (HSV-1). It has been reported that, upon infection, HSV-1 dsDNA genomes activate AIM2-dependent inflammasomes, but the degree of activation during HSV-1 infection differs among HSV-1 strains. However, the detailed mechanism for the strain-specific differences remain uncertain. In this study, the authors addressed this question and found that the variation in poly(T) sequence length in the intergenic region of the HSV-1 genome was responsible for the differences in AIM2 inflammasome activation. They demonstrated that the presence of a longer poly(T) sequence in the viral genome robustly activated AIM2 inflammasomes in murine macrophages, and was linked to lower levels of pathogenicity and viral replication in mice in an AIM2-dpendent manner. This study presents some important and interesting findings regarding AIM2-dependent immune responses against HSV-1 infection. However, the experiments do not include critical controls and therefore, certain conclusions drawn by the authors lack sufficient evidence. More importantly, the mechanism by which the longer poly(T) sequence in the HSV-1 genome specifically activates AIM2 inflammasomes during viral infection remains to be addressed.

Major Comments:

Comment 1: The cited literature (PMID: 22483801) reported that the HIN domain of AIM2, which is responsible for the binding to ligand DNAs, interacts with DNAs containing 0%, 30%, 45%, and 90% GC content with similar efficiency. Additionally, various types of DNAs, including poly(dG:dC), have been shown to efficiently activate the AIM2-dependent inflammasome (PMID: 20457908). Experimental data elucidating the molecular mechanism by which the longer poly(T) sequence specifically serves as a crucial ligand for AIM2 are required. Knowledge of this mechanism would contribute to a conceptual advance in the DNA recognition of sensor proteins in innate immunity.

Comment 2: To properly interpret the data presented in Fig. 1a–g, the efficiencies of HSV-1 HF, HSV-1 F, and HSV-1 KOS infection in BMDMs need to be shown. The authors should analyze the expression levels of representative immediate-early, early, and late HSV-1 proteins in these infected cells at various times after infection. Moreover, the authors should examine the expression levels of VP22, which has been reported to inhibit AIM2 inflammasome activation, in infected cells at various times after infection.

Comment 3: Fig. 2 a–g. The effects of AIM2 knock-out on the expression of VP22 in BMDMs infected with each of the HSV-1 strains should be analyzed.

Comment 4: Fig. 3 c–e. Based on these results, the authors concluded that VP22 did not inhibit HSV-1-HF-induced AIM2 inflammasome activation. However, the data do not include the expression levels of VP22 in transfected BMDMs, and therefore, it is uncertain whether sufficient VP22 was expressed in these cells. The authors should analyze whether transfection with the VP22 expression vector increased the levels of VP22 in the cells. Moreover, the authors should examine the effects of the VP22-null mutation on AIM2 inflammasome activation (caspase-1 cleavage, IL-18, and IL-1β secretion, and cell death) in BMDMs using VP22-null mutant HSV-1 HF together with VP22-null mutant HSV-1 F or KOS as a control.

Comment 5: The authors should show whether the 23-mer poly(T) sequence is conserved in other laboratory strains and clinical isolates of HSV-1. Genetic modification (i.e., mutations associated with syncytial phenotype) sometimes artificially occurs during serial passages in cell cultures. How did the authors eliminate this possibility for the 23-mer poly(T) sequence in the HF strain?

Comment 6: Characterization of HFdelata14 is required. The authors should examine the effects of deltaT14 on viral replication, infection efficiency, the expression levels of neighboring genes (UL20 and UL21), and VP22 expression levels in BMDMs. To eliminate the off-target effect(s) of CRISPR/Cas9-mediated genome editing, the authors should construct a recombinant virus in which the mutation in HFdelta14 is repaired and test it together with wild-type and HFdelta14 viruses in the experiments shown in Fig. 6 and Extended Data Fig. 4.

Comment 7: To further verify the authors' conclusion that the length of the poly(T) sequence in the intergenic region of HSV-1 genome is critical for AIM2 inflammasome activation during HSV-1 infection, the authors should construct a recombinant

virus derived from HSV-1 F or a KOS strain harboring the 27-mer poly(T) sequence in the intergenic region, as well as its repaired virus, and test them together with wild-type HSV-1 F or KOS in the experiments in Fig. 6 and Extended Data Fig. 4, after characterization of the recombinant viruses, as described in Comment 6.

Comment 8: All analyses were conducted using mouse-derived cells. Since HSV-1 is a human pathogen, the authors should perform representative experiments shown in Figs. 2–5 and Extended Data Fig. 4 using human-derived cells.

Minor Comments

Comment 1: The ASC-citrine specks in Fig. 1a and Fig. 2a are unclear. Enlarged or higher-resolution images should be provided to clearly delineate the ASC-citrine specks.

Comment 2: The authors should disclose detailed information about the VP22 expression plasmid, the gRNA sequences, and the names of the gRNA expression plasmids for the knockout iBMDMs, as well as sequence information for each gene after genome editing.

Comment 3: The infection and transfection conditions in the experiments shown in Figs. 1 to 3 should be provided in more detail. What multiplicity of infection was used? When were the samples harvested? When were the BMDMs transfected with the VP22-expression plasmid?

Comment 4: In Extended Data Fig. 3a, the numbers (1)–(3) should be (6)–(8)?

Reviewer #4

(Remarks to the Author)
In the present work Oh et al study the inflammasome activation by Herpes Simplex Virus 1 strains in Mice and BMDMs and identify a poly(T) sequence of the HF strain that specifically activate Aim2. Although it is well established that dsDNA induce Aim2 activation, and that the Aim2 protein per se could have relatively broad specificity for different dsDNA, this study emphasis that in the cellular context the situation is very much more controlled and that (as far as I can see) the poly(T) sequence constitutes the first HSV1 sequence identified that can drive activation of innate immunity through Aim2. Due to this, and when our understanding of the role of Aim2 in virus immunity is still quite fragmented, I feel this work could be well suited for publication in Nature communication.

Overall, the study comprise quite comprehensive data and although the methodology used (knockdowns, IP, etc) is often open for multiple possible interpretations of what is going on in the cellular context, it appears as the data support most of their conclusions. However, I have few specific concerns that I feel should be addressed:

- Several readout assays for Aim2 induction are used through the study, mainly Il1b/il18 release, Casp1 cleavage and cell death which in most cases appears to support a textbook view of Aim2 activation and signaling. However, the knockdown experiments shown in Extended Data Fig 2 of proteins that could be involved downstream of Aim2 activation is poorly followed up and the apparent observation that cell death is not dependent on Casp1 or GSDMD/E is intriguing and would contradict the textbook view. In a HSV1 infection (and the transfection of full virus DNA) it is quite possible that multiple pathways are activated and, although it is clear that Aim2 signaling is one of these, there might be other pathways contributing to e.g. to cell death and Il1b/Il18 release in parallel (Yes, the Aim2 KD support that Aim2 is a key player, but these are KD clones with all the possible caveats, where compensatory and complex distortions of immune signaling might have been induced due by KD of Aim2). The Casp1 or GSDMD KD data is confusing and it is also possible that these knockdown experiments have not worked well/are not informative (where are the WBs showing that these proteins are indeed knocked down?). I feel the authors should add some further follow-up experiment on at least the Casp1 knockdown to show whether IL1b/Il18 release is attenuated in the BMDMs knockdown as expected. Overall, the mechanistic discussions are very sparse and the work would benefit from the authors further develop there point on e.g. RIPK3 dependence in the discussion and potentially also add clarifying experiments. Can it be excluded that there is a significant Toll receptor effect contributing to Il1b/Il18 release or apoptosis? Etc

- The section addressing a potential role of VP22 is an inhibitor of Aim2 is not very crisp and the experiments might eventually not be informative. The initial paragraph contains a somewhat superficial discussion of structural regions and what could be functionally significant. In the end of a paragraph, they say “Variants outside of this core domain are thus not expected to significantly impact VP22 functions and are consequently categorized as functionally neutral”. Although e.g. Aints et al addresses other types of functions, I cannot really find the data excluding that other regions could have additional functions, e.g. in the context of Aim2 inhibition. Although these regions might be intrinsically disordered, they could make important interactions. This paragraph should be rewritten, e.g. to instead just describe what is known about the structure (if the authors do not know about more crisp data excluding functional significance of specific regions).

- It is concluded from the VP22 over-expression experiment that there is no differential effect of VP22 for the different strains. However, there is no positive control experiment showing that the VP22 overexpression in this system can inhibit Aim2. Therefore, I would like to see a control experiment demonstrating that VP22 overexpression can indeed inhibit Aim2 activation, e.g. with a highly potent dsDNA for Aim2 activation. Otherwise, there is no use addressing differential effects.

- In the IP figures (e.g. Fig2 F), in the Input chart, there is no Aim2 protein present for the WT media lane. This suggests that

the virus first induce Aim2, which is subsequently activated by viral dsDNA (?). How is Aim2 induced in this context? It would be particuliarly interesting to shed light on how this is accomplished for HF seq#5 when this is a requirement for Aim2 activation.

- I feel the Discussion section is quite thin, and in addition to above points on the lack of discussion of mechanistic implications, it should be possible to further address the implications of the poly(T) sequence for virus immune response. Are there other Herpesviruses, other viruses, containing similar sequences. This sequence is relative short in relationship to some other Aim2 inducing sequences, etc

Version 1:

Reviewer comments:

Reviewer #1

(Remarks to the Author)
The revised manuscript presents compelling genetic and functional evidence that viral poly(T) DNA length polymorphisms determine strain-specific AIM2 inflammasome activation during HSV-1 infection. The inclusion of precise deletion, reversion, and insertion mutants, together with in vivo validation and confirmation in human macrophages, substantially strengthens the mechanistic framework and causal inference.
The study is carefully designed, technically rigorous, and conceptually significant. I have only minor suggestions aimed at further improving clarity and transparency:

Clarification of DNA length context
The manuscript demonstrates that efficient AIM2 activation requires dsDNA above a length threshold (~≥80 bp), while the HF strain contains a 27-mer poly(T) tract embedded within genomic DNA. A brief clarification in the Discussion explaining how the genomic context provides sufficient DNA length for AIM2 oligomerization would further strengthen mechanistic clarity.

Statistical reporting consistency
Please ensure that exact sample sizes (n), number of biological replicates, and statistical tests are consistently reported in all figure legends, including Extended Data figures.

Immunoblot presentation
Where feasible, include molecular weight markers or indicate expected molecular weights in figure legends for key immunoblots to enhance transparency.

These are presentation-level refinements and do not affect the central conclusions.

Overall, this is a strong and well-supported study that provides important insights into viral genome–encoded determinants of innate immune sensing.

Reviewer #2

(Remarks to the Author)
I co-reviewed this manuscript with one of the reviewers who provided the listed reports. This is part of the Nature Communications initiative to facilitate training in peer review and to provide appropriate recognition for Early Career Researchers who co-review manuscripts.

Reviewer #3

(Remarks to the Author)
The authors have performed an extensive and impressive set of additional experiments, and the revised manuscript is substantially strengthened compared to the original submission.

I am largely satisfied with the authors' responses and the revised manuscript, and I support its publication in Nature Communications after minor revision. I have two suggestions that would further improve the clarity and presentation of the work.

First, in the revised Fig. 3b, the authors have added Western blotting data to confirm VP22 expression following transient overexpression. While this demonstrates that VP22 protein is produced, western blotting has inherent limitations in this experimental context, as it cannot verify whether the VP22-expressing cells are identical to the productively infected cells. Given that the authors have now successfully generated and analyzed genetically defined VP22-deficient viruses in both the HF and F strain backgrounds, the transient VP22 overexpression data (Fig. 3b–e) are of reduced central importance. In contrast, the ΔVP22 virus data provide more direct and physiologically relevant evidence regarding the strain-dependent requirement of VP22. I therefore suggest moving the current Fig. 3b–e to the Extended Data section. Conversely, since one

of the key conclusions of this study is the differential role of VP22 in HF versus F strains, the data for the F strain ΔVP22 mutant (currently shown in Extended Data Fig. 5e–g) would be more impactful if included in the main Figure 3. This rearrangement would facilitate direct comparison between strains and further clarify the distinct roles of VP22 in each viral context.

Second, although the authors’ large-scale genomic analyses strongly support the presence of long poly(T) tracts in both laboratory strains and clinical isolates, many HSV-1 genome sequences deposited in public databases have undergone cell-culture passage. As such, statements describing these long poly(T) motifs as strictly “naturally occurring” may be somewhat overstated. While the data are clearly consistent with their presence in circulating viral populations, it remains difficult to completely exclude a contribution from cell-culture adaptation. I therefore recommend slightly softening the wording in the relevant sections (e.g., pages 14–15 of the revised manuscript), using phrasing such as “consistent with naturally occurring variation” or “present in both clinical and laboratory isolates,” to avoid over-interpretation without diminishing the impact of the findings.

With these minor revisions, I believe the manuscript will be suitable for publication.

Open Access This Peer Review File is licensed under a Creative Commons Attribution 4.0 International License, which permits use, sharing, adaptation, distribution and reproduction in any medium or format, as long as you give appropriate credit to the original author(s) and the source, provide a link to the Creative Commons license, and indicate if changes were made.
In cases where reviewers are anonymous, credit should be given to 'Anonymous Referee' and the source.
The images or other third party material in this Peer Review File are included in the article’s Creative Commons license, unless indicated otherwise in a credit line to the material. If material is not included in the article’s Creative Commons license and your intended use is not permitted by statutory regulation or exceeds the permitted use, you will need to obtain permission directly from the copyright holder.
To view a copy of this license, visit https://creativecommons.org/licenses/by/4.0/

We greatly appreciate the time and expertise the four reviewers have contributed to the peer review process. We are grateful for the constructive feedback which has substantially improved our manuscript.

In the revised version of our manuscript, we have carefully considered and thoroughly addressed all constructive comments raised by the reviewers. We have worked extremely hard to generate a tremendous amount of new insightful data and to improve the quality of our manuscript and strengthen our conclusions following the expert suggestions of the reviewers. We have listed below new data that we have generated for the revised manuscript. These new results show that:

- **Poly(dG:dC) transfection in BMDMs induces inflammasome activation, evidenced by caspase-1 cleavage and IL-1β secretion, validating previous evidence of AIM2 responsiveness to GC-rich DNA.**
- **All HSV-1 genomes analyzed (HF, F, KOS) contain poly(G:C) tracts ≤16 bp, a length commonly found in HSV-1 strains and insufficient to explain strain-specific AIM2 activation, particularly in HF.**
- **Only 90-mer poly(T) DNA, but not 10-mer or 30-mer, induces robust caspase-1 activation and IL-1β release, establishing a length-dependent mechanism of AIM2 activation likely mediated by higher-order DNA structures.**
- **All three HSV-1 strains (HF, F, KOS) can successfully infect HaCaT and BMDM cells, with comparable expression of viral proteins (ICP0, ICP8, gD, gC, and VP22), ruling out infection efficiency as a variable in AIM2 activation.**
- **Successful VP22 overexpression was directly validated by immunoblotting, excluding insufficient protein expression as a reason for the lack of inhibitory activity.**
- **A VP22-null mutant virus in the HF background (HF ΔVP22) shows no difference in inflammasome activation compared with WT HF, as measured by caspase-1 cleavage and IL-1β/IL-18 secretion, demonstrating that VP22 is dispensable in this context.**
- **In the HSV-1 F strain background, deletion of VP22 (F ΔVP22), but not WT F infection, restores robust AIM2-dependent inflammasome activation, including caspase-1 cleavage and IL-1β/IL-18 secretion, demonstrating that VP22 acts as a strain-specific inhibitor of AIM2 in F but not in HF, where poly(T)-mediated activation predominates.**
- **Analysis of 492 public HSV-1 genomes revealed that the 23-mer poly(T) sequence is present in both clinical and laboratory isolates, including H166, KOS63, and multiple patient-derived strains, confirming it is not a cell-culture artifact.**
- **The 14-mer poly(T) sequence is found in 15% of clinical HSV-1 genomes, and the 23-mer in 2.5%, supporting poly(T) length variation as a natural polymorphism among HSV-1 strains.**
- **Cross-species viral genome analysis shows that 23-mer poly(T) sequences are found in multiple DNA and RNA viruses, including Monkeypox virus, adenovirus, Hepacivirus, and SARS-CoV, indicating conserved innate immune relevance.**

- **WT HF, HF ΔT_{14}, and HF ΔT_{14}-Rev viruses exhibit similar expression of viral proteins (ICP0, ICP8, gD, gC, VP22) and neighboring genes UL25/UL26, confirming the poly(T) deletion does not affect viral fitness or local transcriptional context.**
- **Reversion of ΔT_{14} mutation (HF ΔT_{14}-Rev) restores inflammasome activation and host protection *in vivo*, including survival, lung viral titers, caspase-1 activation, and cytokine responses, all lost in the ΔT_{14} mutant and absent in AIM2-deficient mice.**
- ***In vitro*, HF ΔT_{14}-Rev infection restores caspase-1 cleavage, IL-1β/IL-18 secretion, and pyroptosis in BMDMs, mirroring WT HF responses and confirming strict AIM2 dependence for poly(T)-mediated inflammasome activation.**
- **F strain harboring a 27-mer poly(T) insertion (F T_{27}) induces inflammasome activation and improves survival in WT mice, but not in AIM2 KO mice, confirming sufficiency and AIM2 dependence of the poly(T)–mediated response.**
- **F T_{27} infection activates caspase-1 and cytokine release in WT BMDMs, but not in AIM2 KO BMDMs, and shows no differences in viral protein expression across F, F T_{27}, and F T_{27} revertant strains, ruling out replication artifacts.**
- **In human THP-1 macrophages, AIM2 knockdown abolishes cell death in response to HF and F T_{27} infections, validating conservation of poly(T)-AIM2 sensing in human innate immunity.**
- **VP22 does not suppress AIM2-dependent responses in human THP-1 cells, consistent with murine data, and deletion of poly(T) in HF abrogates AIM2-dependent cell death, rescued by the revertant.**
- **Insertion of poly(T) in F (F T_{27}) enables cell death in human cells, which is completely lost upon AIM2 silencing, confirming poly(T) sufficiency and AIM2 specificity.**
- **MYD88 KO and TRIF KO iBMDMs show no difference in HSV-1 HF-induced cell death, excluding significant contribution from TLR pathways in the observed inflammasome response.**
- **cGAS, STING, and IRF1 knockout cells exhibit impaired AIM2-dependent cell death, supporting a cGAS–STING–IRF1–AIM2 axis in viral sensing.**

We would also like to highlight that our original manuscript has already demonstrated that:

- **HSV-1 induced inflammasome activation is dependent on the HF strain of HSV-1.**
- **AIM2 is identified as an inflammasome sensor during HSV-1 infection.**
- **Inflammasome sensors, including NLRP3 and NLRC4, do not play a role in activation or cell death during infection.**
- **AIM2-dependent cell death during HSV-1 infection is characterized by pyroptotic markers (CASP1, GSDMD), apoptotic markers (CASP8, CASP3, CASP7), and necroptotic markers (MLKL), indicating inflammatory cell death.**
- **Deletion of cell death executioner molecules, including RIPK3 and CASP8, but not CASP1, CASP3, CASP6, CASP7, CASP9, GSDMD, GSDME, and MLKL, is sufficient to protect cells from death induced by HSV-1 infection.**
- **Previously known AIM2 inflammasome inhibitors such as the viral protein VP22 do not play a role in inflammasome activation or cell death during HSV-1 strain HF infection.**

- **Viral DNA of HSV-1 strain HF, but not viral DNA of strain KOS, is sufficient to induce inflammasome activation and cell death.**
- **The unique regions of the 14-mer poly(T) DNA sequence of the HF strain are identified within the HSV-1 HF, F, and KOS viral DNA sequences.**
- **The mutant HSV-1 strain HF, with the 14-mer poly(T) DNA sequence deleted (HF ΔT_{14}), is insufficient to induce inflammasome activation and cell death.**
- **The 14-mer poly(T) DNA sequence is critical for AIM2 sensing and host defense against HSV-1 infection.**
- **WT mice succumb to HF ΔT_{14} HSV-1 infection within 11 days, while 80% of mice survive against WT HSV-1.**
- **Mice lacking AIM2 succumb to both WT and mutant HSV-1 infection within 9 and 10 days, respectively.**

Overall, our study uncovers the HSV-1 ligand for AIM2 inflammasome sensor but also establishes the molecular basis of how this ligand can trigger the AIM2 inflammasome for host defense. We firmly believe that the large amount of new data added responds to all of the reviewers' expert comments thoroughly and greatly improves the quality of our manuscript. We have provided substantial new mechanistic data and multiple lines of evidence pointing toward the prominent role of a critical interaction between AIM2 and the Poly(T) DNA sequence. These findings represent a new paradigm in understanding the functions of these molecules in inflammasome and innate immunity, further strengthening the central conclusions of our manuscript.

Reviewer #1 (Remarks to the Author):

This study focuses on the differences in AIM2 inflammasome activation among HSV-1 strains, revealing that the HF strain strongly stimulates the inflammasome, whereas the F and KOS strains show minimal activation. In this work, genomic analysis identified unique 14 bp poly(T) DNA sequences in the HF strain, located between the UL25 and UL26 genes, as critical for AIM2 inflammasome activation. Removing these sequences from the HF strain confirmed their importance, showing reduced inflammasome activation and compromised host defense. Transfection experiments with various lengths of poly(T) DNA further suggested that longer sequences are essential for AIM2 activation. These interesting findings not only clarify the role of poly(T) sequences in AIM2 inflammasome activation during HSV-1 infection but also suggest broader pathological implications of these sequences in other diseases. Understanding these interactions could lead to the development of novel therapeutic strategies against HSV-1 infections and other poly(T)-associated pathologies.
This work contributes to the current literature and brings new information to the field. Indeed, the work presented is original and provides important insights into a particular aspect of AIM2 inflammasome activation induced by different strains of HSV-1 both in vitro and in vivo. The authors have appropriately identified the work's hypothesis that the unique poly(T) DNA sequence of the HF strain interferes in AIM 2 inflammasome activation. The data are presented in a scientific manner, and all results are easy to interpret, making the scientific content easy to understand.
Instead, the information regarding materials and methods needs to be increased to make the procedures used clearer.

We sincerely thank you for your thoughtful and generous evaluation of our work. We are especially grateful for the recognition that our study "brings new information to the field" and "provides important insights into a particular aspect of AIM2 inflammasome activation." We also appreciate your positive remarks on the clarity and scientific presentation of our data.

We have carefully addressed your comment regarding the need for more detailed methodological information. In response, we have significantly expanded the Materials and Methods section to provide clearer descriptions of experimental procedures, including infection conditions, transfection timing, and gene editing protocols. Additional details have also been included in the figure legends and supplementary materials to enhance reproducibility.

We are truly grateful for your constructive feedback, which has helped us improve both the scientific rigor and clarity of our manuscript.

Specific comments to the authors are as follow:

1) Acronym should be explained as it's the first time it appears in the text (AIM2/ASC/IL-1b/IL18/NLRP3/NLRC4/VP22 etc.).

We have now defined all acronyms at their first appearance in the text. For example, AIM2 is introduced as "absent in melanoma 2 (AIM2)," and similarly ASC, IL-1β, IL-18, NLRP3, NLRC4, and VP22 are all spelled out at their initial appearance. This ensures that readers understand each term when it is first mentioned.

2) The manuscript would benefit from a thorough review of the terminology used, specifically the correct usage of HSV-1 and caspase-1 to ensure accuracy and consistency throughout.

We thoroughly reviewed the manuscript for consistency in terminology. We have corrected instances of inconsistent notation – for instance, we have now uniformly used "HSV-1" (with a hyphen) and "caspase-1" throughout the text. All virus strain names and gene/protein names are presented with correct and consistent formatting.

3) In the first paragraph of the results, especially the in vivo part (Fig. 1h, 1i and 1j) I would suggest remarking the site of infection in mice. Regarding the plaque assay I would suggest specifying the type of sample used (lung tissue) to facilitate the reader's understanding.
Also in the first paragraph, I would suggest specifying which major inflammasome sensor you tested.

Infection Route (*In vivo* Experiments): We have clarified the site of infection in our *in vivo* experiments. The revised Results section now explicitly states that mice were intranasally infected with HSV-1 (5×10^5 PFU in 100 μL, as described in the Methods). This additional detail makes the infection route clear for the experiments associated with Figure 1h–j.

Plaque Assay Sample: We have specified the sample source for the plaque assays in the text. In the Results, we now note that the viral titers (Figure 1h) were determined using lung tissue homogenates collected from infected mice. Stating that lung tissue was used for the plaque assay helps the reader understand the origin of the samples for viral load measurements.

Inflammasome Sensor Tested: We have identified the major inflammasome sensor examined in our study to avoid ambiguity. In the revised first paragraph of the Results, we clearly state that we focused on the AIM2 inflammasome pathway. For example, we mention that experiments were performed in AIM2-knockout macrophages to confirm AIM2's role in HSV-1–induced inflammasome activation (shown in Fig. 2a, b). This clarification makes it explicit that AIM2 is the primary sensor being tested in our comparisons.

4) I suggest clarifying any ambiguities in the methodology section, particularly regarding the techniques used for cytokine analysis (what samples did you use? What procedure did you perform to prepare the samples?), protein quantification in Western blots and immunoprecipitation.

Additional details have been added to the Methods to clarify how cytokine levels were measured. We now specify the sources of samples and preparation steps for IL-1β and IL-

18 assays. For instance, the Methods section now explains that cell culture supernatants from infected bone-marrow-derived macrophages were collected and centrifuged to remove debris before IL-1β and IL-18 quantification. In the case of *in vivo* experiments, we now note that bronchoalveolar lavage fluid (BALF) from infected mice was harvested for cytokine measurements. These samples were then analyzed by ELISA following the manufacturer's protocols. These clarifications ensure that the procedures for cytokine analysis are transparent and reproducible.

Additional details have been added to the Methods to clarify protein quantification procedures for Western blotting and immunoprecipitation. For *in vitro* immunoblot analyses, cells were seeded at fixed densities (1×10^6 cells per well for BMDMs and iBMDMs, and 1×10^5 cells per well for THP-1 cells), and lysates were prepared from the entire well. Because equal numbers of cells were processed under each condition, additional protein concentration–based normalization was not performed. Instead, equal volumes of lysates were loaded for SDS–PAGE, and protein loading consistency was verified using internal control proteins, including β-actin or GAPDH.

For *in vivo* immunoblot analyses, approximately 100 mg of lung tissue was harvested from infected mice and homogenized in cold RIPA buffer. Tissue lysates were clarified by centrifugation, and protein concentrations were determined using a BCA protein assay. Lysates were then adjusted to a final concentration of 2 mg/mL with the addition of RIPA buffer, and equal amounts of total protein were loaded for SDS–PAGE followed by immunoblotting with the indicated antibodies.

For immunoprecipitation experiments, cells were seeded at a density of 1×10^7 cells per condition and infected as indicated. After infection, cells were lysed in ice-cold lysis buffer, and lysates were clarified by centrifugation to remove insoluble debris. Protein concentrations were quantified using a BCA protein assay, and lysates were subsequently normalized to a final concentration of 1 mg/mL total protein. Equal amounts of total protein were used for each immunoprecipitation reaction, followed by incubation with the indicated antibodies and protein A/G beads under identical conditions. These procedures ensured equivalent protein input across samples and enabled reliable comparison of immunoprecipitated complexes. Together, these revisions clarify the procedures for cytokine analysis, protein quantification in Western blotting, and immunoprecipitation, ensuring that all experimental methods are described in a transparent and consistent manner so that they are reproducible.

5) Provide additional discussion on the potential clinical implications of your findings and avenues for future research in this field.

Thank you for this insightful suggestion regarding the clinical implications of our findings and directions for future research. In the revised Discussion section, we have expanded our interpretation of the data to explicitly address these points.

Specifically, we now discuss the translational relevance of identifying viral poly(T) DNA as a dominant AIM2 ligand. We highlight that selective modulation of DNA–inflammasome interactions, such as targeting poly(T)-rich genomic regions or downstream AIM2-dependent signaling pathways, may represent a potential strategy to attenuate excessive inflammasome activation and immunopathology during severe HSV-1 infection, while preserving antiviral host defense. Conversely, we also outline the possibility that enhancing AIM2 responsiveness to such DNA motifs could be explored as a means to bolster protective immunity in immunocompromised cases.

In addition, we have expanded the Discussion to emphasize broader implications beyond HSV-1. We note that long poly(T) tracts are present not only in laboratory-adapted strains but also in multiple clinical HSV-1 isolates and across diverse viral families. This observation raises the possibility that similar DNA motifs may function as innate immune triggers in other viral or inflammatory contexts. Accordingly, we outline future research directions, including systematic profiling of poly(T)-rich genomic regions across viral genomes, investigation of how poly(T) length polymorphisms correlate with disease severity and immune outcomes *in vivo*, and exploration of AIM2-dependent sensing mechanisms in related viral infections.

Together, these additions broaden the clinical and conceptual context of our findings and clearly define avenues for future investigation, in accordance with your request.

6) I observed that for some western blots housekeeping is shown (Figures 1j, 6d) in others it is not (Figures 1c;3f and 4c). Why?

Thank you for pointing this out. In our revised submission, we have ensured consistency in our presentation of housekeeping controls. For all Western blots presented in the main and supplementary figures, β-actin or GAPDH was used as the housekeeping control and was consistently run and imaged. In cases where the original figure panels did not include the actin blots, we have now either provided the corresponding actin panels or explicitly clarified this in the figure legends and Supplementary Figures. We sincerely apologize for the oversight in initial figure assembly and have addressed it to improve transparency.

7) Also, why is actin housekeeping represented in Figure 2c for both caspase 1 and GSDMD? Furthermore, I suggest specifying the samples used for western blots in the supplementary materials images as well.

Thank you for this valuable comment regarding the use of β-actin as a housekeeping control in Figure 2c. In our study, different lysis buffers were used to extract caspase-1 and GSDMD proteins owing to their biochemical properties. Specifically, caspase-1 was extracted using CASP lysis buffer optimized for caspase activity and stability, whereas GSDMD was extracted using RIPA buffer, which is suitable for solubilizing membrane-associated proteins. As these two proteins were probed from separately prepared lysates, we appropriately used independent β-actin blots as internal loading controls for each

protein in Figure 2c. In the revised version of the manuscript, we have clarified this point in the figure legend to avoid confusion and to ensure transparency in data interpretation.

Furthermore, in response to your suggestion, we have now specified the sample types used for all Western blot experiments in the supplementary materials (e.g., whole-cell lysates from BMDMs, lung homogenates from infected mice, etc.). To further enhance clarity and traceability of sample identities (such as WT versus AIM2-deficient samples), we have additionally provided the corresponding uncropped original Western blot images alongside the cropped panels in the Supplementary Materials. These uncropped blots include full lane information and labeling, allowing unambiguous identification of each sample used in the presented figures. Together, these revisions improve clarity, reproducibility, and methodological rigor of the Western blot data.

8) Finally, in most of the western blots shown in Supplementary Materials, the marker is not present. Can you show it?

We completely understand your concern and fully agree that molecular weight markers are valuable for verifying protein band sizes in Western blotting. Unfortunately, owing to limitations of our current imaging system (Amersham ImageQuant 800 UV), which relies on chemiluminescence detection without integrated marker-lane annotation functionality, molecular weight markers were not captured in the final blot images. Although the system offers high sensitivity for detecting protein signals, it does not allow co-visualization of markers and targets in a single image without additional processing steps, which are technically challenging under our current experimental setup.

Nevertheless, we consistently used pre-stained molecular weight markers during electrophoresis for every blot. Band sizes were confirmed according to expected molecular weights and verified using specific antibodies, with knockout cell lines serving as negative controls to confirm antibody specificity and band identity. While the markers are not visible in the images, we ensured that size determination and validation were rigorously performed.

We hope this explanation clarifies the technical constraints and reassures the reviewer of the integrity and interpretability of the presented Western blot data.

Reviewer #2 (Remarks to the Author):

I co-reviewed this manuscript with one of the reviewers who provided the listed reports. This is part of the Nature Communications initiative to facilitate training in peer review and to provide appropriate recognition for Early Career Researchers who co-review manuscripts.

We appreciate your participation in the co-review process and the *Nature Communications* initiative to support Early Career Researchers. No further comments were provided in this section, so we thank you for your support.

Reviewer #3 (Remarks to the Author):

AIM2 is a sensor of cytosolic double-stranded (ds)DNA that stimulates ASC- and caspase-1-dependent inflammasome activation and subsequent IL-1β and IL-18 secretion and pyroptosis to combat various pathogens, including herpes simplex virus 1 (HSV-1). It has been reported that, upon infection, HSV-1 dsDNA genomes activate AIM2-dependent inflammasomes, but the degree of activation during HSV-1 infection differs among HSV-1 strains. However, the detailed mechanism for the strain-specific differences remain uncertain. In this study, the authors addressed this question and found that the variation in poly(T) sequence length in the intergenic region of the HSV-1 genome was responsible for the differences in AIM2 inflammasome activation. They demonstrated that the presence of a longer poly(T) sequence in the viral genome robustly activated AIM2 inflammasomes in murine macrophages, and was linked to lower levels of pathogenicity and viral replication in mice in an AIM2-dpendent manner. This study presents some important and interesting findings regarding AIM2-dependent immune responses against HSV-1 infection. However, the experiments do not include critical controls and therefore, certain conclusions drawn by the authors lack sufficient evidence. More importantly, the mechanism by which the longer poly(T) sequence in the HSV-1 genome specifically activates AIM2 inflammasomes during viral infection remains to be addressed.

We sincerely thank the you for your thoughtful and critical evaluation of our manuscript. We greatly appreciate your recognition that our study presents "important and interesting findings regarding AIM2-dependent immune responses against HSV-1 infection" and that it provides new insight into the strain-specific differences in inflammasome activation driven by HSV-1 genomic variation.

We fully agree that a more detailed mechanistic understanding of how longer poly(T) sequences specifically activate AIM2 inflammasomes is of great importance, and we have made efforts to address this in the revised manuscript. Additionally, we have conducted further experiments to include missing controls and strengthen the evidence for our conclusions, as suggested.

Your constructive comments have helped us significantly refine the depth, clarity, and rigor of our study, and we are grateful for your valuable input.

Major Comments:

Comment 1: The cited literature (PMID: 22483801) reported that the HIN domain of AIM2, which is responsible for the binding to ligand DNAs, interacts with DNAs containing 0%, 30%, 45%, and 90% GC content with similar efficiency. Additionally, various types of DNAs, including poly(dG:dC), have been shown to efficiently activate the AIM2-dependent inflammasome (PMID: 20457908). Experimental data elucidating the molecular mechanism by which the longer poly(T) sequence specifically serves as a crucial ligand for AIM2 are required. Knowledge of this mechanism would contribute to a conceptual advance in the DNA recognition of sensor proteins in innate immunity.

We greatly appreciate your insightful question regarding the DNA sequence specificity of AIM2 activation and the relevance of our poly(T) findings in light of prior reports showing GC-content-independent binding of the AIM2 HIN domain (PMID: 22483801) and poly(dG:dC)-mediated inflammasome activation (PMID: 20457908).

To address this, we performed additional comparative experiments. We transfected BMDMs with poly(dG:dC) duplexes and confirmed that they indeed induce caspase-1 cleavage and IL-1β secretion, consistent with previous findings (New Data, Extended Data Figure 6e, 6f [shown below]). However, when comparing the HSV-1 HF strain to the F and KOS strains, we noted that all three genomes contained poly(G:C) tracts of 16 nucleotides or fewer, which are commonly found across HSV-1 strains and are not enriched in HF. Importantly, poly(G:C) sequences longer than 16 nucleotides were not detected in any variant, making them unlikely candidates for explaining HF-specific AIM2 activation.

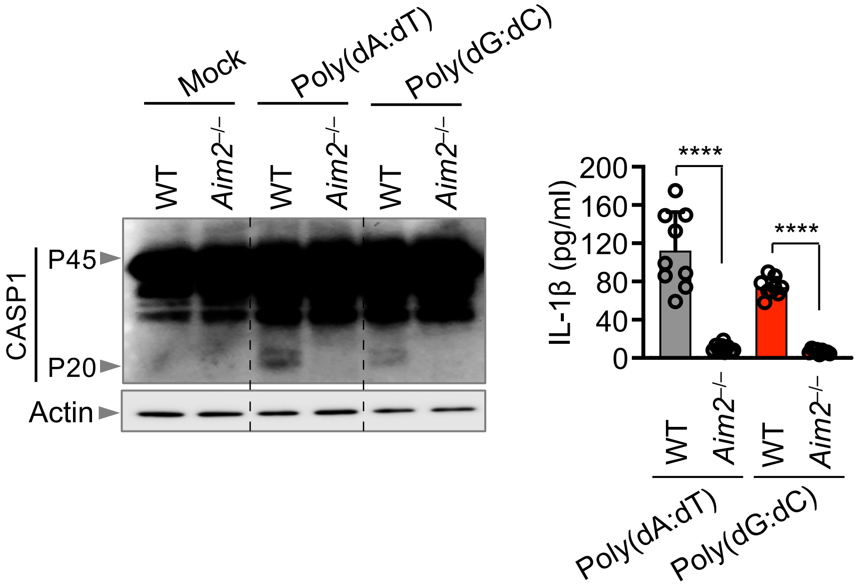

In contrast, we identified a unique 27-mer poly(A:T) sequence, especially poly(T), in the HF strain, which was absent or significantly shorter in the F and KOS strains. This sequence variation led us to hypothesize that poly(A:T), particularly poly(T), contributes to the strain-specific AIM2 activation observed.

To test this, we synthesized double-stranded poly(T) DNA of 10-, 30-, and 90-nucleotide lengths and transfected them into BMDMs. Robust caspase-1 activation and IL-1β release were observed only with the 90-mer poly(T), whereas the 10-mer and 30-mer failed to induce significant caspase-1 cleavage or IL-1β release (New Data, Extended Data Figure 6g, 6h [shown below]). This finding is consistent with a previous study (PMID: 22483801), which demonstrated that double-stranded DNA of at least 80 base pairs is required for efficient AIM2 inflammasome activation. These results suggest that poly(T)-mediated AIM2 activation is highly length-dependent, likely reflecting the requirement for higher-order DNA structures that facilitate AIM2 oligomerization and inflammasome assembly.

Collectively, these findings support the idea that while AIM2 can indeed respond to various DNA ligands *in vitro*, in the cellular context of HSV-1 infection, the presence of a uniquely long poly(T) stretch in the HF genome serves as a potent and physiologically relevant AIM2 ligand, capable of overcoming viral immune evasion mechanisms and triggering a stronger inflammasome response. We have included these new data in the revised Results and believe they help clarify the specificity and significance of the poly(T) motif in our study.

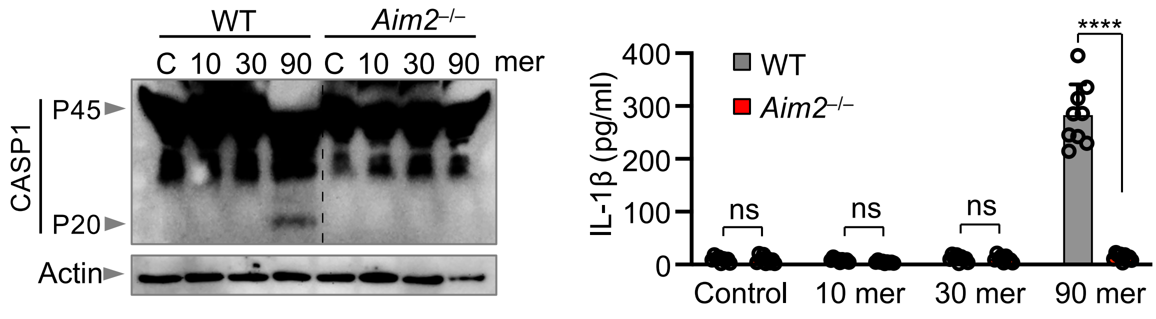

Comment 2: To properly interpret the data presented in Fig. 1a–g, the efficiencies of HSV-1 HF, HSV-1 F, and HSV-1 KOS infection in BMDMs need to be shown. The authors should analyze the expression levels of representative immediate-early, early, and late HSV-1 proteins in these infected cells at various times after infection. Moreover, the authors should examine the expression levels of VP22, which has been reported to inhibit AIM2 inflammasome activation, in infected cells at various times after infection.

Thank you for this important suggestion. To evaluate and compare the infection efficiencies of HSV-1 HF, F, and KOS strains, we performed time-course infection experiments in HaCaT cells, a human keratinocyte line relevant to HSV-1 pathogenesis. Cells were infected with each strain at the same multiplicity of infection (MOI = 3), and samples were collected at multiple time points (3, 6, 9, and 12 hours post-infection). We then assessed the expression of representative HSV-1 viral proteins across different phases of the viral life cycle using immunoblotting: ICP0, ICP8, gD, gC, and VP22.

Our results showed that all three viral strains (HF, F, and KOS) successfully infected both HaCaT cells, with comparable expression levels of ICP0, ICP8, gD, gC, and VP22 over time. The kinetics and intensity of viral protein expression were similar across strains, suggesting that differences in AIM2 activation observed in our study were not due to variations in viral entry, replication efficiency, or protein expression levels.

In addition, to directly address your concern in a macrophage system relevant to inflammasome activation, we performed parallel infection experiments using primary wild-type (WT) and *Aim2*$^{-/-}$ bone marrow–derived macrophages (BMDMs). Primary WT and *Aim2*$^{-/-}$ BMDMs were infected with HSV-1 HF, F, or KOS strains at a high multiplicity of infection (MOI = 10), and viral protein expression was analyzed at 12 hours post-infection.

Immunoblot analysis demonstrated that representative immediate-early (ICP0), early (ICP8), and late (gD and VP22) viral proteins were expressed at comparable levels across all three HSV-1 strains in both WT and *Aim2*$^{-/-}$ BMDMs. These results indicate that, even under conditions optimized for inflammasome activation in primary macrophages, HSV-1 HF, F, and KOS establish infection with similar efficiency and comparable viral protein expression profiles. Therefore, the strain-specific differences in AIM2 inflammasome activation observed in our study cannot be explained by differential viral infection efficiency or viral protein expression in BMDMs.

Importantly, VP22 was robustly expressed in all three strains and at all examined time points, indicating that the differential inflammasome activation cannot be attributed to the absence or delay in VP22 expression in the F or KOS strains.

These new data have been included in the revised manuscript (New Data, Extended Data Figure 1a, 1b [shown below]) and described in the Results section. We believe this analysis addresses your concern and reinforces the conclusion that the enhanced AIM2 activation by the HF strain is due to its unique poly(T) DNA content rather than differences in infection efficiency.

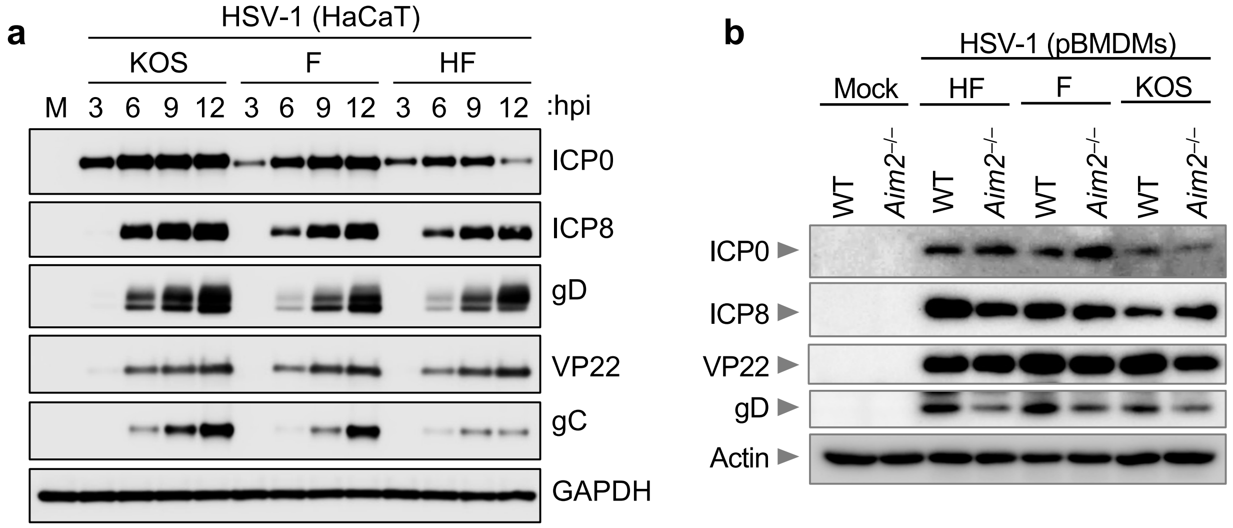

Comment 3: Fig. 2 a–g. The effects of AIM2 knock-out on the expression of VP22 in BMDMs infected with each of the HSV-1 strains should be analyzed.

Thank you for raising this important point. To address your concern regarding the effect of AIM2 deficiency on VP22 expression, we performed parallel infection experiments using primary wild-type (WT) and *Aim2*$^{-/-}$ bone marrow–derived macrophages (BMDMs). Primary WT and *Aim2*$^{-/-}$ BMDMs were infected with HSV-1 HF, F, or KOS strains at a high multiplicity of infection (MOI = 10), and viral protein expression was analyzed at 12 hours post-infection. Immunoblot analysis revealed that the expression levels of VP22 were

comparable between WT and *Aim2*$^{-/-}$ BMDMs for all three HSV-1 strains. Consistently, other representative viral proteins, including the immediate-early protein ICP0, the early protein ICP8, and the late protein gD, also showed similar expression patterns irrespective of AIM2 status. These results indicate that AIM2 deficiency does not affect VP22 expression during HSV-1 infection in BMDMs. Therefore, the differences in AIM2 inflammasome activation observed in our study cannot be attributed to altered VP22 expression resulting from AIM2 knock-out, but rather reflect downstream sensing of viral ligands.

These new data have been included in the revised manuscript (New Data, Extended Data Figure 1b [shown below]) and described in the Results section.

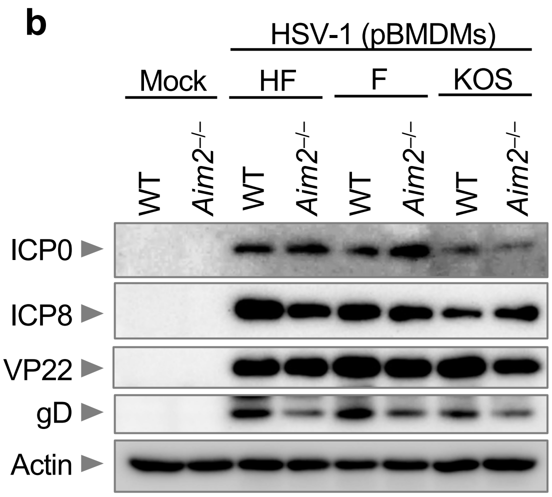

Comment 4: Fig. 3 c–e. Based on these results, the authors concluded that VP22 did not inhibit HSV-1-HF-induced AIM2 inflammasome activation. However, the data do not include the expression levels of VP22 in transfected BMDMs, and therefore, it is uncertain whether sufficient VP22 was expressed in these cells. The authors should analyze whether transfection with the VP22 expression vector increased the levels of VP22 in the cells. Moreover, the authors should examine the effects of the VP22-null mutation on AIM2 inflammasome activation (caspase-1 cleavage, IL-18, and IL-1β secretion, and cell death) in BMDMs using VP22-null mutant HSV-1 HF together with VP22-null mutant HSV-1 F or KOS as a control.

We appreciate this thoughtful and constructive comment. To address the first point, we performed immunoblotting to confirm VP22 expression following transfection of the VP22 expression vector. In mock-infected BMDMs, VP22 was expressed at detectable levels upon transfection, confirming successful overexpression. Moreover, subsequent infection with HSV-1 HF did not further enhance VP22 expression. Importantly, VP22 expression levels were comparable between WT and *Aim2*$^{-/-}$ BMDMs under all conditions tested (New Data, Figure 3b [shown below]). These results indicate that VP22 expression in primary macrophages is not readily augmented by exogenous overexpression or viral infection and is not influenced by AIM2 deficiency. Importantly, even under these physiologically

relevant conditions, HF infection robustly activated the AIM2 inflammasome, supporting our conclusion that VP22 does not inhibit HF-induced AIM2 inflammasome activation.

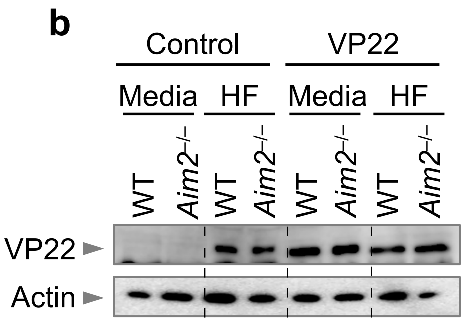

To further investigate the role of VP22 in AIM2 inflammasome regulation, we generated VP22-null mutant viruses in both the HF and F strain backgrounds using CRISPR/Cas9-mediated genome editing (New Data, Extended Data Figure 5a, 5b [shown below]).

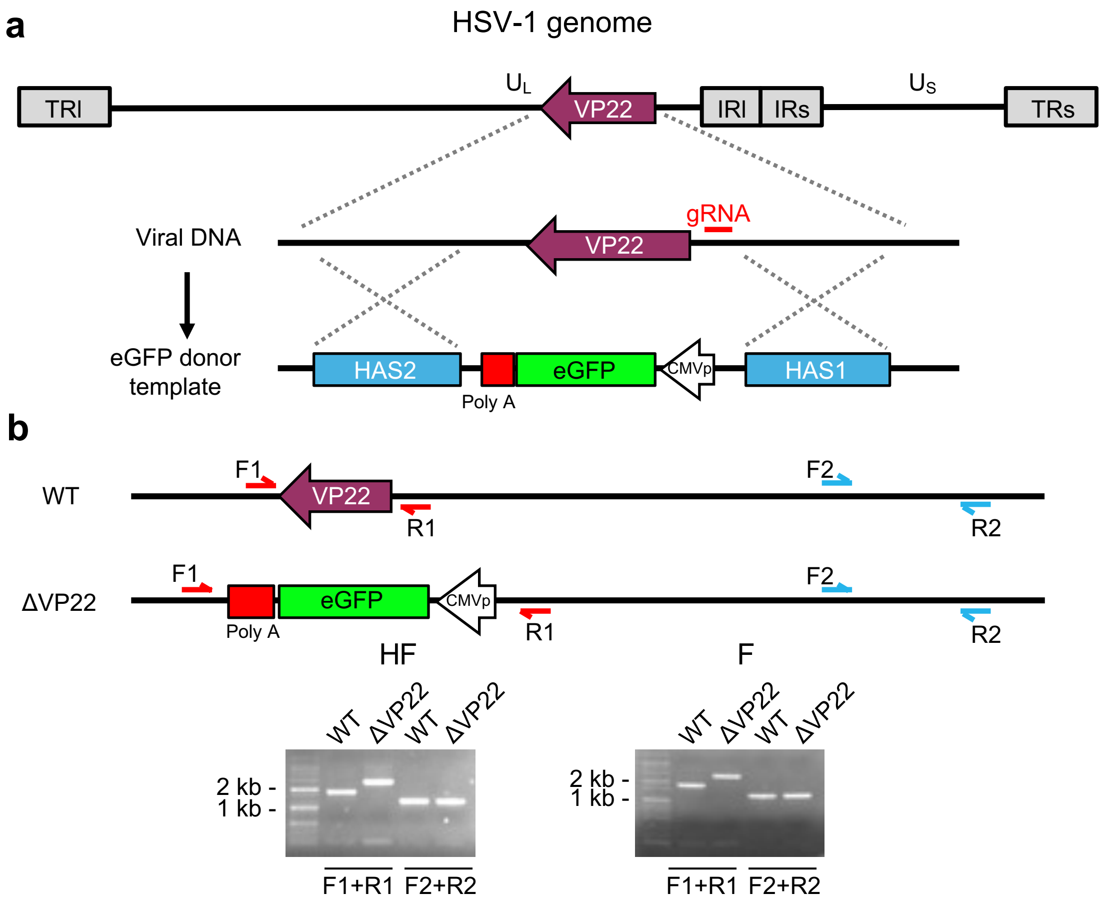

Successful deletion of VP22 was confirmed by immunoblot analysis, which showed complete loss of VP22 protein expression in the mutant viruses. Comparison of viral protein expression between WT and VP22-null viruses revealed that overall viral protein levels were slightly reduced in the VP22-null mutants compared with WT viruses, consistent with the known role of VP22 in optimal viral fitness (New Data, Extended Data Figure 5c, 5d [shown below]).

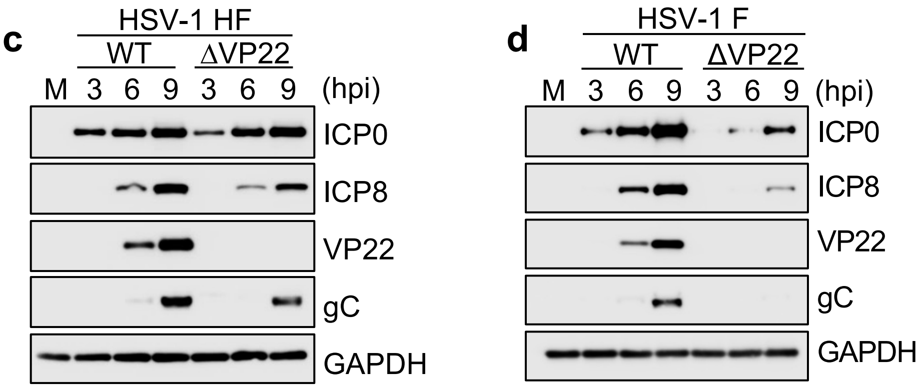

In the HF background, BMDMs infected with WT HF or VP22-null HF showed comparable levels of caspase-1 cleavage and IL-1β and IL-18 secretion (New Data, Figure 3h, 3i, 3j [shown below]). These results indicate that deletion of VP22 does not alter AIM2 inflammasome activation in the HF strain, supporting our conclusion that VP22 is not a critical regulator of inflammasome activation in this context. Accordingly, the enhanced AIM2 activation observed during HF infection is most parsimoniously explained by HF-specific genomic features, such as the long poly(T) DNA sequence.

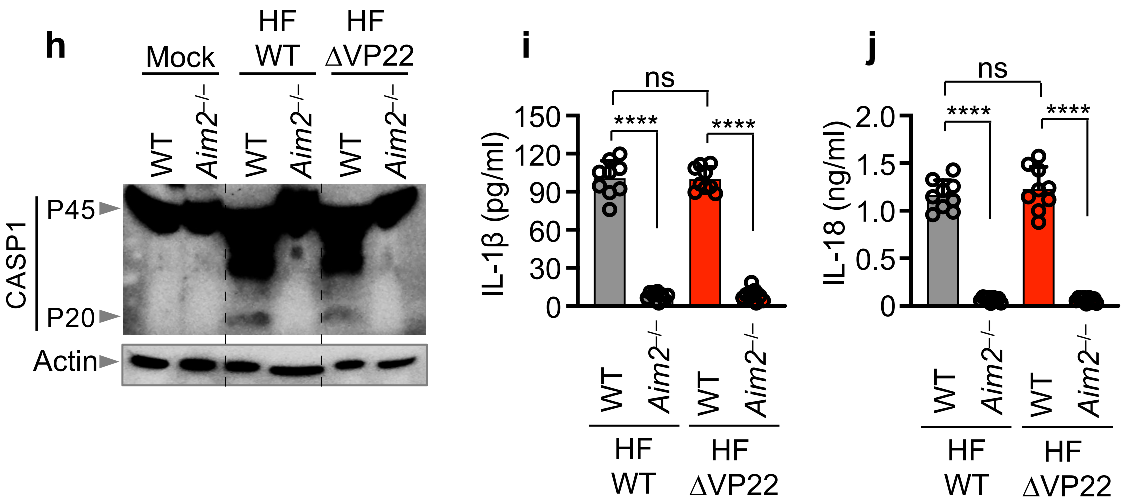

In contrast, a distinct outcome was observed in the F strain background. While WT F virus failed to induce detectable caspase-1 activation or IL-1β and IL-18 secretion, infection with the VP22-null F mutant robustly induced these inflammasome-associated readouts (New Data, Extended Data Figure 5e. 5f. 5g [shown below]). These findings demonstrate that, unlike HF, AIM2 inflammasome activation in the F strain is VP22-dependent. This result is consistent with previous reports showing that VP22 suppresses inflammasome activation in HSV-1 F and related strains (PMID: 29447697).

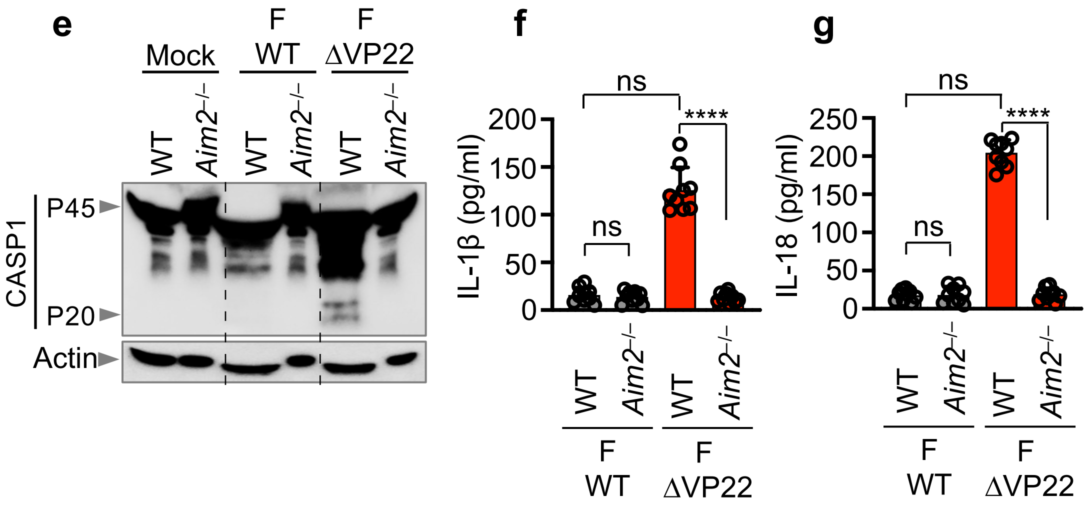

Taken together, our data reveal a strain-dependent role of VP22 in regulating AIM2 inflammasome activation. While VP22 functions as an inhibitory factor in F strain infection, it is dispensable for inflammasome regulation in the HF strain, where viral DNA features such as poly(T) DNA play a dominant role. These new data have been included in the revised manuscript in the Results section.

Comment 5: The authors should show whether the 23-mer poly(T) sequence is conserved in other laboratory strains and clinical isolates of HSV-1. Genetic modification (i.e., mutations associated with syncytial phenotype) sometimes artificially occurs during serial passages in cell cultures. How did the authors eliminate this possibility for the 23-mer poly(T) sequence in the HF strain?

Thank you for this insightful comment. To determine whether the 23-mer poly(T) sequence identified in the HF strain is the result of artificial mutation during laboratory passage or represents a naturally occurring variant, we conducted a comprehensive analysis of publicly available HSV-1 and viral genome sequences.

First, we examined 492 complete HSV-1 genome datasets from the NCBI database. Among these, 18 sequences contained a 23-mer poly(T) sequence, including several well-characterized laboratory strains (e.g., H166, KOS63, ZW63) and multiple clinical isolates (e.g., from Greninger, A.L.; Rathbun, M.; Furlong, J.; Johnston, C.). These data indicate that the 23-mer poly(T) sequence is not unique to HF or lab-adapted strains but is also

present in naturally circulating viral populations (New Data, Extended Data Figure 10a [shown below]).

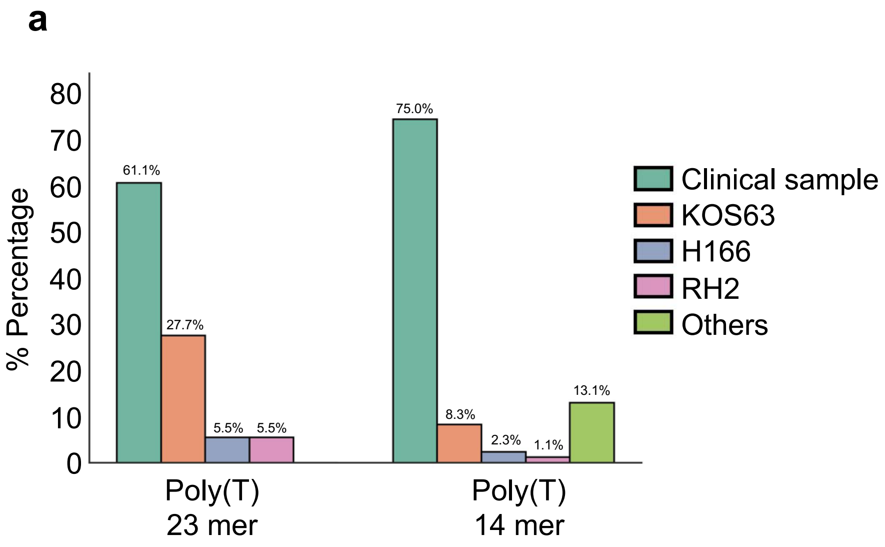

Additionally, we found that a 14-mer poly(T) sequence, which we used as a reference for the ΔT_{14} mutant, was present in approximately 15% (63/426) of clinical HSV-1 samples, while the 23-mer poly(T) sequence appeared in 3% (11/426) of those clinical datasets (New Data, Extended Data Figure 10b [shown below]). This pattern of distribution supports the interpretation that poly(T) sequence length variability is a naturally occurring polymorphism among HSV-1 strains, rather than a cell culture–induced artifact such as those seen in syncytial mutations.

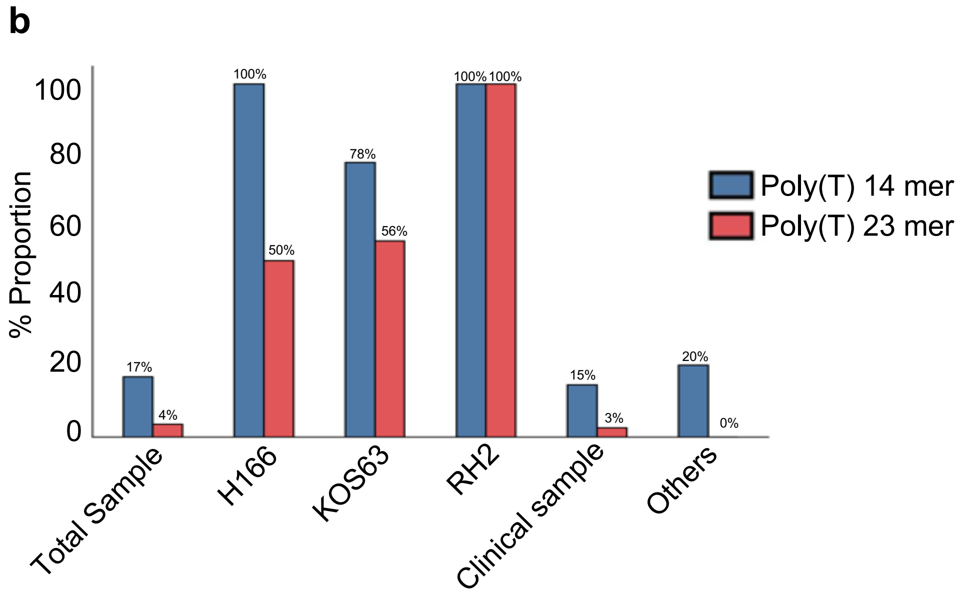

To further explore the biological relevance of these poly(T) tracts, we conducted a broader cross-species sequence analysis using the NCBI viral genome repository. Among the DNA viral genome datasets containing a 23-mer poly(T) sequence, we found that about 90% are from Monkeypox virus, Human herpesvirus and adenovirus. Among the RNA viral genome datasets containing a 23-mer poly(T) sequence, we found that about 90% are from Hepacivirus and SARS-Coronavirus (New Data, Supplementary Table 1).

For the 14-mer poly(T) motif, in addition to Monkeypox virus, we detected matches in Human herpesvirus 4 (EBV), Human papillomavirus 16 (HPV16), and Human mastadenovirus D (HAdV-D) among DNA viruses. Among RNA viruses, we identified poly(T)-containing sequences in Hepacivirus, SARS-CoV-2, and Classical Swine Fever Virus (CSFV).

These findings indicate that long poly(T) tracts are not exclusive to HSV-1, but rather are conserved across a broad spectrum of clinically important DNA and RNA viruses. This widespread occurrence suggests that poly(T) sequences may play a previously underappreciated role in innate immune sensing, and further supports the relevance of our findings in a broader virological and immunological context.

In conclusion, both intra-species HSV-1 analysis and cross-species comparisons strongly refute the idea that the 23-mer poly(T) sequence is a culture-derived artifact. Instead, the data support the view that this sequence represents a naturally occurring, functionally relevant genetic motif that may shape strain-specific host responses via AIM2 inflammasome activation.

Comment 6: Characterization of HFdelata14 is required. The authors should examine the effects of deltaT14 on viral replication, infection efficiency, the expression levels of neighboring genes (UL20 and UL21), and VP22 expression levels in BMDMs. To eliminate the off-target effect(s) of CRISPR/Cas9-mediated genome editing, the authors should construct a recombinant virus in which the mutation in HFdelta14 is repaired and test it together with wild-type and HFdelta14 viruses in the experiments shown in Fig. 6 and Extended Data Fig. 4.

Thank you for this critical and constructive comment. In response, we performed a series of additional experiments to comprehensively characterize the HFΔT_{14} mutant and to assess whether the observed phenotypes were due to the targeted deletion of the 14-bp poly(T) motif or potential off-target effects.

First, to evaluate infection efficiency and viral replication, we infected WT and *Aim2*$^{-/-}$ BMDMs with WT HF, HFΔT_{14}, and the revertant virus (HFΔT_{14}-Rev), in which the deleted 14-bp poly(T) motif was reintroduced via homologous recombination (New Data, Extended Data Figure 7a, 7b [shown below]).

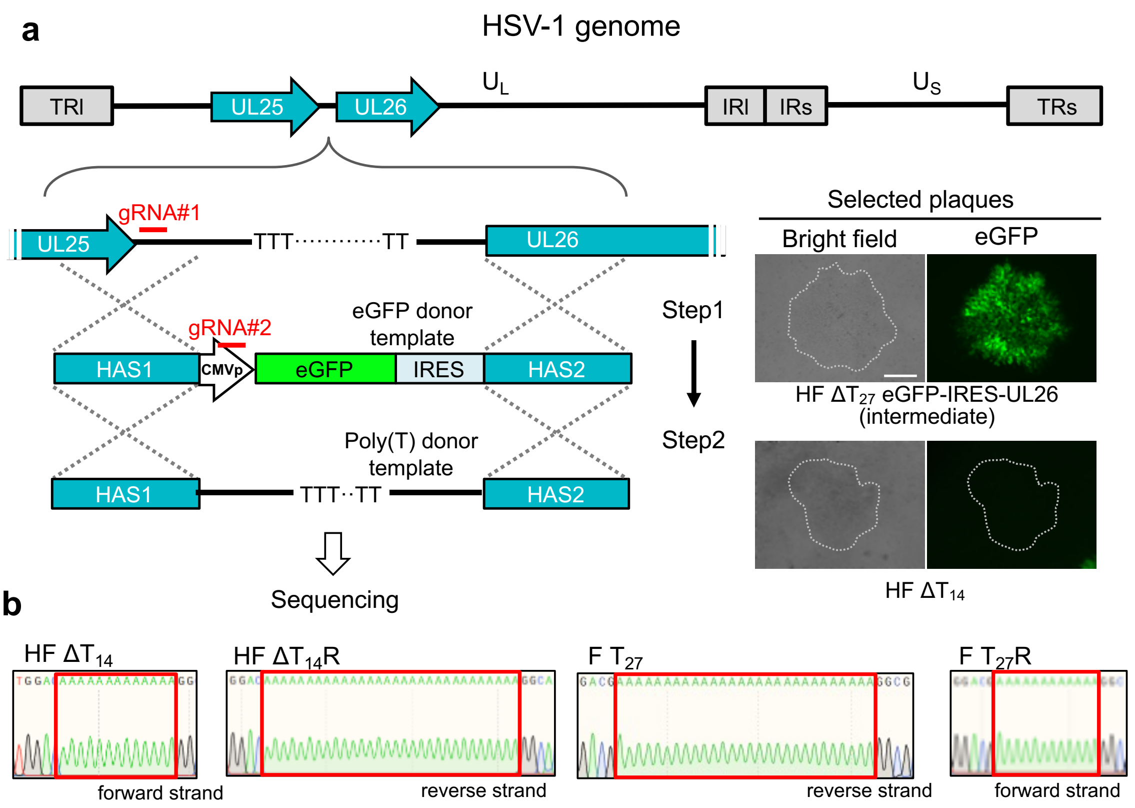

We then examined the expression of viral proteins ICP0 (immediate-early), ICP8 (early), gD, and VP22 (tegument) at 12 hours post-infection. All three viruses showed comparable protein expression levels regardless of host genotype (WT vs. *Aim2*$^{-/-}$) (New Data, Extended Data Figure 1c [shown below]), indicating that the poly(T) deletion does not impair viral gene expression or infectivity.

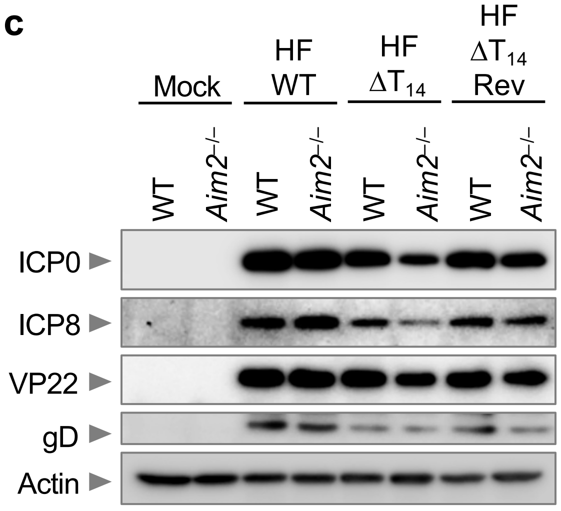

Moreover, we assessed the expression levels of the neighboring genes, UL25 and UL26, using qPCR, and found no significant differences among the three virus groups (New Data, Extended Data Figure 7e [shown below]), confirming that the deletion does not disrupt adjacent loci.

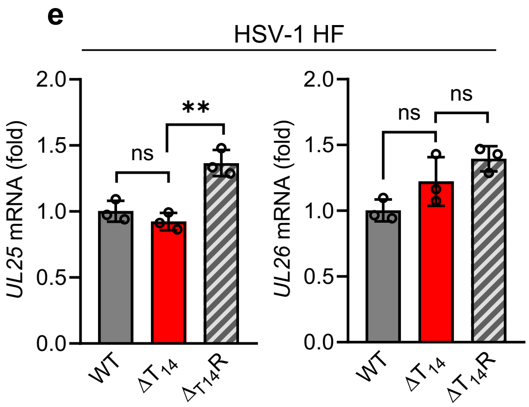

To directly address the concern regarding off-target effects of CRISPR/Cas9 editing, we generated the HF revertant virus by precisely restoring the deleted poly(T) sequence in the HFΔT_{14} genome. We then tested all three viruses, WT HF, HFΔT_{14}, and HFΔT_{14}-Rev, in the same experimental settings presented in Figure 6 and Figure 7.

In *in vitro* experiments, the results were obtained using primary BMDMs. HFΔT_{14}-Rev infection led to robust caspase-1 cleavage, IL-1β and IL-18 secretion, and pyroptotic cell death, restoring the inflammatory responses to levels comparable with those induced by WT HF (New Data, Figure 6a–e). These responses were again significantly attenuated in HFΔT_{14}-infected cells. Importantly, in *Aim2*$^{-/-}$ BMDMs, none of these inflammasome responses were detected, clearly demonstrating that inflammasome activation by the poly(T)-containing HF strain is entirely dependent on AIM2 in cellular level.

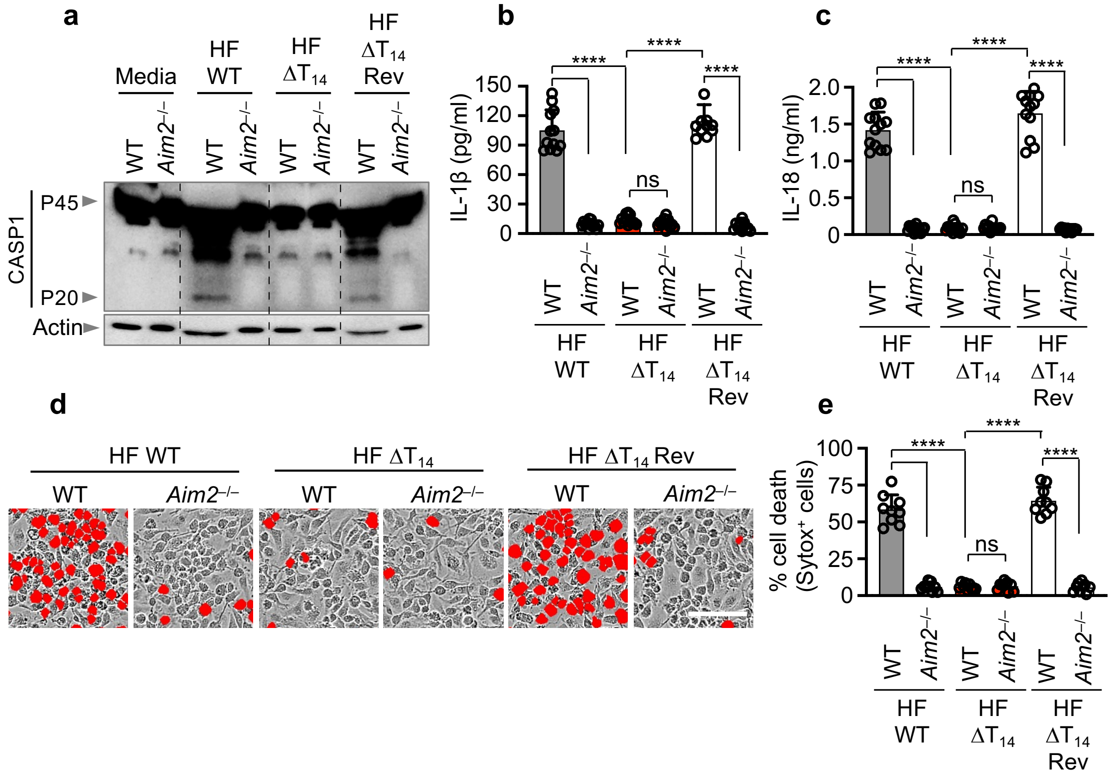

In *in vivo* experiments, the HFΔT_{14}-Rev strain fully rescued the survival phenotype, restoring host protection to levels comparable to those observed in WT HF–infected mice (New Data, Figure 7a, 7b [shown below]). Furthermore, lung viral titers, caspase-1 activation, and proinflammatory cytokine production (IL-1β and IL-18) were significantly restored in mice infected with HFΔT_{14}-Rev, closely resembling the responses observed in WT HF infection (New Data, Figure 7c–7g [shown below]). In contrast, these responses were markedly reduced in mice infected with the HFΔT_{14} mutant (New Data, Figure 7a–7g [shown below]), indicating a loss of inflammasome activation and antiviral defense. Notably, in *Aim2*$^{-/-}$ mice, all inflammasome-associated responses, including caspase-1 activation and cytokine production, were completely abolished regardless of virus strain (New Data, Figure 7a–7g [shown below]), confirming that the observed immune phenotypes are strictly AIM2-dependent *in vivo*.

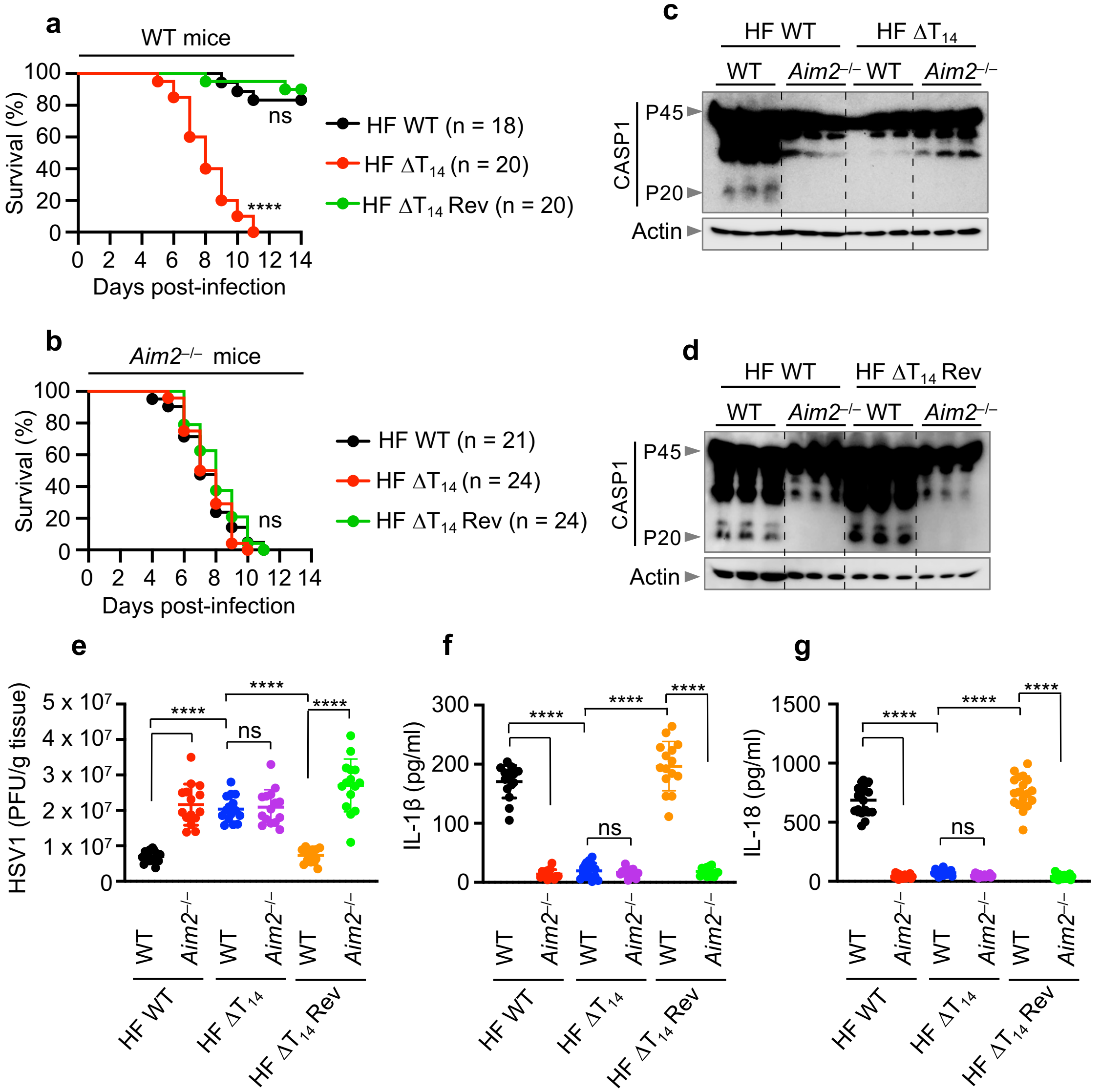

Taken together, these data confirm that the phenotypes observed in HFΔT_{14} are specifically attributable to the loss of the 14-mer poly(T) sequence, rather than off-target effects. The successful rescue of inflammasome activation and protective immunity by the revertant virus provides strong genetic evidence supporting the functional importance of the poly(T) motif in driving AIM2 inflammasome activation.

We have incorporated these new results into the revised manuscript and believe they directly and fully address your concern regarding specificity and genome integrity following genome editing.

Comment 7: To further verify the authors' conclusion that the length of the poly(T) sequence in the intergenic region of HSV-1 genome is critical for AIM2 inflammasome activation during HSV-1 infection, the authors should construct a recombinant virus derived from HSV-1 F or a KOS strain harboring the 27-mer poly(T) sequence in the intergenic region, as well as its repaired virus,

and test them together with wild-type HSV-1 F or KOS in the experiments in Fig. 6 and Extended Data Fig. 4, after characterization of the recombinant viruses, as described in Comment 6.

Thank you for this excellent suggestion, which has helped us further validate the critical role of the poly(T) sequence in AIM2 inflammasome activation. In response, we generated a recombinant HSV-1 F strain harboring a 27-mer poly(T) sequence inserted into the UL25–UL26 intergenic region (F T_{27}), as well as a corresponding revertant virus (F T_{27}-Rev) in which the poly(T) sequence was precisely removed via homologous recombination.

To assess the functional consequences of the poly(T) insertion, we performed *in vivo* infection experiments in both WT and *Aim2*$^{-/-}$ mice. We found that in WT mice, infection with the F T_{27} virus significantly improved survival compared to the parental F or F T_{27}-Rev strains, which induced rapid and severe mortality (New Data, Extended Data Fig. 8b, 8c). In contrast, in *Aim2*$^{-/-}$ mice, all three viral strains caused uniformly lethal infections, regardless of the presence or absence of the poly(T) sequence. These findings clearly demonstrate that the poly(T)-mediated protection is AIM2-dependent.

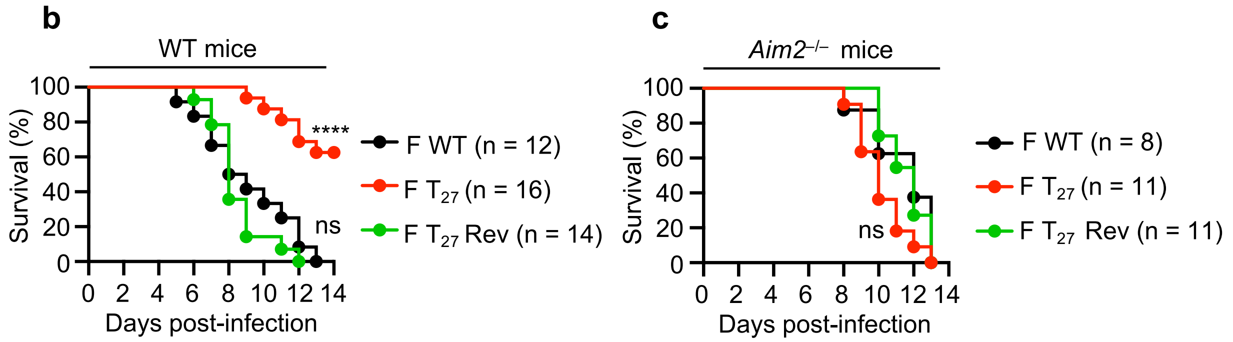

Consistently, *in vivo* caspase-1 activation was robustly induced in F T_{27}–infected WT mice but not in those infected with WT F or F T_{27}-Rev strains. Again, these responses were completely abolished in *Aim2*$^{-/-}$ mice (New Data, Extended Data Fig. 8d, 8e), further confirming that the inserted 27-mer poly(T) sequence was sufficient to activate the AIM2 inflammasome *in vivo*.

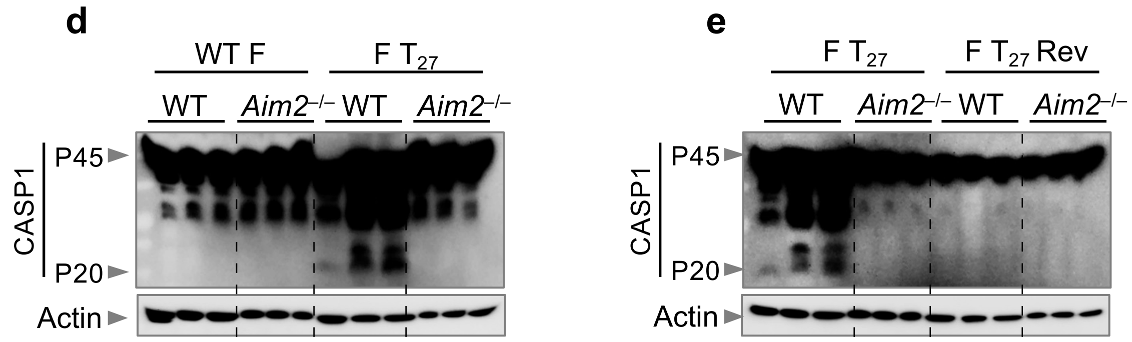

In *in vitro* studies using primary BMDMs, infection with the F T_{27} virus led to strong caspase-1 cleavage, whereas WT F and F T_{27}-Rev strains failed to activate these pathways (**New Data, Extended Data Fig. 8a**). Importantly, inflammasome activation was completely absent in *Aim2*$^{-/-}$ BMDMs, once again confirming the strict dependence on AIM2 sensing.

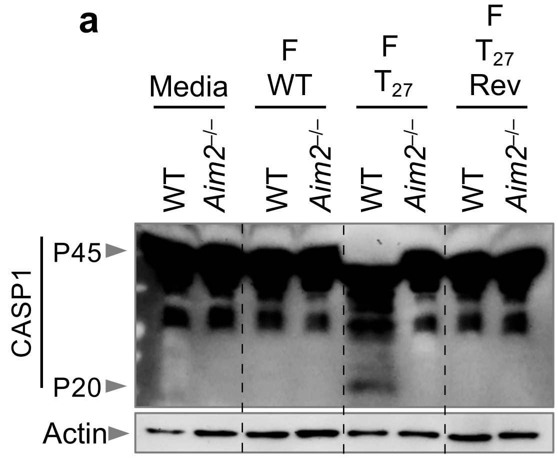

To ensure that these phenotypic differences were not due to variations in viral replication or fitness, we examined the expression levels of representative viral proteins including ICP0 (immediate-early), ICP8 (early), gD and gC (late glycoproteins), and VP22 (tegument). Immunoblot analysis revealed comparable levels of viral protein expression across WT F, F T_{27}, and F T_{27}-Rev strains, indicating that the observed immune phenotypes were not attributable to differences in viral infectivity or gene expression (**New Data, Extended Data Fig. 1d**).

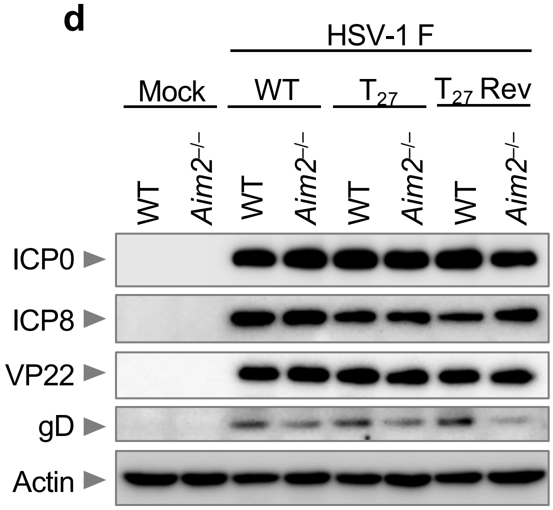

Moreover, we assessed the expression levels of the neighboring genes, UL25 and UL26, using qPCR, and found no significant differences among the three virus groups (**New Data, Extended Data Figure 7f [shown below]**), confirming that the deletion does not disrupt adjacent loci.

f

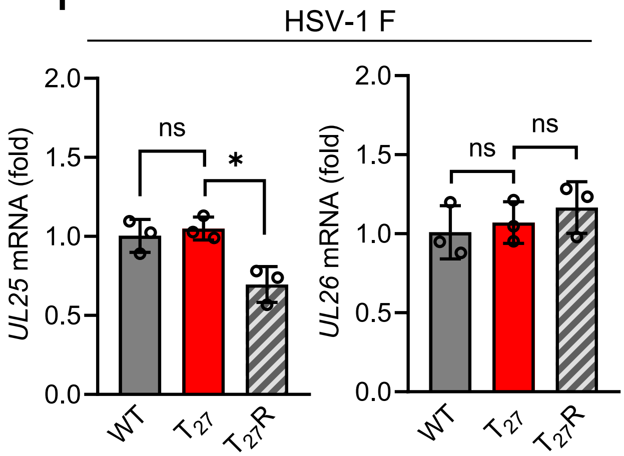

Taken together, these results provide strong genetic and functional evidence that the 27-mer poly(T) sequence is sufficient to confer AIM2 inflammasome activation, and that this response translates into enhanced antiviral defense and survival *in vivo*. These findings further reinforce the conclusion that the poly(T) sequence length in the HSV-1 genome is a key determinant of strain-specific AIM2 activation.

Comment 8: All analyses were conducted using mouse-derived cells. Since HSV-1 is a human pathogen, the authors should perform representative experiments shown in Figs. 2–5 and Extended Data Fig. 4 using human-derived cells.

Thank you for this important suggestion. Recognizing that HSV-1 is a human-specific pathogen, we extended our analysis to a human cellular system by performing representative validation experiments in THP-1–derived human macrophages, which are widely used for studying inflammasome biology.

To specifically assess the role of AIM2, we performed siRNA-mediated knockdown of AIM2 in PMA-differentiated THP-1 macrophages (New Data, Extended Data Fig. 9). We first compared WT HF and the HF VP22-null mutant in both WT and siAIM2 THP-1 cells. In WT cells, both viruses induced comparable levels of cell death, suggesting that VP22 does not significantly suppress AIM2 activation in the HF background, consistent with our findings in murine BMDMs. Importantly, this HF-induced cell death was abolished in siAIM2-treated cells, confirming the AIM2-dependence of the response in human macrophages (New Data, Extended Data Fig. 9a, 9b).

We next compared WT HF, HF ΔT_{14}, and the HFΔT_{14}-Rev viruses. In WT THP-1 macrophages, HFΔT_{14}-Rev restored the cell death response to levels similar to WT HF, while the ΔT_{14} mutant failed to induce significant cell death. This pattern was again reversed by siAIM2 knockdown, indicating that the poly(T)-dependent activation of AIM2 is conserved in human cells (New Data, Extended Data Fig. 9c, 9d).

Finally, we tested WT F, F T_{27}, and the F T_{27}-Rev viruses. As expected, the F-T_{27} virus induced strong AIM2-dependent cell death in WT THP-1 cells, whereas both WT F and F-T_{27}-Rev failed to do so. In AIM2-silenced cells, all strains failed to trigger cell death, confirming that the poly(T)-mediated activation of inflammasome signaling is specifically dependent on AIM2 (New Data, Extended Data Fig. 9e, 9f).

Across all tested viral conditions, including VP22 deletion, poly(T) insertion/deletion, and strain background, we observed that the inflammasome activation phenotypes observed in murine BMDMs were faithfully recapitulated in human macrophages, and that AIM2 was essential for these responses in both systems.

These findings provide strong evidence that our conclusions regarding poly(T)-mediated AIM2 activation and the non-essential role of VP22 in the HF background are applicable to human innate immune contexts. We have incorporated these new data into the revised manuscript (New Data, Extended Data Fig. 9) and extended the Discussion to emphasize the translational relevance of our findings in the human setting.

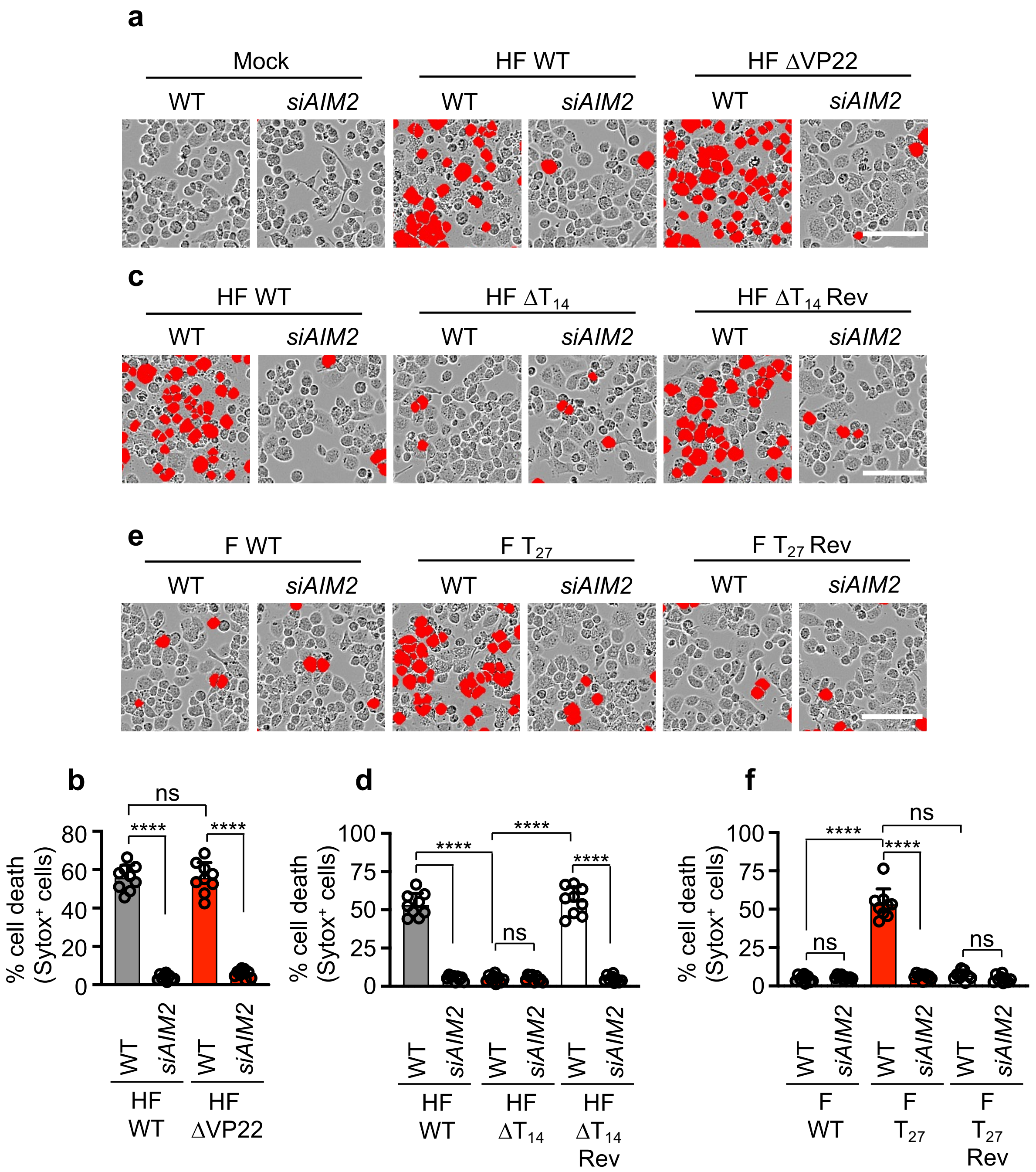

Minor Comments

Comment 1: The ASC-citrine specks in Fig. 1a and Fig. 2a are unclear. Enlarged or higher-resolution images should be provided to clearly delineate the ASC-citrine specks.

Thank you for pointing out the need for higher-resolution imaging to better visualize ASC-citrine specks. The original images presented in Figures 1a and 4a were acquired using the IncuCyte live-cell imaging system, which is optimized for real-time monitoring of speck

kinetics but offers limited spatial resolution for fine structural features. As a result, we agree that the visualization of ASC specks in these images was suboptimal.

In response to this concern, we performed new imaging using high-resolution confocal microscopy (Zeiss LSM 900) to provide clearer and more detailed visualization of ASC speck formation. These confocal images were acquired using z-stack projection and high-magnification settings, allowing us to resolve individual ASC specks with much greater clarity and precision.

Accordingly, we have removed the original IncuCyte-derived images from Figures 1a and 4a and replaced them entirely with confocal images. We believe this revision addresses your concern and significantly improves the quality and interpretability of the imaging data in our manuscript.

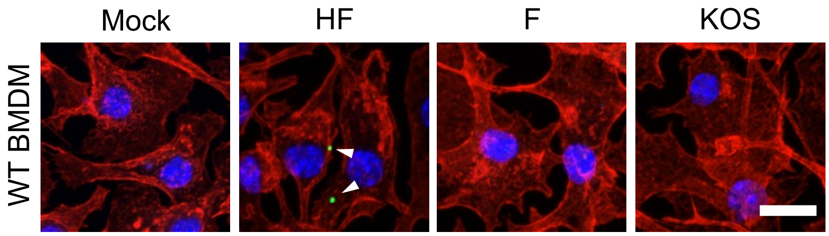

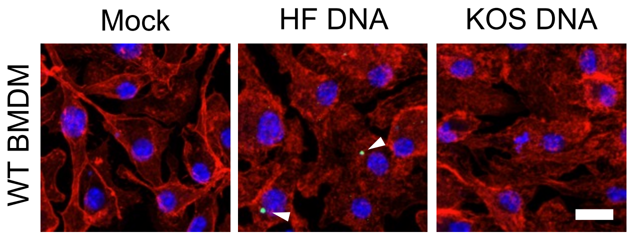

Comment 2: The authors should disclose detailed information about the VP22 expression plasmid, the gRNA sequences, and the names of the gRNA expression plasmids for the knockout iBMDMs, as well as sequence information for each gene after genome editing.

Thank you for this important and practical suggestion. In response, we have now provided comprehensive information regarding all constructs and reagents used in this study, including:

1) **VP22 expression plasmid: Detailed information on the VP22 expression construct has been added to the revised Methods section. Specifically, the F strain VP22 expression vector (pLV[Exp]-EGFP:T2A:Puro-EF1A>VP22) was purchased from VectorBuilder (VB220920-1383rfw). The construct encodes VP22 under the control of the EF1A promoter and includes an EGFP:T2A:Puro cassette for expression tracking and selection. BMDMs were transfected with the VP22 expression plasmid using DOTAP (Roche, 11202375001) 12 hours prior to HSV-1 infection, as**

described in the Methods. These details clarify the source, design, and experimental use of the VP22 expression plasmid in this study.

2) **gRNA sequences and plasmid names: For each gene knockout generated in immortalized BMDMs (iBMDMs), we have now provided the complete gRNA sequence information together with the names and catalog numbers of the corresponding gRNA expression plasmids used for CRISPR/Cas9-mediated genome editing. As described in the revised Methods section, *Sting*$^{-/-}$, *cGas*$^{-/-}$, *Myd88*$^{-/-}$, and *Trif*$^{-/-}$ iBMDMs were newly generated in this study by electroporation of WT iBMDMs with a Cas9-encoding plasmid (Addgene #43945) and individual gRNA expression plasmids (Addgene #104174) using the Amaxa 4D-Nucleofector system. All gRNA sequences and plasmid details are now explicitly listed in Source data 1 [shown below] and are referenced in the "Generation of knockout clonal cells" subsection of the Methods, thereby ensuring transparency and reproducibility of the genome-editing procedures.**

**Casp1$^{-/-}$**	
GAGGAAGCAATTTATCAACTCAGTGAGTATAGGGACAATAAATGGATTGTTGGATGAACTTTTAGAGAAGAGAGTGCTGAATCAGGAA	Reference
GAGGAAGCAATTT---TAGAGAAGAGAGTGCTGAATCAGGAA	-49 bp
**Casp3$^{-/-}$**	
GTTGACTAGCTTCTTCAGAGGCGACTACTGCCGGAGTCTGACTGGAAAGCCGAAACTCTTCATCATTCAGGTAGGGCATTGCCCAGCA	Reference
GTTGA---CCAGCA	-77 bp
**Casp6$^{-/-}$**	
TGCGAGTCAGGTTGTCTCTGTCGCGTTGGTGCCCCGCCTCTCTGGGAGGGTCAGGTGCCAAAAGAACCTCTCGTGATTGAAGATGAGG	Reference
TGCGAGTCAGGTTGTCTCTGTCGCGTTGGTGCCCCGC----CTGGGAGGGTCAGGTGCCAAAAGAACCTCTCGTGATTGAAGATGAGG	-4 bp
**Casp7$^{-/-}$**	
CGGTACAGATAAGTGGGCACTCGGTCCCGGCCGGTCCTGACGGGGCCCGCAGAGGCATTTCTCTTCTTCTTCCTTGGGTTACAGAAAG	Reference
CGGTACAGATAAGTGGGCACTCGGTCCCGGCCGGTCCT-ACGGGGCCCGCAGAGGCATTTCTCTTCTTCTTCCTTGGGTTACAGAAAG	-1 bp
**Casp9$^{-/-}$**	
CACATGAGGACTCACCATATCTGCATGTCCCCTGATCTTCCCTGGAACACCTGAGAAAACAGGAGAAAGAAGAAATGTGGGTGTTGAG	Reference
CACATGAGGACTCACCATATCTGCATGTCCCCTGATCT-CCCTGGAACACCTGAGAAAACAGGAGAAAGAAGAAATGTGGGTGTTGAG	-1 bp
**Gsdme$^{-/-}$**	
AAGTGTGAGAACCATAAGAGCGGGGCTATTGGGACAGTC-GTGGGGAAGGTCAAGCTGAACGTTGGTGGCAAAGGCGTGGTGGAGAGT	Reference
AAGTGTGAGAACCATAAGAGCGGGGCTATTGGGAC------------AGGTCAAGCTGAACGTTGGTGGCAAAGGCGTGGTGGAGAGT	-11 bp
AAGTGTGAGAACCATAAGAGCGGGGCTATTGGGACAGTCCGTGGGGAAGGTCAAGCTGAACGTTGGTGGCAAAGGCGTGGTGGAGAGT	+1 bp
**Tmlkl$^{-/-}$**	
TCTGCTGGTTAGCCTCCTTCAGGACTTCATCAAAACGGCCCAGGGCAGCAGTAATGTCATCGGGCAGGTTCTTCTTTCCTTGGGCCTG	Reference
TCTATCTGCTGGTTAGCCTCCT--------------------------------------TCATCGGGCAGGTTCTTCTTTCCTTGGG	-38 bp
TCTGCTGGTTAGCCTCCTTCAGGACTTCATCAAAACGG-CCAGGGCAGCAGTAATGTCATCGGGCAGGTTCTTCTTTCCTTGGGCCTG	-1 bp
**Ripk3$^{-/-}$**	
GAAGAACCTTTCTCAGGCCTACTGGTGCGTCAGCGGTTCCTCTGGTGAGCCGTGAAGAACTGAAGAAGCTGGAGTTTGTGGGTAAAGG	Reference
GAAGAACCTTTCTCAGGCCTACTGGTGCGTCAGCGGTT-CTCTGGTGAGCCGTGAAGAACTGAAGAAGCTGGAGTTTGTGGGTAAAGG	-1 bp
**Ripk3$^{-/-}$Caps8$^{-/-}$**	
**RIPK3**	
GAGCAAGAGAGAATGAAGAATGGGTACCTGTCATTGGATTCGGTGGGTCCAGGGATACCAAGGAGTGCCGTGTCTTCCATCTCCCTGC	Reference
GAGCAAGAGAGAATGAAGAATGGG------------------------------ATACCAAGGAGTGCCGTGTCTTCCATCTCCCTGC	-30 bp
**Casp8**	
CGGAATATAGTTCTTGTGAGATGATGAATCCACTTCTAAAGTGTGGTTCTGTTGCTCGAAGCCTGCCTCATCAGGCACTCCTTTCTGG	Reference
CGGAATATAGTTCTTGTGAGATGATGAATCCACTTCTAA-GTGTGGTTCTGTTGCTCGAAGCCTGCCTCATCAGGCACTCCTTTCTGG	-1 bp
CGGAATATAGTTCTTGTGAGATGATGAATCCACTT-----GTGTGGTTCTGTTGCTCGAAGCCTGCCTCATCAGGCACTCCTTTCTGG	-5 bp
**AIM2$^{-/-}$**	
CTTTTCAGGCTGATCCTGGGACTGTGAGATGGAGAGTGAGTACCGGGAAATGCTGTTGTTGACCGGCCTGGACCACATCACGGAGGAAGA	Reference
CTTTTCAGGCTGATCCTGGGACTGTGAGATGGAGAGTG----CCGGGAAATGCTGTTGTTGACCGGCCTGGACCACATCACGGAGGAAGA	-4 bp
CTTTTCAGGCTGATCCTGGGACTGTGAGATGGAGAGTGAG-ACCGGGAAATGCTGTTGTTGACCGGCCTGGACCACATCACGGAGGAAGA	-1 bp
**cGAS$^{-/-}$**	
GCCATCTTTCACATGTGGACCCAGGACCCGCAGGACAGTCAGTGGGACCCCAGGAACCTCAGCTCCTGCTTCGATAAGTTGTTAGCATTC	Reference
GCCATCTTTCACATGTGGACCCAGGACCCGCAGGACAA--AGTGGGACCCCAGGAACCTCAGCTCCTGCTTCGATAAGTTGTTAGCATTC	-2bp
GCCATCTTTCACATGTGGACCCAGGACCCGCAGGACAG--AGTGGGACCCCAGGAACCTCAGCTCCTGCTTCGATAAGTTGTTAGCATTC	-2bp
**IRF1$^{-/-}$**	
TAACACTCACTTTATTGATCCAGATCAGCCCTGGGATTTGGTTGGAATTAATCTGCATCTCTAGCCAGGGTCTCATCCGCATTCGAGTGA	Reference
TAACACTCACTTTATTGATCCAGATCAGCCCTGGGATT-GGTTGGAATTAATCTGCATCTCTAGCCAGGGTCTCATCCGCATTCGAGTGA	-1bp
TAACACTCACTTTATTGATCCAGATCAGCCCTGG-----GATTGGAATTAATCTGCATCTCTAGCCAGGGTCTCATCCGCATTCGAGTGA	-5bp
**STING$^{-/-}$**	
CACTCTGAAGTACCTAGCACTTCACCTAGCCTCGCACGA-ACTTGGACTACTGTTGAAAAACCTCTGCTGTCTGGCTGAAGAGCTGTGCC	Reference
CACTCTGAAGTACCTAGCACTTCACCTAGCCTCGCACGAGACTTGGACTACTGTTGAAAAACCTCTGCTGTCTGGCTGAAGAGCTGTGCC	+1bp
**MYD88$^{-/-}$**	
TGGCAATGGACCAGACACAGGTGCCCGGCAGGACGTCACGGTCGGACACACACAACTTAAGCCGATAGTCTGTCTGTTCTAGTTGCCGGA	Reference
TGGCAATGGACCAGACACAGGTGCCCGG--------------------------------GCCGATAGTCTGTCTGTTCTAGTTGCCGGA	-32
TGGCAATGGACCAGACACAGGTGCCCGGCAGGACGTCA-GGTCGGACACACACAACTTAAGCCGATAGTCTGTCTGTTCTAGTTGCCGGA	-1
**TRIF$^{-/-}$**	
GGGAGGAAGCAGAGGACAGAGGGGAAGGAGGCAGCTTAGGGGAGGCTTGGAGGGATGGTGGCTGGGCAGGAGGGCTGGATGACGTGGTGT	Reference
GGGAGGAAGCAGAGGACAGAGGGGAAGGAGGCAGC-----GGAGGCTTGGAGGGATGGTGGCTGGGCAGGAGGGCTGGATGACGTGGTGT	-5

3) **Validation of gene deletion: To confirm successful knockout of the target genes, we performed immunoblotting (Western blot) for each edited gene product, including CASP1, CASP3, CASP6, CASP7, CASP9, Gsdmd, Gsdme, Mlkl, Ripk3, Casp8, cGas, Sting, and Irf1. The absence of protein expression in the corresponding knockout cell lines verified effective gene deletion. Representative validation data are now included in New Data, Extended Data Fig. 3a, 3b, 4e [shown below].**

We believe that the inclusion of this detailed information enhances the transparency and reproducibility of our study. All related datasets and validation data have been incorporated into the revised manuscript and supplementary materials as noted above.

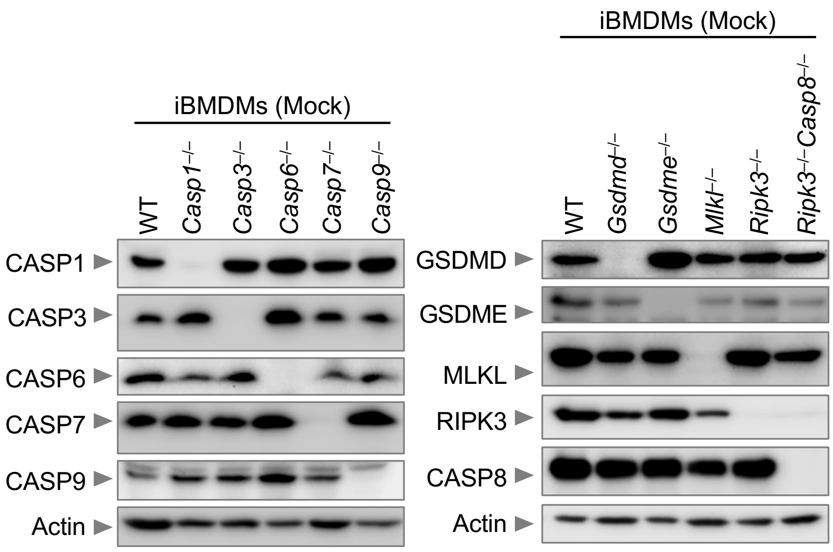

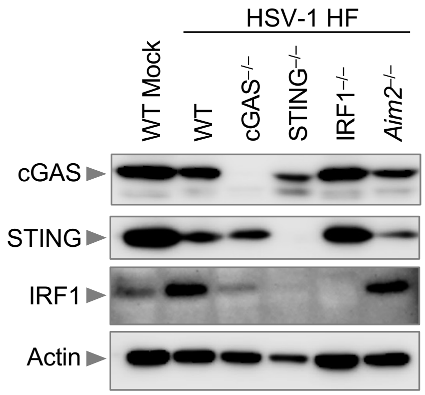

Comment 3: The infection and transfection conditions in the experiments shown in Figs. 1 to 3 should be provided in more detail. What multiplicity of infection was used? When were the samples harvested? When were the BMDMs transfected with the VP22-expression plasmid?

We appreciate your request for additional methodological clarity. In the revised manuscript, we have updated the Methods section and figure legends for Figures 1–3 to include the following detailed information:

1) **Multiplicity of infection (MOI): For all HSV-1 infection and viral DNA transfection experiments involving primary murine BMDMs, we used an MOI of 10 at 16 hours post-infection, unless otherwise noted. For THP-1–derived human macrophages, an MOI of 5 at 16 hour post infection was used to achieve comparable infection efficiency without excessive cytotoxicity.**

2) **Sample collection timing: In infection-based experiments shown in Figures 1–3, cells were harvested at 16 hours post-infection for immunoblotting, cytokine analysis, and ASC speck quantification.**

3) **VP22 expression plasmid transfection: For experiments involving VP22 overexpression (Fig. 3), BMDMs were transfected with the VP22 expression plasmid 12 hours prior to viral infection using DOTAP (Roche, 11202375001). Cells were then infected with HSV-1 and harvested 16 hours post-infection.**

These clarified parameters are now explicitly described in the updated Methods section to ensure full experimental reproducibility.

Comment 4: In Extended Data Fig. 3a, the numbers (1)–(3) should be (6)–(8)?

Thank you for identifying this labeling error. We have carefully reviewed Extended Data Fig. 3a and confirm that the numbers (1)–(3) were indeed incorrect. As you correctly noted, these should be labeled as (6)–(8) to accurately reflect the experimental conditions.

This has been corrected in the revised figure and figure legend. We appreciate your attention to detail, and this change has helped improve the clarity and accuracy of the manuscript.

Reviewer #4 (Remarks to the Author):

In the present work Oh et al study the inflammasome activation by Herpes Simplex Virus 1 strains in Mice and BMDMs and identify a poly(T) sequence of the HF strain that specifically activate Aim2. Although it is well established that dsDNA induce Aim2 activation, and that the Aim2 protein per se could have relatively broad specificity for different dsDNA, this study emphasis that in the cellular context the situation is very much more controlled and that (as far as I can see) the poly(T) sequence constitutes the first HSV1 sequence identified that can drive activation of innate immunity through Aim2. Due to this, and when our understanding of the role of Aim2 in virus immunity is still quite fragmented, I feel this work could be well suited for publication in Nature communication.

Overall, the study comprise quite comprehensive data and although the methodology used (knockdowns, IP, etc) is often open for multiple possible interpretations of what is going on in the cellular context, it appears as the data support most of their conclusions. However, I have few specific concerns that I feel should be addressed:

We sincerely thank you for taking the time to provide constructive comments and invaluable suggestions, which have significantly improved the clarity and depth of our manuscript. We are particularly grateful for your encouraging remarks that "this work could be well suited for publication in *Nature Communications*" and that "the data support most of their conclusions." These comments are highly motivating and affirm the direction of our study.

In response to your thoughtful feedback, we have further elaborated on the underlying mechanisms and conducted additional experiments to strengthen the evidence supporting our conclusions. We believe that these new data provide multiple, independent lines of support for our central findings. Below, we address each of your specific comments in detail.

- Several readout assays for Aim2 induction are used through the study, mainly Il1b/il18 release, Casp1 cleavage and cell death which in most cases appears to support a textbook view of Aim2 activation and signaling. However, the knockdown experiments shown in Extended Data Fig 2 of proteins that could be involved downstream of Aim2 activation is poorly followed up and the apparent observation that cell death is not dependent on Casp1 or GSDMD/E is intriguing and would contradict the textbook view. In a HSV1 infection (and the transfection of full virus DNA) it is quite possible that multiple pathways are activated and, although it is clear that Aim2 signaling is one of these, there might be other pathways contributing to e.g. to cell death and Il1b/Il18 release in parallel (Yes, the Aim2 KD support that Aim2 is a key player, but these are KD clones with all the possible caveats, where compensatory and complex distortions of immune signaling might have been induced due by KD of Aim2). The Casp1 or GSDMD KD data is confusing and it is also possible that these knockdown experiments have not worked well/are not informative (where are the WBs showing that these proteins are indeed knocked down?). I feel the authors should add some further follow-up experiment on at least the Casp1 knockdown to show whether

IL1b/Il18 release is attenuated in the BMDMs knockdown as expected. Overall, the mechanistic discussions are very sparse and the work would benefit from the authors further develop there point on e.g. RIPK3 dependence in the discussion and potentially also add clarifying experiments. Can it be excluded that there is a significant Toll receptor effect contributing to Il1b/Il18 release or apoptosis? Etc

Thank you for this thoughtful and insightful comment, which has prompted us to further clarify the mechanistic framework of our study. In the revised manuscript, we have addressed each of these concerns through new experimental data and an updated discussion.

First, we have confirmed the successful knockout of the following genes using immunoblotting at the protein level: Casp1, Casp3, Casp6, Casp7, Casp9, Gsdmd, Gsdme, Mlkl, Ripk3, and Casp8. All gene-edited cell lines displayed complete or near-complete loss of their respective proteins, as shown in New Data, Extended Data Fig. 3a, 3b, confirming that the observed phenotypes are not due to partial knockdown or incomplete editing.

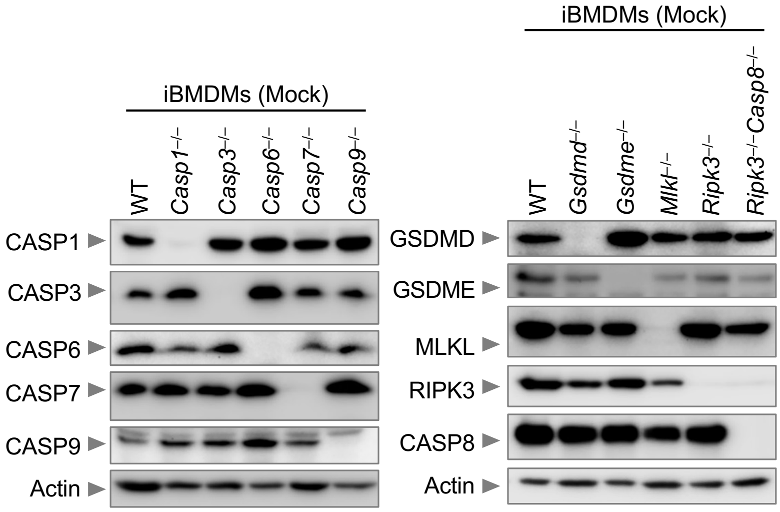

As expected, CASP1 knockout BMDMs showed a substantial reduction in IL-1β and IL-18 secretion following HSV-1 infection (New Data, Extended Data Fig. 4a, 4b), confirming the essential role of caspase-1 in cytokine maturation. Interestingly, cell death was still observed in CASP1-deficient cells, suggesting that additional caspase-1–independent cell death pathways are also engaged during HSV-1 infection.

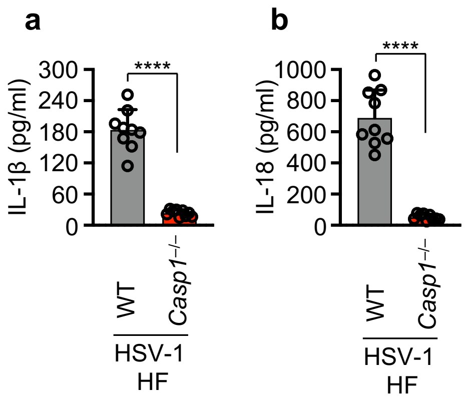

To better understand these non-canonical mechanisms, we referred to our previous study (Lee et al., Nature 2021 [PMID: 34471287]), in which we demonstrated that AIM2 can coordinate with other innate immune sensors such as Pyrin and ZBP1 to form a multiprotein complex known as the PANoptosome. This platform mediates PANoptosis, a form of inflammatory cell death that integrates pyroptosis, apoptosis, and necroptosis. In that work, we showed that AIM2 drives the assembly of a complex containing ASC, caspase-1, RIPK3, caspase-8, and FADD, leading to inflammatory cell death during viral infection.

Building on this concept, our previous study demonstrated that RIPK3 plays a critical role in the assembly and function of the AIM2-driven PANoptosome. Specifically, in Ripk3 knockout BMDMs infected with HSV-1, we observed a marked reduction in ASC–caspase-8 colocalization and impaired cell death, indicating that RIPK3 is essential for full execution of the PANoptotic cell death program.

To address the possibility that Toll-like receptor (TLR) signaling contributes to cell death, we infected MYD88 KO and TRIF KO iBMDMs with HSV-1 HF. No significant changes in cell death were observed compared with that of WT iBMDMs, indicating that TLR pathways are not major contributors to the inflammatory cell death observed in our model (New Data, Extended Data Fig. 4c, 4d).

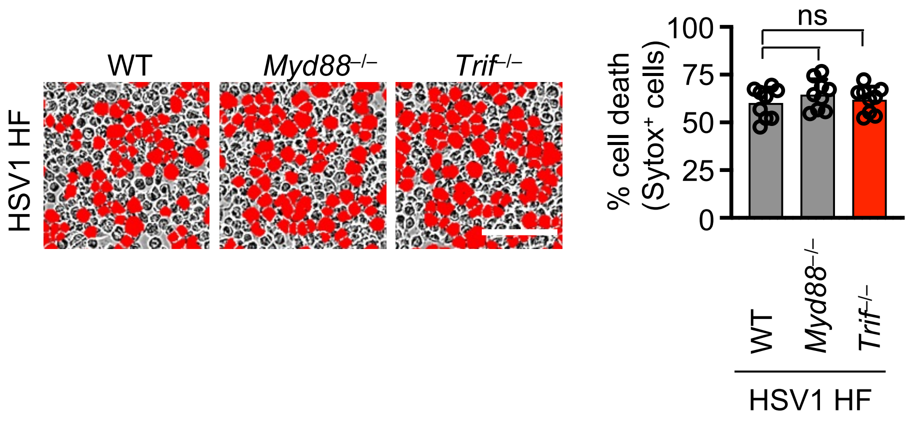

Together, these findings support a model in which AIM2 recognizes viral DNA and initiates PANoptosis via a multi-sensor inflammasome complex involving RIPK3 and caspase-8, in addition to caspase-1, and that TLR signaling does not play a dominant role in this process. We have revised the Results section accordingly to incorporate this expanded mechanistic understanding.

- The section addressing a potential role of VP22 is an inhibitor of Aim2 is not very crisp and the experiments might eventually not be informative. The initial paragraph contains a somewhat superficial discussion of structural regions and what could be functionally significant. In the end of a paragraph, they say "Variants outside of this core domain are thus not expected to significantly impact VP22 functions and are consequently categorized as functionally neutral". Although e.g. Aints et al addresses other types of functions, I cannot really find the data excluding that other regions could have additional functions, e.g. in the context of Aim2 inhibition. Although these regions might be intrinsically disordered, they could make important interactions. This paragraph should be rewritten, e.g. to instead just describe what is known about the structure (if the authors do not know about more crisp data excluding functional significance of specific regions).

Thank you for this valuable comment and agree that the functional interpretation of VP22's structural domains warrants a more cautious and precise discussion.

To directly investigate whether VP22 suppresses AIM2 inflammasome activation in the HF strain, we generated a VP22-null mutant virus in the HF background using CRISPR/Cas9-mediated genome editing (New Data, Extended Data Figure 5a, 5b [shown below]).

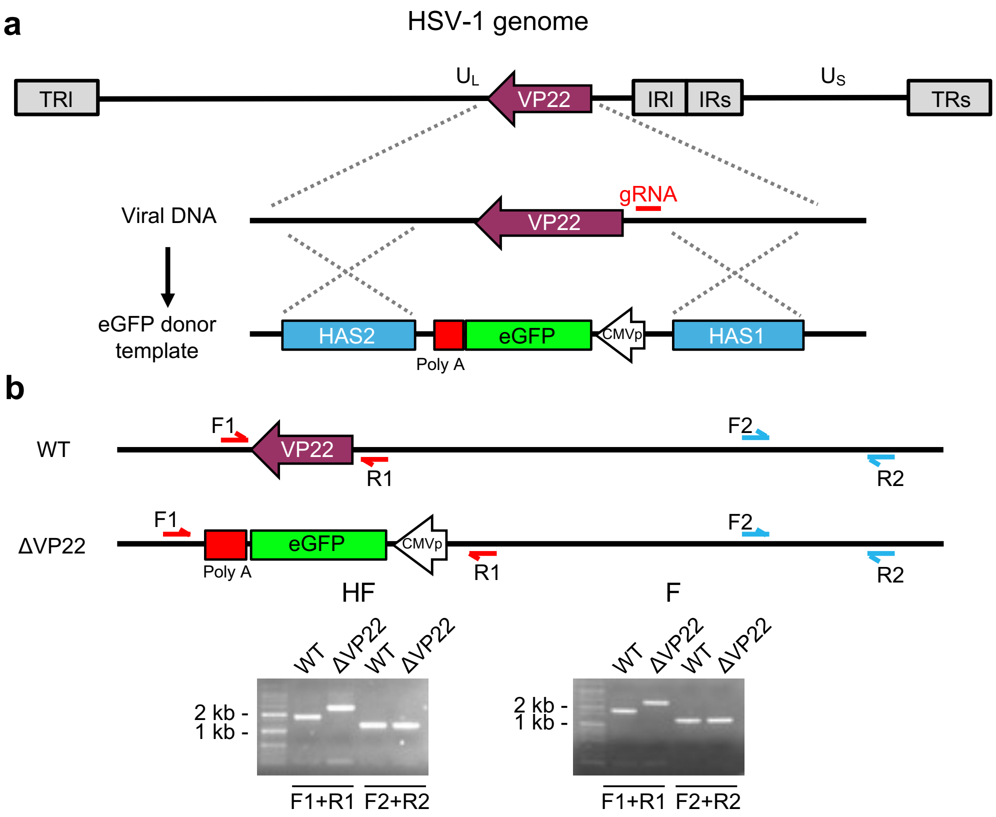

We then infected primary BMDMs with this mutant virus and assessed caspase-1 activation, and IL-1β and IL-18 secretion as key readouts of inflammasome activation. The results showed that VP22 deletion in the HF strain did not alter inflammasome activation, with responses comparable to those induced by WT HF virus (**New Data, Fig. 3h–3j**). These findings suggest that, in the HF background, VP22 is not required for the suppression or activation of the AIM2 inflammasome.

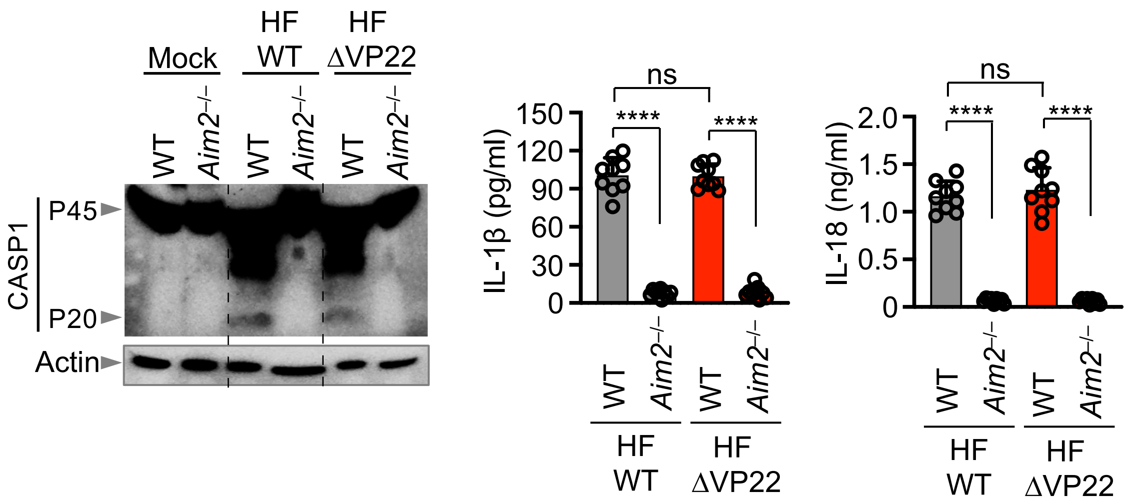

In contrast, a distinct outcome was observed in the F strain background. While WT F virus failed to induce detectable caspase-1 activation or IL-1β and IL-18 secretion, infection with the VP22-null F mutant robustly induced these inflammasome-associated readouts (New Data, Extended Data Figure 5e, 5f, 5g [shown below]). These findings demonstrate that, unlike HF, AIM2 inflammasome activation in the F strain is VP22-dependent. This result is consistent with previous reports showing that VP22 suppresses inflammasome activation in HSV-1 F and related strains (PMID: 29447697).

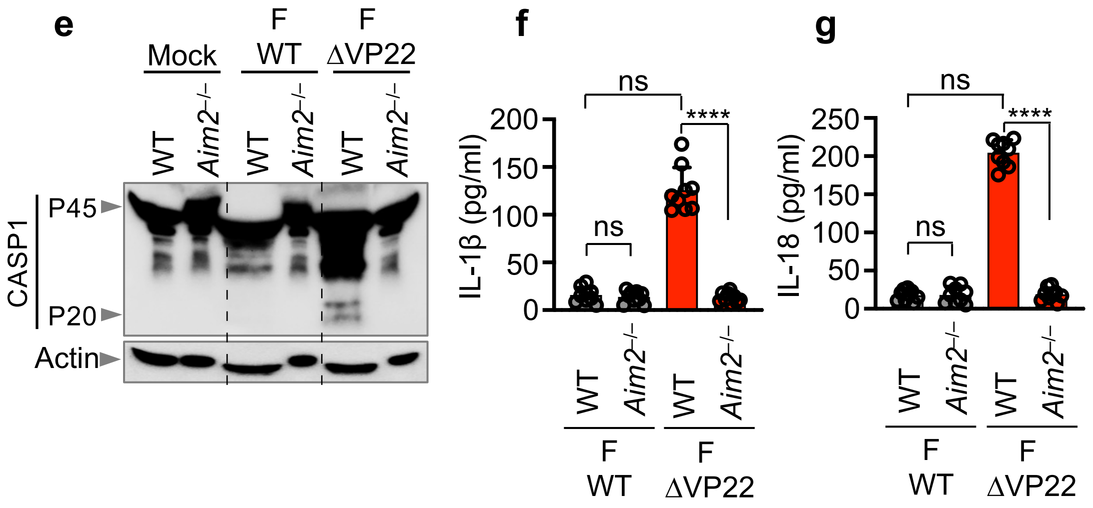

In light of the reviewer's suggestion, we have revised the paragraph discussing VP22 to make it more concise and evidence based. Rather than inferring functional neutrality of sequence variations outside the conserved core domain, we now limit the description to established structural features of VP22, including the presence of a conserved core region defined by prior structural and comparative studies.

We also acknowledge that regions outside the structured core are less well-characterized, and therefore have refrained from making definitive statements regarding their functional relevance, particularly in the context of AIM2 inflammasome regulation. This revision avoids overinterpretation and more accurately reflects the current understanding and limitations of VP22 structure–function relationships.

We appreciate your constructive critique, which has helped us refine both the experimental and conceptual clarity of this section.

- It is concluded from the VP22 over-expression experiment that there is no differential effect of VP22 for the different strains. However, there is no positive control experiment showing that the VP22 overexpression in this system can inhibit Aim2. Therefore, I would like to see a control experiment demonstrating that VP22 overexpression can indeed inhibit Aim2 activation, e.g. with a highly potent dsDNA for Aim2 activation. Otherwise, there is no use addressing differential effects.

Thank you for this important and constructive suggestion. To determine whether VP22 can inhibit AIM2 inflammasome activation, we performed a set of control experiments using potent synthetic AIM2 ligands, including poly(dA:dT) and poly(dG:dC), as well as dsDNA poly(T) 90-mer, which was shown in this study to robustly activate AIM2 **(New Data, Extended Data Figure 6e-6h [shown below])**. In BMDMs transfected with any of these dsDNA ligands, we consistently observed strong caspase-1 activation and IL-1β secretion, confirming effective AIM2 inflammasome engagement.

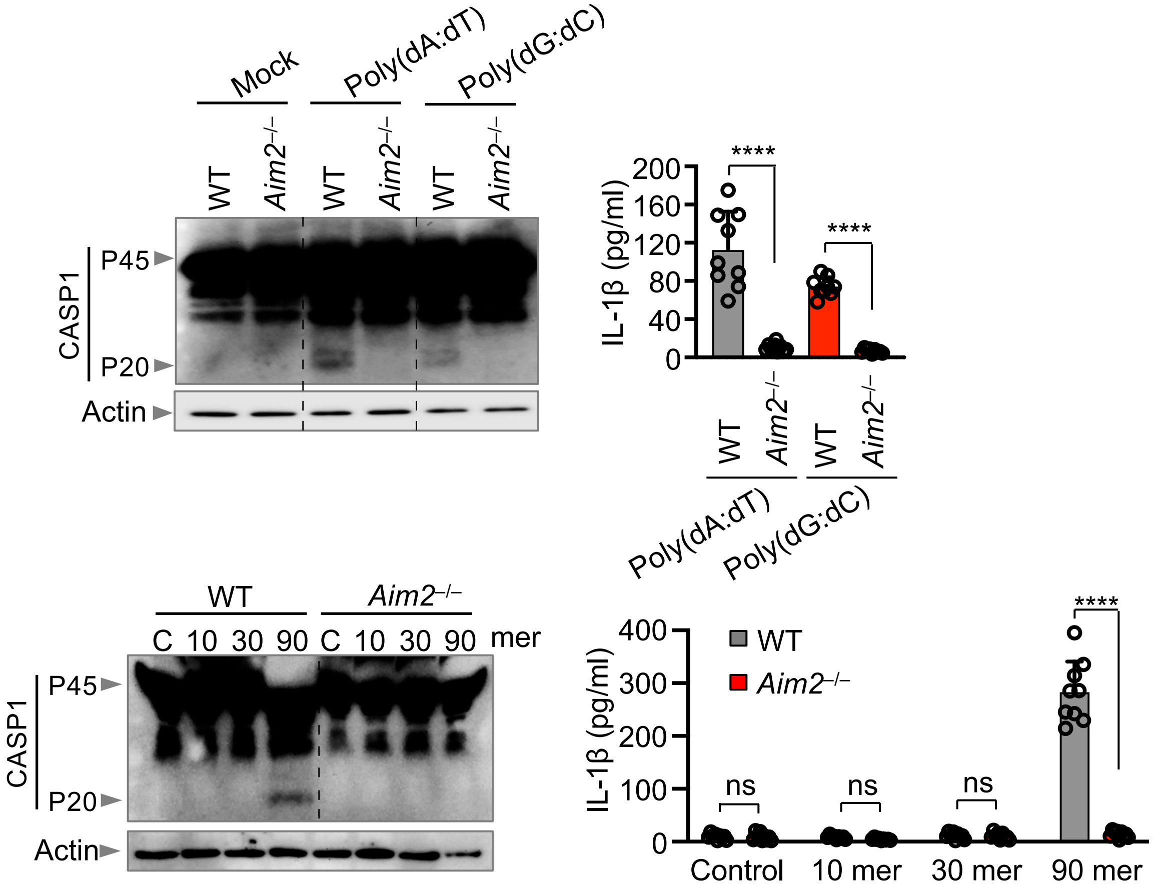

We next performed immunoblotting to confirm VP22 expression following transfection of the VP22 expression vector. In mock-infected BMDMs, VP22 was expressed at detectable levels upon transfection, confirming successful overexpression. Moreover, subsequent infection with HSV-1 HF did not further enhance VP22 expression. Importantly, VP22 expression levels were comparable between WT and *Aim2*$^{-/-}$ BMDMs under all conditions tested (**New Data, Figure 3b [shown below]**). These results indicate that VP22 expression in primary macrophages is not readily augmented by exogenous overexpression or viral infection and is not influenced by AIM2 deficiency. Importantly, even under these physiologically relevant conditions, HF infection robustly activated the AIM2

inflammasome, supporting our conclusion that VP22 does not inhibit HF-induced AIM2 inflammasome activation.

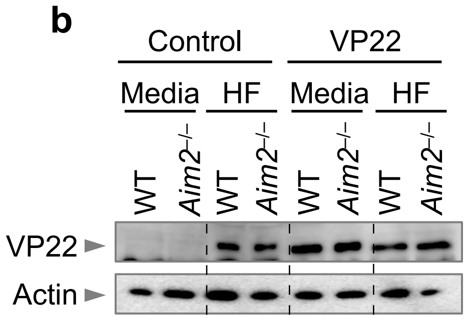

To further investigate the role of VP22 in AIM2 inflammasome regulation, we generated VP22-null mutant viruses in both the HF and F strain backgrounds using CRISPR/Cas9-mediated genome editing. Successful deletion of VP22 was confirmed by immunoblot analysis, which showed complete loss of VP22 protein expression in the mutant viruses. Comparison of viral protein expression between WT and VP22-null viruses revealed that overall viral protein levels were slightly reduced in the VP22-null mutants compared with WT viruses, consistent with the known role of VP22 in optimal viral fitness (New Data, Extended Data Figure 5c, 5d [shown below]).

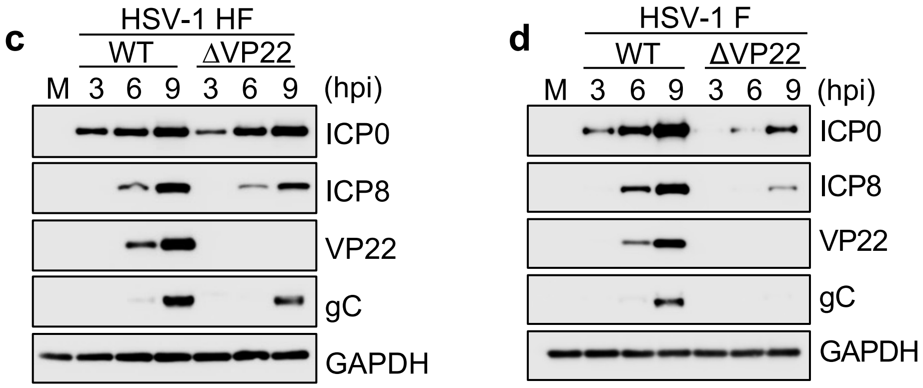

In the HF background, BMDMs infected with WT HF or VP22-null HF showed comparable levels of caspase-1 cleavage and IL-1β and IL-18 secretion (New Data, Figure 3h, 3i, 3j [shown below]). These results indicate that deletion of VP22 does not alter AIM2 inflammasome activation in the HF strain, supporting our conclusion that VP22 is not a critical regulator of inflammasome activation in this context. Accordingly, the enhanced AIM2 activation observed during HF infection is most parsimoniously explained by HF-specific genomic features, such as the long poly(T) DNA sequence.

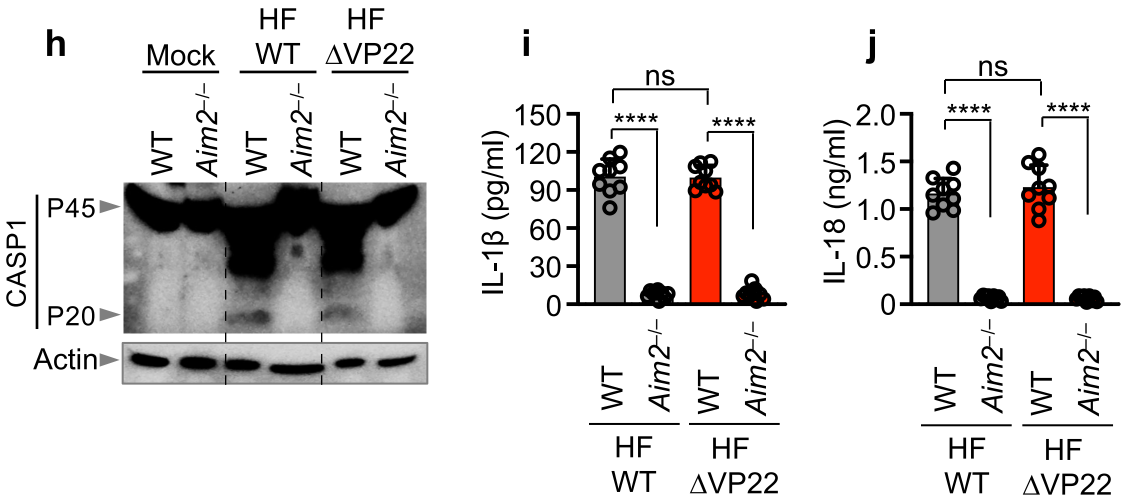

In contrast, a distinct outcome was observed in the F strain background. While WT F virus failed to induce detectable caspase-1 activation or IL-1β and IL-18 secretion, infection with the VP22-null F mutant robustly induced these inflammasome-associated readouts **(New Data, Extended Data Figure 5e. 5f. 5g [shown below])**. These findings demonstrate that, unlike HF, AIM2 inflammasome activation in the F strain is VP22-dependent. This result is consistent with previous reports showing that VP22 suppresses inflammasome activation in HSV-1 F and related strains (PMID: 29447697).

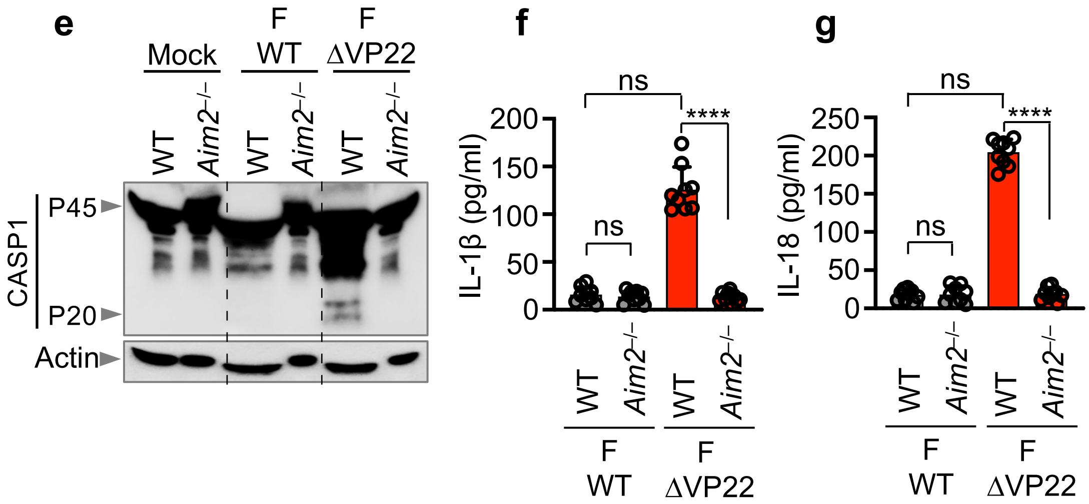

Taken together, our data reveal a strain-dependent role of VP22 in regulating AIM2 inflammasome activation. While VP22 functions as an inhibitory factor in F strain infection, it is dispensable for inflammasome regulation in the HF strain, where viral DNA features such as poly(T) DNA play a dominant role. These new data have been included in the revised manuscript in the Results section.

- In the IP figures (e.g. Fig2 F), in the Input chart, there is no Aim2 protein present for the WT media lane. This suggests that the virus first induce Aim2, which is subsequently activated by viral dsDNA (?). How is Aim2 induced in this context? It would be particiularly interesting to shed light on how this is accomplished for HF seq#5 when this is a requirement for Aim2 activation.

Thank you for raising this important question regarding the regulation of AIM2 expression in response to viral infection.

Indeed, as you observed, AIM2 protein was not detectable in uninfected BMDMs (WT media lane). This observation is consistent with previous studies (e.g., PMID: 34471287, 38008850, 41225161), which have shown that AIM2 is not constitutively expressed at high levels under resting conditions, and is instead inducible upon innate immune stimulation.

The upstream regulatory mechanisms controlling AIM2 expression have been well described in the literature. Notably, prior studies (e.g., PMID: 25774715, 37557956) have demonstrated that AIM2 transcription is directly regulated by IRF1, a transcription factor activated downstream of various cytosolic DNA sensing pathways, including cGAS–STING signaling.

To investigate whether this pathway contributes to AIM2 induction in our system, we generated cGAS, STING, and IRF1 knockout (KO) iBMDMs and examined cell death responses in IRF1 KO, cGAS KO, and STING KO iBMDMs following HSV-1 HF infection. Interestingly, all three KO lines exhibited marked suppression of virus-induced cell death, comparable to the phenotype observed in AIM2 KO cells (New Data, Extended Data Figure 4f, 4g [shown below]). These findings support a model in which the cGAS–STING–IRF1 axis mediates the transcriptional induction of AIM2, enabling subsequent inflammasome activation by viral DNA.

We have included these mechanistic insights and supporting experimental data in the revised manuscript to clarify how AIM2 inflammasome is regulated in the context of HSV-1 HF infection.

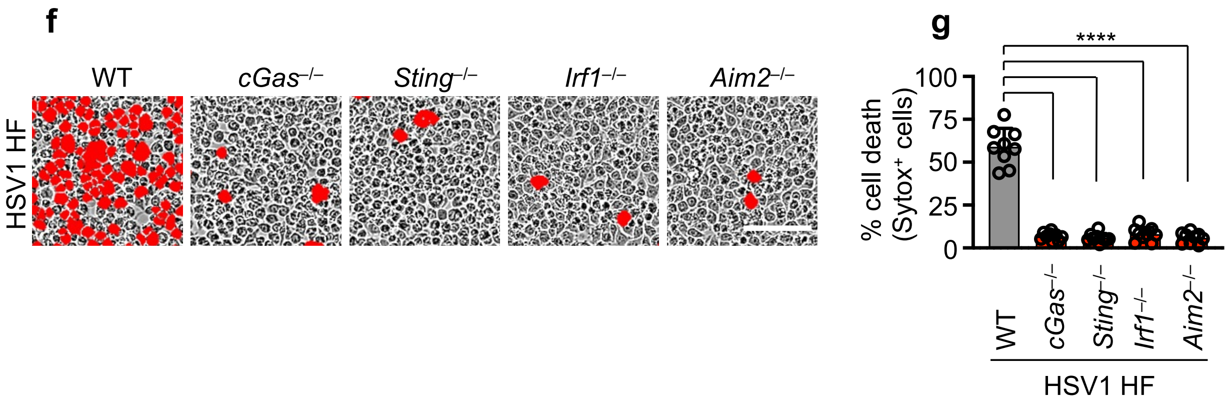

- I feel the Discussion section is quite thin, and in addition to above points on the lack of discussion of mechanistic implications, it should be possible to further address the implications of the poly(T) sequence for virus immune response. Are there other Herpesviruses, other viruses, containing similar sequences. This sequence is relative short in relationship to some other Aim2 inducing sequences, etc

Thank you for this thoughtful suggestion, which has prompted us to more thoroughly explore the immunological significance of the poly(T) sequence and its evolutionary context across viruses.

First, we examined 492 complete HSV-1 genome datasets from the NCBI database. Among these, 18 sequences contained a 23-mer poly(T) sequence, including several well-characterized laboratory strains (e.g., H166, KOS63, ZW63) and multiple clinical isolates (e.g., from Greninger, A.L.; Rathbun, M.; Furlong, J.; Johnston, C.). These data indicate that the 23-mer poly(T) sequence is not unique to HF or lab-adapted strains but is also present in naturally circulating viral populations (New Data, Extended Data Figure 10a [shown below]).

a

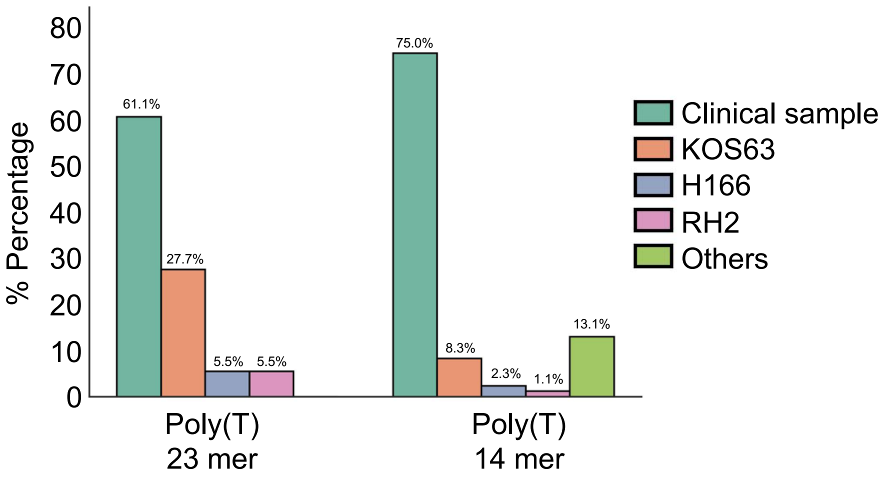

Additionally, we found that a 14-mer poly(T) sequence, which we used as a reference for the ΔT_{14} mutant, was present in approximately 15% (63/426) of clinical HSV-1 samples, while the 23-mer poly(T) sequence appeared in 3% (11/426) of those clinical datasets (New Data, Extended Data Figure 10b [shown below]). This pattern of distribution supports the interpretation that poly(T) sequence length variability is a naturally occurring polymorphism among HSV-1 strains, rather than a cell culture–induced artifact such as those seen in syncytial mutations.

b

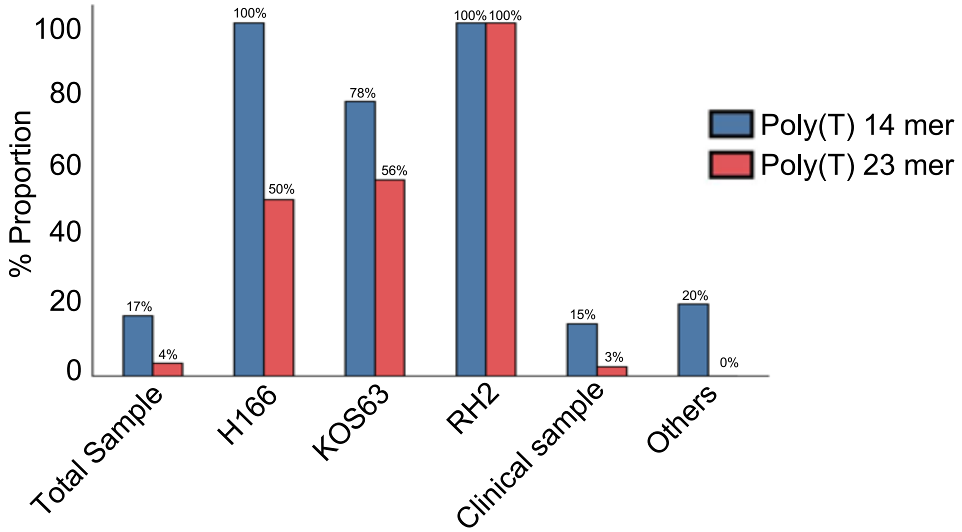

To further explore the biological relevance of these poly(T) tracts, we conducted a broader cross-species sequence analysis using the NCBI viral genome repository. Among the DNA viral genome datasets containing a 23-mer poly(T) sequence, we found that about 90% are from Monkeypox virus, Human herpesvirus and adenovirus. Among the RNA viral genome datasets containing a 23-mer poly(T) sequence, we found that about 90% are from Hepacivirus and SARS-Coronavirus (New Data, Supplementary Table 1).

For the 14-mer poly(T) motif, in addition to Monkeypox virus, we detected matches in Human herpesvirus 4 (EBV), Human papillomavirus 16 (HPV16), and Human mastadenovirus D (HAdV-D) among DNA viruses. Among RNA viruses, we identified poly(T)-containing sequences in Hepacivirus, SARS-CoV-2, and Classical Swine Fever Virus (CSFV).

These findings indicate that long poly(T) tracts are not exclusive to HSV-1, but rather are conserved across a broad spectrum of clinically important DNA and RNA viruses. This widespread occurrence suggests that poly(T) sequences may play a previously underappreciated role in innate immune sensing, and further supports the relevance of our findings in a broader virological and immunological context.

In conclusion, both intra-species HSV-1 analysis and cross-species comparisons strongly refute the idea that the 23-mer poly(T) sequence is a culture-derived artifact. Instead, the data support the view that this sequence represents a naturally occurring, functionally relevant genetic motif that may shape strain-specific host responses via AIM2 inflammasome activation.

According to the reviewer's comment against the thin Discussion section, we added the following paragraph at the end of the Discussion:

Future studies will be required to determine how poly(T) length polymorphisms influence disease severity in patients and whether similar DNA motifs contribute to inflammasome activation in other viral or inflammatory disorders. Systematic profiling of poly(T)-rich genomic regions across viral genomes, coupled with clinical outcome data, may further elucidate how viral genomic variation shapes innate immune responses in vivo.

Collectively, our study establishes viral poly(T) DNA as a physiologically relevant ligand that governs strain-specific AIM2 inflammasome activation during HSV-1 infection. By uncovering how naturally occurring genomic sequence variation shapes innate immune sensing, these findings advance fundamental understanding of host-virus interactions and highlight poly(T) DNA as a potential target for therapeutic modulation of inflammasome responses. More broadly, this work provides a conceptual framework for exploring how subtle viral genome polymorphisms can decisively influence immune outcomes, extending beyond HSV-1 to other viral and inflammatory diseases.

Reviewer #1 (Remarks to the Author):

Reviewer #1 (Remarks to the Author):

The revised manuscript presents compelling genetic and functional evidence that viral poly(T) DNA length polymorphisms determine strain-specific AIM2 inflammasome activation during HSV-1 infection. The inclusion of precise deletion, reversion, and insertion mutants, together with in vivo validation and confirmation in human macrophages, substantially strengthens the mechanistic framework and causal inference.
The study is carefully designed, technically rigorous, and conceptually significant. I have only minor suggestions aimed at further improving clarity and transparency:

We sincerely thank the reviewer for the positive and thoughtful evaluation of our work. We greatly appreciate the reviewer's recognition of the conceptual significance of our study and the rigorous experimental design supporting our conclusions. We are also grateful for the helpful suggestions aimed at improving the clarity and transparency of the manuscript. We have carefully considered these comments and revised the manuscript accordingly.

Clarification of DNA length context
The manuscript demonstrates that efficient AIM2 activation requires dsDNA above a length threshold (~≥80 bp), while the HF strain contains a 27-mer poly(T) tract embedded within genomic DNA. A brief clarification in the Discussion explaining how the genomic context provides sufficient DNA length for AIM2 oligomerization would further strengthen mechanistic clarity.

We thank the reviewer for this insightful suggestion. We agree that clarifying the relationship between the poly(T) motif and the DNA length requirement for AIM2 activation improves the mechanistic clarity of our study. Although the poly(T) tract itself is 27 nucleotides in length, it is embedded within the much longer HSV-1 genomic DNA, which provides an extended dsDNA platform capable of supporting AIM2 filament formation and inflammasome assembly. Thus, the poly(T) motif likely functions as a sequence determinant within a longer genomic DNA context rather than as an isolated ligand. We have now added a brief clarification in the Discussion to explain this point.

Statistical reporting consistency
Please ensure that exact sample sizes (n), number of biological replicates, and statistical tests are consistently reported in all figure legends, including Extended Data figures.

We thank the reviewer for this helpful suggestion. We have carefully reviewed all figure legends and ensured that the exact sample sizes (n), number of biological replicates, and statistical tests are consistently reported throughout the manuscript. This information has now been clearly indicated in all figure legends, including those of the Extended Data figures.

Immunoblot presentation
Where feasible, include molecular weight markers or indicate expected molecular weights in figure legends for key immunoblots to enhance transparency.

These are presentation-level refinements and do not affect the central conclusions.

Overall, this is a strong and well-supported study that provides important insights into viral genome–encoded determinants of innate immune sensing.

We sincerely thank the reviewer for the positive and encouraging evaluation of our study. We greatly appreciate the reviewer's recognition of the significance of our findings and the strength of the experimental evidence supporting our conclusions.

In response to this suggestion, we have revised the figure legends to indicate the expected molecular weights for key immunoblot bands where appropriate. These additions improve the transparency and clarity of data presentation.

We thank the reviewer for these helpful suggestions, which have further improved the presentation of the manuscript.

Reviewer #2 (Remarks to the Author):

I co-reviewed this manuscript with one of the reviewers who provided the listed reports. This is part of the Nature Communications initiative to facilitate training in peer review and to provide appropriate recognition for Early Career Researchers who co-review manuscripts.

We appreciate your participation in the co-review process and the *Nature Communications* initiative to support Early Career Researchers. No further comments were provided in this section, so we thank you for your support.

Reviewer #3 (Remarks to the Author):

The authors have performed an extensive and impressive set of additional experiments, and the revised manuscript is substantially strengthened compared to the original submission.

I am largely satisfied with the authors' responses and the revised manuscript, and I support its publication in Nature Communications after minor revision. I have two suggestions that would further improve the clarity and presentation of the work.

We sincerely thank the reviewer for the positive and encouraging evaluation of our revised manuscript. We greatly appreciate the reviewer's recognition that the additional experiments have strengthened the study and improved the manuscript. We are also grateful for the supportive comments regarding publication in Nature Communications. We have carefully considered the reviewer's suggestions and revised the manuscript accordingly to further improve the clarity and presentation of the work.

First, in the revised Fig. 3b, the authors have added Western blotting data to confirm VP22 expression following transient overexpression. While this demonstrates that VP22 protein is produced, western blotting has inherent limitations in this experimental context, as it cannot verify whether the VP22-expressing cells are identical to the productively infected cells. Given that the authors have now successfully generated and analyzed genetically defined VP22-deficient viruses in both the HF and F strain backgrounds, the transient VP22 overexpression data (Fig. 3b–e) are of reduced central importance. In contrast, the ΔVP22 virus data provide more direct and physiologically relevant evidence regarding the strain-dependent requirement of VP22. I therefore suggest moving the current Fig. 3b–e to the Extended Data section. Conversely, since one of the key conclusions of this study is the differential role of VP22 in HF versus F strains, the data for the F strain ΔVP22 mutant (currently shown in Extended Data Fig. 5e–g) would be more impactful if included in the main Figure 3. This rearrangement would facilitate direct comparison between strains and further clarify the distinct roles of VP22 in each viral context.

We thank the reviewer for this insightful suggestion regarding the presentation of the VP22-related data. We agree that the genetically defined ΔVP22 virus experiments provide more direct and physiologically relevant evidence for the strain-dependent role of VP22 during HSV-1 infection.

Following the reviewer's suggestion, we have reorganized the figures accordingly. The transient VP22 overexpression data (previously shown in Fig. 3b–e) have been moved to the Extended Data section, as they primarily serve as supportive validation of VP22 expression. Conversely, the data for the F strain ΔVP22 mutant (previously shown in Extended Data Fig. 5e–g) have now been incorporated into the main Figure 3, enabling clearer comparison between HF and F strain backgrounds.

We agree that this rearrangement improves the clarity of the presentation and more directly highlights the distinct roles of VP22 in the two viral contexts.

Second, although the authors' large-scale genomic analyses strongly support the presence of long poly(T) tracts in both laboratory strains and clinical isolates, many HSV-1 genome sequences deposited in public databases have undergone cell-culture passage. As such, statements describing these long poly(T) motifs as strictly "naturally occurring" may be somewhat overstated. While the data are clearly consistent with their presence in circulating viral populations, it remains difficult to completely exclude a contribution from cell-culture adaptation. I therefore recommend slightly softening the wording in the relevant sections (e.g., pages 14–15 of the revised manuscript), using phrasing such as "consistent with naturally occurring variation" or "present in both clinical and laboratory isolates," to avoid over-interpretation without diminishing the impact of the findings.

With these minor revisions, I believe the manuscript will be suitable for publication.

We thank the reviewer for this thoughtful suggestion. We agree that, although our genomic analyses support the presence of long poly(T) tracts in both laboratory strains and publicly available HSV-1 genomes, it is difficult to completely exclude the potential contribution of cell-culture passage in some database sequences.

Following the reviewer's recommendation, we have softened the wording in the relevant sections of the manuscript (pages 14–15). Specifically, we have revised the text to use more cautious phrasing such as "present in both clinical and laboratory isolates" and "consistent with naturally occurring variation," rather than describing these motifs as strictly "naturally occurring."

We appreciate this suggestion, which has helped us improve the accuracy and clarity of our interpretation without altering the overall conclusions of the study.
